# Divergent functionalization of alkenes enabled by photoredox activation of CDFA and α-halo carboxylic acids†

**DOI:** 10.1039/d4sc01084a

**Published:** 2024-06-06

**Authors:** Rahul Giri, Egor Zhilin, Dmitry Katayev

**Affiliations:** a Department of Chemistry, Biochemistry, and Pharmaceutical Sciences, University of Bern Freiestrasse 3 3012 Bern Switzerland dmitry.katayev@unibe.ch

## Abstract

Herein we present our studies on the solvent-controlled difunctionalization of alkenes utilizing chlorodifluoroacetic acid (CDFA) and α-halo carboxylic acids for the synthesis of γ-lactones, γ-lactams and α,α-difluoroesters. Mechanistic insights revealed that photocatalytic reductive mesolytic cleavage of the C–X bond delivers elusive α-carboxyl alkyl radicals. In the presence of an olefin molecule, this species acts as a unique bifunctional intermediate allowing for stipulated formation of C–O, C–N and C–H bonds on Giese-type adducts *via* single electron transfer (SET) or hydrogen atom transfer (HAT) events. These protocols exhibit great efficiency across a broad spectrum of readily available α-halo carboxylic acids and are amenable to scalability in both batch and flow. To demonstrate the versatility of this concept, the synthesis of (±)-boivinianin A, its fluorinated analog and eupomatilone-6 natural products was successfully accomplished.

## Abstract 2

The direct activation of α-halo carboxylic acids using visible-light-mediated photoredox catalysis facilitates the formation of bifunctional radical intermediates, allowing reactivity toward olefins to be fine-tuned by varying the solvent system.

## Graphical abstract

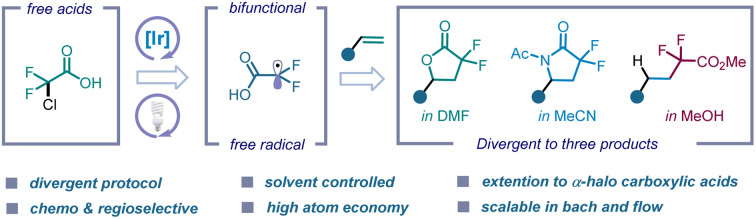

## Introduction

**Scheme 1:**
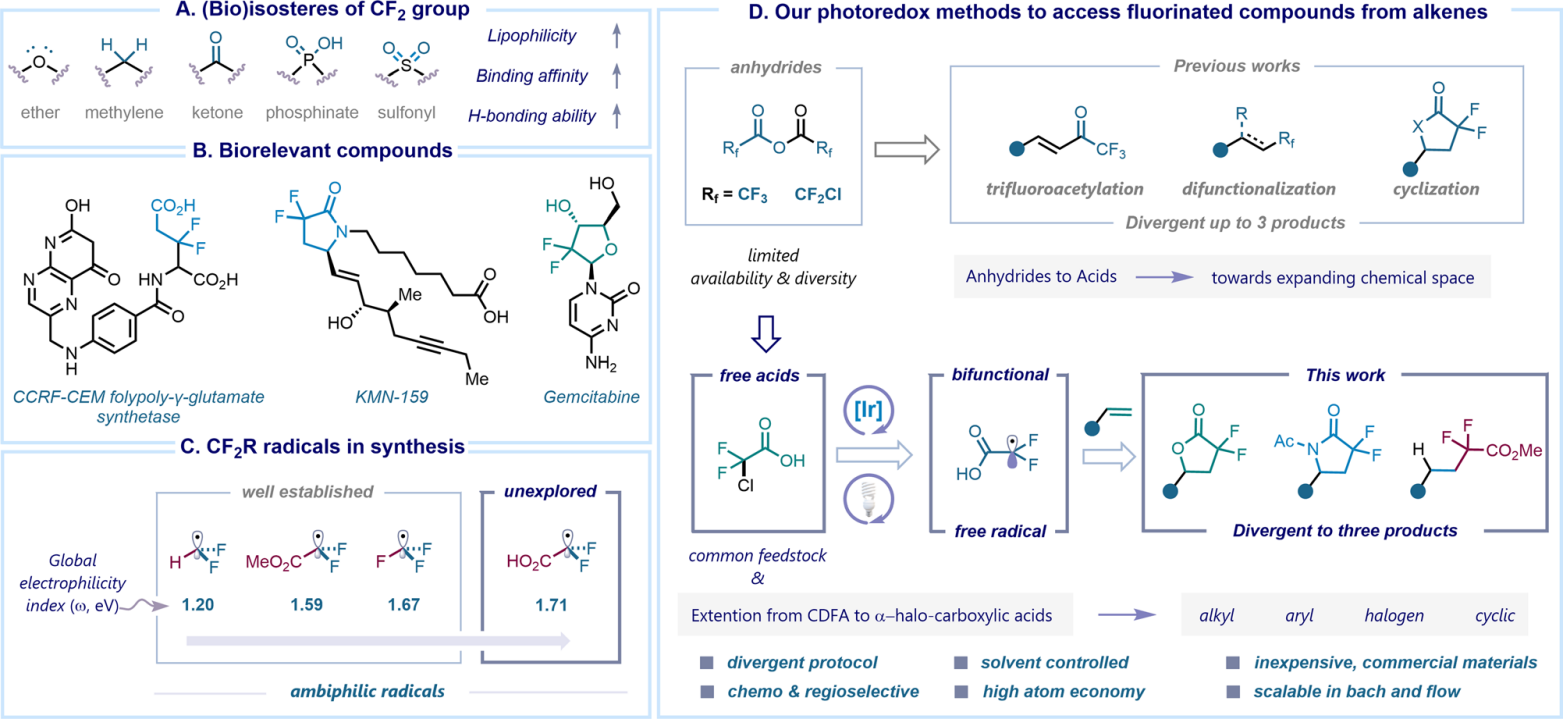
Introduction: (A) (bio)isostere of the CF_2_ group; (B) notable examples of *gem*-difluoro (CF_2_) containing biorelevant structures; (C) philicity trend of ˙CF_2_R free radicals; (D) our photoredox methods to access fluorinated compounds from alkenes.

Fluorine is a unique element present across various scientific disciplines, spanning from environmental science to medicinal chemistry. Its small atomic size, high electronegativity, and lipophilicity make this element an attractive choice for drug design and discovery. It is now estimated that about 20% of marketed drugs and 50% of agrochemicals contain at least one fluorine atom in the core of their structures.^1–4^ Incorporation of fluorine motifs as a bioisosteric replacement has recently become a valuable platform for the rapid modification of chemo-physical and pharmacological properties of a molecule. For instance, the difluoromethylene group (CF_2_) has often been considered as a bioisostere for ether, methylene, carbonyl, phosphinate, and sulfonyl functional groups to improve lipophilicity, binding affinity, and H-bonding ability (Scheme 1A).^5–7^ The widespread occurrence of the *gem*-difluoro (CF_2_) group in biologically active compounds highlights its potential for improving therapeutical outcomes.^8,9^ For example, *gem*-difluoro derivatives of difluoroglutamic acids have been identified as more favorable substrates for human CCRF-CEM folylpoly-γ-glutamate synthetase compared to non-fluorinated folic acid handles,^10^ while difluorolactam prostanoid EP_4_ receptor agonists such as KMN-159 exhibit five times greater potency than their methylene counterparts.^11^ Gemcitabine, a chemotherapy drug employed in the treatment of various cancers, is another representative molecule featuring a *gem*-difluoro (CF_2_) group (Scheme 1B).^12^

Notably, γ-lactones and γ-lactams are valuable and versatile functional groups commonly found in numerous pharmaceutical applications.^13–17^ Therefore, several classical approaches towards obtaining nonfluorinated γ-lactones and γ-lactams have been employed using esters, acids, and anhydrides in combination with olefins.^18–30^ Alongside, hydrofunctionalization of alkenes *via* hydrogen atom transfer (HAT) catalysis has gained considerable interest in recent years and accelerated the chemical space of hydro alkylated products.^31–34^ Merging the distinct structural features of *gem*-difluoro compounds with these molecules presents an exciting avenue for the design and development of novel drug candidates. In recent years, the preparation of difluoro-γ-lactones directly from alkenes has been explored, particularly utilizing photoredox catalysis. The Xi group reported a sequential protocol involving the hydroxydifluoroacetylation of alkenes using Chen's reagent (FSO_2_CF_2_CO_2_Me) followed by esterification in the presence of MgF_2_ (5.0 equiv.) as a base.^35^ Similarly, Xiao and co-workers enabled lactonization of alkenes with BrCF_2_CO_2_K reagent with the addition of over-stoichiometric amounts of boron-based Lewis acids (3.0 equiv.).^36^ Conversely, the utilization of pre-functionalized starting materials, such as cinnamyl alcohols^37^ or vinyl azides,^38^ also resulted in the generation of difluoro γ-lactones and γ-lactams respectively. In this context, our interest in the preparation of fluorinated molecules has led us to utilize readily available and stable fluorinated anhydrides as redox-active reagents for functionalizing unsaturated hydrocarbons. For example, we previously demonstrated the concentration-driven divergence to achieve trifluoromethyl (˙CF_3_) or trifluoroacetyl (˙CF_3_CO) radical species through the C–O bond cleavage of trifluoroacetic anhydride under photoredox catalysis.^39^ Additionally, we developed a visible light-mediated activation of chlorodifluoroacetic anhydride to accomplish the synthesis of *gem*-difluorinated γ-lactones and γ-lactams, while also promoting oxy-perfluoroalkylation reactions. The observed divergence was influenced by the distinct polarities exhibited by various solvents (Scheme 1D).^40^

Consequently, synthetic chemists are actively involved in the development of methodologies for the facile and straightforward incorporation of fluorine-containing fragments into organic structures. This dynamic branch of organofluorine chemistry is experiencing a rapid growth, with the core principle involving the generation of fluorinated radicals from readily available functional group transfer reagents (FGTRs).^41,42^ The reactivity of these radicals is often impacted by their philicity character, where a “polarity match” effect with the reaction partner plays a key role. In the latter scenario, a nucleophilic radical tends to preferentially add to an electron-poor reactive site, while an electrophilic radical selects an electron-rich center.^43,44^ In our recent theoretical studies, we observed a significant impact on the electrophilicity of free fluorinated radicals upon the introduction of electronegative atoms. For instance, the global electrophilicity index (*ω*) values of ˙CF_2_R radicals increase in the following order: ˙CF_2_H (*ω* = 1.20 eV), ˙CF_2_CO_2_Me (*ω* = 1.59 eV), ˙CF_3_ (*ω* = 1.67 eV) and ˙CF_2_CO_2_H (*ω* = 1.71 eV) (Scheme 1C).^45^ The slightly electrophilic nature of the former three radical species facilitated their widespread application in the synthesis of fluorinated compounds. Meanwhile, the distinctive feature of the ˙CF_2_CO_2_H radical, bearing two reactive centers in a single intermediate, an electrophilic radical and a nucleophilic carboxyl group, makes it an ideal bifunctional species.^46^ As the reactivity potential of the radical remains underdeveloped, additional studies are necessary to gain a comprehensive understanding of its chemical behavior.

Although anhydrides have shown a potential as suitable precursors for constructing cyclic *gem*-difluoro compounds in our previous studies, their origin lies in conventional reactions with carboxylic acids or acyl chlorides, which restricts their diversity and availability. In this work, we describe our efforts to overcome the challenge posed by the limited availability of anhydrides through leveraging chlorodifluoroacetic acid (CDFA), a commercially available feedstock, to a visible-light mediated photoredox catalysis, accessing unique *gem*-difluoro α-carboxy alkyl radicals (˙CF_2_CO_2_H).^47^ This intermediate serves as a bifunctional species that further reacts with alkenes, enabling a divergent synthesis^48^ of γ-lactones, γ-lactams and α,α-difluoroesters by simply switching the solvent system. Notably, the methodology can be extended to a variety of α-halo carboxylic acids, streamlining the preparation of structurally diverse γ-lactones and γ-lactams in an atom-economical and chemoselective manner (Scheme 1D). We also applied this approach to prepare the natural product (±)-boivinianin A and its fluorinated analogue, along with the development of a three-step synthesis of the eupomatilone-6. This procedure is scalable in both batch and flow, showcasing its synthetic robustness and versatility.

## Results and discussion

We started our exploration by determining the reduction potential of chlorodifloroacetic acid (CDFA) through cyclic voltammetry measurements, revealing a cathodic wave at a negatively shifted potential of −0.5 V *vs.* SCE. Initial experiments were conducted with 4-*tert*-butylstyrene in the presence of the *fac*-Ir(ppy)_3_ (
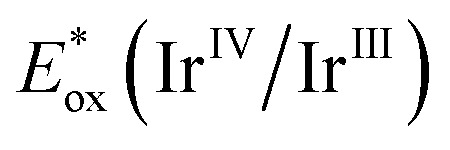
= −1.73 V *vs.* SCE) photocatalyst (PC) in dimethylformamide (DMF) using a blue LED emitting at 440 nm, resulting in the formation of the desired product 1 in up to 72% yield (Table 1, entry 1). Switching the solvent system to DMSO provided the product only in moderate yield (entry 2), however follow-up screening revealed the presence of product 2 in the reaction mixture with a yield of 21%, when performed in MeCN (entry 3). Encouraged by this preliminary observation, we conducted a comprehensive examination of the reaction conditions in order to acquire lactone (1), lactam (2) or HAT-product 3 in a selective manner (see ESI,† pages 5–12 for details). The variation of concentration, and the loading of acid and the photocatalyst were further investigated. It was found that a concentration of 0.5 M of MeCN, 2 equivalents of acid and 1 mol% of PC are optimal for the formation of γ-lactone 1 (entries 4–6). Utilization of different metals and organo-photocatalysts was found to be ineffective (entries 7–9). We then carefully evaluated the conditions to improve a three component Ritter-type reaction.^49^ Adjusting the concentration and the loading of the photocatalyst slightly enhanced the reaction efficiency (entries 10–12). Nevertheless, γ-lactone persisted as a side product in most cases. This can be attributed to the favored intramolecular reaction of the carboxyl group (–CO_2_H) in the reaction intermediate, which outpaces the comparatively slower addition of intermolecular MeCN. Overcoming this substantial challenge proved to be quite intricate. To decrease the nucleophilicity of the carboxylate group, we tested various additives. Notably, di-*tert*-butyl decarbonate (Boc_2_O, I) emerged as the optimal choice (entries 13–14). Additional assessment of the loading of I resulted in the formation of the desired γ-lactam 2 in a decent 58% yield, while formation of γ-lactone was almost suppressed (entry 15). Further exploration of alcohol-based solvents revealed distinctive reactivity, markedly observing hydro difluoroalkylation (3) of unactivated alkenes in the presence of sodium ascorbate as a hydrogen atom transfer (HAT) reagent (entries 16 and 17, for details see ESI,† page 12).

**Table 1:** Optimization table

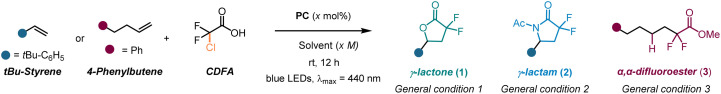						
Entry	PC (1 mol%)	Solvent	Concentration [M]	1^a^^,^^c^ (%)	2^b^^,^^c^ (%)	3^c^^,^^d^ (%)
1^e^	[Ir-1]	DMF	0.25	72	—	—
2^e^	[Ir-1]	DMSO	0.25	60	—	—
3^e^	[Ir-1]	MeCN	0.25	24	21	—
4^e^	[Ir-1]	DMF	0.50	82	—	—
**5**	**[Ir-1]**	**DMF**	**0.50**	**94 (90)**	**—**	**—**
6	[Ir-1]^f^	DMF	0.50	90		—
7	[Ru-1]	DMF	0.50	<5	—	—
8	[Cu-1]	DMF	0.50	8	—	—
9	Mes-Acr	DMF	0.50	0	—	—
10^e^	[Ir-1]	MeCN	0.17	23	29	—
11	[Ir-1]	MeCN	0.17	21	33	—
12	[Ir-1]^f^	MeCN	0.17	24	26	—
13	[Ir-1]^g^	MeCN	0.17	18	49	—
14	[Ir-1]^h^	MeCN	0.17	35	38	—
**15**	**[Ir-1]** i	**MeCN**	**0.17**	**9**	**58 (55)**	**—**
16	[Ir-1]^j^	MeOH	0.17	—	—	65
**17**	**[Ir-1]** j	**MeOH : MeCN (1 : 1 v/v)**	**0.17**	**—**	**—**	79
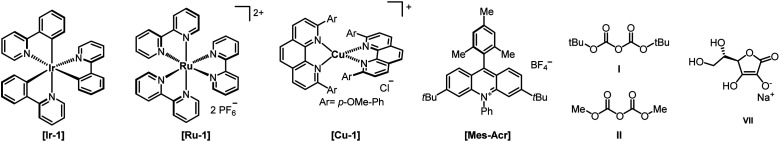						

a Conditions 1: alkene (1 equiv.), photocatalyst (1 mol%), CDFA (2 equiv.), DMF (*x* M), blue LEDs, rt, 12 h.

b Conditions 2: alkene (1 equiv.), *fac*-Ir(ppy)_3_ (1 mol%), CDFA (2 equiv.), MeCN (*x* M), Boc anhydride (*x* equiv.), blue LEDs, rt, 24 h.

c Conditions 3: alkene (1 equiv.), photocatalyst (1 mol%), CDFA (2 equiv.), VII (2 equiv.), MeOH : MeCN (1 : 1 v/v), blue LEDs, rt, 12 h.

d Yields of 1, 2 and 3 were determined by GC against *n*-decane as the internal standard.

e 1.1 equiv. of CDFA.

f 2 mol% of PC.

g 4 equiv. of I.

h 4 equiv. of II.

i 2 equiv. of I.

j 2 equiv. of VII.

**Scheme 2:**
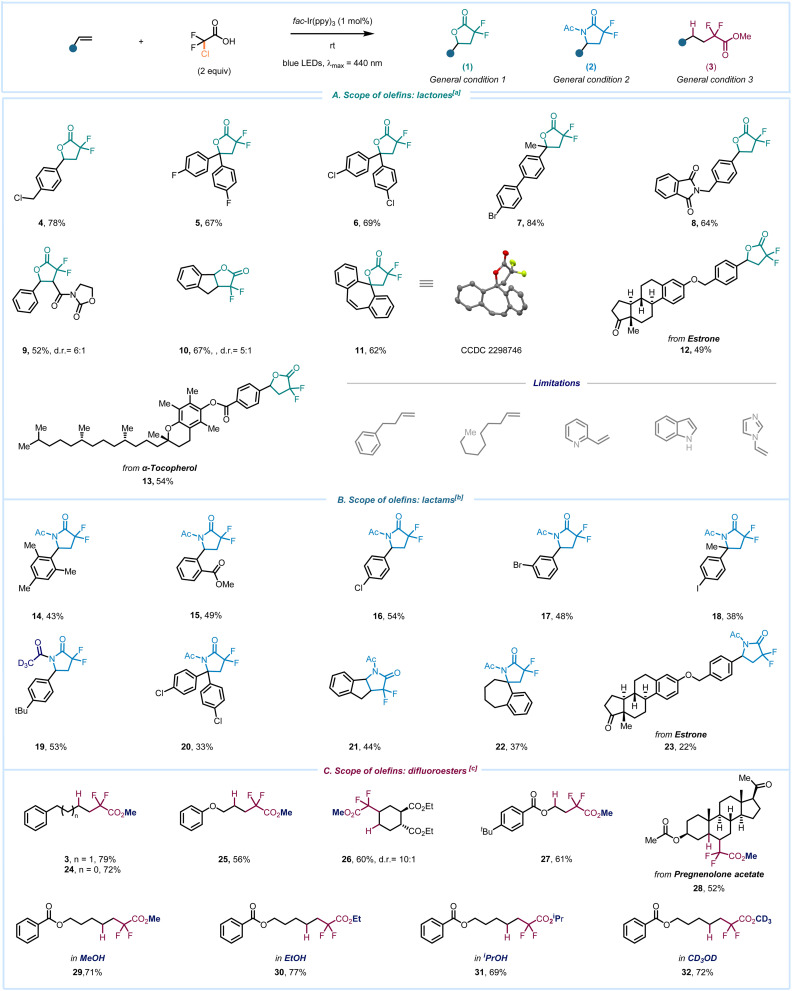
Reaction scope: [a] conditions 1: alkene (1 equiv.), *fac*-Ir(ppy)_3_ (1 mol%), CDFA (2 equiv.), DMF (0.5 M), blue LEDs, rt, 12 h. [b] Conditions 2: alkene (1 equiv.), *fac*-Ir(ppy)_3_ (1 mol%), CDFA (2 equiv.), MeCN (0.17 M), Boc anhydride (2 equiv.), blue LEDs, rt, 24 h. [c] Conditions 3: alkene (1 equiv.), photocatalyst (1 mol%), CDFA (2 equiv.), VII (2 equiv.), alcohol : MeCN (1 : 1 v/v), blue LEDs, rt, 12 h; (A) scope of γ-lactams. (B) Scope of γ-lactones. (C) Scope of α,α-difluoroesters.

After identifying optimal reaction conditions, we sought to explore the reaction scope with respect to styrene derivatives and to evaluate the compatibility with several functional groups (Scheme 2). We were pleased to find that the benzylic chlorine remains intact without undergoing fragmentation under our reaction conditions (4). Likewise, α,α-disubstituted olefins featuring F (5) or Cl (6) groups also yielded the product in good chemical yields, while the biphenyl group with bromo substitution furnished 7 with 84% yield. Other functional groups such as benzylic amide (8) or oxazolidin-2-one (9) also remained unaffected. Moreover, indene yielded the fused fluorinated lactone 10 in 67% yield.

Considering the prevalence of spirocyclic lactones in bioactive molecules,^50^ we extended this methodology to the [7]annulene core, and the structure of the product 11 was distinctly determined through single-crystal X-ray crystallography. Late-stage functionalization (LSF) emerges as an attractive strategy for incorporating functional groups at the end of the reaction sequence.^51^ Our method was utilized for LSF of a derivative of the natural steroid hormone estrone, resulting in the product 12. Similarly, the derivative of α-tocopherol, one form of vitamin E, readily underwent the reaction to yield fluorinated lactone 13. Having successfully investigated our strategy for synthesizing γ-lactones, we turned our attention to the synthesis of γ-lactams. Tri-substituted styrene furnished the desired product 14 in moderate yield. Essential functional groups and halogen substituents such as ester, -bromo, -chloro, and -iodo remained intact during photoredox reaction conditions, providing the corresponding adducts 15–18. The use of deuterated MeCN resulted in the formation of lactam 19 with isotope incorporation. α,α-Disubstituted styrene likewise yielded the product 20, and the lactam fused ring 21 was obtained with indene. The reaction was equally effective for the spirocyclic [7]annulene construction (22). Additionally, the olefin derived from estrone also underwent lactam formation 23, providing a compelling illustration of our divergent protocol.

Attempts to expand the reaction scope for lactone and lactam to include unactivated olefins were unfortunately unsuccessful, primarily attributed to the electrophilic nature of the α-carboxyl radical and its polarity mismatch that deter effective reaction with non-styrenes, and the inadequacy of the photocatalyst in oxidizing the generated Giese-type alkyl radical intermediates. This prompted us to delve deeper into the potential of this work, aiming to investigate the reactivity of unactivated olefins in polar solvents and intercept the resulting radical. To our delight, the hydrodifluoroalkylation process occurred in the presence of a HAT reagent. Olefins such as 4-phenylbutene and allylbenzene yielded the corresponding products 3 and 24 in good yields. Allyl ether, disubstituted cyclohexane, and phenyl benzoate delivered the α,α-difluoro methyl esters 25, 26 and 27, respectively. Pregnenolone acetate, a synthetic pregnane steroid, also underwent the reaction to provide 28, showcasing the applicability for the late-stage functionalization. To illustrate the extent of this protocol, we broadened its application to include various alcohol solvents, resulting in the synthesis of diverse alkylated difluoro compounds (29–32). Notably, the protocol enabled the synthesis of a deuterated ester in methanol d_4_ in a single step highlighting the uniqueness of this approach.

**Scheme 3:**
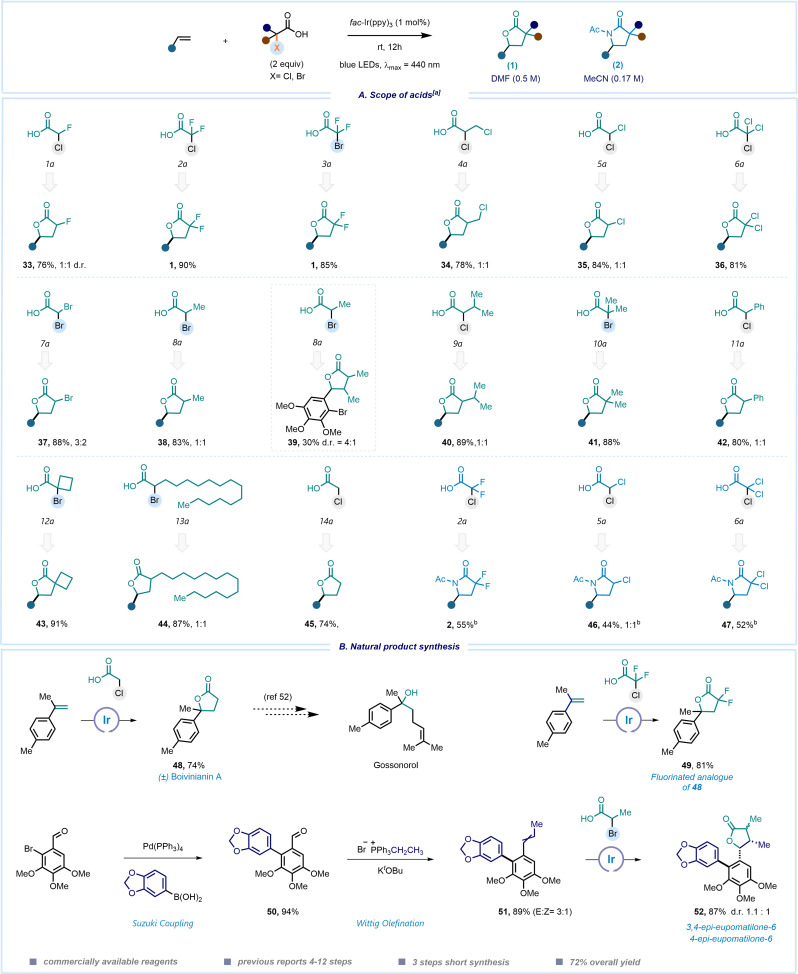
Reaction scope: [a] conditions 1: alkene (1 equiv.), *fac*-Ir(ppy)_3_ (1 mol%), acid (2 equiv.), DMF (0.5 M), blue LEDs, rt, 12 h; [b] conditions 2: alkene (1 equiv.), *fac*-Ir(ppy)_3_ (1 mol%), acid (2 equiv.), MeCN (0.17 M), Boc anhydride (2 equiv.), blue LEDs, rt, 24 h; (A) scope of α-halo carboxylic acids; (B) natural product synthesis.

After exploring the scope of olefins, we attempted to integrate these reaction conditions into a wide spectrum of commercially accessible α-halo carboxylic acids (Scheme 3). Remarkably, among the 17 acids subjected to testing, 15 effectively produced the desired product under our photoredox reaction conditions (see ESI,† page 14 for details). We employed 4-*tert*-butylstyrene as a model substrate to assess the feasibility of various fluorine-containing acids 1a–3a which underwent the desired transformation in good yields to produce 33 and 1. Consequently, when we employed acids 4a–6a, different chlorine-substituted lactones were obtained (34–36). Dibromoacetic acid yielded the corresponding α-bromo lactone 37. Overall, this approach offers an operationally simple method to rapidly access γ-lactones or lactams bearing one or two halogen atoms at the alpha-position, facilitating their use in post-functionalization and integration into complex molecule synthesis. Subsequently, we explored alkyl and phenyl substituted acids 9a–12a, which also gave the products in very good yields (39–42). When using a highly oxygenated aromatic ring with substitutions at the *ortho*-, *meta*-, and *para*-positions in the reaction with 2-bromopropanoic acid (9a), the desired product 39 was also isolated, however in moderate 30% yield. Interestingly, sterically hindered α-alkyl substituted carboxylic acids 13a and 14a demonstrated excellent reactivity similar to that of non-substituted chloroacetic acid 15a in the synthesis of lactones. Additionally, we explored the scope of acids for accessing lactams. Although the yields were moderate, we successfully synthesized lactams with *gem*-difluoro-, mono-chloro, and dichloro-substituents in the lactam ring (2, 46, 47, respectively).

Demonstrating the scope of the reaction, we next envisioned to explore our methodology for the total synthesis of natural products. The effectiveness of our mild protocol is highlighted by its application in the one-step synthesis of (±)-boivinianin A (48) from all readily available materials. Notably, compound 48 was also already used as a key intermediate for the preparation of gossonorol and other natural products.^52,53^ This method was also applied to synthesize the fluorinated analogue of 48, underscoring the versatility and practicality of the employed method. Our attention was then turned to another important class of natural products – eupomatilones. In 1991, Carroll and Taylor isolated eupomatilones from the Australian shrub *Eupomatia bennettii*. A variety of structurally diverse lignan natural products coexist with these compounds.^54^ The structural complexity of eupomatilones presents an enticing synthetic challenge due to their tri-substituted chiral γ-lactone motifs and highly oxygenated biaryl skeletons. Therefore, multiple asymmetric and racemic methods have been documented, often involving longer reaction sequences ranging from 4 to 12 steps.^55–61^ We commenced the synthesis by opting for 2-bromo-3,4,5-trimethoxybenzaldehyde as the starting point. The Suzuki cross-coupling of this compound with 3,4-(methylenedioxy)-phenylboronic acid resulted in the formation of the highly oxygenated product 50. In the subsequent step, the aldehyde group was transformed into an olefin using a Wittig olefination reaction, yielding product 51 as a mixture of *E*/*Z* isomers (3 : 1). Consequently, 51 was subjected to the lactonization step, resulting in the formation of 52 as a mixture of 3,4-*epi*-eupomatilone-6 and 4-*epi*-eupomatilone-6 in 1.1 : 1 ratio. In this study, we were able to access a complex molecule in only three steps using all commercially available materials. This method offers a radical approach for the late-stage functionalization, providing a straightforward, cost-effective, and sustainable alternative to conventional ionic pathways.

**Scheme 4:**
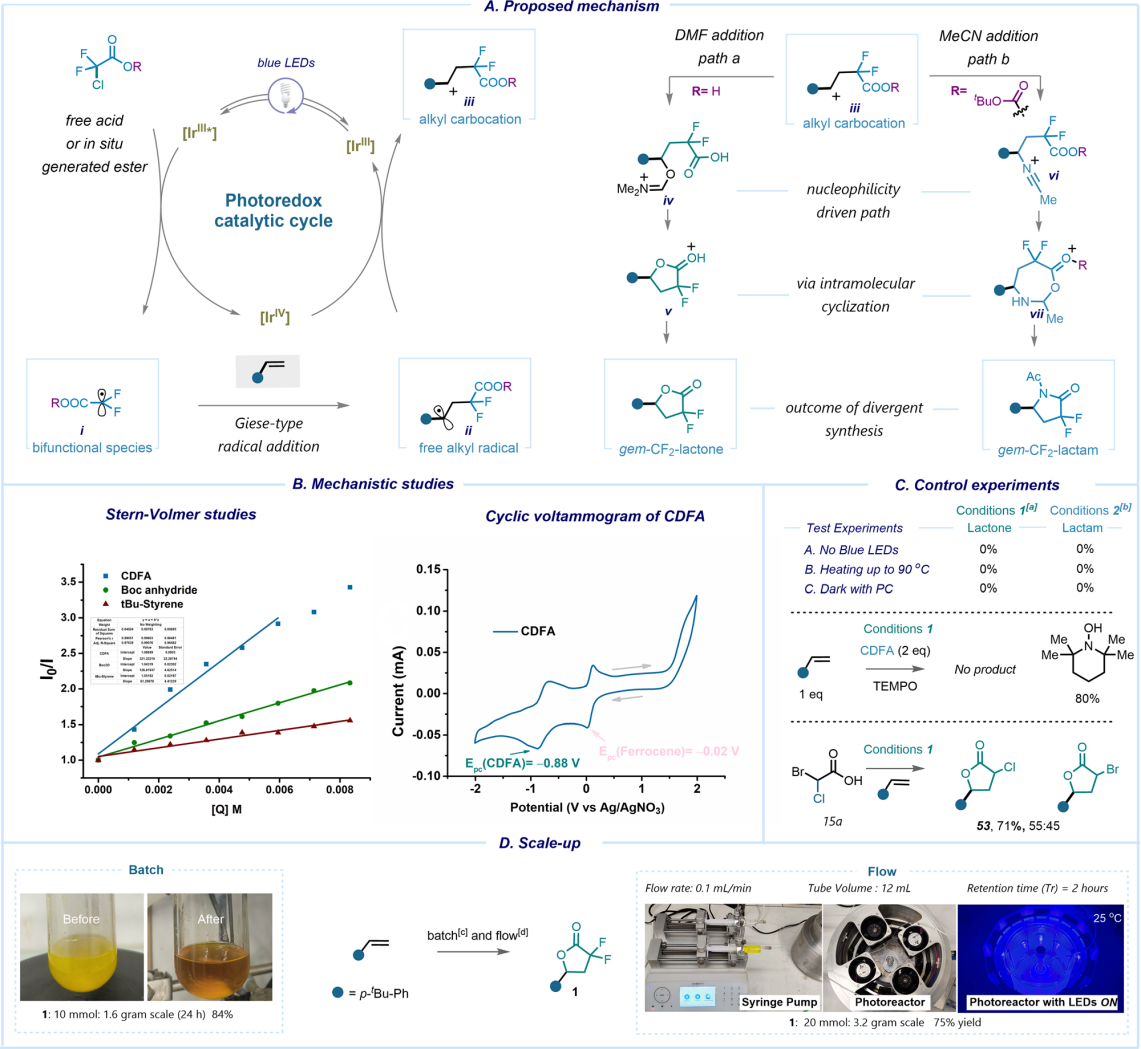
Proposed mechanism: (A) proposed mechanism; (B) mechanistic studies towards formation of 1 and 2; (C) control experiments; (D) batch and flow scale-up. [a] Conditions 1: alkene (1 equiv.), *fac*-Ir(ppy)_3_ (1 mol%), CDFA (2 equiv.), DMF (0.5 M), blue LEDs rt, 12 h; [b] conditions 2: alkene (1 equiv.), *fac*-Ir(ppy)_3_ (1 mol%), CDFA (2 equiv.), MeCN (0.17 M), Boc anhydride (2 equiv.), blue LEDs, rt, 12 h; [c] alkene (10 mmol), *fac*-Ir(ppy)_3_ (0.1 mmol), CDFA (20 mmol), DMF (10 mL), blue LEDs, rt, 24 h, isolated yields; [d] alkene (20 mmol), *fac*-Ir(ppy)_3_ (0.2 mmol), CDFA (40 mmol), DMF (20 mL), flow rate = 0.3 mL min^−1^, *τ*_R_ = 2 h, isolated yields.

Our curiosity then led us to elucidate the mechanistic pathway of this photoredox process *via* experimental and spectroscopic techniques (Scheme 4A). Our investigation commenced with control experiments, revealing the pivotal roles of both the photocatalyst and light in our transformation. Notably, we observed no reaction taking place under heating conditions at 90 °C with or without the PC (Scheme 4C). Preliminary evidence indicates that introduction of the radical scavenger, 2,2,6,6-(tetramethylpiperidin-1-yl)oxyl (TEMPO), prevented the formation of the desired products (Scheme 4C) suggesting a likely radical pathway. In our reaction, the use of acids substituted either at α-chloro or α-bromo demonstrated nearly identical efficiency. This assertion was confirmed *via* a competition experiment utilizing acid 15a, which led to a product mixture comprising both α-chloro lactone and α-bromo lactone. There was a slight preference for the former, indicating that C–Br cleavage occurs faster than C–Cl cleavage (Scheme 4C). We propose that the reaction proceeds through excitation of photocatalyst *fac*-Ir(ppy)_3_ in the presence of high intensity blue LED irradiation. Owing to the low reduction potential *E*_0_(Ir^IV^/Ir^III*^) = −1.73 V (*vs.* SCE), the excited state of the photocatalyst readily reduces the chlorodifluoroacetic acid having an onset potential of −0.5 V (*vs.* SCE) *via* single electron transfer (SET). The consecutive C–Cl bond mesolytic cleavage occurs with the release of the electrophilic α-carboxyl radical (i) (*ω* = 1.71 eV). This is further supported by Stern–Volmer quenching analysis, which demonstrated an efficient quenching of the excited T_1_-state of Ir^III^ by CDFA (*K* = 1.7 × 10^8^ M^−1^ s^−1^) (Scheme 4B, see ESI,† pages 19–21). Notably, the quenching efficiency was lower for other reagents. After the formation of radical i, it undergoes a Giese-type addition reaction with the olefin molecule, resulting in the generation of a benzylic alkyl radical intermediate. The catalytic cycle closes with the subsequent radical oxidation to the benzylic carbocation iii and regeneration of the photocatalyst Ir^III^. In the presence of DMF, we assume the formation of iminium species iv, while in the presence of MeCN a Ritter-type addition takes place with the generation of vi. In the latter case, to reduce the nucleophilicity of the –CO_2_H group, it is necessary to utilize di-*tert*-butyl dicarbonate *in situ* to selectively protect the carboxylate. Subsequent intramolecular cyclization and rearrangement lead to the formation of *gem*-difluorolactone or *gem*-difluorolactam adducts. In contrast to SET-assisted oxidative cyclization to form lactone or lactam derivatives, the presence of sodium ascorbate allows HAT^30,63^ on Giese-type intermediate ii,^62,64^ resulting in the formation of α,α-difluoroesters. The excess of ascorbate further facilitates the restoration of the Ir-based photocatalyst, thereby completing the catalytic cycle (see ESI,† page 26). Notably, employing styrenes in the HAT-mediated protocol primarily leads to lactone formation, showcasing the predominance of the SET event over HAT for activated olefins, and *vice versa* for unactivated alkenes.

In order to enhance the applicability of our protocol, we sought to investigate its scalability (Scheme 4D). When employing a batch process with an extended reaction time of 24 hours, we achieved an 84% yield of 1. To improve the efficiency and to reduce the reaction time, we implemented this photoredox process in an in-house designed flow reactor, resulting in a shorter retention time of 2 hours and a product yield of 75%.

## Conclusions

In summary, we have successfully demonstrated the activation of CDFA and α-halo carboxylic acids through visible-light-mediated photoredox catalysis, enabling the generation of an electrophilic and bifunctional radical intermediate, the reactivity of which with olefins can be further adjusted by the nature of the solvent system. This simple and divergent protocol utilizing CDFA or α-halogenated acetic acids features the choice of C–O (lactones), C–N (lactams) and C–H (HAT) bond construction. Notably, there is no requirement for pre-functionalization of the initial α-halo carboxylic acids as a distinct chemical step. This approach considerably advances the toolbox of modern synthetic transformations, enabling readily accessible acids and olefin feedstocks to be converted into value-added γ-lactams, γ-lactones and α,α-difluoroesters. The synthesis of several complex frameworks including biologically active molecules has been accomplished. The robustness and scalability of the method have been demonstrated in both batch and flow setups. Follow up applications of such bifunctional intermediates for the synthesis of fluorinated compounds are currently underway in our laboratory.

## Data availability

All data supporting the fndings of this study are available within the article and its ESI file.†

## Author contributions

D. K. and R. G. designed and conceived the project. R. G. and E. Z. performed the experiments and wrote the ESI. All authors wrote the manuscript.

## Conflicts of interest

There are no conflicts to declare.

## Supplementary Material

SC-015-D4SC01084A-s001

SC-015-D4SC01084A-s002
