# Supplementary material for: Divergent functionalization of alkenes enabled by photoredox activation of CDFA and α-halo carboxylic acids
Source: Chem Sci. 2024 Jun 6;15(27):10659–67. doi: 10.1039/d4sc01084a (PMC11234866; doi:10.1039/d4sc01084a)
Supplement: SC-015-D4SC01084A-s001 [file SC-015-D4SC01084A-s001.pdf]

Electronic Supplementary Material (ESI) for Chemical Science.
This journal is © The Royal Society of Chemistry 2024

Supporting Information

Divergent Functionalization of Alkenes Enabled by Photoredox Activation of CDFA and α-Halo Carboxylic Acids

Rahul Giri,[a] Egor Zhilin,[a] Dmitry Katayev[a]*

a. University of Bern, Department of Chemistry, Biochemistry, and Pharmaceutical Sciences, Freiestrasse 3, 3012 Bern

* Corresponding author

Email: dmitry.katayev@unibe.ch

Table of contents

1. Material and methods ---------- 3

1.1. High intensity photoreactors ---------- 4

2. Reaction development ---------- 5

2.1. Solvent effect ---------- 5

2.2. Concentration effect ---------- 6

2.3. Equivalents of acid ---------- 7

2.4. Photocatalyst screening ---------- 8

2.5. fac-Ir(ppy)$_3$ loading ---------- 9

2.6. Additive screening for lactams ---------- 10

2.7. Loading of additive ---------- 10

2.8. Control experiments ---------- 11

2.9. Optimization of hydro gem-difluoro esterification (3) ---------- 12

3. General procedures ---------- 13

4. Availability of starting materials ---------- 14

5. Cyclic voltammetry ---------- 15

6. Radical trapping experiment ---------- 22

7. Competition experiment ---------- 23

8. Scale-up ---------- 24

9. Proposed mechanism for product 3 ---------- 26

10. Computational parameters ---------- 26

11. X-Ray Data ---------- 51

12. NMR spectra of isolated compounds ---------- 69

13. References ---------- 217

1. Material and methods

All reactions were performed in flame-dried glassware under an argon atmosphere containing a teflon-coated stirring bar and dry septum. In addition, glassware was dried overnight at 150 °C before use. Starting materials are commercially available and were purchased from Thermoscientific – Acros, Sigma Aldrich, Apollo Scientific, Fluorochem, and TCI unless otherwise noted. Anhydrous acetonitrile was distilled over CaH_2 and stored over pre-conditioned 3 Å mol sieves for at least 12 h before use. Analytical thin-layer chromatography (TLC) was performed on Merck silica gel 60 F254 TLC glass plates and visualized with 254 nm light and potassium permanganate staining solutions followed by heating when required. Purification of reaction products was carried out by flash chromatography using Brunschwig silica 32-63, 60Å under 0.3-0.5 bar overpressure. Medium pressure liquid chromatography (MPLC) was performed on a CombiFlash R_f200 System from Teledyne ISCO with a built-in UV-detector and fraction collector or manually using silica gel SilicaFlash P60, 40-63 µm. Teledyne ISCO RediSep Rf flash columns used having 0.035–0.070 mm particle size and 230–400 mesh. Normal phase preparatory HPLC purification was conducted on a Teledyne Isco CombiFlash EZ Prep system using a Macherey-Nagel VP 250/21 Nucleosil 50-5 columns. ^{1}H- and ^{13}C-NMR spectra were recorded on Bruker Ultrashield 300 (operating at 300.1 MHz and 75.5 MHz, respectively), Bruker Ascend 400 (operating at 400.1 MHz and 100.6 MHz, respectively), Bruker AVANCE III 500 (operating at 500.1 MHz and 125.6 MHz, respectively), ^{19}F-NMR spectra on Bruker DPX-300 and Bruker Ultrashield 300 (at 282 MHz) and Bruker DPX-400 and Bruker Ascend 400 (at 376 MHz) Bruker DPX-500 and Bruker AVANCE III 500 (at 477 MHz). The chemical shifts are reported in parts per million (ppm) and coupling constants (*J*) are given in Hertz (Hz). ^{1}H-NMR spectra are reported with the solvent resonance as the reference unless noted otherwise ($CDCl_3$ at 7.26 ppm). Peaks are reported as (s = singlet, d = doublet, t = triplet, q = quartet, m = multiplet or unresolved, coupling constant(s) in Hz, integration). ^{13}C-NMR spectra were recorded with ^{1}H-decoupling and are reported with the solvent resonance as the reference unless noted otherwise ($CDCl_3$ at 77.16 ppm). ^{19}F-NMR spectra were recorded with ^{1}H-decoupling unless noted otherwise. A Bruker Tensor III spectrometer equipped with a golden gate was used to record infrared spectra HR-MS (ESI^+) mass spectra were measured on a Bruker FTMS 4.7T BioAPEX II and Thermo Scientific LTQ Orbitrap XL equipped with a static nanospray ion source and mass spectrometry service operated on VG-TRIBRID for electron impact ionization (EI), or Varian IonSpec Spectrometer for electrospray ionization (ESI) and are reported as (*m/z,*). Electron impact ionization mass spectra (EI-MS) were run on a gas chromatography – mass spectrometry (GC-MS) instrument of Agilent 8890 series GC system and Agilent 5977B GC/MSD. Single crystals for were made on a RIGAKU Synergy S area-detector diffractometer using mirror optics monochromated Cu Kα radiation (λ = 1.54184 Å). Fluorescence spectroscopy was measured FS5 Spectrofluorometer from Edinburgh Instruments. Cyclic voltammetry was measured using the Osilla potentiostat, an Ag^+ (0.01M $AgNO_3$)/Ag reference electrode, a platinum disc working electrode, and a platinum counter electrode. All measurements were carried out in MeCN (0.1 M NBu_4PF_6) if not stated otherwise.

1.1. High intensity photoreactors

The photoreactor was custom designed and built by Katayev and co-workers in collaboration with the mechanical workshop at the Department of Chemistry and Applied Biosciences at ETH Zürich. It features blue LEDs arranged in a circular pattern, meticulously powered by a 10.3 A power supply, generating 300 W of light. The emitted light spectrum was precisely characterized through UV-Vis spectroscopy (Figure S2). To prevent overheating, the LEDs are water-cooled and equipped with built-in fans to regulate ambient temperature effectively.

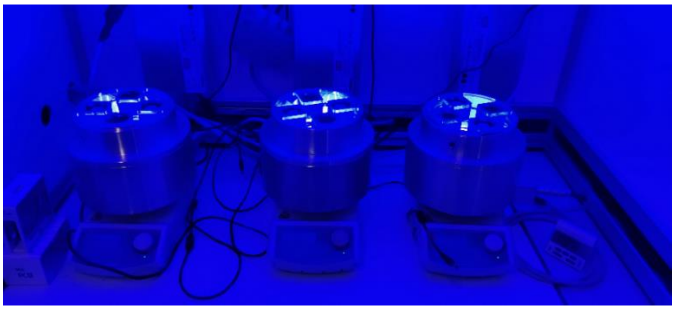

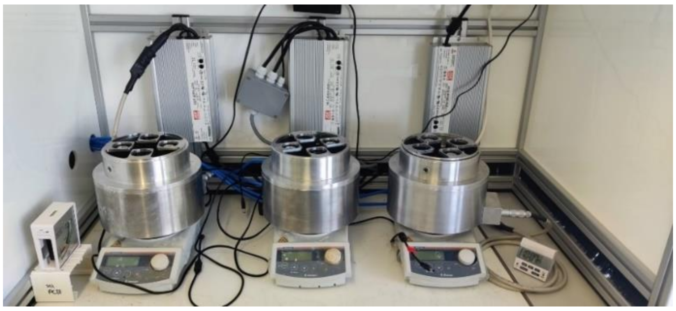

Figure S1. Custom high-intensity, blue LED photoreactors for photocatalytic reactions.

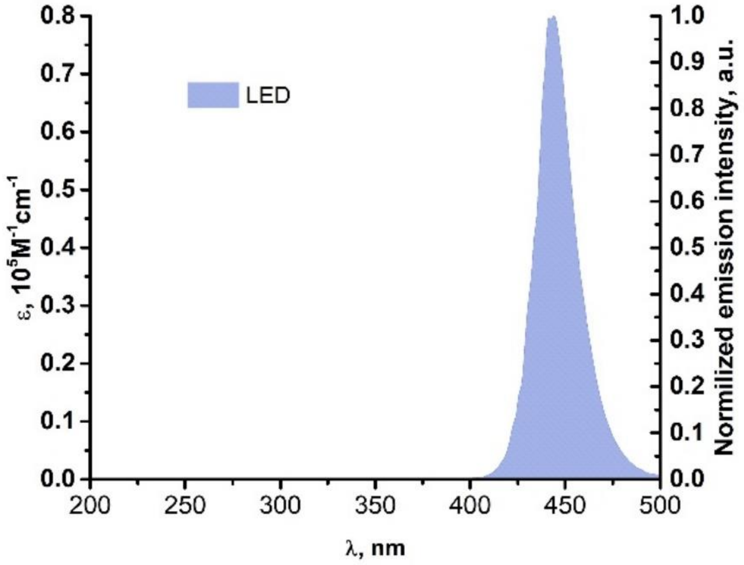

Figure S2. UV-Vis emission spectrum of high-intensity, blue LED photoreactor (λ_{max} = 440 nm, FWHM = 20 nm).

2. Reaction development

2.1. Solvent effect

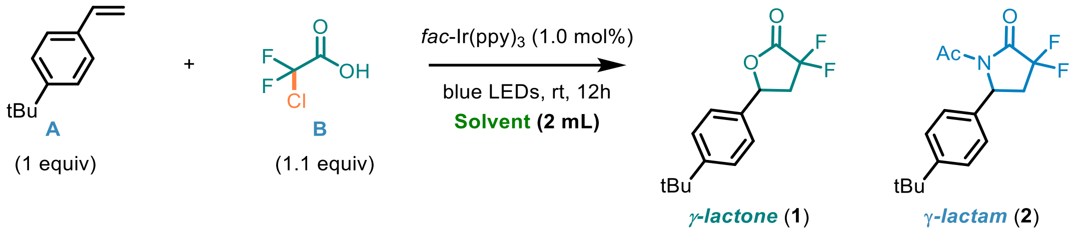

Entry[a]	Solvent	Yield of 1 [%][b]	Yield of 2 [%][b]
1	EtOAc	20	-
2	DMF	72	-
3	THF	21	-
4	$CHCl_3$	-	-
5	Isopropanol	1	-
6	Et_2O	-	-
7	DMSO	60	-
8	DCM	1	-
9	HFIP	-	-
10	1,2 DCE	-	-
11	Acetone	-	-
12	MeCN	24	21
13	Methanol	14	-
14	2,2,2 Trifluoroethanol	-	-

Table S1. a. Reaction conditions:4-*tert*-butylstyrene (1.0 equiv), *acid* B (1.1 equiv), *fac*-Ir(ppy)$_3$ (1.0 mol%), solvent (0.25 M) **b.** Yield of **1** and **2** are determined by GC against an internal standard of *n*-decane.

2.2. Concentration effect

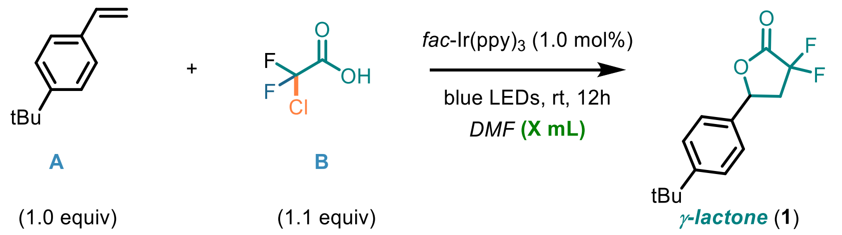

Entry[a]	Substrate [M]	Volume (ml)	Yield of 1 [%][b]
1	1	0.5	80
2	0.50	1	82
3	0.17	3	74
4	0.10	5	76
5	0.05	10	78
6	0.033	15	80

Table S2 a. Reaction conditions: 4-*tert*-butylstyrene (1.0 equiv), *acid* B (1.1 equiv), *fac*-Ir(ppy)$_3$ (1.0 mol%) and DMF(x M). **b.** Yield of **1** is determined by GC against an internal standard of *n*-decane.

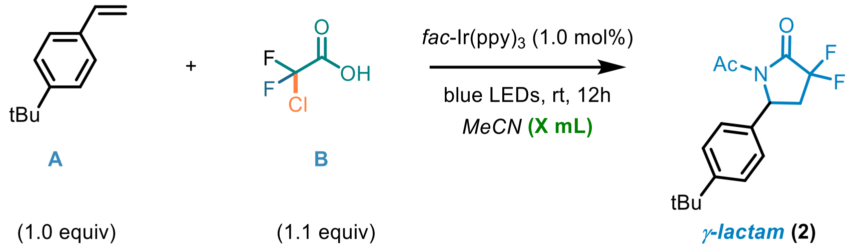

Entry[a]	Substrate [M]	Volume (ml)	Yield of 1 [%][b]	Yield of 2 [%][b]
1	1	0.5	30	13
2	0.50	1	24	26
3	0.17	3	23	29
4	0.10	5	32	23
5	0.05	10	28	traces
6	0.033	15	39	traces

Table S3 a. Reaction conditions: 4-*tert*-butylstyrene (1.0 equiv), *acid* B (1.1 equiv), *fac*-Ir(ppy)$_3$ (1.0 mol%) and MeCN (x M). **b.** Yield of **1** and **2** are determined by GC against an internal standard of *n*-decane.

2.3. Equivalents of acid

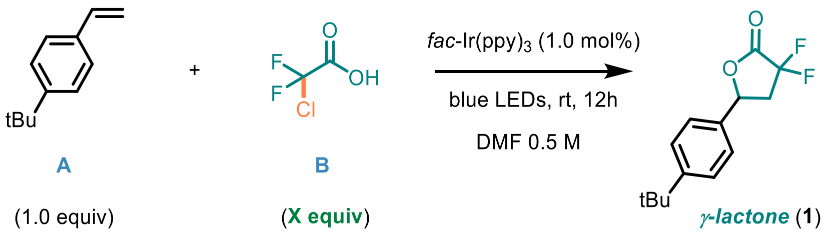

Entry[a]	Equivalent	Yield of 1 [%][a]
1	1	80
2	1.1	82
3	1.5	90
4	2	94
5	3	76
6	5	78

Table S4. a. Reaction conditions: 4-*tert*-butylstyrene (1.0 equiv), *acid* B (x equiv), *fac*-Ir(ppy)3 (1.0 mol%) and solvent. **b.** Yield of **1** is determined by GC against an internal standard of *n*-decane. **c.** DMF is used as solvent. **d.** MeCN is used as solvent.

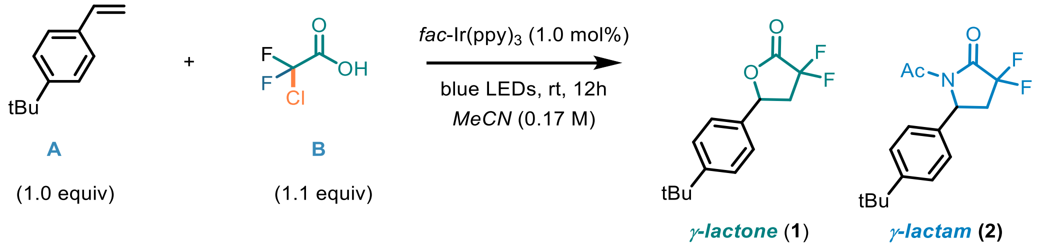

Entry[a]	Equivalent	Yield of 1 [%][b]	Yield of 2 [%][b]
1	1	24	26
2	1.1	23	29
3	1.5	26	31
4	2	21	33
5	3	36	18
6	5	45	12

Table S5. a. Reaction conditions: 4-*tert*-butylstyrene (1.0 equiv), *acid* B (x equiv), *fac*-Ir(ppy)3 (1.0 mol%) and MeCN (0.17 M). **b.** Yield of **1** and **2** are determined by GC against an internal standard of *n*-decane.

2.4. Photocatalyst screening

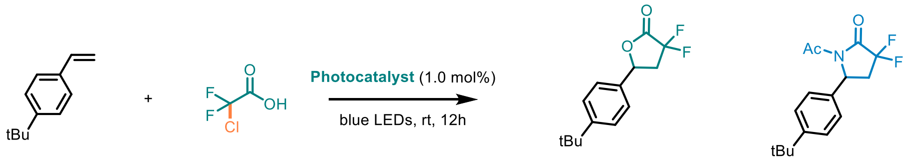

A	B	γ-lactone (1)	γ-lactam (2)
(1.0 equiv)	(2.0 equiv)	in DMF (0.5 M)	in MeCN (0.17 M)

Photocatalyst [a,b]

	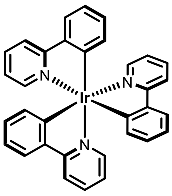	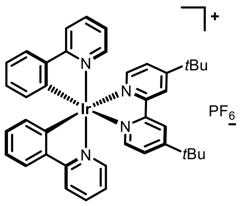	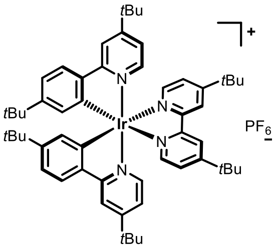	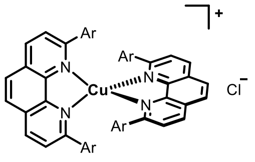
	***fac*-[Ir(ppy)$_3$]**	**[Ir(dtbbpy)(ppy)$_2$]PF$_6$**	**[Ir(bpy)$_3$tBu]PF$_6$**	**[Cu(dap)$_2$Cl]** (Ar= *p*-OMe-C_6H_4)
	CAS: 94928-86-6	CAS: 676525-77-2	CAS: 808142-80-5	CAS: 1392109-17-9
1	94%	12%	34%	8%
2	33%	10%	2%	5%

	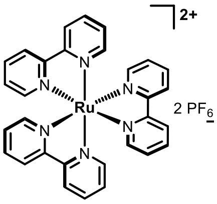	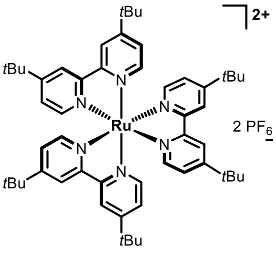	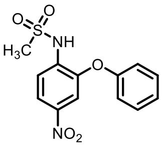	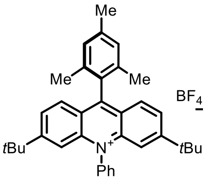
	**Ru(bpy)$_3$(PF$_6$)$_2$**	**[Ru(dtbbpy)$_3$](PF$_6$)$_2$**	**Nimesulide**	**Mes-Acr**
	CAS: 60804-74-2	CAS: 75777-87-6	CAS: 51803-78-2	CAS: 1810004-87-5
1	traces	0%	0%	0%
2	-	0%	0%	traces

	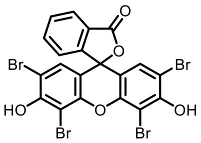	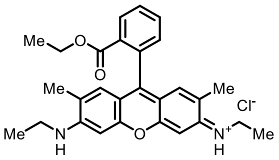
	**Eosin Y**	**Rhodamine 6G**
	CAS: 7372-87-1	CAS: 989-38-8
1	0%	0%
2	-	0%

Table S6. a. Reaction conditions: 4-*tert*-butylstyrene (1.0 equiv), *acid* B (2.0 equiv), photocatalyst (1.0 mol%) and solvent. **b.** Yield of **1** and **2** are determined by GC against an internal standard of *n*-decane.

2.5. *fac*-Ir(ppy)3 loading

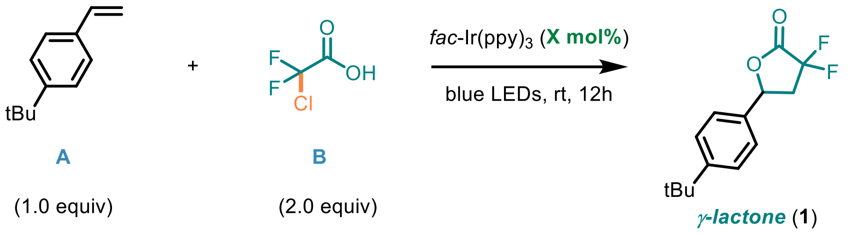

Entry[a]	mol %	Yield of 1 [%][b]
1	1	94
2	2	90
3	5	93
4	10	91
5	50	72
6	100	79

Table S7. a. Reaction conditions: 4-*tert*-butylstyrene (1.0 equiv), *acid* B (2.0 equiv), *fac*-Ir(ppy)3 (1.0 mol%) and solvent. **b.** Yield of **1** is determined by GC against an internal standard of *n*-decane.

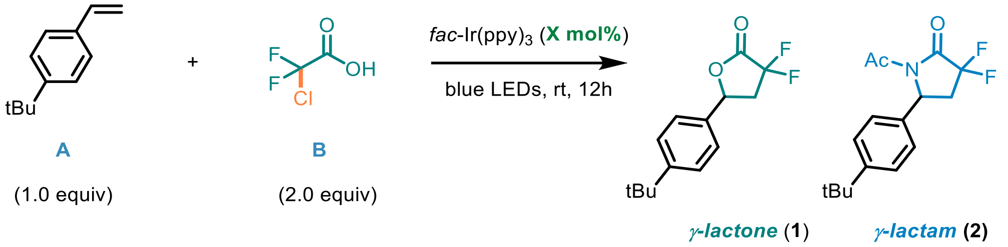

Entry[a]	mol %	Yield of 1 [%][b]	Yield of 2 [%][b]
1	1	21	33
2	2	24	26
3	5	25	29
4	10	31	36
5	50	30	24
6	100	24	12

Table S8. a. Reaction conditions: 4-*tert*-butylstyrene (1.0 equiv), *acid* B (2.0 equiv), *fac*-Ir(ppy)3 (1.0 mol%) and solvent. **b.** Yield **1** of **2** are determined by GC against an internal standard of *n*-decane.

2.6. Additive screening for lactams

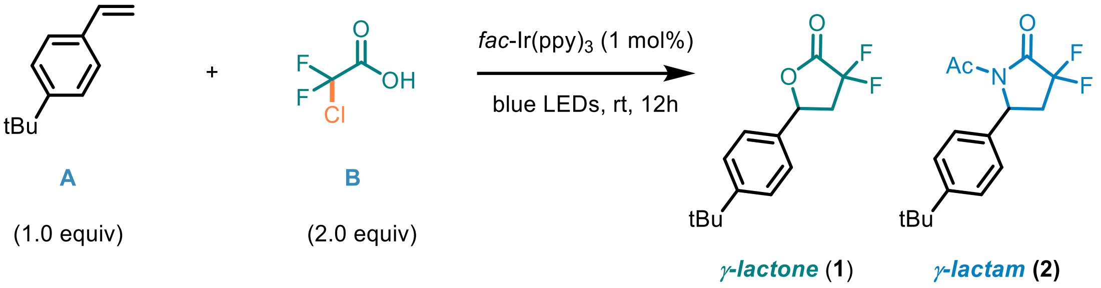

Additive (*4 equiv*)

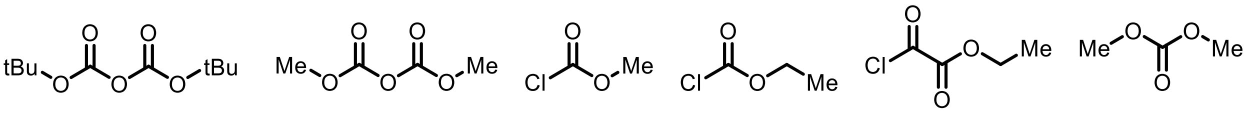

I **II** **III** **IV** **V** **VI**

Entry[a]	Additive	Yield of 1 [%][b]	Yield of 2 [%][b]
1	I	18	49
2	II	35	38
3	III	9	5
4	IV	10	7
5	V	10	10
6	VI	3	traces

Table S9. a. Reaction conditions: 4-*tert*-butylstyrene (1.0 equiv), *acid* B (2.0 equiv), *fac*-Ir(ppy)$_3$ (1.0 mol%) and MeCN (0.17 M). **b.** Yield of **1** and **2** are determined by GC against an internal standard of *n*-decane.

2.7. Loading of additive

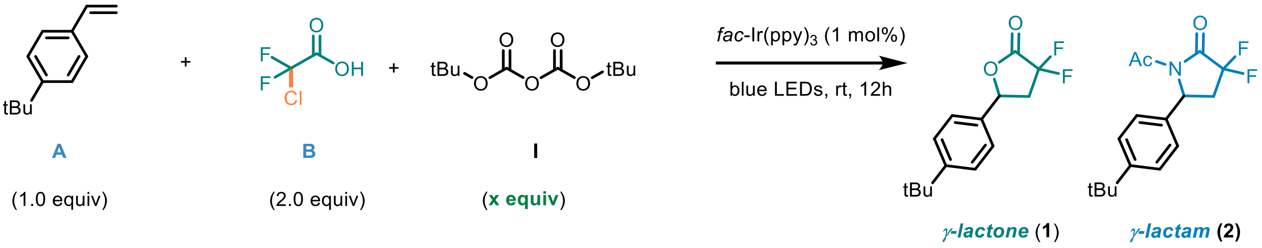

Entry[a]	Equivalent	Yield of 1 [%][b]	Yield of 2 [%][b]
1	0.5	17	35
2	1.0	13	54
3	2.0	9	58
4	3.0	10	51
5	5.0	16	46
6	10.0	9	12

Table S10. a. Reaction conditions: 4-*tert*-butylstyrene (1.0 equiv), *acid* B (2.0 equiv), *fac*-Ir(ppy)$_3$ (1.0 mol%) and MeCN (0.17 M). **b.** Yield of **1** and **2** are determined by GC against an internal standard of *n*-decane.

2.8. Control experiments

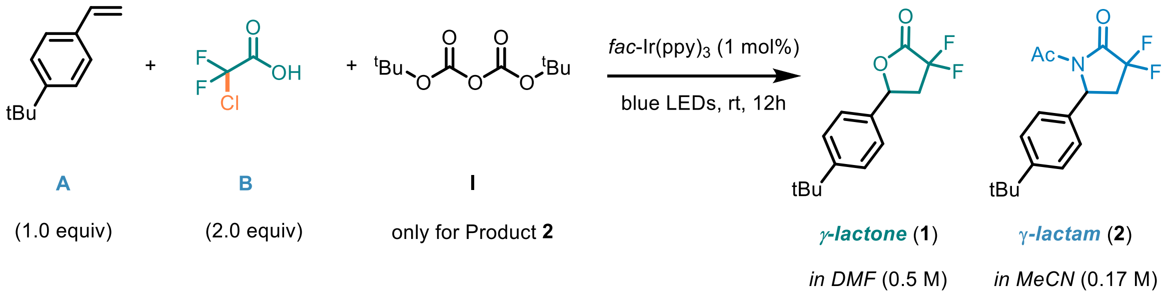

**Entry**[a]	**Deviation from standard conditions**	**Yield of 1** [%][b,c]	**Yield of 2** [%][b,d]
1	No deviation	94	58
2	No light	0	0
3	No PC	0	0
4	No light and no PC	0	0
5	Heating at 70 °C	0	0
6	H_2O 2 eq	traces	complex mixture
7	H_2O 20 eq	traces	complex mixture
8	Without degassing	86	12
9	I added initally	-	57
10	B and I premixed for 12 h	-	48

Table S11. a. Reaction conditions: 4-*tert*-butylstyrene (1.0 equiv), *acid* B (2.0 equiv), *fac*-Ir(ppy)$_3$ (1.0 mol%) and solvent. **b.** Yield of **1** and **2** are determined by GC against an internal standard of *n*-decane. **c.** DMF is used as solvent. **d.** MeCN is used as solvent.

2.9. Optimization of hydro *gem*-difluoro esterification (3)

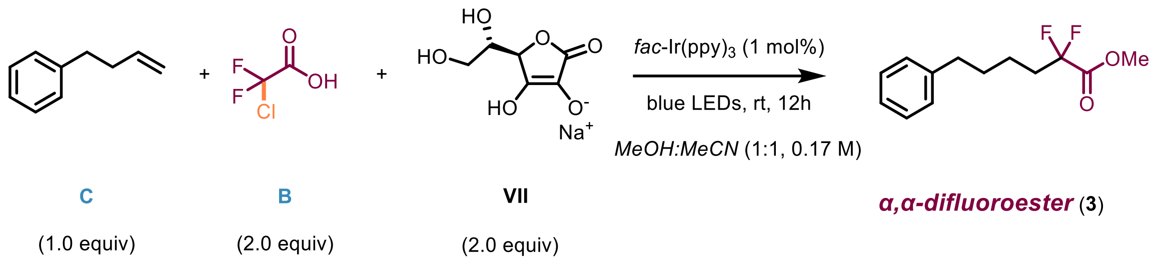

Entry[a]	Deviation from standard conditions	Yield of 2 [%][b,d]
1	No deviation	79
2	No light	0
3	No PC	0
4	No light and no PC	0
5	Heating at 70 °C	0
6	Without **VII**	complex mixture
7	Without MeOH	complex mixture
8	Without MeCN	65
9	Hantsch Ester insted of **VII**	43
10	Ascorbic acid insted of **VII**	51
11	Bromodifluoroacetic acid insted of **B**	75

Table S12. a. alkene **C** (1 equiv), *fac*-Ir(ppy)$_3$ (1.0 mol%), CDFA (2 equiv), **VII** (2 equiv), MeOH: MeCN (1:1 v/v), blue LEDs, rt, 12 h. **b.** Yield of **3** is determined by GC against an internal standard of *n*-decane.

3. General procedures

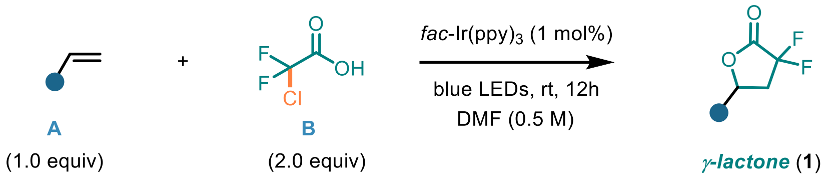

Procedure 1: A flame dried 8 mL crimp cap vial was charged with *fac*-[Ir(ppy)$_3$] (3.3 mg, 5 µmol, 1.0 mol%) and equipped with a magnetic bar. The contents of the vial were then subjected to three vacuum/argon cycles. Anhydrous DMF (1 mL) was added under an N_2 atmosphere, and the solution was degassed for 1 min. The substrate[a] (0.5 mmol, 1.0 equiv) and *CDFA (88* µL, 1.0 mmol, 2.0 equiv) were introduced to the solution *via* microsyringes. The reaction mixture was irradiated at room temperature under blue LEDs for 12 h. Reaction contents were added to an appropriately sized separatory funnel and 10 mL EtOAc was added and washed with water (3×5) mL; further organic layer was washed with 5 mL brine, dried over Na_2SO_4, filtered, and concentrated under reduced pressure. The residue was purified by flash column chromatography or manually over silica gel as indicated to afford γ-lactone derivatives.

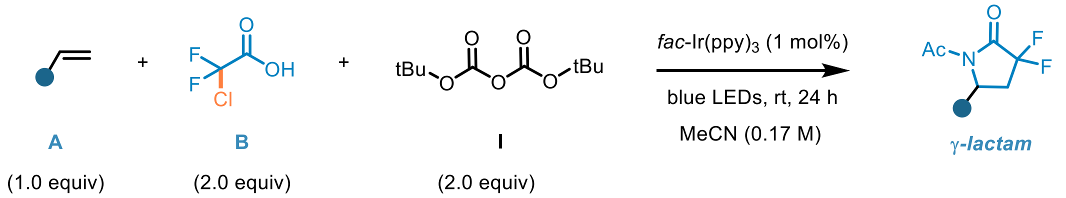

Procedure 2: A flame dried 8 mL crimp cap vial was charged with *fac*-[Ir(ppy)$_3$] (3.3 mg, 5.0 µmol, 1.0 mol%) and equipped with a magnetic bar. The contents of the vial were then subjected to three vacuum/argon cycles. Anhydrous DMF (3 mL) was added under an N_2 atmosphere, and the solution was sparged for 4 min. The substrate[a] (0.5 mmol, 1.0 equiv) and *CDFA* (88 µL, 1.0 mmol, 2.0 equiv) were introduced to the solution *via* microsyringes. The reaction mixture was irradiated at room temperature under blue LEDs for 24 h. The solvent was evaporated under reduced pressure, and the crude product was purified by flash column chromatography or manually over silica gel as indicated to get γ-lactams.

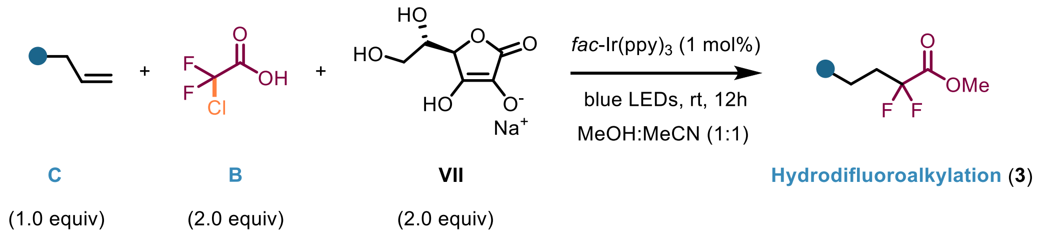

Procedure 3: A flame dried 8 mL crimp cap vial was charged with *fac*-[Ir(ppy)$_3$] (3.3 mg, 5.0 µmol, 1.0 mol%), sodium ascorbate (1.0 mmol, 2 equiv) and equipped with a magnetic bar. The contents of the vial were then subjected to three vacuum/argon cycles. Anhydrous MeOH (1.5 mL) and MeCN (1.5 mL) was added under an N_2 atmosphere, and the solution was sparged for 4 min. The substrate[a] (0.5 mmol, 1.0 equiv) and *CDFA* (88 µL, 1.0 mmol, 2.0 equiv) were introduced to the solution *via* microsyringes. The reaction mixture was irradiated at room temperature under blue LEDs for 12 h. The solvent was evaporated under reduced pressure, and the crude product was filtered on celite with DCM, the obtained solution was evaporated under reduced pressure and crude was purified by flash column chromatography or manually over silica gel as indicated to obtain hydro gem-difluoro esters.

[a] solid olefins were added after photocatalyst and then the crimp vials were sealed.

4. Availability of starting materials

Commercially available starting materials.

Commercially available starting materials were purchased from Thermoscientific – Acros, Sigma Aldrich, Apollo Scintific, Fluorochem and TCI or Chemie-brunschwig.

Olefins:

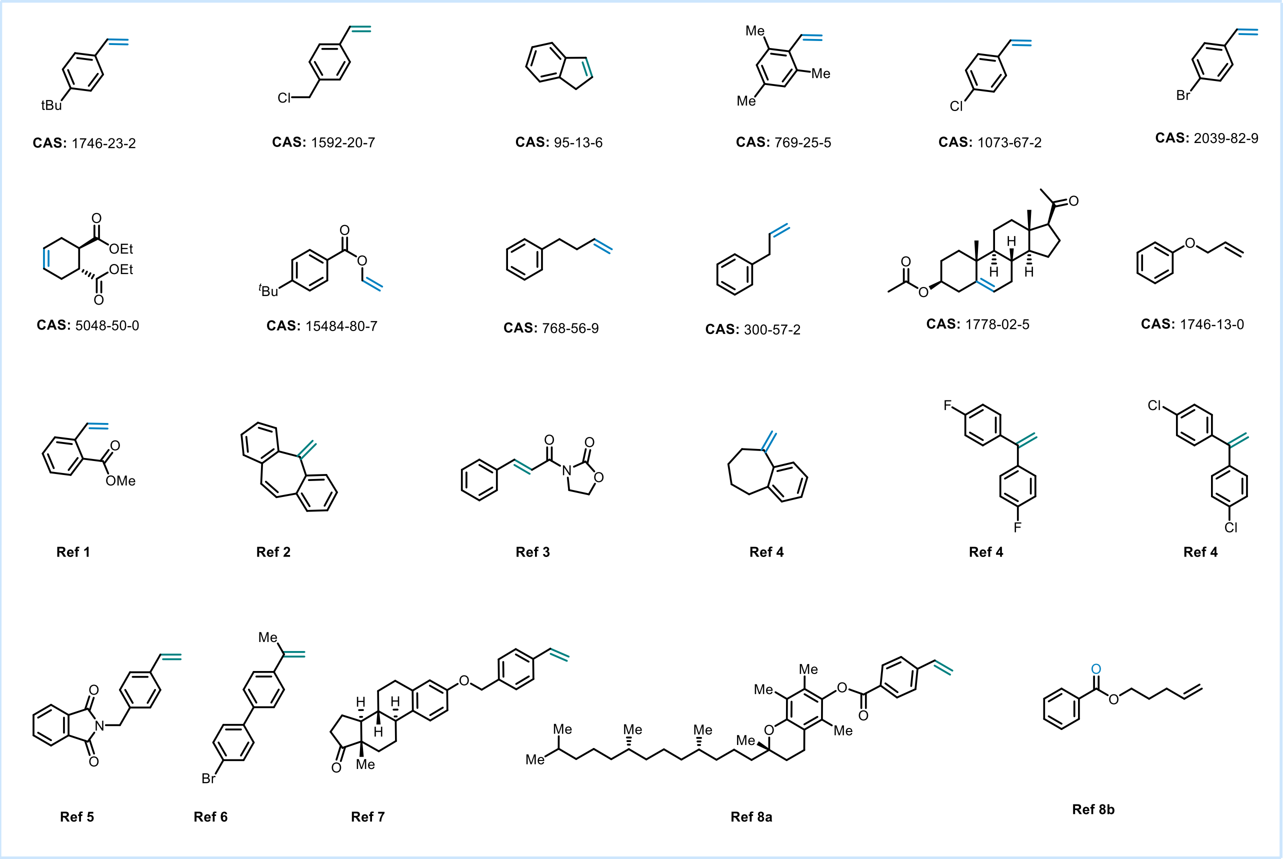

Figure S3. Source of olefins

Acids:

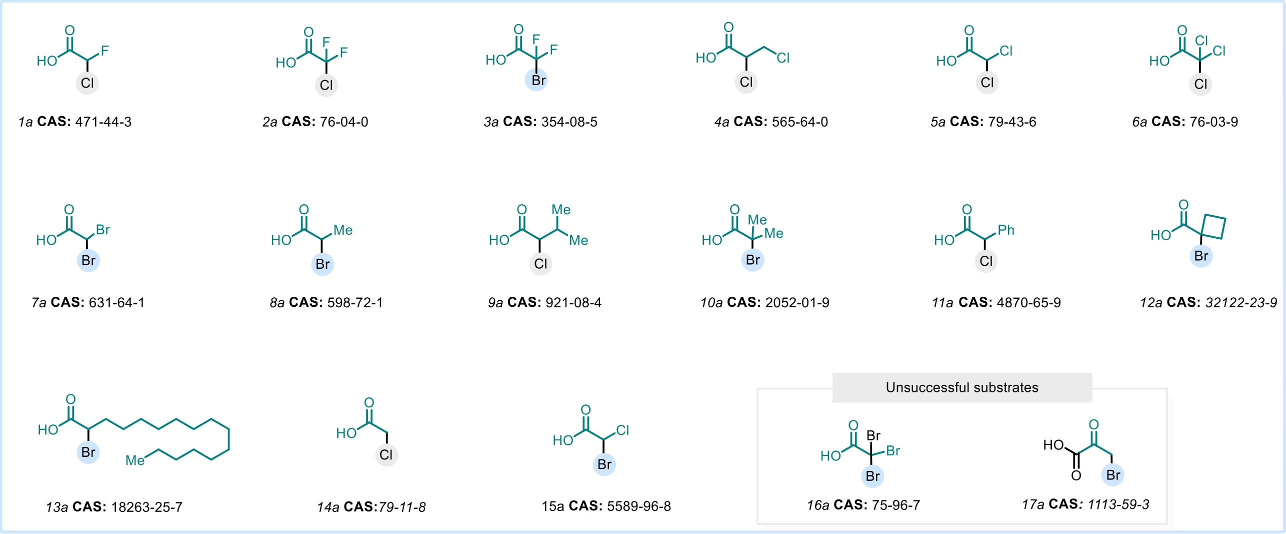

Figure S4. Source of acids

5. Cyclic voltammetry

Cyclic voltammetry data for halogenated acids

In a volumetric flask, 1.94 g (5.0 mmol, 0.1 M) of NBu_4PF_6 was dissolved in 50 mL of anhydrous acetonitrile. The solution was then degassed for 20 minutes by bubbling N_2. For each measurement, 10 mL of the solution was transferred to the cyclic voltammetric cell, followed by the addition of 0.1 mmol of analyte. The resulting solution was stirred for 1 minute to ensure homogeneity. Prior to each measurement, the solution was purged with Ar, and the glassy carbon electrode was rotated to homogenize the probe solution. Throughout the measurement process, the solution was shielded by a positive N_2 stream (Figure S5).

The cyclic voltammetry setup comprised a Pt-counter electrode and an Ag^+/Ag reference electrode (0.01 M $AgNO_3$ in 0.1 M NBu_4PF_6). The working electrode selected was a Pt disc electrode with a diameter of 2 mm. An overview of cyclic voltammetry was referenced from the provided sources. Typically, a scan rate of 100 mV/s was employed to measure the reaction components.

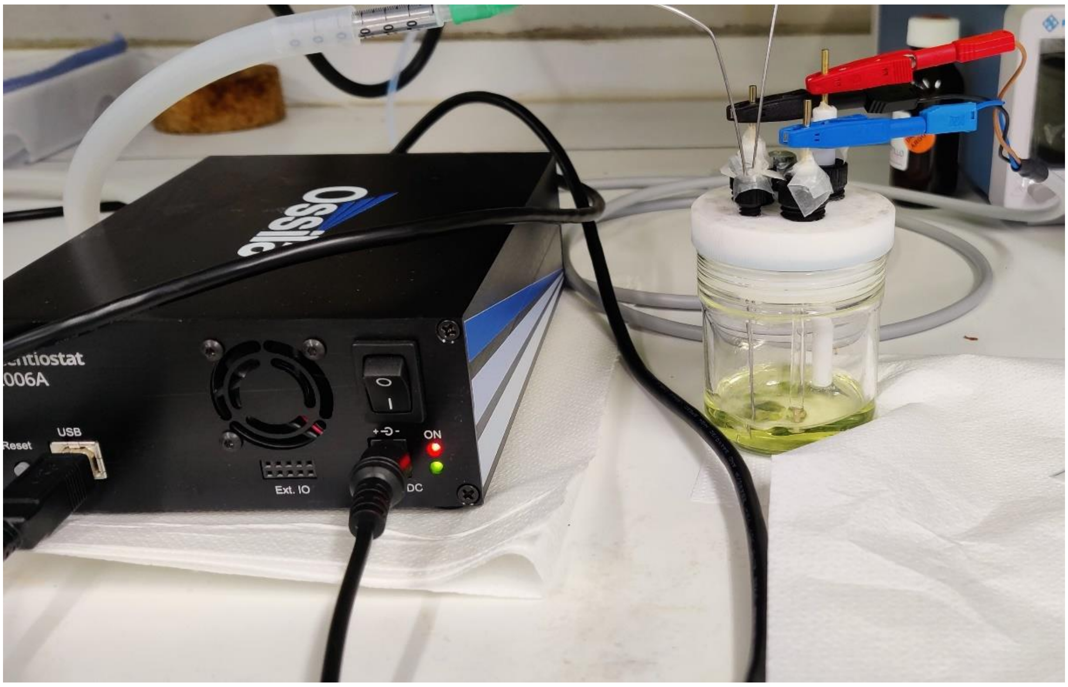

Figure S5. Cyclic voltammetry setup using ossila source measure unit.

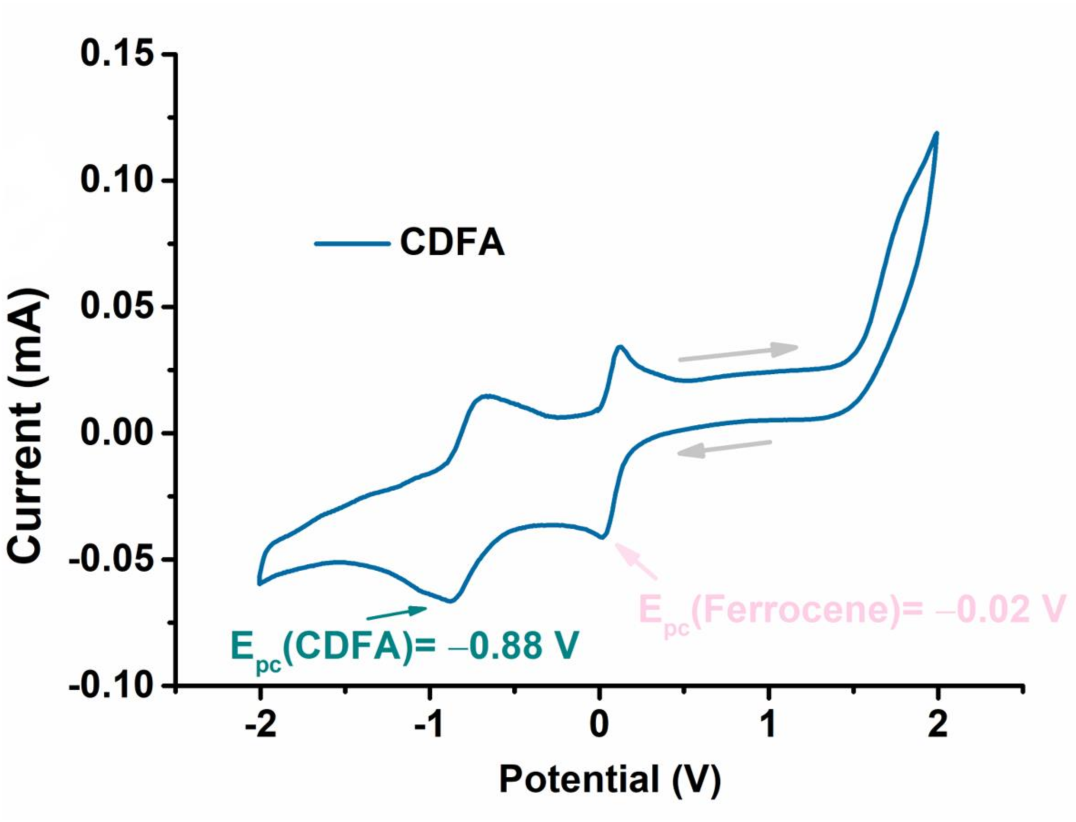

Figure S6. Cyclic voltammogram of chlorodifluoroacetic acid (CDFA, 0.1 mmol vs Ag/AgNO3), scan rate 100 mV/s.

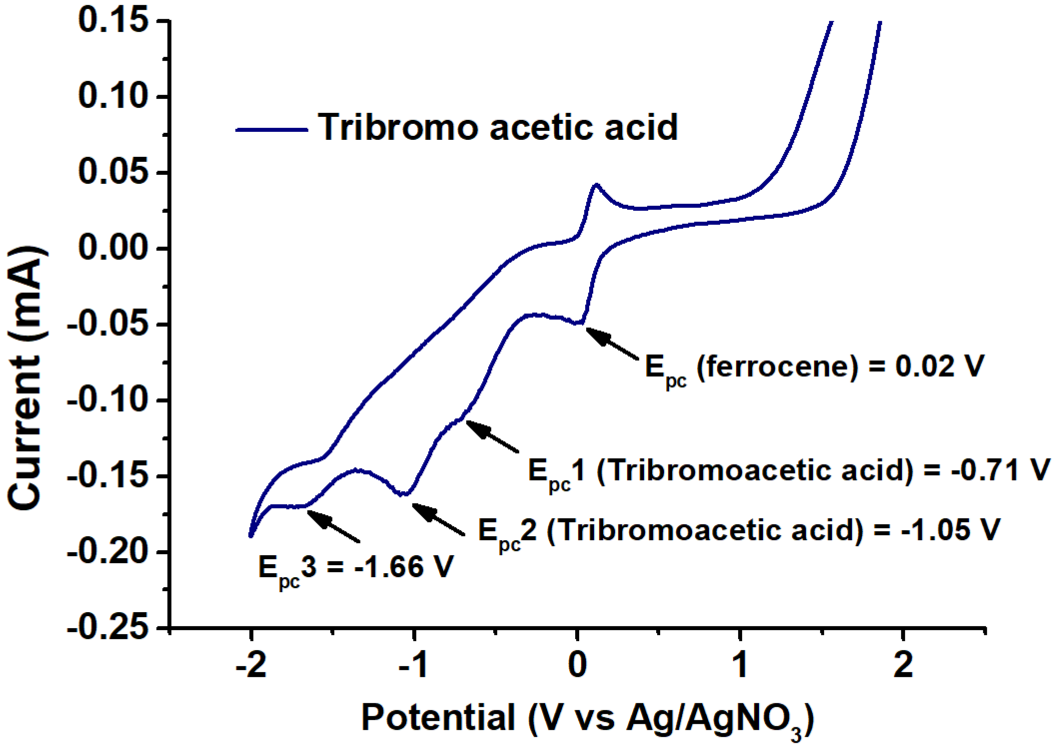

Figure S7. Cyclic voltammogram of tribromoacetic acid (0.1 mmol), scan rate 100 mV/s.

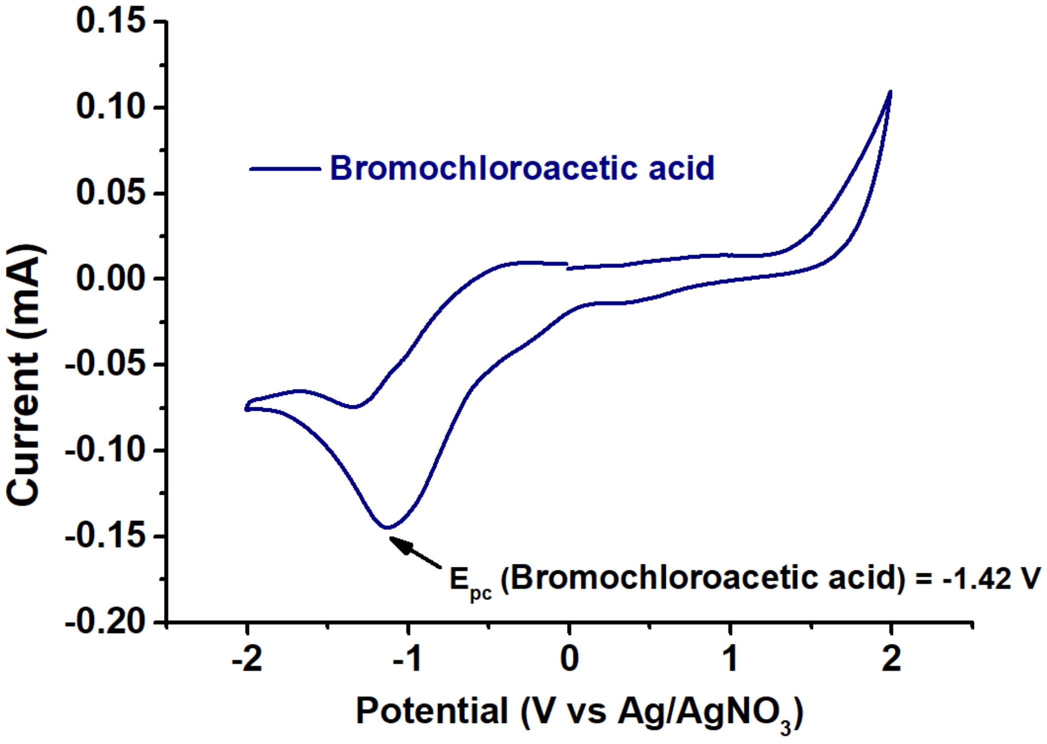

Figure S8. Cyclic voltammogram of bromochloroacetic acid (0.1 mmol), scan rate 100 mV/s.

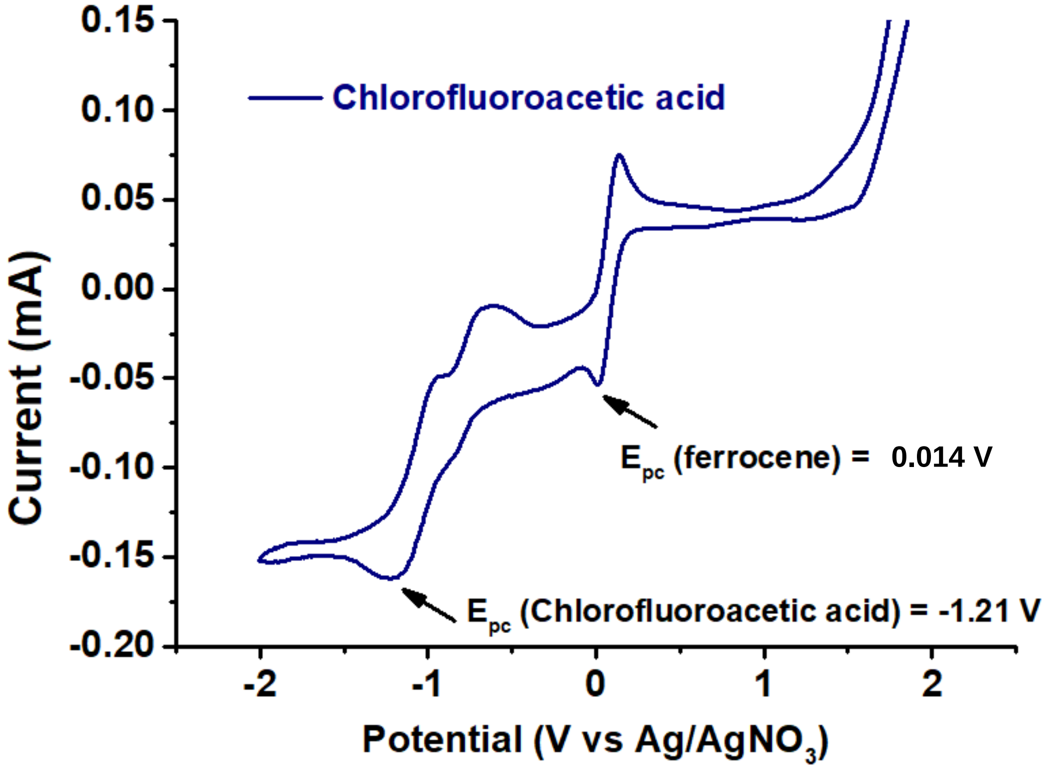

Figure S9. Cyclic voltammogram of chlorofluoroacetic acid (0.1 mmol), scan rate 100 mV/s.

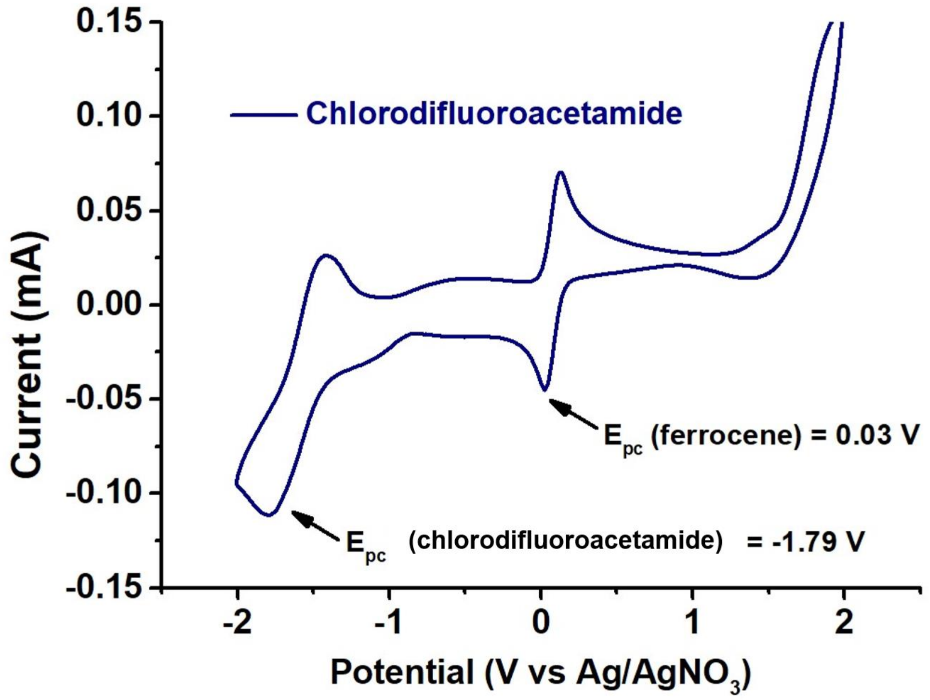

Figure S10. Cyclic voltammogram of chlorodifluoroacetamide (0.1 mmol), scan rate 100 mV/s.

6. Stern-Volmer quenching studies

To explore the dynamics of the excited state, we conducted Stern-Volmer quenching studies. The quenching efficiency, as defined by Stern and Volmer.

$(I_0 \div I) - 1 = k_q \tau_0$ [quencher]

Where I_0 is the luminescence intensity in the absence of any quencher, I is the luminescence intensity in the presence of a predefined quencher concentration while τ_0 is the excited state lifetime of photocatalyst which has been previously reported as 1.90 x 10^{-6} s for *fac*-Ir(ppy)$_3$ in acetonitrile at 25 °C).[9-11]

Preparation of stock solutions for Stern-Volmer measurements

A stock solution of the photocatalyst was prepared by dissolving 0.33 mg *fac*-Ir(ppy)$_3$ (0.499 µmol, 50 µM) in oxygen- and water-free 10 mL DMF under argon. A 2.5 mM stock solution was prepared for each component of the reaction in 10 mL of DMF, including chlorodifluoroacetic acid, 4-*tert*-butylstyrene, and Boc anhydride.

For evaluation of the quenching ability of chlorodifluoroacetic acid, 4-*tert*-butylstyrenerene and Boc anhydride, samples of *fac*-Ir(ppy)$_3$ and the reagent were prepared under argon in dark. Photocatalyst stock solution (1.5 mL) was added to quartz cuvettes (1.5 mL, 10 mm x 4 mm, PTFE cap), followed by the appropriate amount of reagent stock solution. All samples have been kept in the dark and were only taken out for measurements. Fluorescence Emission spectra were acquired as fast as possible after sample preparation (excitation at 430 ± 2 nm, 1 nm steps, excitation slit, and emission slit: 2 nm).

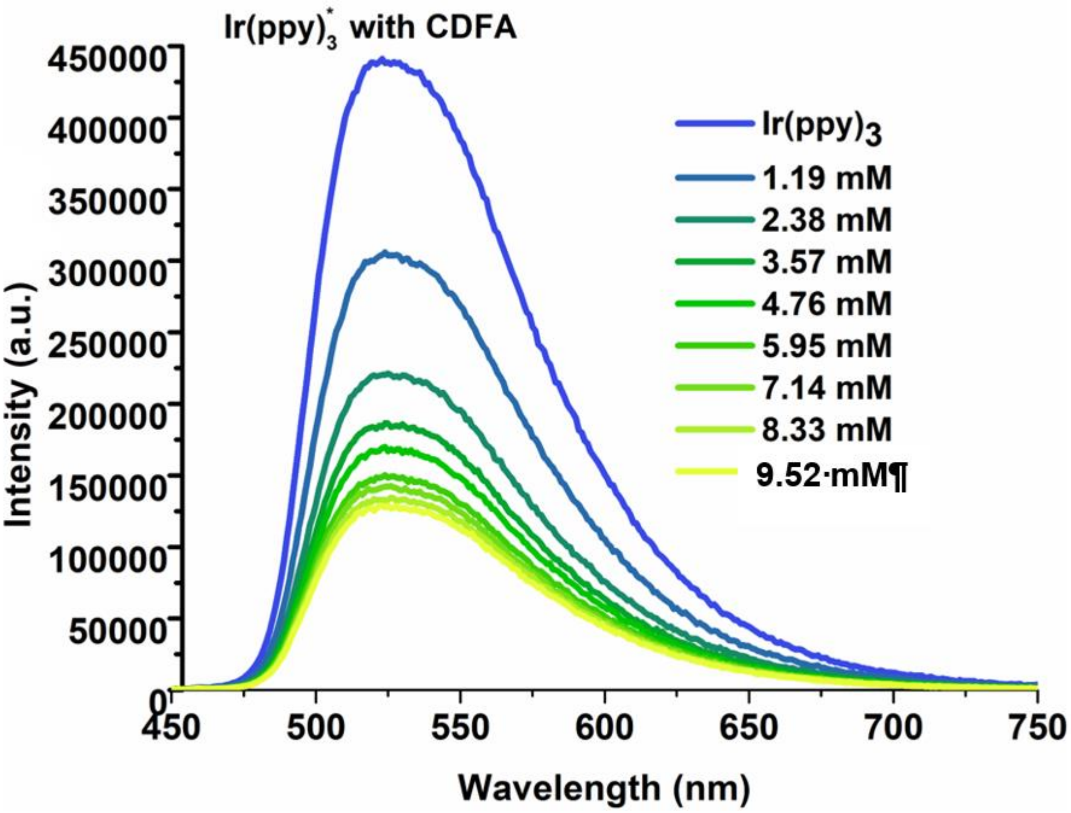

Figure S11. Emission quenching of *fac*-Ir(ppy)$_3$ with *CDFA* in DMF (50 µM *fac*-Ir(ppy)$_3$. In DMF) 20°C, d_{em} = 2 nm, d_{ex} = 2 nm, λ_{ex} = 430 nm, λ_{em} = 450-450 nm).

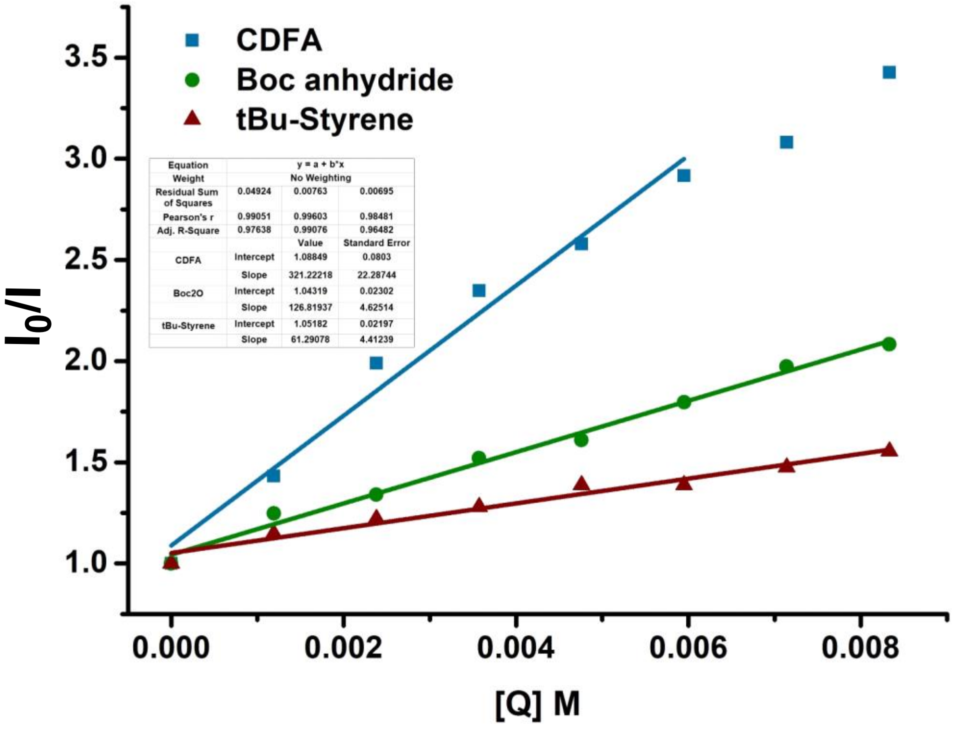

Figure S12. Correlation between emission intensity and concentration of reaction components.

First, the influence on the emission spectrum of the catalyst by *CDFA* was investigated. The emission quenching data clearly show that quenching of the excited state of the catalyst by *CDFA* is highly efficient and happening with a calculated quenching constant of $k_q = 2.0 \times 10^8$ $M^{-1}s^{-1}$ (Figures S11 and S12). Calculation of the quenching constants was performed for *CDFA* according to the following equation:

$(I_0 \div I) - 1 = k_q \tau_0$ [quencher]

Where $\tau_0 = 1.90 \times 10^{-6}$ s

Thus $k_q \tau_0 = 321$

$k_q = 1.7 \times 10^8$ M^{-1} s^{-1}

Later on, the quenching experiment was replicated independently with *t*Bu-styrene and Boc anhydride as quenchers. It was observed that in the presence of these substrates, the quenching of the catalyst's excited state was less pronounced, suggesting an inability to effectively compete with the efficient quenching caused by CDFA (Figure S13 and S14).

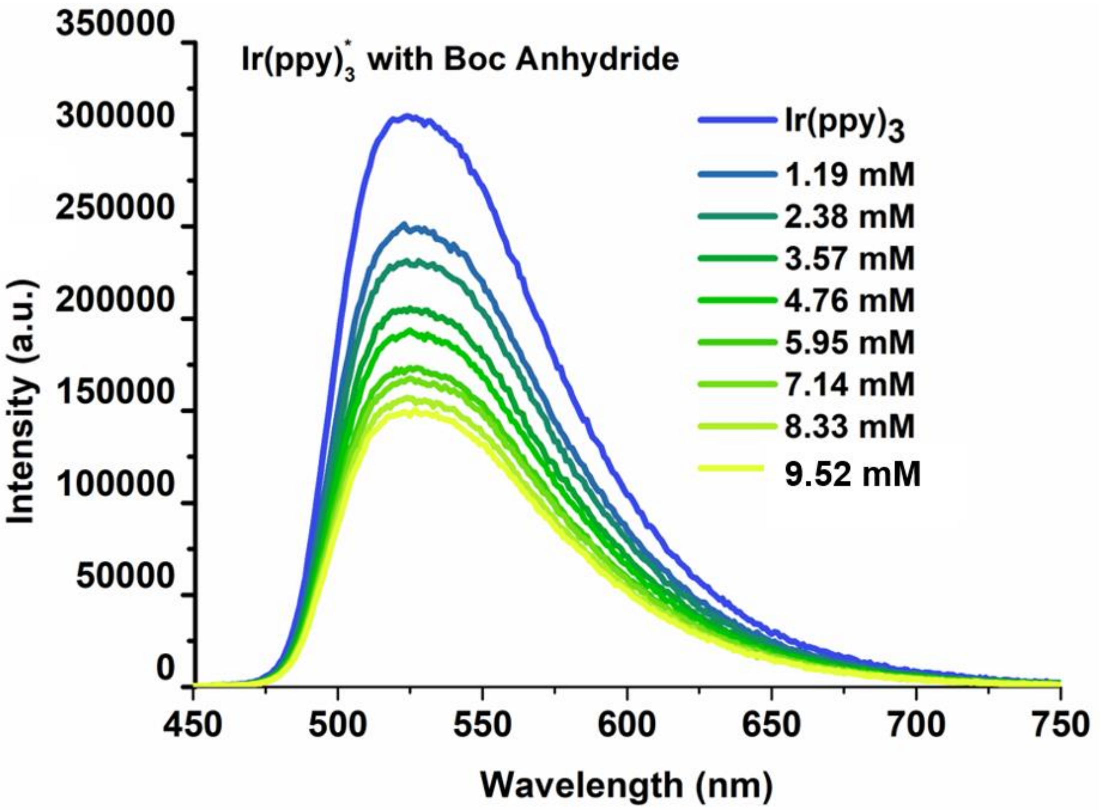

Figure S13. Emission quenching of *fac*-Ir(ppy)$_3$ with *Boc* anhydride in DMF (50 µM *fac*-Ir(ppy)$_3$. In DMF) 20°C, d_{em} = 2 nm, d_{ex} = 2 nm, λ_{ex} = 430 nm, λ_{em} = 450-750 nm).

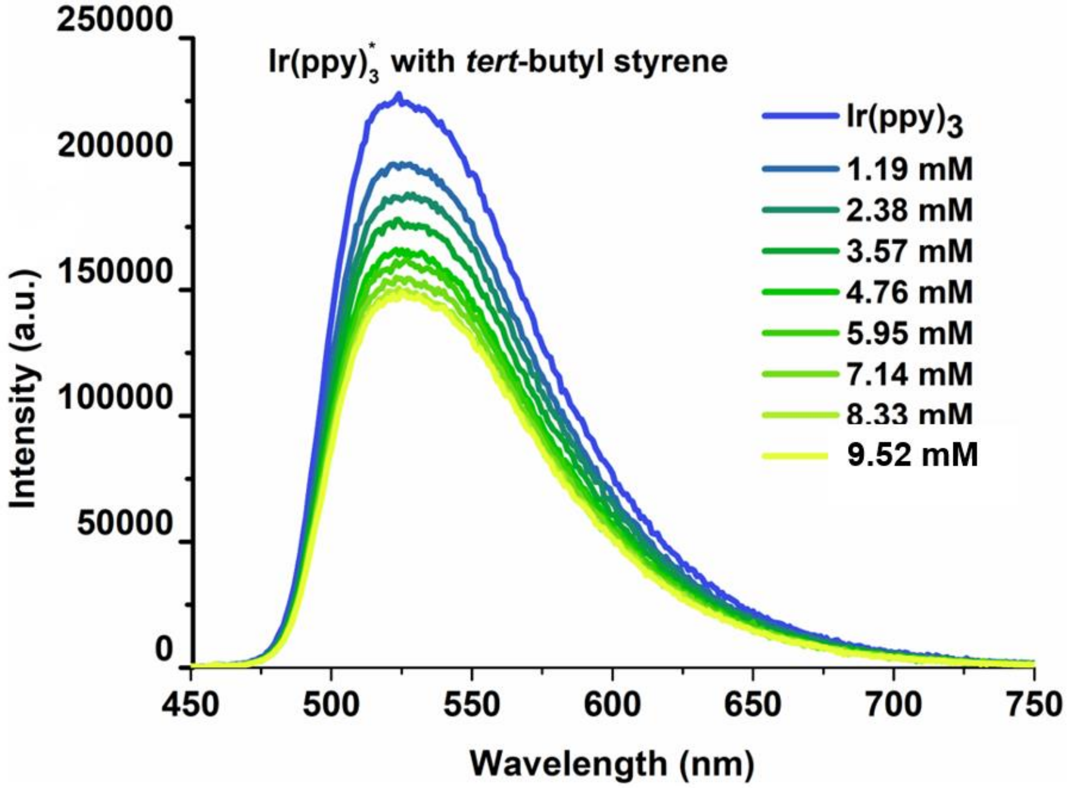

Figure S14. Emission quenching of *fac*-Ir(ppy)$_3$ with 4-*tert*-butylstyrene in DMF (50 µM *fac*-Ir(ppy)$_3$. In DMF) 20°C, d_{em} = 2 nm, d_{ex} = 2 nm, λ_{ex} = 430 nm, λ_{em} = 450-750 nm).

6. Radical trapping experiment

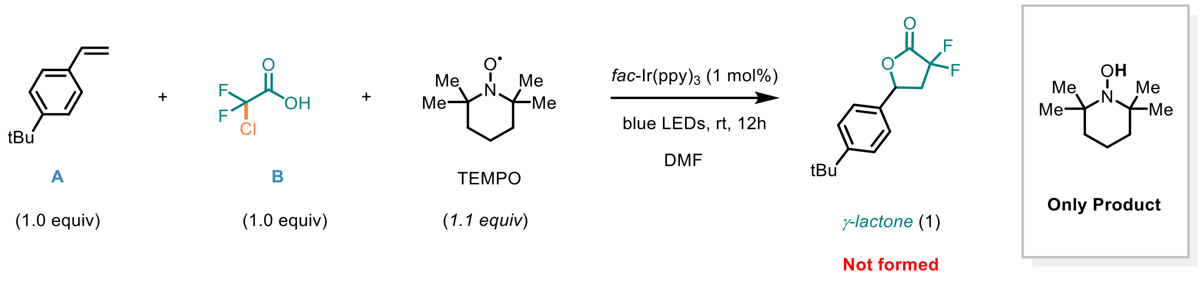

A flame dried 20 mL crimp cap vial was charged with *fac*-[Ir(ppy)$_3$] (3.3 mg, 5.0 μmol, 1.0 mol%) and equipped with a magnetic bar. The content of the vial was then subject to three vacuum/argon cycles. Anhydrous DMF (1 mL) was added under an argon atmosphere, and the solution was sparged for 5 min. 4-*tert*-butylstyrene (0.5 mmol, 1.0 equiv), 2,2,6,6-tetramethylpiperidin-1-yl)oxidanyl (TEMPO) (86 mg, 0.55 mmol, 1.1 equiv) and *chlorodifluoroacetic acid* (44 μL, 0.5 mmol, 1.0 equiv) were introduced to the solution *via* microsyringes. The reaction mixture was stirred at room temperature under blue LEDs irradiation for 12 h. Crude product was analysed by GC-MS and NMR, confirming no product formation and starting material remains unreacted.

7. Competition experiment

In this reaction, the use of acids either substituted at α-chloro or α-bromo demonstrated nearly identical efficiency. This assertion was confirmed *via* a competition experiment utilizing bromochloroacetic acid which led to a product mixture comprising both α-chloro lactone and α-bromo lactone. There was a slight preference for the former, indicating that C-Br cleavage in the acid occurs faster than C-Cl cleavage

A flame dried 20 mL crimp cap vial was charged with *fac*-[Ir(ppy)$_3$] (3.3 mg, 5.0 µmol, 1.0 mol%) and equipped with a magnetic bar. The content of the vial was then subject to three vacuum/argon cycles. Anhydrous DMF (1 mL) was added under an argon atmosphere, and the solution was sparged for 5 min. 4-*tert*-butylstyrene (0.5 mmol, 1.0 equiv), and *bromochloroacetic acid* (100 µL, 1.0 mmol, 2.0 equiv) were introduced to the solution *via* microsyringes. The reaction mixture was stirred at room temperature under blue LEDs irradiation for 12 h. Crude product was analysed by GC-MS and ratio of isomers were found to be 55:45.

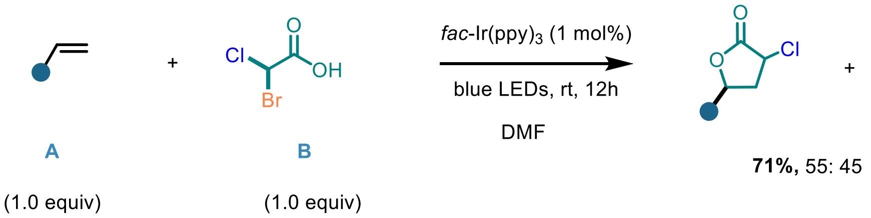

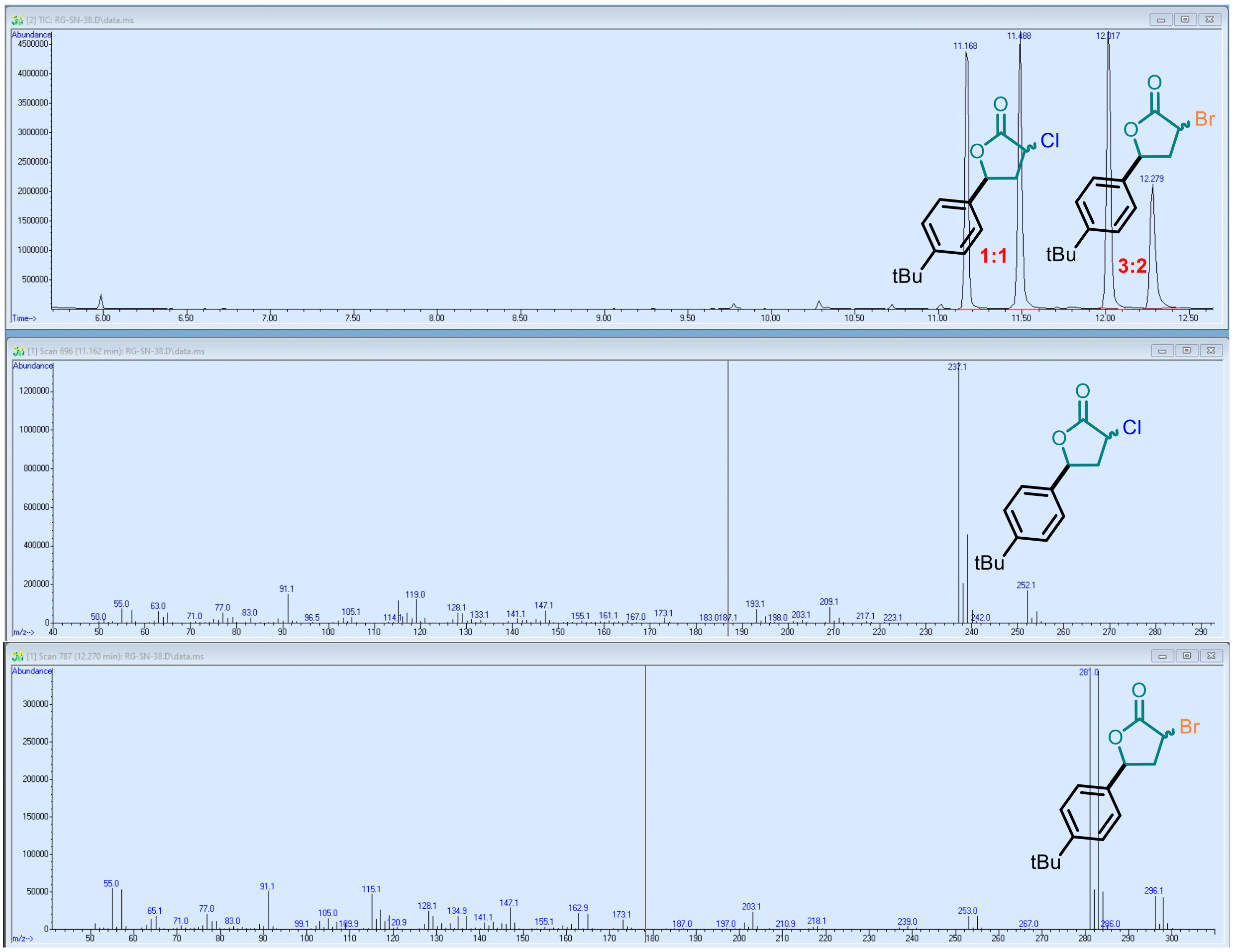

Figure S15. GC-MS for competition experiment.

8. Scale-up

Flow set-up

Procedure for reaction in flow: In an oven dried flask, 4-*tert*-butylstyrene(obtained commercially from Thermoscientific - Alfa Aesar, 3.65 mL, 20.0 mmol, 1.0 equiv) and *fac*-[Ir(ppy)$_3$] (131.0 mg, 0.2 mmol, 1.0 mol%) were dissolved in 20 mL of DMF. In another flask, chlorodifluoroacetic acid (40.0 mmol, 3.4 mL) was dissolved in 20 mL of DMF. Both solutions were drawn up into syringes and connected to a syringe pump. These syringes were then attached to a 12 mL flow coil tubing (PFA tubing, 1 mm inner diameter) *via* a PEEK Y-mixer. The stock solutions were pumped into the flow reactor at a flow rate of 0.1 mL/min from each syringe, corresponding to a residence time of 2 hours. When the syringe became empty, fresh stock solution was loaded into another syringe and injected into the reactor to take all the product at the end. Following this, the syringe filled with nitrogen was purged at the same flow rate to remove any remaining reaction mixture from the tubes. The reaction mixture was added to an appropriately sized separatory funnel and 200 mL EtOAc was added and washed with water (4×80) mL; further organic layer was washed with 60 mL brine. The combined organic layers were then dried over Na_2SO_4, filtered, and concentrated under reduced pressure. The residue was purified by flash column chromatography over silica gel as indicated to afford γ–lactone as white solid (3.8 g, 75% yield).

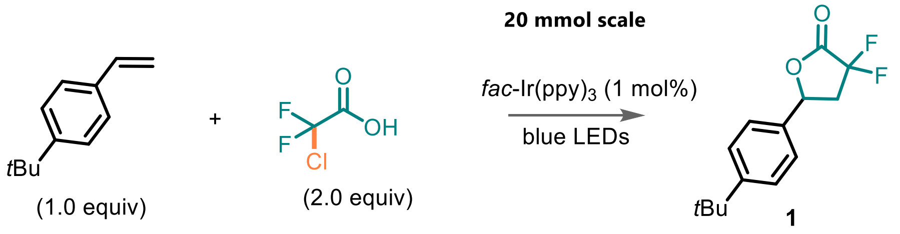

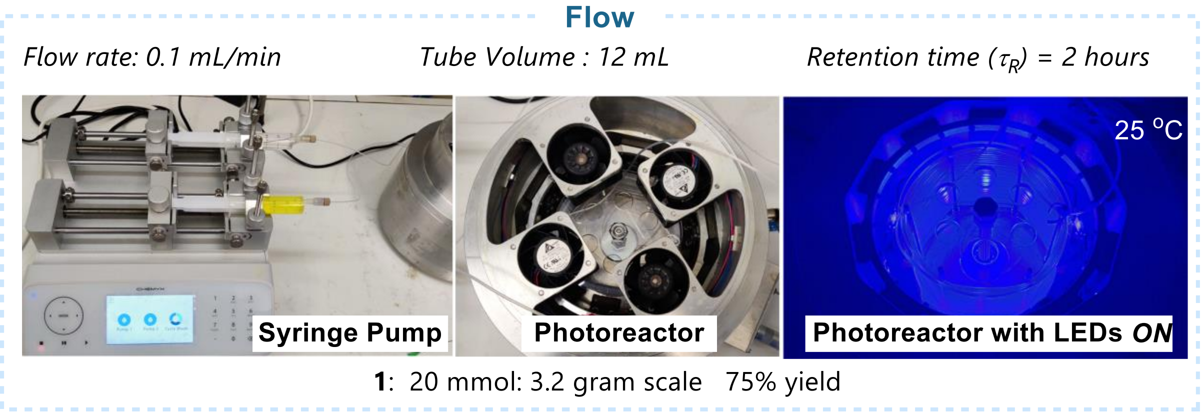

Figure S16. Flow setup for scale-up lactone in DMF.

Tube volume

$V = \pi r^2 h$ (r= 0.5 mm; h = 15240 mm)
$V = 3.14 \times (0.5)^2 \times (15240)\ mm^3$
$V = 11969.46\ mm^3$
$V = 11.97$ mL

Retention time

τ_R = Tube volume / Flow rate
$\tau_R = 11.97$ mL / 0.1 $mLmin^{-1}$
$\tau_R = 119.7$ min
$\tau_R = 2$ h

Batch set-up

Procedure for reaction in Batch: A flame-dried 150 mL Schlenk tube was charged with *fac*-[Ir(ppy)$_3$] (65.0 mg, 0.1 mmol, 1.0 mol%) and equipped with a magnetic stir bar. The contents of the tube were subjected to three vacuum/nitrogen cycles. Anhydrous DMF (30 mL) was added under an argon atmosphere, and the solution was purged for 5 minutes. 4-*tert*-butylstyrene (1.8 mL, 10.0 mmol, 1.0 equiv) and chlorodifluoroacetic acid (1.7 mL, 20.0 mmol, 2.0 equiv) were then added to the solution. The reaction mixture was stirred at room temperature under blue LED irradiation for 24 hours. The reaction contents were transferred to an appropriately sized separatory funnel, and 100 mL of EtOAc was added. The mixture was washed with water (3 × 50 mL), and the organic layer was further washed with 30 mL of brine. The combined organic layers were dried over Na_2SO_4, filtered, and concentrated under reduced pressure. The residue was purified by flash column chromatography over silica gel, yielding γ-lactone as a yellow solid (2.13 g, 84% yield).

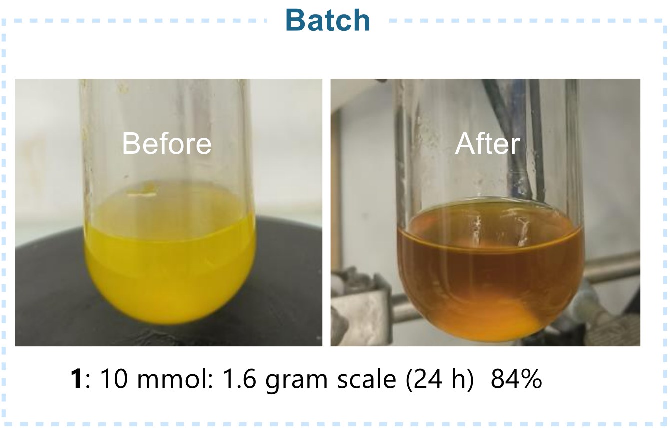

Figure S17. Batch setup for scale-up lactone in DMF.

9. Proposed mechanism for product 3

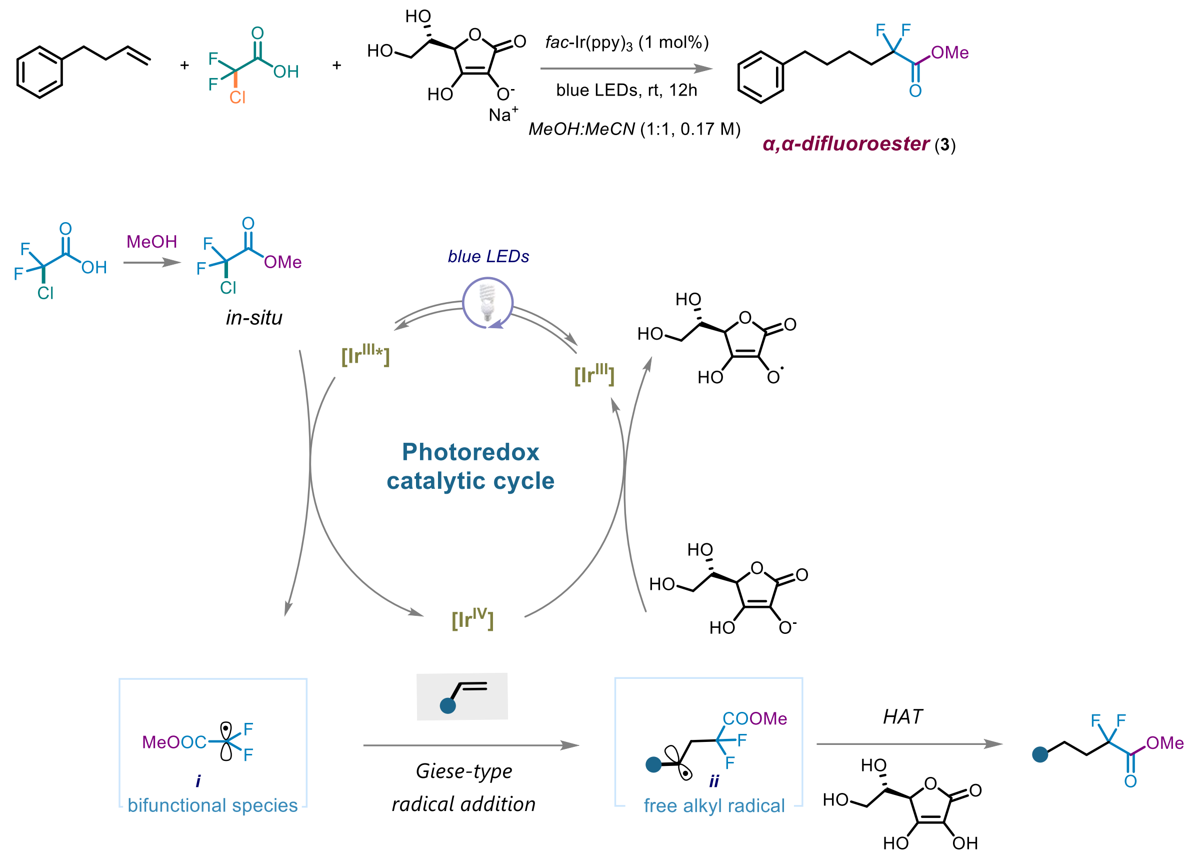

Figure S18. Proposed mechanism for hydro esterification reaction.

10. Computational parameters

The global electrophilicity index **(*ω* eV)** values depicted in the manuscript are taken from the review by our group. The CF_2COOH radical was not computed in the review and its parameters were determined using the same level of theory as the other compounds. See supporting information (*Angew. Chem. Int. Ed.* **2024**, e202318377).[12] **section 2.2** of the review for details.

	**Parameters in gas phase (U)B3LYP-D3(BJ)/6-311+G(d,p)**					
**Radical**	**IP (eV)**	**EA (eV)**	***η* (eV)**	***μ* (eV)**	***ω* (eV)**	***ω*⁻ (eV)**
$^{\bullet}CF_2H$	10.34	-0.26	10.60	-5.04	1.196	0.268
$^{\bullet}CF_2CO_2Me$	9.40	0.97	8.43	-5.18	1.594	0.242
$^{\bullet}CF_3$	11.41	0.61	10.80	-6.01	1.673	0.183
$^{\bullet}CF_2CO_2H$	9.75	1.12	8.63	-5.43	1.709	0.222

Table S13 **IP**: Ionization Potential; **EA**: Electron Affinity; **η**: Chemical hardness; **μ**: Chemical potential; **ω**: Global Electrophilicity index ω^-: Nucleophilicity index

NMR Data

5-(4-(Chloromethyl)phenyl)dihydrofuran-2(3*H*)-one (4)

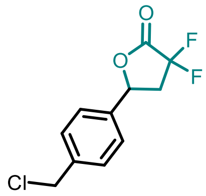

This compound was obtained according to general procedure **1** from 1-(chloromethyl)-4-vinylbenzene (77.0 mg, 0.5 mmol), *fac*-[Ir(ppy)$_3$] (3.3 mg, 1.0 mol%), *CDFA (88* µL, 1.0 mmol), and anhydrous DMF (1 mL). Isolated as a white solid (96.0 mg, 78% yield) after purification by flash column chromatography (12 g SiO_2, hexane/EA = 25:1). Spectroscopic data match with those reported in the literature.[13]

^{1}H-NMR (500 MHz, $CDCl_3$): δ 7.50 – 7.42 (m, 2H), 7.37 – 7.31 (m, 2H), 5.62 (dd, *J* = 8.8, 6.3 Hz, 1H), 4.60 (s, 2H), 3.20 – 3.08 (m, 1H), 2.71 – 2.55 (m, 1H).

^{13}C-NMR (126 MHz, $CDCl_3$): δ 164.8 (dd, *J* = 33.9, 32.0 Hz),139.2, 136.6, 129.5, 126.2, 115.6 (dd, *J* = 258.9, 250.2 Hz), 76.7 (dd, *J* = 7.1, 1.9 Hz), 45.5, 40.0 (dd, *J* = 22.3, 20.8 Hz)

^{19}F-NMR (282 MHz, $CDCl_3$): δ -106.5 (d, *J* = 279.2 Hz), -109.1 (d, *J* = 279.2 Hz).

3,3-Difluoro-5,5-bis(4-fluorophenyl)dihydrofuran-2(3*H*)-one (5)

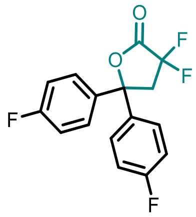

This compound was obtained according to general procedure **1** 4,4'-(ethene-1,1-diyl)bis(fluorobenzene) (108.1 mg, 0.5 mmol), *fac*-[Ir(ppy)$_3$] (3.3 mg, 1.0 mol%), *CDFA (88 µL*, 1.0 mmol), and anhydrous DMF (1 mL). Isolated as a white crystalline solid (103.9 mg, 67% yield) after purification by flash column chromatography (12 g SiO_2, hexane/EA = 20:1). Spectroscopic data match with those reported in the literature.[13]

^{1}H-NMR (300 MHz, $CDCl_3$): δ 7.38 – 7.24 (m, 4H), 7.15 – 7.01 (m, 4H), 3.39 (t, *J* = 13.7 Hz, 2H).

^{13}C-NMR (75 MHz, $CDCl_3$): δ 164.4 (t, *J* = 16.2 Hz), 161.1, 137.4 (d, *J* = 3.4 Hz), 127.4 (d, *J* = 8.6 Hz), 116.2 (d, *J* = 21.9 Hz), 115.5 (t, *J* = 254.9 Hz), 85.9 (t, *J* = 4.0 Hz), 44.8 (t, *J* = 20.8 Hz).

^{19}F-NMR (282 MHz, $CDCl_3$): δ -112.4, -104.6.

5,5-Bis(4-chlorophenyl)-3,3-difluorodihydrofuran-2(3*H*)-one (6)

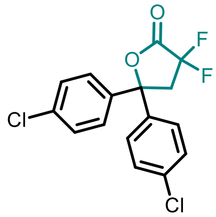

This compound was obtained according to general procedure **1** 4,4'-(ethene-1,1-diyl)bis(chlorobenzene) (124.6 mg, 0.5 mmol), *fac*-[Ir(ppy)$_3$] (3.3 mg, 1.0 mol%), *CDFA (88* µL, 1.0 mmol), and anhydrous DMF (1 mL). Isolated as a white crystalline solid (118.3 mg, 69% yield) after purification by flash column chromatography (12 g SiO_2, hexane/EA = 20:1). Spectroscopic data match with those reported in the literature.[13]

^{1}H-NMR (300 MHz, $CDCl_3$): δ 7.39 – 7.33 (m, 4H), 7.30 – 7.24 (m, 4H), 3.37 (t, *J* = 13.6 Hz, 2H).

^{13}C-NMR (75 MHz, $CDCl_3$): δ 164.0 (t, *J* = 32.9 Hz), 139.8, 135.2, 129.4, 126.8, 115.3 (t, *J* = 254.9 Hz), 85.6 (t, *J* = 4.6 Hz), 44.5 (t, *J* = 21.0 Hz).

^{19}F-NMR (282 MHz, $CDCl_3$): δ -104.7.

5-(4'-Bromo-[1,1'-biphenyl]-4-yl)-3,3-difluoro-5-methyldihydrofuran-2(3*H*)-one (7)

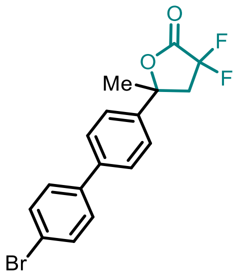

This compound was obtained according to general procedure **1** 4-bromo-4'-(prop-1-en-2-yl)-1,1'-biphenyl (136.1 mg, 0.5 mmol), *fac*-[Ir(ppy)$_3$] (3.3 mg, 1.0 mol%), *CDFA (88* µL, 1.0 mmol), and anhydrous DMF (1 mL). Isolated as a white crystalline solid (153.7 mg, 84% yield) after purification by flash column chromatography (12 g SiO_2, hexane/EA = 20:1). Spectroscopic data match with those reported in the literature.[13]

^{1}H-NMR (300 MHz, $CDCl_3$): δ 7.64 – 7.54 (m, 4H), 7.52 – 7.36 (m, 4H), 3.07 – 2.91 (m, 2H), 1.86 (s, 3H).

^{13}C-NMR (126 MHz, $CDCl_3$): δ 164.7 (t, *J* = 33.0 Hz), 142.0, 140.3, 139.1, 132.2, 128.8, 127.6, 124.6, 122.2, 116.0 (dd, *J* = 255.7, 253.0 Hz), 83.9 (dd, *J* = 5.1, 3.5 Hz), 45.1 (t, *J* = 20.6 Hz), 30.3.

^{19}F-NMR (282 MHz, $CDCl_3$): δ -101.8 (d, *J* = 282.6 Hz), -104.8 (d, *J* = 280.9 Hz).

2-(4-(4,4-Difluoro-5-oxotetrahydrofuran-2-yl)benzyl)isoindoline-1,3-dione (8)

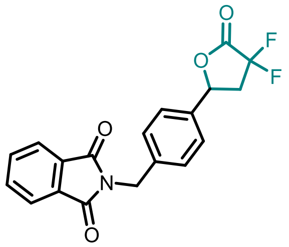

This compound was obtained according to general procedure **1** from 2-(4-vinylbenzyl)isoindoline-1,3-dione (132.0 mg, 0.5 mmol), *fac*-[Ir(ppy)$_3$] (3.3 mg, 1.0 mol%), *CDFA (88* µL, 1.0 mmol), and anhydrous DMF (1 mL). Isolated as a white crystalline solid (114.2 mg, 64% yield) after purification by flash column chromatography (12 g SiO_2, hexane/EA = 20:1). Spectroscopic data match with those reported in the literature.[13]

^{1}**H-NMR** (500 MHz, $CDCl_3$): δ 7.85 (dd, *J* = 5.5, 3.0 Hz, 2H), 7.72 (dd, *J* = 5.5, 3.0 Hz, 2H), 7.50 (d, *J* = 8.2 Hz, 2H), 7.29 (d, *J* = 8.2 Hz, 2H), 5.58 (dd, *J* = 8.8, 6.2 Hz, 1H), 4.86 (s, 2H), 3.15 – 3.03 (m, 1H), 2.67 – 2.51 (m, 1H).

^{13}**C-NMR** (126 MHz, $CDCl_3$): δ 168.1, 164.9 (dd, *J* = 33.8, 31.9 Hz), 138.1, 136.0, 134.3, 132.1, 129.5, 126.2, 123.6, 115.7 (dd, *J* = 259.1, 250.0 Hz), 76.8 (dd, *J* = 7.2, 1.9 Hz), 41.3, 40.0 (dd, *J* = 22.2, 20.7 Hz).

^{19}**F-NMR** (282 MHz, $CDCl_3$): δ -107.0 (d, *J* = 279.2 Hz), -109.2 (d, *J* = 279.2 Hz).

3-(4,4-Difluoro-5-oxo-2-phenyltetrahydrofuran-3-carbonyl)oxazolidin-2-one (9)

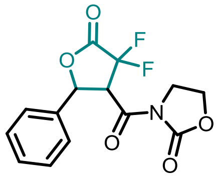

This compound was obtained according to general procedure **1** from 3-(3-phenylacryloyl)oxazolidin-2-one (108.6 mg, 0.5 mmol), *fac*-[Ir(ppy)$_3$] (3.3 mg, 1.0 mol%), *CDFA (88* µL, 1.0 mmol), and anhydrous DMF (1 mL). Isolated as a white crystalline solid (80.0 mg, 52% yield) after purification by flash column chromatography (12 g SiO_2, hexane/EA = 20:1). Crude NMR indicated the d.r. ratio 6:1. Spectroscopic data match with those reported in the literature.[13]

^{1}**H-NMR** (300 MHz, $CDCl_3$): δ 7.57 – 7.30 (m, 5H), 6.07 (d, *J* = 7.8 Hz, 1H), 5.47 – 5.27 (m, 1H), 4.57 – 4.36 (m, 2H), 4.20 – 3.96 (m, 2H).

^{13}**C-NMR** (101 MHz, $CDCl_3$): δ 163.1 (dd, *J* = 2.7, 1.6 Hz), 162.9 (dd, *J* = 32.7, 31.2 Hz), 152.9, 134.8, 130.2, 129.4, 126.5, 114.2 (dd, *J* = 266.8, 257.3 Hz), 78.8 (dd, *J* = 1.3, 1.2 Hz), 62.5, 53.0 (dd, *J* = 20.2, 18.3 Hz), 42.9.

^{19}**F-NMR** (282 MHz, $CDCl_3$): δ -107.4 (d, *J* = 274.0 Hz), -111.6 (d, *J* = 274.0 Hz).

3,3-Difluoro-3,3a,4,8b-tetrahydro-2*H*-indeno[1,2-*b*]furan-2-one (10)

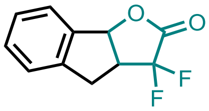

This compound was obtained according to general procedure **1** from indene (59.0 µL, 0.5 mmol), *fac*-[Ir(ppy)$_3$] (3.3 mg, 1.0 mol%), *CDFA (88* µL, 1.0 mmol), and anhydrous DMF (1 mL). Isolated as a white crystalline solid (70.0 mg, 67% yield) after purification by flash column chromatography (12 g SiO_2, hexane/EA = 20:1). Crude NMR indicated the d.r. ratio 5:1. Spectroscopic data match with those reported in the literature.[14]

^{1}**H-NMR** (300 MHz, $CDCl_3$): δ 7.58 – 7.49 (m, 1H), 7.47 – 7.39 (m, 1H), 7.38 – 7.29 (m, 2H), 5.99 (d, *J* = 6.7 Hz, 1H), 3.57 (ddtd, *J* = 19.8, 8.2, 7.0, 3.3 Hz, 1H), 3.30 (d, *J* = 7.6 Hz, 2H).

^{13}**C-NMR** (75 MHz, $CDCl_3$): δ 165.0 (dd, *J* = 31.4, 29.8 Hz), 142.8 (d, *J* = 1.2 Hz), 136.7 (d, *J* = 1.1 Hz), 131.3, 128.2, 126.5, 125.3, 116.4 (dd, *J* = 249.7, 249.6 Hz), 84.8 (dd, *J* = 3.5, 3.4 Hz), 44.7 (dd, *J* = 19.9, 19.7 Hz), 30.2 (dd, *J* = 5.1, 4.8 Hz).

^{19}**F-NMR** (282 MHz, $CDCl_3$): δ -101.1 (d, *J* = 282.6 Hz), -114.6 (d, *J* = 280.9 Hz).

HRMS (EI) *m/z*, $[M]^+$ calcd for $C_{11}H_8F_2O_2^+$: 210.0492; found: 210.0489.

4',4'-Difluoro-3',4'-dihydro-5'H-spiro[dibenzo[*a,d*][7]annulene-5,2'-furan]-5'-one (11)

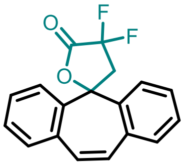

This compound was obtained according to general procedure **1** 5-methylene-5*H*-dibenzo[*a*,*d*][7]annulene (104.0 mg, 0.5 mmol), *fac*-[Ir(ppy)$_3$] (3.3 mg, 1.0 mol%), *CDFA (88* μL, 1.0 mmol), and anhydrous DMF (1 mL). Isolated as a white crystalline solid (124.0 mg, 62% yield) after purification by flash column chromatography (12 g SiO_2, hexane/EA = 20:1). Spectroscopic data match with those reported in the literature.[13]

^{1}H-NMR (300 MHz, $CDCl_3$): δ 7.77 – 7.67 (m, 2H), 7.51 – 7.29 (m, 6H), 7.12 (s, 2H), 2.97 (t, *J* = 14.8 Hz, 2H).

^{13}C-NMR (75 MHz, $CDCl_3$): δ 164.6 (t, *J* = 33.9 Hz), 138.6, 131.8, 131.4, 129.7, 129.4, 128.0, 122.3, 115.3 (t, *J* = 253.6 Hz), 84.2 (t, *J* = 4.7 Hz), 42.6 (t, *J* = 20.7 Hz).

^{19}F-NMR (282 MHz, $CDCl_3$): δ -104.3.

3,3-Difluoro-5-(4-((((8*R*,9*S*,13*S*,14*S*)-13-methyl-17-oxo-7,8,9,11,12,13,14,15,16,17-decahydro-6H-cyclopenta[*a*]phenanthren-3-yl)oxy)methyl)phenyl)dihydrofuran-2(3*H*)-one (12)

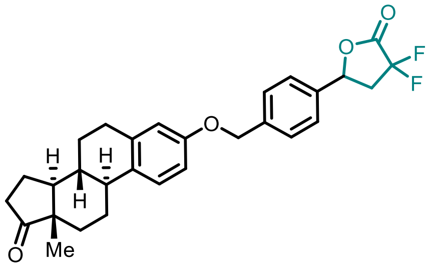

This compound was obtained according to general procedure **1** from (8*R*,9*S*,13*S*,14*S*)-13-methyl-3-((4-vinylbenzyl)oxy)-6,7,8,9,11,12,13,14,15,16-decahydro-17*H*-cyclopenta[*a*]phenanthren-17-one (193.1 mg, 0.5 mmol), *fac*-[Ir(ppy)$_3$] (3.3 mg, 1.0 mol%), *CDFA (88* μL, 1.0 mmol), and anhydrous DMF (1 mL). Isolated as a white crystalline solid (117.7 mg, 49% yield) after purification by flash column chromatography (12 g SiO_2, hexane/EA = 5:1). Spectroscopic data match with those reported in the literature.[13]

^{1}H-NMR (300 MHz, $CDCl_3$): δ 7.55 – 7.47 (m, 2H), 7.40 – 7.31 (m, 2H), 7.21 (d, *J* = 8.7 Hz, 1H), 6.82 – 6.68 (m, 2H), 5.63 (dd, *J* = 8.8, 6.1 Hz, 1H), 5.06 (s, 2H), 3.24 – 3.04 (m, 1H), 2.90 (q, *J* = 5.1 Hz, 2H), 2.78 – 2.30 (m, 4H), 2.24 – 1.88 (m, 4H), 1.70 – 1.38 (m, 6H), 0.91 (s, 3H).

^{13}C-NMR (101 MHz, $CDCl_3$): δ 221.0, 164.9 (dd, *J* = 33.7, 32.0 Hz), 156.7, 139.2, 138.1, 135.9, 132.8, 128.2, 126.6, 126.1, 115.7 (dd, *J* = 258.9, 250.2 Hz), 115.0, 112.5, 76.93 (dd, *J* = 7.3, 5.4 Hz), 69.4, 50.6, 48.1, 44.1, 40.1 (dd, *J* = 22.2, 20.8 Hz), 38.5, 36.0, 31.7, 29.8, 26.7, 26.0, 21.7, 14.0.

^{19}F-NMR (282 MHz, $CDCl_3$): δ -106.5 (d, *J* = 279.2 Hz), -109.1 (d, *J* = 279.2 Hz).

(*S*)-2,5,7,8-Tetramethyl-2-((4*S*,8*S*)-4,8,12-trimethyltridecyl)chroman-6-yl4-(4,4-difluoro-5-oxotetrahydrofuran-2-yl)benzoate (13)

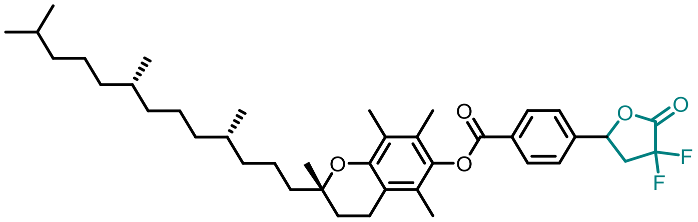

This compound was obtained according to general procedure **1** (*S*)-2,5,7,8-tetramethyl-2-((4*S*,8*S*)-4,8,12-trimethyltridecyl)chroman-6-yl 4-vinylbenzoate (280.4 mg, 0.5 mmol), *fac*-[Ir(ppy)$_3$] (3.3 mg, 1.0 mol%), *CDFA (88* µL, 1.0 mmol), and anhydrous DMF (1 mL). Isolated as a white crystalline solid (111.3 mg, 54% yield) after purification by flash column chromatography (12 g SiO_2, hexane/EA = 5:1). Spectroscopic data match with those reported in the literature.[13]

^{1}H-NMR (400 MHz, $CDCl_3$): δ 8.36 – 8.28 (m, 2H), 7.55 – 7.47 (m, 2H), 5.72 (dd, *J* = 8.8, 6.3 Hz, 1H), 3.30 – 3.15 (m, 1H), 2.75 – 2.57 (m, 3H), 2.12 (s, 3H), 2.05 (s, 3H), 2.01 (s, 3H), 1.89 – 1.77 (m, 3H), 1.61 – 1.45 (m, 5H), 1.34 – 1.18 (m, 12H), 1.17 – 1.04 (m, 6H), 0.90 – 0.82 (m, 12H).

^{13}C-NMR (101 MHz, $CDCl_3$): δ 164.6 (dd, *J* = 32.3, 32.2 Hz), 164.5, 149.8, 141.7, 140.6, 131.2, 131.0, 126.9, 125.7, 125.1, 123.4, 117.7, 115.4 (dd, *J* = 258.4, 250.8 Hz), 77.4, 76.3 (dd, *J* = 1.8, 2.2 Hz), 75.3, 40.1 (dd, *J* = 21.1, 21.3 Hz), 39.5, 37.7, 37.7, 37.6, 37.6, 37.5, 37.4, 32.9, 32.9, 28.1, 25.0, 25.0, 24.6, 22.9, 22.8, 21.2, 20.8, 19.9, 19.8, 19.8, 19.8, 19.8, 13.2, 12.3, 12.0.

^{19}F-NMR (376 MHz, $CDCl_3$): δ -106.1 (dd, *J* = 23.2, 15.7 Hz), -106.9 (dd, *J* = 23.5, 16.0 Hz), -108.5 (dd, *J* = 12.6, 4.6 Hz), -109.3 (dd, *J* = 12.6, 4.0 Hz).

1-Acetyl-3,3-difluoro-5-mesitylpyrrolidin-2-one (14)

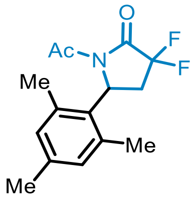

This compound was obtained according to general procedure **2** 1,3,5-trimethyl-2-vinylbenzene (73.1 mg, 0.5 mmol), *fac*-[Ir(ppy)$_3$] (3.3 mg, 1.0 mol%), *CDFA (88* µL, 1.0 mmol), Boc anhydride (218.3 mg, 1.0 mmol) and anhydrous MeCN (3 mL). Isolated as a white crystalline solid (60.5 mg, 43% yield) after purification by flash column chromatography (12 g SiO_2, hexane/EA = 20:1). Spectroscopic data match with those reported in the literature.[13]

^{1}H-NMR (300 MHz, $CDCl_3$): δ 6.84 (d, *J* = 25.4 Hz, 2H), 5.59 (td, *J* = 8.2, 2.7 Hz, 1H), 2.97 (dddd, *J* = 21.7, 15.7, 8.9, 2.9 Hz, 1H), 2.54 (s, 3H), 2.53 – 2.45 (m, 1H), 2.44 (s, 3H), 2.24 (s, 3H), 2.13 (s, 3H).

^{13}C-NMR (75 MHz, $CDCl_3$): δ 170.0, 164.4 (dd, *J* = 31.4, 31.0 Hz), 137.8, 136.5, 133.5, 132.1, 131.4 (d, *J* = 1.4 Hz), 130.1, 116.9 (dd, *J* = 256.5, 248.2 Hz), 51.5 (dd, *J* = 1.4, 1.4 Hz), 34.4 (dd, *J* = 22.7, 20.5 Hz), 25.3, 20.9, 20.8, 20.6.

^{19}F-NMR (282 MHz, $CDCl_3$): δ -99.7 (d, *J* = 275.7 Hz), -106.1 (d, *J* = 275.7 Hz).

Methyl 2-(1-acetyl-4,4-difluoro-5-oxopyrrolidin-2-yl)benzoate (15)

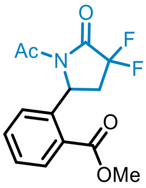

This compound was obtained according to general procedure **2** methyl 2-vinylbenzoate (81.0 mg, 0.5 mmol), *fac*-[Ir(ppy)$_3$] (3.3 mg, 1.0 mol%), *CDFA (88* µL, 1.0 mmol), Boc anhydride (218.3 mg, 1.0 mmol) and anhydrous MeCN (3 mL). Isolated as a white crystalline solid (72.8 mg, 49% yield) after purification by flash column chromatography (12 g SiO_2, hexane/EA = 20:1). Spectroscopic data match with those reported in the literature.[13]

^{1}H-NMR (300 MHz, $CDCl_3$): δ 8.07 (dd, *J* = 7.8, 1.5 Hz, 1H), 7.51 (td, *J* = 7.7, 1.6 Hz, 1H), 7.38 (td, *J* = 7.6, 1.3 Hz, 1H), 7.08 (d, *J* = 7.9 Hz, 1H), 6.22 (d, *J* = 9.3 Hz, 1H), 3.92 (s, 3H), 3.09 (ddd, *J* = 39.2, 15.4, 9.4 Hz, 1H), 2.64 (s, 3H), 2.60 – 2.43 (m, 1H).

^{13}C-NMR (75 MHz, $CDCl_3$): δ 170.0, 167.0, 164.9 (dd, *J* = 32.7, 30.5 Hz), 141.8, 133.2, 131.9, 128.1, 127.5, 124.0, 117.3 (dd, *J* = 250.9, 250.4 Hz), 52.7 (d, *J* = 6.9 Hz), 52.5, 37.7 (t, *J* = 21.1 Hz), 25.6.

^{19}F-NMR (282 MHz, $CDCl_3$): δ -99.1 (d, *J* = 279.2 Hz), -105.1 (d, *J* = 275.7 Hz).

1-Acetyl-5-(4-chlorophenyl)-3,3-difluoropyrrolidin-2-one (16)

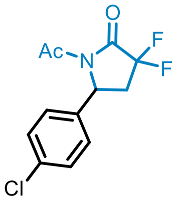

This compound was obtained according to general procedure **2** methyl 4-chlorostyrene (68.0 mg, 0.5 mmol), *fac*-[Ir(ppy)$_3$] (3.3 mg, 1.0 mol%), *CDFA (88* µL, 1.0 mmol), Boc anhydride (218.3 mg, 1.0 mmol) and anhydrous MeCN (3 mL). Isolated as a white crystalline solid (73.7 mg, 54% yield) after purification by flash column chromatography (12 g SiO_2, hexane/EA = 20:1). Spectroscopic data match with those reported in the literature.[13]

^{1}H-NMR (400 MHz, $CDCl_3$): δ 7.36 – 7.30 (m, 2H), 7.19 – 7.11 (m, 2H), 5.35 (dt, *J* = 9.3, 3.0 Hz, 1H), 3.01 – 2.84 (m, 1H), 2.59 (s, 3H), 2.56 – 2.44 (m, 1H).

^{13}C-NMR (101 MHz, $CDCl_3$): δ 170.1, 166.9 – 161.3 (m), 138.0, 134.5, 129.5, 127.1 (d, *J* = 1.7 Hz), 117.1 (dd, *J* = 254.1, 251.3 Hz), 54.2 (dd, *J* = 5.2, 1.8 Hz), 37.3 (t, *J* = 21.4 Hz), 25.6.

^{19}F-NMR (471 MHz, $CDCl_3$): δ -99.5 (d, *J* = 276.5 Hz), -104.0 (d, *J* = 276.5 Hz).

1-Acetyl-5-(4-chlorophenyl)-3,3-difluoropyrrolidin-2-one (17)

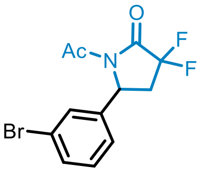

This compound was obtained according to general procedure **2** methyl 3-bromoostyrene (91.5 mg, 0.5 mmol), *fac*-[Ir(ppy)$_3$] (3.3 mg, 1.0 mol%), *CDFA (88* µL, 1.0 mmol), Boc anhydride (218.3 mg, 1.0 mmol) and anhydrous MeCN (3 mL). Isolated as a white crystalline solid (73.7 mg, 48% yield) after

purification by flash column chromatography (12 g SiO_2, hexane/EA = 20:1). Spectroscopic data match with those reported in the literature.[13]

^{1}H-NMR (500 MHz, $CDCl_3$): δ 7.45 (ddd, *J* = 8.0, 1.9, 0.9 Hz, 1H), 7.35 (t, *J* = 1.9 Hz, 1H), 7.27 – 7.21 (m, 1H), 7.13 (dt, *J* = 8.0, 1.9 Hz, 1H), 5.33 (dt, *J* = 9.4, 3.0 Hz, 1H). 3.01 – 2.85 (m, 1H), 2.61 (s, 3H), 2.58 – 2.44 (m, 1H).

^{13}C-NMR (126 MHz, $CDCl_3$): δ 170.0, 164.0 (t, *J* = 32.3 Hz), 141.8, 131.8, 130.8, 128.9 (d, *J* = 1.6 Hz), 124.2 (d, *J* = 1.7 Hz), 123.3, 117.0 (dd, *J* = 254.1, 251.6 Hz), 54.2 (dd, *J* = 5.1, 1.5 Hz), 37.3 (t, *J* = 21.6 Hz), 25.6.

^{19}F-NMR (471 MHz, $CDCl_3$): δ -99.6 (d, *J* = 276.5 Hz), -104.0 (d, *J* = 276.5 Hz).

1-Acetyl-3,3-difluoro-5-(4-iodophenyl)-5-methylpyrrolidin-2-one (18)

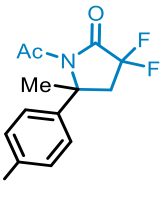

This compound was obtained according to general procedure **2** 1-iodo-4-(prop-1-en-2-yl)benzene (122.0 mg, 0.5 mmol), *fac*-[Ir(ppy)$_3$] (3.3 mg, 1.0 mol%), *CDFA (88* µL, 1.0 mmol), Boc anhydride (218.3 mg, 1.0 mmol) and anhydrous MeCN (3 mL). Isolated as a white crystalline solid (72.0 mg, 38% yield) after purification by flash column chromatography (12 g SiO_2, hexane/EA = 20:1). Spectroscopic data match with those reported in the literature.[13]

^{1}H-NMR (300 MHz, $CDCl_3$): δ 7.76 – 7.63 (m, 2H), 7.04 – 6.93 (m, 2H), 2.72 – 2.58 (m, 2H), 2.56 (s, 3H), 1.98 (s, 3H).

^{13}C-NMR (75 MHz, $CDCl_3$): δ 170.3, 164.7 (dd, *J* = 31.8, 31.7 Hz), 143.2 (d, *J* = 1.7 Hz), 138.2, 126.4, 116.2 (dd, *J* = 253.1, 250.3 Hz), 93.4, 62.3 (dd, *J* = 4.4, 2.2 Hz), 47.1 (t, *J* = 20.0 Hz), 26.9, 25.2.

^{19}F-NMR (282 MHz, $CDCl_3$): δ -101.4 (d, *J* = 277.4 Hz), -103.8 (d, *J* = 275.7 Hz).

1-(Acetyl-*d*$_3$)-5-(4-(*tert*-butyl)phenyl)-3,3-difluoropyrrolidin-2-one (19)

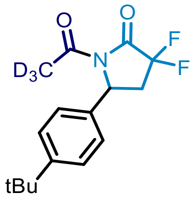

This compound was obtained according to general procedure **2** 4-*tert*-butylstyrene (90.0 µL, 0.5 mmol, 0.5 mmol), *fac*-[Ir(ppy)$_3$] (3.3 mg, 1.0 mol%), *CDFA (88* µL, 1.0 mmol), Boc anhydride (218.3 mg, 1.0 mmol) and anhydrous CD_3CN (3 mL). Isolated as a white crystalline solid (79.1 mg, 53% yield) after purification by flash column chromatography (12 g SiO_2, hexane/EA = 20:1). Spectroscopic data match with those reported in the literature.[13]

^{1}H-NMR (300 MHz, $CDCl_3$): δ 7.36 (d, *J* = 8.5 Hz, 2H), 7.12 (d, *J* = 8.3 Hz, 2H), 5.37 (dt, *J* = 9.4, 3.0 Hz, 1H), 2.90 (dddd, *J* = 19.6, 16.6, 15.0, 9.3 Hz, 1H), 2.54 (dddd, *J* = 17.9, 15.2, 7.1, 3.0 Hz, 2H), 1.30 (s, 9H).

^{13}C-NMR (101 MHz, $CDCl_3$): 170.1, 164.2 (t, J = 32.2 Hz), 151.3, 136.3, 126.0, 125.3 – 125.1 (m), 117.3 (dt, J = 14229.9, 252.1 Hz), 54.36 (dd, J = 4.9, 1.6 Hz), 37.34 (d, J = 21.2 Hz), 34.6, 31.3, 24.89 (dt, J = 40.1, 19.9 Hz).

^{19}F-NMR (282 MHz, $CDCl_3$): δ -99.6 (d, J = 275.7 Hz), -103.6 (d, J = 275.7 Hz).

1-Acetyl-5,5-bis(4-chlorophenyl)-3,3-difluoropyrrolidin-2-one (20)

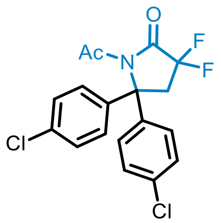

This compound was obtained according to general procedure **2** 4,4'-(ethene-1,1-diyl)bis(chlorobenzene) (124.5 μL, 0.5 mmol, 0.5 mmol), *fac*-[Ir(ppy)$_3$] (3.3 mg, 1.0 mol%), *CDFA (88* μL, 1.0 mmol), Boc anhydride (218.3 mg, 1.0 mmol) and CH_3CN (3 mL). Isolated as a white solid (63.4 mg, 33% yield) after purification by flash column chromatography (12 g SiO_2, hexane/EA = 20:1). Spectroscopic data match with those reported in the literature.[13]

^{1}H-NMR (400 MHz, $CDCl_3$): δ 7.40 – 7.30 (m, 4H), 7.21 – 7.12 (m, 4H), 3.19 (t, J = 14.1 Hz, 2H), 2.58 (s, 3H).

^{13}C-NMR (101 MHz, $CDCl_3$): δ 170.3, 164.7, 138.6, 134.5, 129.2, 128.8, 115.8 (t, J = 252.0 Hz), 68.6, 49.3 (t, J = 20.3 Hz).

^{19}F-NMR (376 MHz, $CDCl_3$): δ -103.6 (t, J = 14.0 Hz).

1-Acetyl-3,3-difluoro-3,3a,4,8b-tetrahydroindeno[1,2-b]pyrrol-2(1*H*)-one (21)

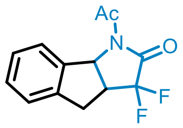

This compound was obtained according to general procedure **2**, 1*H*-indene (58 μL, 0.5 mmol, 0.5 mmol), *fac*-[Ir(ppy)$_3$] (3.3 mg, 1.0 mol%), *CDFA (88* μL, 1.0 mmol), Boc anhydride (218.3 mg, 1.0 mmol) and CH_3CN (3 mL). Isolated as a white solid (55.2 mg, 44% yield) after purification by flash column chromatography (12 g SiO_2, hexane/EA = 20:1).

^{1}H-NMR (400 MHz, $CDCl_3$): δ 7.57 (d, J = 7.7 Hz, 1H), 7.35 – 7.26 (m, 2H), 7.26 – 7.19 (m, 1H), 5.86 (dd, J = 8.0, 2.5 Hz, 1H), 3.44 – 3.17 (m, 3H), 2.61 (s, 3H).

^{13}C-NMR (101 MHz, $CDCl_3$): δ 164.1 (t, J = 32.9 Hz), 142.1, 139.0, 129.8, 127.8, 127.5, 124.9, 117.4 (dd, J = 258.0, 252.9 Hz), 61.8 (d, J = 4.9 Hz), 42.4 (dd, J = 23.0, 18.9 Hz), 29.7 (dd, J = 8.7, 2.1 Hz), 25.7.

^{19}F-NMR (376 MHz, $CDCl_3$): δ -102.4 (dd, J = 276.1, 17.8 Hz), -111.7 (dd, J = 276.1, 17.8 Hz).

HRMS (ESI) *m/z*, $[M+Na]^+$ calcd for $C_{13}H_{11}F2O_2+Na^+$: 274.0650; found: 274.0655.

1'-Acetyl-4',4'-difluoro-6,7,8,9-tetrahydrospiro[benzo[7]annulene-5,2'-pyrrolidin]-5'-one (22)

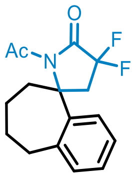

This compound was obtained according to general procedure **2** 5-methylene-6,7,8,9-tetrahydro-5H-benzo[7]annulene (136.1 mg, 0.5 mmol), *fac*-[Ir(ppy)$_3$] (3.3 mg, 1.0 mol%), *CDFA (88* µL, 1.0 mmol), boc$_2$O (218.3 mg, 1.0 mmol) and anhydrous MeCN (3 mL). Isolated as a white crystalline solid (54.3 mg, 37% yield) after purification by flash column chromatography (12 g SiO$_2$, hexane/EA = 20:1). Spectroscopic data match with those reported in the literature.[13]

^{1}H-NMR (300 MHz, CDCl$_3$): δ 7.24 – 7.07 (m, 3H), 6.89 – 6.78 (m, 1H), 3.11 – 2.95 (m, 2H), 2.90 – 2.74 (m, 3H), 2.65 (s, 3H), 2.05 – 1.93 (m, 1H), 1.92 – 1.77 (m, 3H), 1.77 – 1.68 (m, 1H).

^{13}C-NMR (75 MHz, CDCl$_3$): δ 171.1, 163.7 (dd, *J* = 31.3, 28.1 Hz), 140.3, 139.3, 132.1, 127.9, 126.6, 125.8, 116.3 (t, *J* = 251.1 Hz), 68.7 (dd, *J* = 2.5, 2.2 Hz), 42.5 (t, *J* = 19.9 Hz), 37.8, 36.1, 27.4, 26.8, 24.9.

^{19}F-NMR (282 MHz, CDCl$_3$): δ -102.4 (d, *J* = 274.0 Hz), -104.4 (d, *J* = 275.7 Hz).

1-Acetyl-3,3-difluoro-5-(4-((((8*R*,9*S*,13*S*,14*S*)-13-methyl-17-oxo-7,8,9,11,12,13,14,15,16,17-decahydro-6*H*-cyclopenta[*a*]phenanthren-3-yl)oxy)methyl)phenyl)pyrrolidin-2-one (23)

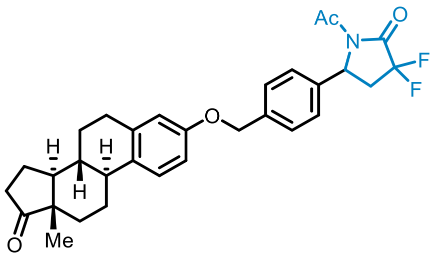

This compound was obtained according to general procedure **2** (8*R*,9*S*,13*S*,14*S*)-13-methyl-3-((4-vinylbenzyl)oxy)-6,7,8,9,11,12,13,14,15,16-decahydro-17*H*-cyclopenta[*a*]phenanthren-17-one (193.1 mg, 0.5 mmol), *fac*-[Ir(ppy)$_3$] (3.3 mg, 1.0 mol%), *CDFA (88* µL, 1.0 mmol), Boc anhydride (218.3 mg, 1.0 mmol) and anhydrous MeCN (3 mL). Isolated as a white crystalline solid (57.4 mg, 22% yield) after purification by flash column chromatography (12 g SiO$_2$, hexane/EA = 20:1). Spectroscopic data match with those reported in the literature.[13]

^{1}H-NMR (300 MHz, CDCl$_3$): δ 7.46 – 7.40 (m, 2H), 7.24 – 7.18 (m, 3H), 6.82 – 6.68 (m, 2H), 5.39 (dt, *J* = 9.2, 2.8 Hz, 1H), 5.01 (s, 2H), 3.08 – 2.78 (m, 3H), 2.60 (s, 3H), 2.58 – 2.44 (m, 2H), 2.20 – 1.90 (m, 5H), 1.69 – 1.40 (m, 7H), 0.91 (s, 3H).

^{13}C-NMR (101 MHz, CDCl$_3$): δ 221.1, 170.1, 164.2 (dd, *J* = 31.8, 31.5 Hz), 156.8, 139.2, 138.0, 137.7, 132.7, 128.3, 126.6, 125.9 (d, *J* = 1.7 Hz), 117.3 (dd, *J* = 252.0, 251.6 Hz), 115.0, 112.5 (d, *J* = 1.6 Hz), 69.5, 54.6 (dd, *J* = 1.5, 1.5 Hz), 50.6, 48.2, 44.1, 38.5, 37.5 (t, *J* = 21.4 Hz), 36.0, 31.7, 29.8, 26.7, 26.1, 25.7, 21.7, 14.0.

^{19}F-NMR (282 MHz, CDCl$_3$): δ -99.6 (d, *J* = 275.7 Hz), -103.8 (d, *J* = 275.7 Hz).

Methyl 2,2-difluoro-6-phenylhexanoate (3)

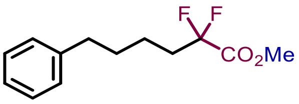

This compound was obtained according to general procedure **3** from but-3-en-1-ylbenzene (95.0 µL, 0.5 mmol), *fac*-[Ir(ppy)$_3$] (3.3 mg, 1.0 mol%), sodium ascorbate (198 mg, 1.0 mmol), methanol (1.5 mL), anhydrous MeCN (1.5 mL), and CDFA (88 µL, 1.0 mmol). Isolated as a transparent gel (96.0 mg, 79% yield) after purification by flash column chromatography (12 g SiO_2, hexane/EA = 10:1). Spectroscopic data match with those reported in the literature.[15]

^{1}H-NMR (300 MHz, $CDCl_3$): δ 7.35 – 7.22 (m, 2H), 7.23 – 7.13 (m, 3H), 3.86 (s, 3H), 2.63 (t, *J* = 7.6 Hz, 2H), 2.20 – 1.98 (m, 2H), 1.77 – 1.60 (m, 2H), 1.61 – 1.38 (m, 2H).

^{19}F-NMR (282 MHz, $CDCl_3$): δ-105.7 (t, *J* = 16.9 Hz).

Methyl 2,2-difluoro-5-phenylpentanoate (24)

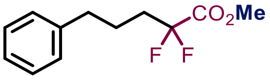

This compound was obtained according to general procedure **3** from allylbenzene (66.0 µL, 0.5 mmol), *fac*-[Ir(ppy)$_3$] (3.3 mg, 1.0 mol%), sodium ascorbate (198 mg, 1.0 mmol), anhydrous methanol (1.5 mL), anhydrous MeCN (1.5 mL), and CDFA (88 µL, 1.0 mmol). Isolated as a transparent gel (82.0 mg, 72% yield) after purification by flash column chromatography (12 g SiO_2, hexane/EA = 10:1). Spectroscopic data match with those reported in the literature.[16]

^{1}H-NMR (300 MHz, $CDCl_3$): δ 7.26 – 7.18 (m, 2H), 7.17 – 7.07 (m, 3H), 3.78 (s, 3H), 2.61 (t, *J* = 7.6 Hz, 2H), 2.12 – 1.89 (m, 2H), 1.83 – 1.66 (m, 2H).

^{13}C-NMR (75 MHz, $CDCl_3$): δ 168.1 – 161.0 (m), 141.0, 128.6, 128.5, 126.3, 116.5 (t, *J* = 253.8 Hz), 53.4, 35.2, 33.9 (d, *J* = 23.2 Hz), 23.2 (t, *J* = 4.2 Hz).

^{19}F-NMR (282 MHz, $CDCl_3$): δ -105.7 (t, *J* = 16.8 Hz)

Methyl 2,2-difluoro-5-phenoxypentanoate (25)

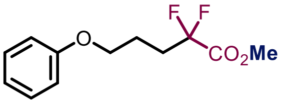

This compound was obtained according to general procedure **3** from (allyloxy)benzene (67.0 µL, 0.5 mmol), *fac*-[Ir(ppy)$_3$] (3.3 mg, 1.0 mol%), sodium ascorbate (198 mg, 1.0 mmol), anhydrous methanol (1.5 mL), anhydrous MeCN (1.5 mL), and CDFA (88 µL, 1.0 mmol). Isolated as a transparent gel (68.3 mg, 56% yield) after purification by flash column chromatography (12 g SiO_2, hexane/EA = 10:1).

^{1}H-NMR (400 MHz, $CDCl_3$): δ 7.3 – 7.3 (m, 2H), 7.0 – 6.9 (m, 1H), 6.9 – 6.9 (m, 2H), 4.0 (t, *J* = 6.1 Hz, 2H), 3.9 (s, 3H), 2.4 – 2.2 (m, 2H), 2.1 – 1.9 (m, 2H).

^{13}C-NMR (101 MHz, $CDCl_3$): δ 164.8 (t, *J* = 33.1 Hz), 158.8, 129.6, 121.1, 116.4 (t, *J* = 250.1 Hz), 114.6, 66.5, 53.5, 31.6 (t, *J* = 23.6 Hz), 21.9.

^{19}F-NMR (376 MHz, $CDCl_3$): -105.9 (t, *J* = 17.1 Hz)

HRMS (ESI) *m/z*, $[M+Na]^+$ calcd for $C_{12}H_{14}O_3F_2Na^+$: 267.0803; found: 267.0799.

Diethyl (1*R*,2*R*)-4-(1,1-difluoro-2-methoxy-2-oxoethyl)cyclohexane-1,2-dicarboxylate (26)

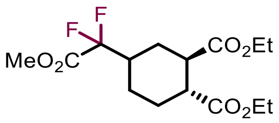

This compound was obtained according to general procedure **3** from diethyl (1*R*,2*R*)-cyclohex-4-ene-1,2-dicarboxylate (100.0 μL, 0.5 mmol), *fac*-[Ir(ppy)$_3$] (3.3 mg, 1.0 mol%), sodium ascorbate (198 mg, 1.0 mmol), anhydrous methanol (1.5 mL), anhydrous MeCN (1.5 mL), and CDFA (88 μL, 1.0 mmol). Isolated as a transparent gel (101.0 mg, 60% yield) after purification by flash column chromatography (12 g SiO_2, hexane/EA = 10:1). Crude NMR indicated the d.r. ratio 10:1.

^{1}H-NMR (300 MHz, $CDCl_3$): δ 4.28 – 4.07 (m, 4H), 3.87 (s, 3H), 3.24 (q, *J* = 4.5 Hz, 1H), 3.06 (q, *J* = 4.3 Hz, 1H), 2.40 – 2.20 (m, 1H), 2.21 – 1.98 (m, 2H), 1.80 – 1.57 (m, 3H), 1.52 – 1.33 (m, 1H), 1.32 – 1.20 (m, 6H).

^{13}C-NMR (101 MHz, $CDCl_3$): 173.4, 164.6 (t, *J* = 33.1 Hz), 117.3 (t, *J* = 253.1 Hz), 61.0 (d, *J* = 5.5 Hz), 53.4, 41.1, 40.2, 37.9 (t, *J* = 22.1 Hz), 23.6, 23.4 (t, *J* = 4.1 Hz), 21.3 (t, *J* = 3.8 Hz), 14.3 (d, *J* = 2.2 Hz).

^{19}F-NMR (282 MHz, $CDCl_3$): δ -112.4 (d, *J* = 14.9 Hz)

HRMS (ESI) *m/z*, $[M+H]^+$ calcd for $C_{15}H_{23}O_6F_2^+$: 337.1457; found: 337.1450.

3,3-Difluoro-4-methoxy-4-oxobutyl 4-(tert-butyl)benzoate (27)

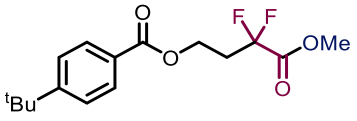

This compound was obtained according to general procedure **3** from vinyl 4-(*tert*-butyl)benzoate (100.0 μL, 0.5 mmol), *fac*-[Ir(ppy)$_3$] (3.3 mg, 1.0 mol%), sodium ascorbate (198 mg, 1.0 mmol), methanol (1.5 mL), anhydrous MeCN (1.5 mL), and CDFA (88 μL, 1.0 mmol). Isolated as a transparent gel (96.0 mg, 61% yield) after purification by flash column chromatography (12 g SiO_2, hexane/EA = 10:1).

^{1}H-NMR (400 MHz, $CDCl_3$): δ 7.93 (d, *J* = 8.6 Hz, 1H), 7.46 (d, *J* = 8.7 Hz, 2H), 4.52 (t, *J* = 6.2 Hz, 2H), 3.78 (s, 3H), 2.60 (tt, *J* = 15.7, 6.2 Hz, 2H), 1.34 (s, 9H).

^{13}C-NMR (101 MHz, $CDCl_3$): δ 166.2, 164.4, 157.2, 129.7, 126.9, 125.6, 115.1*, 57.9 (d, *J* = 6.0 Hz), 53.6, 35.3, 34.4 (t, *J* = 23.7 Hz), 31.3.(*CF_2 signals are weak)

^{19}F-NMR (376 MHz, $CDCl_3$): δ -105.1(t, *J* = 15.5 Hz)

HRMS (ESI) *m/z*, $[M+NH_4]^+$ calcd for $C_{16}H_{20}O_4F_2$+NH_4^+: 332.1668; found: 332.1661.

Methyl 2-((3S,8R,9S,10R,13S,14S,17S)-3-acetoxy-17-acetyl-10,13-dimethylhexadecahydro-1H-cyclopenta[a]phenanthren-6-yl)-2,2-difluoroacetate (28)

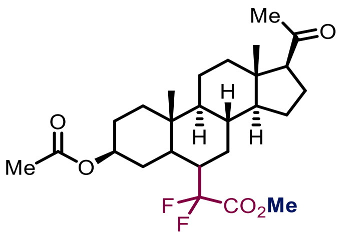

This compound was obtained according to general procedure **3** from Pregnenolone acetate (180.0 mg, 0.5 mmol), *fac*-[Ir(ppy)$_3$] (3.3 mg, 1.0 mol%), sodium ascorbate (198 mg, 1.0 mmol), methanol (1.5

mL), anhydrous MeCN (1.5 mL), and CDFA (88 μL, 1.0 mmol). Isolated as a transparent gel (122.0 mg, 52% yield) after purification by flash column chromatography (12 g SiO_2, hexane/EA = 10:1).

^{1}H-NMR (400 MHz, $CDCl_3$): δ 4.62 – 4.47 (m, 1H), 3.78 (s, 3H), 2.48 – 2.29 (m, 2H), 2.16 – 2.07 (m, 1H), 2.04 (s, 3H), 1.96 (s, 3H), 1.84 – 1.70 (m, 4H), 1.68 – 1.37 (m, 8H), 1.36 – 0.96 (m, 6H), 0.94 – 0.88 (m, 3H), 0.68 – 0.59 (m, 1H), 0.57 (s, 3H).

^{13}C-NMR (101 MHz, $CDCl_3$): δ 209.4, 170.8, 165.6 (dd, *J* = 34.7, 32.6 Hz), 118.6 (dd, J = 258.3, 256.5 Hz), 74.2, 63.9, 56.6, 54.8, 53.4, 45.8, 44.3, 41.2 (dd, *J* = 22.0, 19.6 Hz), 39.8, 39.0, 35.2, 32.7 (d, *J* = 5.9 Hz), 32.2 (d, *J* = 2.5 Hz), 31.7 (d, *J* = 5.7 Hz), 31.6, 27.6, 24.4, 22.9, 21.5, 21.2, 15.0 (dd, *J* = 9.6, 3.5 Hz), 13.7.

^{19}F-NMR (376 MHz, $CDCl_3$): δ -99.8 (dd, *J* = 255.1, 12.1 Hz), -103.2 (dd, *J* = 255.1, 29.6 Hz).

HRMS (ESI) *m/z*, $[M+Na]^+$ calcd for $C_{26}H_{38}O_5F_2Na^+$: 491.2580; found: 491.2565.

6,6-Difluoro-7-methoxy-7-oxoheptyl benzoate (29)

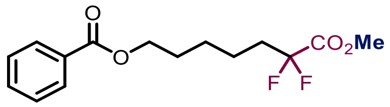

This compound was obtained according to general procedure **1** from pent-4-en-1-yl benzoate (95.0 μL, 0.5 mmol), *fac*-[Ir(ppy)$_3$] (3.3 mg, 1.0 mol%), sodium ascorbate (198 mg, 1.0 mmol), anhydrous MeOH (1.5 mL), anhydrous MeCN (1.5 mL), and *CDFA* (88 μL, 1.0 mmol). Isolated as a transparent gel (108.0 mg, 71% yield) after purification by flash column chromatography (12 g SiO_2, hexane/EA = 10:1).

^{1}H-NMR (400 MHz, $CDCl_3$): δ 8.08 – 7.99 (m, 2H), 7.60 – 7.51 (m, 1H), 7.49 – 7.39 (m, 2H), 4.32 (t, *J* = 6.5 Hz, 2H), 3.86 (s, 3H), 2.19 – 2.01 (m, 2H), 1.79 (dq, *J* = 7.9, 6.6 Hz, 2H), 1.63 – 1.45 (m, 4H).

^{13}C-NMR (101 MHz, $CDCl_3$): δ 166.7, 164.9 (t, *J* = 33.2 Hz), 133.0, 130.5, 129.7, 128.5, 116.4 (t, *J* = 250.0 Hz), 64.7, 53.4, 34.6 (t, *J* = 23.2 Hz), 28.6, 25.8, 21.4 (t, *J* = 4.3 Hz).

^{19}F-NMR (376 MHz, $CDCl_3$): δ -105.7 (t, *J* = 16.5 Hz).

7-Ethoxy-6,6-difluoro-7-oxoheptyl benzoate (30)

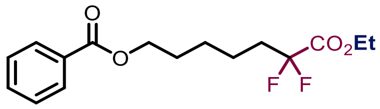

This compound was obtained according to general procedure **3** from pent-4-en-1-yl benzoate (95.0 μL, 0.5 mmol), *fac*-[Ir(ppy)$_3$] (3.3 mg, 1.0 mol%), sodium ascorbate (198 mg, 1.0 mmol), anhydrous EtOH (1.5 mL), anhydrous MeCN (1.5 mL), and CDFA (88 μL, 1.0 mmol). Isolated as a transparent gel (121.0 mg, 77% yield) after purification by flash column chromatography (12 g SiO_2, hexane/EA = 10:1). Spectroscopic data match with those reported in the literature.[17]

^{1}H-NMR (300 MHz, $CDCl_3$): δ 8.08 – 7.99 (m, 2H), 7.60 – 7.52 (m, 1H), 7.49 – 7.39 (m, 2H), 4.39 – 4.25 (m, 4H), 2.20 – 1.98 (m, 2H), 1.80 (p, *J* = 6.7 Hz, 2H), 1.66 – 1.44 (m, 6H), 1.35 (t, *J* = 7.1 Hz, 3H).

^{19}F-NMR (282 MHz, $CDCl_3$): δ -106.0 (t, *J* = 16.6 Hz)

HRMS (ESI) *m/z*, $[M+NH_4]^+$ calcd for $C_{16}H_{20}O_4F_2+NH_4^+$: 332.1668; found: 332.1661.

6,6-Difluoro-7-isopropoxy-7-oxoheptyl benzoate (31)

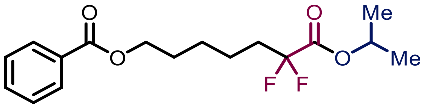

This compound was obtained according to general procedure **3** from pent-4-en-1-yl benzoate (95.0 µL, 0.5 mmol), *fac*-[Ir(ppy)$_3$] (3.3 mg, 1.0 mol%), sodium ascorbate (198 mg, 1.0 mmol), anhydrous isopropanol (1.5 mL), anhydrous MeCN (1.5 mL), and CDFA (88 µL, 1.0 mmol). Isolated as a transparent gel (113.0 mg, 69% yield) after purification by flash column chromatography (12 g SiO_2, hexane/EA = 10:1).

^{1}H-NMR (400 MHz, $CDCl_3$): δ 8.08 – 7.99 (m, 2H), 7.60 – 7.51 (m, 1H), 7.49 – 7.40 (m, 2H), 5.21 – 5.07 (m, 1H), 4.32 (t, J = 6.3 Hz, 2H), 2.16 – 2.00 (m, 2H), 1.85 – 1.74 (m, 2H), 1.63 – 1.46 (m, 3H), 1.32 (d, J = 6.3 Hz, 6H).

^{13}C-NMR (101 MHz, $CDCl_3$): 166.8, 164.0 (t, J = 32.9 Hz), 133.0, 130.5, 129.7, 128.5, 116.3 (t, J = 250.2 Hz), 71.1, 64.8, 34.5 (t, J = 23.3 Hz), 28.6, 25.8, 21.7, 21.4 (d, J = 4.4 Hz).

^{19}F-NMR (376 MHz, $CDCl_3$): δ -106.1 (t, J = 16.8 Hz)

HRMS (ESI) *m/z*, $[M+Na]^+$ calcd for $C_{17}H_{22}O_4F_2Na^+$: 351.1384; found: 351.1368.

6,6-Difluoro-7-(methoxy-*d*$_3$)-7-oxoheptyl benzoate (32)

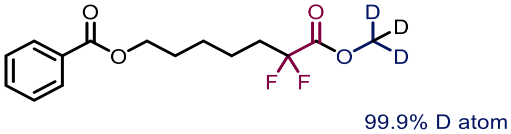

This compound was obtained according to general procedure **3** from pent-4-en-1-yl benzoate (95.0 µL, 0.5 mmol), *fac*-[Ir(ppy)$_3$] (3.3 mg, 1.0 mol%), sodium ascorbate (198 mg, 1.0 mmol), methanol-d$_4$ (1.5 mL, 99.9% from ampules sigma Aldrich), anhydrous MeCN (1.5 mL), and CDFA (88 µL, 1.0 mmol). Isolated as a transparent gel (109.0 mg, 72% yield) after purification by flash column chromatography (12 g SiO_2, hexane/EA = 10:1).

^{1}H-NMR (400 MHz, $CDCl_3$): δ 8.08 – 7.99 (m, 2H), 7.60 – 7.52 (m, 1H), 7.49 – 7.40 (m, 2H), 4.32 (t, J = 6.5 Hz, 2H), 2.18 – 2.00 (m, 2H), 1.85 – 1.74 (m, 2H), 1.63 – 1.45 (m, 3H).

^{13}C-NMR (101 MHz, $CDCl_3$): δ 166.8, 165.0 (t, J = 33.2 Hz), 133.0, 130.5, 129.7, 128.5, 116.4 (t, J = 250.0 Hz), 64.8, 39.8 – 37.2 (m), 34.6 (t, J = 23.2 Hz), 28.6, 25.8, 21.4.

^{19}F-NMR (282 MHz, $CDCl_3$): δ -105.7 (t, J = 16.9 Hz).

HRMS (ESI) *m/z*, $[M+H]^+$ calcd for $C_{15}H_{15}D_3F_2O_4^+$: 304.1427; found: 304.1427

5-(4-(*tert*-Butyl)phenyl)-3-fluorodihydrofuran-2(3*H*)-one (33)

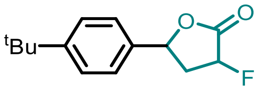

This compound was obtained according to general procedure **3** from 4-*tert*-butylstyrene (95.0 µL, 0.5 mmol), *fac*-[Ir(ppy)$_3$] (3.3 mg, 1.0 mol%), *chlorofluoroacetic acid* (74 µL, 1.0 mmol), and anhydrous DMF (1 mL). Collected 108 mg of diastereomeric mixture (1:1, 76% yield). After purification by flash column chromatography (12 g SiO_2, hexane/EA = 20:1). We were able to isolate single diastereomer as a white crystalline solid.

¹H-NMR (300 MHz, $CDCl_3$): δ 7.49 – 7.38 (m, 2H), 7.25 – 7.20 (m, 2H), 5.72 (t, *J* = 6.6 Hz, 1H), 5.23 (ddd, *J* = 51.5, 6.6, 4.5 Hz, 1H), 2.88 (dddd, *J* = 22.9, 14.3, 6.6, 4.5 Hz, 1H), 2.56 (ddt, *J* = 26.0, 14.3, 6.6 Hz, 1H), 1.33 (s, 3H).

¹³C-NMR (75 MHz, $CDCl_3$): δ 171.2 (d, *J* = 20.3 Hz), 152.3, 134.9, 126.1, 125.2, 86.5 (d, *J* = 185.8 Hz), 79.3 (d, *J* = 3.0 Hz), 37.7 (d, *J* = 20.3 Hz), 34.8, 31.4.

¹⁹F-NMR (282 MHz, $CDCl_3$): δ -194.1.

FT-IR (ATR, neat; cm^{-1}): 3005, 2961, 2866, 1779, 1275, 1261.

HRMS (ESI) *m/z*, $[M+H]^+$ calcd for $C_{14}H_{18}FO_2^+$: 237.1286, 238.1319; found: 237.1282, 238.1316.

5-(4-(*tert*-Butyl)phenyl)-3,3-difluorodihydrofuran-2(3*H*)-one (1)

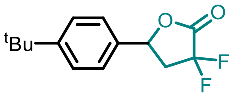

This compound was obtained according to general procedure **1** from 4-*tert*-butylstyrene (90.0 µL, 0.5 mmol), *fac*-[Ir(ppy)$_3$] (3.3 mg, 1.0 mol%), *CDFA (88* µL, 1.0 mmol) or *BDFA* (175.0 mg, 1.0 mmol), and anhydrous DMF (1 mL). Isolated as a white crystalline solid (133.0 mg, 90% yield for *CDFA*, 125.6 mg, 85% yield for *BDFA*) after purification by flash column chromatography (12 g SiO_2, hexane/EA = 20:1). Spectroscopic data match with those reported in the literature.[13]

¹H-NMR (300 MHz, $CDCl_3$): δ 7.51 – 7.41 (m, 2H), 7.33 – 7.23 (m, 2H), 5.59 (dd, *J* = 9.0, 6.2 Hz, 1H), 3.21 – 3.01 (m, 1H), 2.80 – 2.54 (m, 1H), 1.33 (s, 9H).

¹³C-NMR (126 MHz, $CDCl_3$): δ 165.1 (dd, *J* = 33.9, 31.8 Hz), 153.2, 133.2, 126.2, 125.8, 115.9 (dd, *J* = 259.0, 249.8 Hz), 77.3 – 77.2 (m), 40.0 (dd, *J* = 22.2, 20.5 Hz), 34.9, 31.4.

¹⁹F-NMR (282 MHz, $CDCl_3$): δ -106.5 (d, *J* = 279.1 Hz), -109.3 (d, *J* = 279.1 Hz).

5-(4-(*tert*-Butyl)phenyl)-3-(chloromethyl)dihydrofuran-2(3*H*)-one (34)

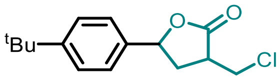

This compound was obtained according to general procedure **1** from 4-*tert*-butylstyrene (90.0 µL, 0.5 mmol), *fac*-[Ir(ppy)$_3$] (3.3 mg, 1.0 mol%), *2,3-dichloropropanoic acid* (94 µL, 1.0 mmol), and anhydrous DMF (1 mL). Collected 104 mg of diastereomeric mixture (1:1, 78% yield). After purification by flash column chromatography (12 g SiO_2, hexane/EA = 20:1). We were able to isolate single diastereomer as a white crystalline solid.

¹H-NMR (500 MHz, $CDCl_3$): δ 7.45 – 7.38 (m, 2H), 7.28 – 7.20 (m, 2H), 5.64 (dd, *J* = 8.4, 4.1 Hz, 1H), 3.92 – 3.79 (m, 2H), 3.19 – 3.10 (m, 1H), 2.82 – 2.73 (m, 1H), 2.55 – 2.46 (m, 1H), 1.32 (s, 1H).

¹³C-NMR (126 MHz, $CDCl_3$): δ 175.8, 151.7, 136.6, 125.9, 124.9, 79.1, 43.5, 41.6, 34.8, 34.0, 31.4.

FT-IR (ATR, neat; cm^{-1}): 2959, 2869, 2360, 1761, 1198, 1151.

HRMS (ESI) *m/z*, $[M+H]^+$ calcd for $C_{15}H_{20}ClO_2^+$: 267.1146; found: 267.1145.

5-(4-(*tert*-Butyl)phenyl)-3-chlorodihydrofuran-2(3*H*)-one (35)

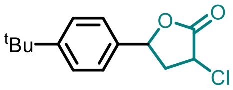

This compound was obtained according to general procedure **1** from 4-*tert*-butylstyrene (90.0 μL, 0.5 mmol, 1.0 equiv), *fac*-[Ir(ppy)$_3$] (3.3 mg, 5.0 μmol, 1.0 mol%), *2,2-dichloroacetic acid* (90 μL, 1.0 mmol, 2.0 equiv), and anhydrous DMF (15 mL). Collected 106 mg of diastereomeric mixture of white solids (1:1, 84% yield). After purification by flash column chromatography (24 g SiO_2, hexane/EA = 20:1).

diastereomer 1: **^{1}H-NMR** (500 MHz, $CDCl_3$): δ 7.47 – 7.41 (m, 1H), 7.34 – 7.26 (m, 1H), 5.41 (dd, *J* = 10.3, 5.6 Hz, 1H), 4.70 (dd, *J* = 11.2, 8.3 Hz, 1H), 3.19 (ddd, *J* = 13.2, 8.3, 5.6 Hz, 1H), 2.49 (ddd, *J* = 13.2, 11.2, 10.3 Hz, 1H), 1.33 (s, 5H).

^{13}C-NMR (126 MHz, $CDCl_3$): δ 172.0, 152.6, 134.3, 126.0, 125.8, 78.8, 51.8, 41.5, 34.8, 31.4.

diastereomer 2: **^{1}H-NMR** (500 MHz, $CDCl_3$): δ 7.46 – 7.39 (m, 2H), 7.29 – 7.24 (m, 2H), 5.75 (dd, *J* = 8.6, 5.8 Hz, 1H), 4.54 (dd, *J* = 6.7, 2.7 Hz, 1H), 2.80 (ddd, *J* = 14.4, 5.8, 2.7 Hz, 1H), 2.66 (ddd, *J* = 14.4, 8.6, 6.7 Hz, 1H), 1.32 (s, 9H).

^{13}C-NMR (126 MHz, $CDCl_3$): δ 172.0, 152.4, 134.3, 126.0, 125.5, 80.0, 51.5, 41.6, 34.9, 31.4.

FT-IR (ATR, neat; cm^{-1}):2958, 2901, 2862, 2162, 1783, 1514, 1179.

HRMS (ESI) *m/z*, $[M+H]^+$ calcd for $C_{14}H_{18}O_2Cl^+$: 253.0990; found: 253.0989.

5-(4-(*tert*-Butyl)phenyl)-3,3-dichlorodihydrofuran-2(3*H*)-one (36)

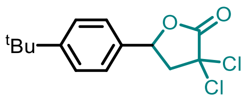

This compound was obtained according to general procedure **1** from 4-*tert*-butylstyrene (90.0 μL, 0.5 mmol), *fac*-[Ir(ppy)$_3$] (3.3 mg, 1.0 mol%), *trichloroacetic acid* (164.0 mg, 1.0 mmol), and anhydrous DMF (1 mL). Isolated as a white crystalline solid (116.0 mg, 81% yield) after purification by flash column chromatography (12 g SiO_2, hexane/EA = 20:1).

^{1}H-NMR (300 MHz, $CDCl_3$): δ 7.48 – 7.42 (m, 2H), 7.40 – 7.23 (m, 2H), 5.59 (dd, *J* = 10.1, 4.9 Hz, 1H), 3.41 (dd, *J* = 14.2, 5.0 Hz, 1H), 2.94 (dd, *J* = 14.3, 10.1 Hz, 1H), 1.33 (s, 9H).

^{13}C-NMR (75 MHz, $CDCl_3$): δ 167.7, 153.1, 132.5, 126.2, 125.9, 78.8, 77.7, 52.4, 34.9, 31.4.

FT-IR (ATR, neat; cm^{-1}): 2963, 2903, 2361, 2340, 1788, 1614, 1517, 1321.

3-bromo-5-(4-(*tert*-Butyl)phenyl)dihydrofuran-2(3*H*)-one (37)

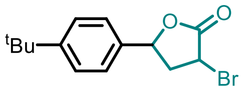

This compound was obtained according to general procedure **1** from 4-*tert*-butylstyrene (90.0 μL, 0.5 mmol), *fac*-[Ir(ppy)$_3$] (3.3 mg, 1.0 mol%), *dibromoacetic acid* (218 mg, 1.0 mmol), and anhydrous DMF (1 mL). Collected 131 mg of diastereomeric mixture (3:2, 88% yield). After purification by flash column chromatography (12 g SiO_2, hexane/EA = 20:1). We were able to isolate single diastereomer as a white crystalline solid.

^{1}H-NMR (300 MHz, $CDCl_3$): δ 7.49 – 7.38 (m, 2H), 7.35 – 7.27 (m, 2H), 5.73 (dd, *J* = 9.4, 5.4 Hz, 1H), 4.55 (dd, *J* = 6.5, 1.7 Hz, 1H), 2.82 (ddd, *J* = 14.9, 5.5, 1.7 Hz, 1H), 2.75 – 2.63 (m, 1H), 1.33 (s, 9H).

^{13}C-NMR (75 MHz, $CDCl_3$): δ 172.4, 152.4, 134.0, 126.0, 125.7, 80.4, 42.1, 39.1, 34.8, 31.3.

HRMS (EI) *m/z*, $[M]^+$ calcd for $C_{14}H_{17}BrO_2{}^+$: 296.0412, 298.0391, 297.0445, 299.0425; found: 296.0410, 298.0390, 297.0444, 299.0422.

(3*S*,5*R*)-5-(4-(*tert*-Butyl)phenyl)-3-methyldihydrofuran-2(3*H*)-one (38)

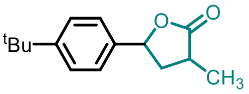

This Compound was obtained according to general procedure **1** from 4-*tert*-butylstyrene (90.0 µL, 0.5 mmol, 1.0 equiv), *fac*-[Ir(ppy)$_3$] (3.3 mg, 5.0 µmol, 1.0 mol%), *2-bromopropanoic acid* (45 µL, 1.0 mmol, 2.0 equiv), and anhydrous DMF (1 mL). Collected 97 mg of diastereomeric mixture of white solids (1:1, 83% yield). After purification by flash column chromatography (24 g SiO_2, hexane/EA = 20:1).

diastereomer 1: **^{1}H-NMR** (500 MHz, $CDCl_3$): δ 7.43 – 7.37 (m, 2H), 7.27 – 7.21 (m, 2H), 5.55 (dd, *J* = 7.8, 4.6 Hz, 1H), 2.80 – 2.69 (m, 1H), 2.50 – 2.41 (m, 1H), 2.39 – 2.29 (m, 1H), 1.33 (s, 3H), 1.32 (s, 9H).

^{13}C-NMR (126 MHz, $CDCl_3$): δ 180.1, 151.4, 136.8, 125.7, 124.9, 78.4, 38.3, 34.7, 33.8, 31.4, 15.5.

diastereomer 2: **^{1}H-NMR** (500 MHz, $CDCl_3$): δ 7.44 – 7.38 (m, 2H), 7.32 – 7.26 (m, 2H), 5.34 (dd, *J* = 10.7, 5.2 Hz, 1H), 2.88 – 2.73 (m, 3H), 1.93 – 1.82 (m, 1H), 1.34 (s, 3H), 1.32 (s, 9H).

^{13}C-NMR (126 MHz, $CDCl_3$): δ 179.3, 151.7, 136.1, 125.7, 125.5, 79.3, 39.9, 36.5, 34.7, 31.4, 15.1.

FT-IR (ATR, neat; cm^{-1}): 2955, 2899, 2867, 1770, 1457, 1360, 1168.

HRMS (ESI) *m/z*, $[M+H]^+$ calcd for $C_{15}H_{21}O_2^+$: 233.1536; found: 233.1536.

2-Bromo-3,4,5-trimethoxy-1-(prop-1-en-1-yl)benzene (39a)

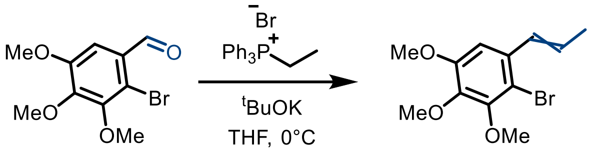

This compound was obtained according to following procedure:

To a suspension of 2-bromo-3,4,5-trimethoxybenzaldehyde (0.59 g, 2.14 mmol), ethyltriphenylphosphonium bromide (1.20 g, 3.2 mmol) in THF (25 ml) was slowly added portion wise at 0°C tBuOK powder (0.34 g, 3.0 mmol) under N_2 atmosphere. The reaction mixture was allowed to warm at room temperature for 6h. The completion of the reaction was monitored by TLC, then 10 ml of saturated NH_4Cl solution was added. Reaction mixture was diluted by 40 ml of distilled H_2O, extracted 3x25 ml EtOAc. Combined organic phase was dried with $MgSO_4$, filtered, evaporated and purified by flash column chromatography (12g SiO_2, hexane/EA = 20:1). 2-Bromo-3,4,5-trimethoxy-1-(prop-1-en-1-yl)benzene was isolated as a yellowish oil (0.45 g, 73% yield, *E*/*Z* ratio 3:2).

^{1}H-NMR (300 MHz, $CDCl_3$): δ 6.82 (s, 1H), 6.71 (dq, *J* = 15.6, 1.8 Hz, 1H), 6.66 (s, 1.5H), 6.43 (dqd, *J* = 11.4, 1.8, 0.6 Hz, 1.5H), 6.09 (dq, *J* = 15.5, 6.6 Hz, 1H), 5.85 (dq, *J* = 11.4, 7.1 Hz, 1.5H), 3.91 – 3.89 (m, 9H), 3.89 – 3.86 (m, 9H), 3.85 (s, 4.5H), 1.92 (dd, *J* = 6.6, 1.8 Hz, 3H), 1.80 (dd, *J* = 7.1, 1.8 Hz, 4.5H).

^{13}C-NMR (75 MHz, $CDCl_3$): δ 152.8, 152.2, 151.1, 150.9, 142.4, 142.0, 133.6, 133.1, 130.1, 129.6, 128.3, 127.8, 110.5, 109.9, 109.6, 105.5, 61.3, 61.1, 61.0, 56.3, 56.2, 18.7, 14.6.

HRMS (EI) *m/z*, $[M]^+$ calcd for $C_{12}H_{15}BrO_3^+$: 286.0205, 288.0184, 287.0238 289.0218; found: 286.0202, 288.0181, 287.0236, 289.0215.

5-(2-Bromo-3,4,5-trimethoxyphenyl)-3,4-dimethyldihydrofuran-2(3*H*)-one (39)

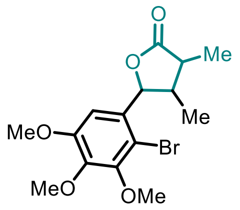

This compound was obtained according to general procedure **1** from 2-bromo-3,4,5-trimethoxy-1-(prop-1-en-1-yl)benzene (**29a**) (143.6 mg, 0.5 mmol), *fac*-[Ir(ppy)$_3$] (3.3 mg, 1.0 mol%), *2-bromopropionic acid* (90 µL, 1.0 mmol), and anhydrous DMF (1 mL). Collected 53.9 mg of diastereomeric mixture (4:1, 30% yield). After purification by flash column chromatography (12 g SiO_2, hexane/EA = 20:1). We were able to isolate single diastereomer as a white crystalline solid. Spectroscopic data match with those reported in the literature.[20]

^{1}H-NMR (300 MHz, $CDCl_3$): δ 6.69 (s, 1H), 5.44 (d, *J* = 9.4 Hz, 1H), 3.90 (s, 3H), 3.89 (s, 3H), 3.86 (s, 3H), 2.40 (dq, *J* = 11.6, 7.0 Hz, 1H), 2.00 (ddq, *J* = 11.5, 9.4, 6.6 Hz, 1H), 1.30 (d, *J* = 7.1 Hz, 3H), 1.22 (d, *J* = 6.6 Hz, 3H).

^{13}C-NMR (75 MHz, $CDCl_3$): δ 178.7, 153.5, 150.8, 143.6, 132.7, 109.9, 106.2, 84.2, 61.2, 61.2, 56.5, 49.0, 43.5, 15.2, 13.2.

HRMS (ESI) *m/z*, $[M+H]^+$ calcd for $C_{15}H_{20}BrO_5{}^+$: 359.0489, 361.0469, 360.0523, 362.0502; found: 359.0489, 361.0467, 360.0523, 362.0500.

5-(4-(*tert*-Butyl)phenyl)-3-isopropyldihydrofuran-2(3*H*)-one (40)

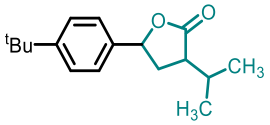

This compound was obtained according to general procedure **1** from 4-*tert*-butylstyrene (94%, 95.0 µL, 0.5 mmol, 1.0 equiv), *fac*-[Ir(ppy)$_3$] (3.3 mg, 5.0 µmol, 1.0 mol%), *2-chloro-3-methylbutanoic acid* (134 µL, 1.0 mmol, 2.0 equiv), and anhydrous DMF (1 mL). Collected 115 mg of diastereomeric mixture of white solids (1:1, 89% yield). After purification by flash column chromatography (24 g SiO_2, hexane/EA = 20:1).

diastereomer 1: **^{1}H-NMR** (500 MHz, $CDCl_3$): δ 7.43 – 7.37 (m, 2H), 7.28 – 7.21 (m, 2H), 5.50 (dd, *J* = 8.2, 4.7 Hz, 1H), 2.61 (ddd, *J* = 9.3, 8.2, 5.3 Hz, 1H), 2.51 (dt, *J* = 13.0, 8.2 Hz, 1H), 2.31 – 2.17 (m, 2H), 1.32 (s, 9H), 1.06 (d, *J* = 6.8 Hz, 3H), 1.00 (d, *J* = 6.8 Hz, 3H).

^{13}C-NMR (126 MHz, $CDCl_3$): δ 178.7, 151.3, 137.4, 125.7, 124.9, 78.7, 45.0, 34.7, 32.4, 31.4, 28.5, 20.7, 18.9.

diastereomer 2:**^{1}H-NMR** (500 MHz, $CDCl_3$): δ δ 7.45 – 7.37 (m, 2H), 7.31 – 7.28 (m, 2H), 5.31 (dd, *J* = 10.7, 5.9 Hz, 1H), 2.78 – 2.69 (m, 1H), 2.63 – 2.46 (m, 1H), 2.32 – 2.18 (m, 1H), 2.02 – 1.92 (m, 1H), 1.33 (s, 9H), 1.07 (d, *J* = 6.7 Hz, 3H), 0.96 (d, *J* = 6.9 Hz, 3H).

^{13}C-NMR (126 MHz, $CDCl_3$): δ 177.8, 151.6, 136.4, 125.7, 125.4, 79.1, 47.5, 34.7, 33.3, 31.4, 27.6, 20.8, 18.3.

FT-IR (ATR, neat; cm^{-1}): 2959, 2904, 2870, 1768, 1465, 1362.

HRMS (ESI) *m/z*, $[M+H]^+$ calcd for $C_{14}H_{18}ClO_2{}^+$: 261.1849 found: 261.1849

5-(4-(*tert*-Butyl)phenyl)-3,3-dimethyldihydrofuran-2(3*H*)-one (41)

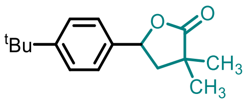

This compound was obtained according to general procedure **1** from 4-*tert*-butylstyrene (90.0 µL, 0.5 mmol), *fac*-[Ir(ppy)$_3$] (3.3 mg, 1.0 mol%), *2-bromo-2-methylpropionic acid* (167.0 mg, 1.0 mmol), and anhydrous DMF (1 mL). Isolated as a white crystalline solid (108.4 mg, 88% yield) after purification by flash column chromatography (12 g SiO_2, hexane/EA = 20:1).

^{1}H-NMR (300 MHz, $CDCl_3$): δ 7.46 – 7.36 (m, 2H), 7.31 – 7.25 (m, 2H), 5.43 (dd, *J* = 10.0, 6.2 Hz, 1H), 2.46 (dd, *J* = 12.8, 6.2 Hz, 1H), 2.09 (dd, *J* = 12.9, 10.0 Hz, 1H), 1.37 (s, 3H), 1.33 (s, 9H), 1.31 (s, 3H).

^{13}C-NMR (75 MHz, $CDCl_3$): δ 181.8, 151.5, 136.4, 125.7, 125.3, 77.7, 46.0, 40.9, 34.7, 31.4, 25.1, 24.3.

FT-IR (ATR, neat; cm^{-1}): 2964, 2868, 2361, 1768, 1461, 1326.

HRMS (NSI) *m/z*, $[M+H]^+$ calcd for $C_{16}H_{23}O_2^+$: 247.1693, 248.1727; found: 247.1691, 248.1724.

5-(4-(*tert*-Butyl)phenyl)-3-phenyldihydrofuran-2(3*H*)-one (42)

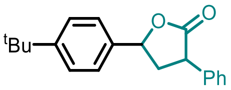

This compound was obtained according to general procedure **1** from 4-*tert*-butylstyrene (90.0 µL, 0.5 mmol), *fac*-[Ir(ppy)$_3$] (3.3 mg, 1.0 mol%), *chloro-phenyl-acetic acid* (170.6 mg, 1.0 mmol), and anhydrous DMF (1 mL). Collected 117.8 mg of diastereomeric mixture of white solids (1:1, 80% yield). After purification by flash column chromatography (24 g SiO_2, hexane/EA = 20:1).

diastereomer 1: **^{1}H-NMR** (300 MHz, $CDCl_3$): δ 7.50 – 7.15 (m, 9H), 5.68 (dd, *J* = 7.4, 5.4 Hz, 1H), 4.02 – 3.90 (m, 1H), 2.91 – 2.66 (m, 2H), 1.34 (s, 9H).

^{13}C-NMR (75 MHz, $CDCl_3$): δ 177.2, 151.6, 136.7, 136.3, 129.0, 127.8, 127.7, 125.8, 124.9, 78.8, 45.2, 39.2, 34.6, 31.3.

diastereomer 2: **^{1}H-NMR** (300 MHz, $CDCl_3$): δ 7.50 – 7.23 (m, 9H), 5.50 (dd, *J* = 10.8, 5.5 Hz, 1H), 4.04 (dd, *J* = 12.9, 8.4 Hz, 1H), 3.05 (ddd, *J* = 12.8, 8.4, 5.5 Hz, 1H), 2.42 (td, *J* = 12.8, 10.8 Hz, 1H), 1.34 (s, 9H).

^{13}C-NMR (75 MHz, $CDCl_3$): δ 176.6, 152.0, 136.3, 135.7, 129.0, 128.3, 127.9, 125.9, 125.6, 79.3, 47.8, 40.5, 34.8, 31.4.

FT-IR (ATR, neat; cm^{-1}): 2961, 2866, 1766, 1706, 1493, 1455, 1361.

HRMS (NSI) *m/z*, $[M+H]^+$ calcd for $C_{20}H_{23}O_2^+$: 295.1693, 296.1727; found: 295.1690, 296.1723.

7-(4-(*tert*-Butyl)phenyl)-6-oxaspiro[3.4]octan-5-one (43)

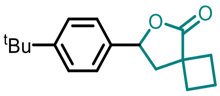

This compound was obtained according to general procedure **1** from 4-*tert*-butylstyrene (90.0 µL, 0.5 mmol), *fac*-[Ir(ppy)$_3$] (3.3 mg, 1.0 mol%), *1-bromocyclobutane-1-carboxylic acid* (179.0 mg, 1.0 mmol), and anhydrous DMF (1 mL). Isolated as a white crystalline solid (117.5 mg, 91% yield) after purification by flash column chromatography (12 g SiO_2, hexane/EA = 20:1).

^{1}H-NMR (300 MHz, $CDCl_3$): δ 7.44 – 7.31 (m, 2H), 7.28 – 7.20 (m, 2H), 5.32 (dd, *J* = 9.0, 6.2 Hz, 1H), 2.75 (dd, *J* = 12.9, 6.1 Hz, 1H), 2.67 – 2.44 (m, 2H), 2.33 – 1.87 (m, 6H), 1.31 (s, 9H).

^{13}C-NMR (75 MHz, $CDCl_3$): δ 180.9, 151.5, 136.3, 125.7, 125.3, 78.1, 44.9, 44.5, 34.7, 31.7, 31.4, 29.2, 16.6.

FT-IR (ATR, neat; cm^{-1}): 2959, 2867, 1753, 1689, 1606, 1460, 1300.

HRMS (ESI) *m/z*, $[M+H]^+$ calcd for $C_{17}H_{23}O_2^+$: 259.1693, 260.1727; found: 259.1690, 260.1723.

5-(4-(*tert*-Butyl)phenyl)-3-tetradecyldihydrofuran-2(3*H*)-one (44)

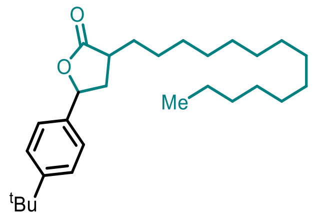

This compound was obtained according to general procedure **1** from 4-*tert*-butylstyrene (90.0 µL, 0.5 mmol), *fac*-[Ir(ppy)$_3$] (3.3 mg, 1.0 mol%), *2-bromopalmitic acid* (335 mg, 1.0 mmol), and anhydrous DMF (1 mL). Collected 180.4 mg of diastereomeric mixture of white solids (1:1, 87% yield). After purification by flash column chromatography (24 g SiO_2, hexane/EA = 20:1).

diastereomer 1: **^{1}H-NMR** (300 MHz, $CDCl_3$): δ 7.45 – 7.35 (m, 2H), 7.28 – 7.19 (m, 2H), 5.53 (dd, *J* = 7.2, 5.4 Hz, 1H), 2.73 – 2.58 (m, 1H), 2.47 – 2.31 (m, 2H), 1.96 – 1.83 (m, 1H), 1.61 – 1.35 (m, 4H), 1.32 (s, 9H), 1.26 (br s, 21H), 0.93 – 0.83 (m, 3H).

^{13}C-NMR (75 MHz, $CDCl_3$): δ 179.6, 151.4, 137.0, 125.8, 125.0, 78.8, 39.1, 36.5, 34.7, 32.1, 31.4, 30.7, 29.8, 29.8, 29.8, 29.8, 29.7, 29.6, 29.5, 29.5, 27.5, 22.8, 14.3.

diastereomer 2: **^{1}H-NMR** (300 MHz, $CDCl_3$): δ δ 7.49 – 7.35 (m, 2H), 7.33 – 7.23 (m, 2H), 5.34 (dd, *J* = 10.6, 5.2 Hz, 1H), 2.82 – 2.65 (m, 2H), 2.02 – 1.76 (m, 2H), 1.57 – 1.21 (m, 34H), 1.01 – 0.80 (m, 3H).

^{13}C-NMR (75 MHz, $CDCl_3$): d 178.9, 151.7, 136.3, 125.7, 125.4, 79.5, 41.6, 38.0, 34.7, 32.1, 31.4, 30.3, 29.8, 29.8, 29.8, 29.8, 29.7, 29.6, 29.5, 27.5, 22.8, 14.3.

FT-IR (ATR, neat; cm^{-1}): 2955, 2915, 2848, 1753, 1468, 1361, 1185, 1167.

HRMS (ESI) *m/z*, $[M+H]^+$ calcd for $C_{28}H_{47}O_2{}^+$: 415.3571, 416.3605, 417.3638; found: 415.3562, 416.3595, 417.3629.

5-(4-(*tert*-Butyl)phenyl)dihydrofuran-2(3*H*)-one (45)

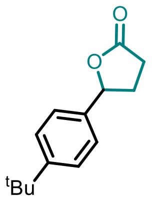

This compound was obtained according to general procedure **1** from 4-*tert*-butylstyrene (90.0 µL, 0.5 mmol), *fac*-[Ir(ppy)$_3$] (3.3 mg, 1.0 mol%), chloroacetic acid (94.5 mg, 1.0 mmol), and anhydrous DMF (1 mL). Isolated as a white crystalline solid (80.7 mg, 74% yield) after purification by flash column chromatography (12 g SiO_2, hexane/EA = 20:1).

^{1}H-NMR (300 MHz, $CDCl_3$): δ 7.47 – 7.34 (m, 2H), 7.33 – 7.22 (m, 2H), 5.50 (dd, *J* = 8.1, 6.2 Hz, 1H), 2.73 – 2.56 (m, 3H), 2.33 – 2.14 (m, 1H), 1.33 (s, 9H).

^{13}C-NMR (75 MHz, $CDCl_3$): δ 177.2, 151.8, 136.4, 125.8, 125.3, 81.4, 34.8, 31.4, 31.0, 29.2.

FT-IR (ATR, neat; cm^{-1}): 2955, 2866, 1759, 1615, 1509, 1458.

1-Acetyl-5-(4-(*tert*-butyl)phenyl)-3,3-difluoropyrrolidin-2-one (2)

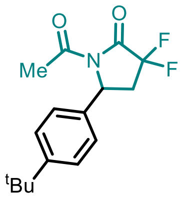

This compound was obtained according to general procedure **2** 4-*tert*-butylstyrene (90.0 μL, 0.5 mmol, 0.5 mmol), *fac*-[Ir(ppy)$_3$] (3.3 mg, 1.0 mol%), *CDFA (88* μL, 1.0 mmol), Boc anhydride (218.3 mg, 1.0 mmol) and anhydrous MeCN (3 mL). Isolated as a white crystalline solid (81.0 mg, 55% yield) after purification by flash column chromatography (12 g SiO_2, hexane/EA = 20:1). Spectroscopic data match with those reported in the literature.[13]

^{1}H-NMR (400 MHz, $CDCl_3$): δ 7.36 7.46 – 7.32 (m, 2H), 7.16 – 7.09 (m, 2H), 5.37 (dt, *J* = 9.3, 3.0 Hz, 1H), 2.90 (dddd, *J* = 19.6, 16.7, 15.1, 9.3 Hz, 1H), 2.59 (s, 3H), 2.57 – 2.48 (m, 1H), 1.30 (s, 9H).

^{13}C-NMR (101 MHz, $CDCl_3$): δ 170.2, 164.3 (dd, *J* = 31.5, 31.3 Hz), 151.5, 136.4, 126.1, 125.4 (d, *J* = 2.1 Hz), 117.4 (dd, *J* = 253.3, 251.8 Hz), 54.5 (dd, *J* = 2.1, 1.7 Hz), 37.5 (t, *J* = 21.4 Hz), 34.7, 31.4, 25.7.

^{19}F-NMR (376 MHz, $CDCl_3$): δ -99.7 (dt, *J* = 278.7, 20.0 Hz), -103.5 (ddd, *J* = 275.8, 16.9, 6.9 Hz).

1-Acetyl-5-(4-(*tert*-butyl)phenyl)-3-chloropyrrolidin-2-one (46)

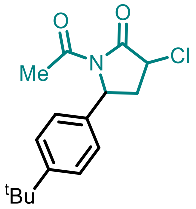

This compound was obtained according to general procedure **2** 4-*tert*-butylstyrene (90.0 μL, 0.5 mmol), *fac*-[Ir(ppy)$_3$] (3.3 mg, 1.0 mol%), *dichloroacetic acid* (83 μL, 1.0 mmol), Boc anhydride (218.3 mg, 1.0 mmol) and anhydrous MeCN (3 mL). Collected 64.4 mg of diastereomeric mixture (1:1, 44% yield). After purification by flash column chromatography (12 g SiO_2, hexane/EA = 20:1).We were able to isolate single diastereomer as a white crystalline solid.

^{1}H-NMR (400 MHz, $CDCl_3$): δ 7.38 – 7.30 (m, 2H), 7.13 – 7.01 (m, 2H), 5.43 (dd, *J* = 6.5, 4.1 Hz, 1H), 4.69 (dd, *J* = 9.7, 8.6 Hz, 1H), 2.66 – 2.61 (m, 2H), 2.61 (s, 3H), 1.29 (s, 9H).

^{13}C-NMR (101 MHz, $CDCl_3$): δ 171.2, 170.4, 151.2, 136.5, 126.2, 124.8, 77.4, 57.1, 54.5, 38.8, 31.4, 25.4.

FT-IR (ATR, neat; cm^{-1}): 2958, 2901, 2862, 2162, 1783, 1327, 1179.

1-Acetyl-5-(4-(*tert*-butyl)phenyl)-3,3-dichloropyrrolidin-2-one (47)

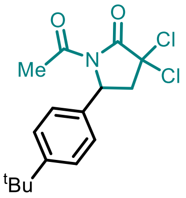

This compound was obtained according to general procedure **2** 4-*tert*-butylstyrene (90.0 μL, 0.5 mmol), *fac*-[Ir(ppy)$_3$] (3.3 mg, 1.0 mol%), *trichloroacetic acid* (164.0 mg, 1.0 mmol), Boc anhydride (218.3 mg, 1.0 mmol) and anhydrous MeCN (3 mL). Isolated as a white crystalline solid (85.3 mg, 52% yield) after purification by flash column chromatography (12 g SiO_2, hexane/EA = 20:1).

^{1}H-NMR (300 MHz, $CDCl_3$): δ 7.41 – 7.28 (m, 2H), 7.17 – 7.07 (m, 2H), 5.24 (t, *J* = 7.1 Hz, 1H), 3.33 (dd, *J* = 14.8, 7.4 Hz, 1H), 2.80 (dd, *J* = 14.8, 6.8 Hz, 1H), 2.59 (s, 3H), 1.30 (s, 9H).

^{13}C-NMR (75 MHz, $CDCl_3$): δ 170.7, 167.4, 151.3, 135.7, 126.0, 125.3, 80.6, 57.3, 49.6, 34.7, 31.4, 25.7.

FT-IR (ATR, neat; cm^{-1}): 2964, 1762, 1714, 1372, 1328, 1243, 1230.

HRMS (ESI) *m/z*, $[M+H]^+$ calcd for $C_{16}H_{20}O_2NCl_2^+$: 328.0866; found: 328.0861.

5-Methyl-5-(*p*-tolyl)dihydrofuran-2(3*H*)-one, (±)-boivinianin A (48)

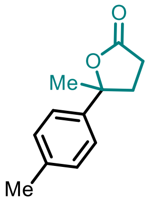

This compound was obtained according to general procedure **1** from 1-methyl-4-(prop-1-en-2-yl)benzene (73.0 μL, 0.5 mmol), *fac*-[Ir(ppy)$_3$] (3.3 mg, 1.0 mol%), *chloroacetic acid* (94.5 mg, 1.0 mmol), and anhydrous DMF (1 mL). Isolated as a white crystalline solid (70.4 mg, 74% yield) after purification by flash column chromatography (12 g SiO_2, hexane/EA = 20:1).[18]

^{1}H-NMR (300 MHz, $CDCl_3$): δ 7.30 – 7.23 (m, 2H), 7.21 – 7.14 (m, 2H), 2.69 – 2.36 (m, 4H), 2.35 (s, 3H), 1.71 (s, 3H).

^{13}C-NMR (75 MHz, $CDCl_3$): δ 176.8, 141.4, 137.5, 129.3, 124.2, 87.2, 36.3, 29.5, 29.1, 21.1.

HRMS (ESI) *m/z*, $[M+Na]^+$ calcd for $C_{12}H_{14}O_2+Na^+$: 213.0886, 214.0920; found: 213.0888, 214.0921.

3,3-Difluoro-5-methyl-5-(*p*-tolyl)dihydrofuran-2(3*H*)-one (49)

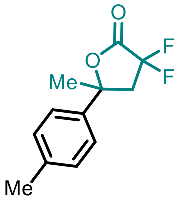

This compound was obtained according to general procedure **1** from 4-*tert*-butylstyrene (73.0 μL, 0.5 mmol), *fac*-[Ir(ppy)$_3$] (3.3 mg, 1.0 mol%), *CDFA (88* μL, 1.0 mmol), and anhydrous DMF (1 mL). Isolated as a white crystalline solid (92.0 mg, 81% yield) after purification by flash column chromatography (12 g SiO_2, hexane/EA = 20:1).

^{1}H-NMR (300 MHz, $CDCl_3$): δ 7.28 – 7.16 (m, 4H), 3.01 – 2.87 (m, 2H), 2.36 (s, 3H), 1.81 (s, 3H).

^{13}C-NMR (75 MHz, $CDCl_3$): δ 165.4 (dd, *J* = 32.9, 32.1 Hz), 139.7 (d, *J* = 1.4 Hz), 138.5, 129.7, 123.9, 116.2 (dd, *J* = 252.8, 252.8 Hz), 84.1 (dd, *J* = 3.7, 3.6 Hz), 45.1 (t, *J* = 20.5 Hz), 30.3, 21.1.

^{19}F-NMR (282 MHz, $CDCl_3$): δ -101.8 (d, *J* = 280.9 Hz), -104.8 (d, *J* = 280.9 Hz).

FT-IR (ATR, neat; cm^{-1}): 2957, 2923, 1810, 1725, 1514, 1493, 1031.

HRMS (EI) *m/z*, $[M]^+$ calcd for $C_{12}H_{12}F_2O_2^+$: 226.0805, 227.0839; found: 226.0803, 227.0837.

2-(Benzo[*d*][1,3]dioxol-5-yl)-3,4,5-trimethoxybenzaldehyde (50)

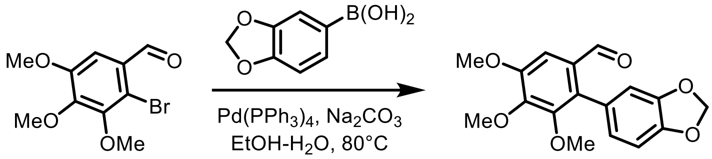

This compound was obtained according to following procedure:

A suspension of 2-bromo-3,4,5-trimethoxybenzaldehyde (0.86 g, 3.14 mmol), benzo[*d*][1,3]dioxol-5-ylboronic acid (0.52 g, 3.14 mmol), $Pd(PPh_3)_4$ (0.020 g, 0.017 mmol) and Na_2CO_3 in EtOH-H_2O 8:1 (v/v, 9 ml) was vigorously stirred for 8h at 80°C. Then reaction mixture was allowed cooling to the room temperature, extracted 3x15 ml EtOAc, organic phases were combined, dried with $MgSO_4$, filtered and evaporated to provide crude residue, which was purified further by flash column chromatography (12g SiO_2, hexane/EA = 9:1), **40** was isolated as a brownish solid (0.93 g, 94% yield).[19]

^{1}H-NMR (300 MHz, $CDCl_3$): δ 9.69 (s, 1H), 7.33 (s, 1H), 6.92 – 6.81 (m, 2H), 6.73 (dd, *J* = 7.9, 1.7 Hz, 1H), 6.04 (s, 2H), 3.99 (s, 3H), 3.95 (s, 3H), 3.64 (s, 3H).

^{13}C-NMR (75 MHz, $CDCl_3$): δ 191.4, 153.2, 151.4, 147.7, 147.6, 147.6, 134.1, 130.1, 126.4, 125.0, 111.4, 108.0, 105.3, 101.4, 61.3, 61.2, 56.3.

HRMS (ESI) *m/z*, $[M+H]^+$ calcd for $C_{17}H_{17}O_6^+$: 317.1020, 318.1054; found: 317.1015, 318.1049.

5-(2,3,4-Trimethoxy-6-(prop-1-en-1-yl)phenyl)benzo[*d*][1,3]dioxole (51)

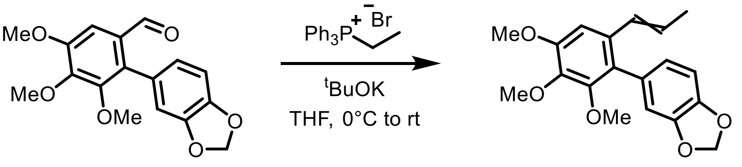

50 51

This compound was obtained according to following procedure:

To a suspension of **50** (1.080 g, 3.46 mmol), ethyltriphenylphosphonium bromide (1.7 g, 4.5 mmol) in THF (25 ml) was slowly added portionwise at 0°C ^{t}BuOK powder (0.5 g, 4.1 mmol) under N_2 atmosphere. The reaction mixture was allowed to warm at room temperature for 24h. The completion of the reaction was monitored by TLC, then 10 ml of saturated NH_4Cl solution was added. Reaction mixture was diluted by 40 ml of distilled H_2O and extracted 3x25 ml EtOAc. Combined organic phase was dried with $MgSO_4$, filtered, evaporated and purified by flash column chromatography (12g SiO_2, hexane/EA = 20:1), **51** was isolated as a yellowish oil (1.0 g, 89% yield). Crude NMR indicates the *E*/*Z* ratio 3:1.

^{1}H-NMR (300 MHz, $CDCl_3$): δ 6.90 – 6.78 (m, 1.67H), 6.76 – 6.62 (m, 3.66H), 6.17 – 5.99 (m, 2.66H), 5.98 (s, 2H), 5.60 (dq, *J* = 11.6, 7.1 Hz, 1H), 3.92 (s, 4H), 3.90 – 3.84 (m, 5H), 3.63 (s, 3H), 3.61 (s, 1H), 1.82 (dd, *J* = 7.1, 1.8 Hz, 3H), 1.75 (dd, *J* = 6.0, 1.1 Hz, 1H).

^{13}C-NMR (75 MHz, $CDCl_3$): δ 152.7, 151.9, 151.6, 147.3, 147.1, 146.5, 146.4, 141.4, 141.2, 132.7, 132.2, 130.4, 130.2, 129.6, 129.4, 128.4, 127.4, 126.0, 125.8, 124.2, 124.1, 111.3, 111.3, 108.8, 108.0, 107.9, 106.1, 104.1, 101.1, 101.0, 61.1, 61.1, 61.0, 56.2, 56.1, 56.1, 18.6, 14.7.

FT-IR (ATR, neat; cm^{-1}): 2933, 2832, 1589, 1559, 1504, 1478, 1452.

HRMS (ESI) *m/z*, $[M+H]^+$ calcd for $C_{19}H_{21}O_5^+$: 329.1384, 330.1418; found: 329.1375, 330.1408.

3,4-*epi*-Eupomatilone-6 and 4-*epi*-eupomatilone-6 (52)

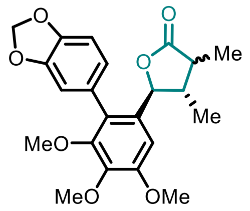

This compound was obtained according to general procedure **1** from **51** (164.2 mg, 0.5 mmol), *fac*-[Ir(ppy)$_3$] (3.3 mg, 1.0 mol%), *2-bromopropionic acid* (90 µL, 1.0 mmol), and anhydrous DMF (1 mL). Isolated as a white powder (174.2 mg, 87% yield) after purification by flash column chromatography (12 g SiO_2, hexane/EA = 4:1). NMR shows the inseparable d.r. ratio 1.1:1. Spectroscopic data match with those reported in the literature.[20]

^{1}H-NMR (400 MHz, $CDCl_3$): δ 6.91 – 6.81 (m, 2H), 6.74 – 6.68 (m, 4H), 6.67 – 6.60 (m, 2H), 6.05 – 5.97 (m, 4H), 5.06 (dd, *J* = 39.9, 4.4 Hz, 0.9H), 4.79 (t, *J* = 9.8 Hz, 1.1H), 3.95 – 3.84 (m, 12H), 3.66 – 3.57 (m, 6H), 2.81 – 2.68 (m, 0.95H), 2.45 – 2.31 (m, 0.95H), 2.21 – 2.10 (m, 1.05H), 2.09 – 1.98 (m, 1.05H), 1.24 (d, *J* = 6.8 Hz, 3.30H), 1.07 (dd, *J* = 7.5, 4.3 Hz, 2.70H), 0.87 (dd, *J* = 6.4, 3.7 Hz, 3.30H), 0.67 (dd, *J* = 9.9, 7.2 Hz, 2.70H).

^{13}C-NMR (101 MHz, $CDCl_3$): δ 180.1 (d, *J* = 1.2 Hz), 178.8 (d, *J* = 7.9 Hz), 153.4 (d, *J* = 1.7 Hz), 153.2 (d, *J* = 3.3 Hz), 151.9 (d, *J* = 3.7 Hz), 151.4 (d, *J* = 5.0 Hz), 147.8 (d, *J* = 2.5 Hz), 147.5 (d, *J* = 11.2 Hz), 147.0 (d, *J* = 5.8 Hz), 142.7 (d, *J* = 2.1 Hz), 142.1 (d, *J* = 3.7 Hz), 132.9 (d, *J* = 5.8 Hz), 130.8 (d, *J* = 10.8 Hz), 130.0, 129.1 (d, *J* = 2.9 Hz), 129.0, 127.6 (d, *J* = 9.5 Hz), 124.4, 124.2, 123.5, 123.0, 111.5, 111.4, 110.7, 110.3, 108.5, 108.3 (d, *J* = 3.3 Hz), 108.0, 105.3 (d, *J* = 4.1 Hz), 103.8, 101.3 (t, *J* = 4.6 Hz), 83.0, 82.8 (d, *J* = 10.0 Hz), 61.2 (d, *J* = 10.0 Hz), 61.2 (d, *J* = 9.1 Hz), 56.3 (d, *J* = 2.9 Hz), 47.7 (d), 43.5 (d, *J* = 2.5 Hz), 41.7 (d, *J* = 10.0 Hz), 37.2, 14.4 (d, *J* = 2.5 Hz), 13.5 (d, *J* = 2.5 Hz), 13.0 (d), 9.9 (d, *J* = 2.1 Hz).

HRMS (ESI) *m/z*, $[M+H]^+$ calcd for $C_{22}H_{25}O_7^+$: 401.1595, 402.1629; found: 401.1588, 402.1622.

FT-IR (ATR, neat; cm^{-1}): 2938, 1774, 1595, 1503, 1482, 1455, 1436.

^{1}H NMR correlation with reported data:

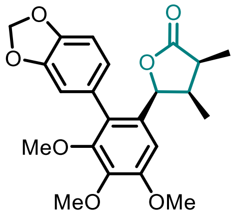

eupomatilone-6 (**1**)

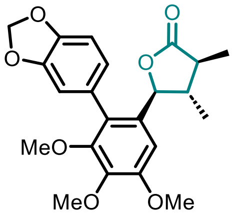

3,4-*epi*-eupomatilone-6

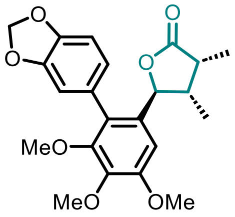

4-*epi*-eupomatilone-6

	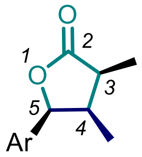	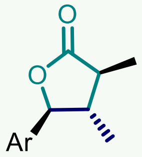	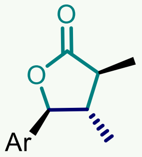	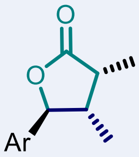	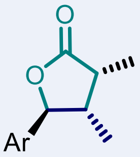
	**eupomatilone-6 (1)**	**3,4 *epi*-1**	**3,4 *epi*-1**	**4 *epi*-1**	**4 *epi*-1**
**H**	Taylor[21]	Hall[20] (300 MHz)	This work (400 MHz)	Hall[20] (300 MHz)	This work (400 MHz)
3-Me	δ 1.20, 1.19, d	δ 1.23, d *J*= 6.9 Hz	δ 1.24, d *J*= 6.8 Hz	δ 1.07, d *J*= 7.3 Hz δ 1.08, d *J*= 7.3 Hz	δ 1.07, dd *J*= 7.5, 4.3 Hz
4-Me	δ 0.70, 0.73, d	δ 0.86, 0.87, d *J*= 6.4 Hz	δ 0.87, dd *J*= 6.4, 3.7 Hz	δ 0.66, d *J*= 7.1 Hz 0.69, d *J*= 7.1 Hz	δ 0.67, dd *J* = 9.9, 7.2 Hz
H-3	δ 2.36, 2.37, m	δ 2.14, m	δ 2.16, m	δ 2.75, m	δ 2.75, m
H-4	δ 2.36, 1.97, m	δ 2.03, m	δ 2.03, m	δ 2.39, m	δ 2.38, m
H-5	δ 5.54, 5.65, d	δ 4.78, 4.80, d *J*= 9.8 Hz	δ 4.79, t *J*= 9.8 Hz	δ 5.01, d *J*= 4.5 Hz 5.11, d *J*= 4.5 Hz	δ 5.01, d *J*= 4.4 Hz δ 5.11, d *J*= 4.4 Hz

Table S14: ^{1}H NMR correlation with reported data for Eupomatilone-6.

Ar =

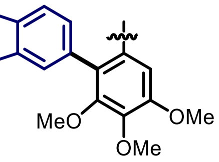

11. X-Ray Data

X-RAY CRYSTAL STRUCTURE REPORT

Sample No.: 23DK001 (11)

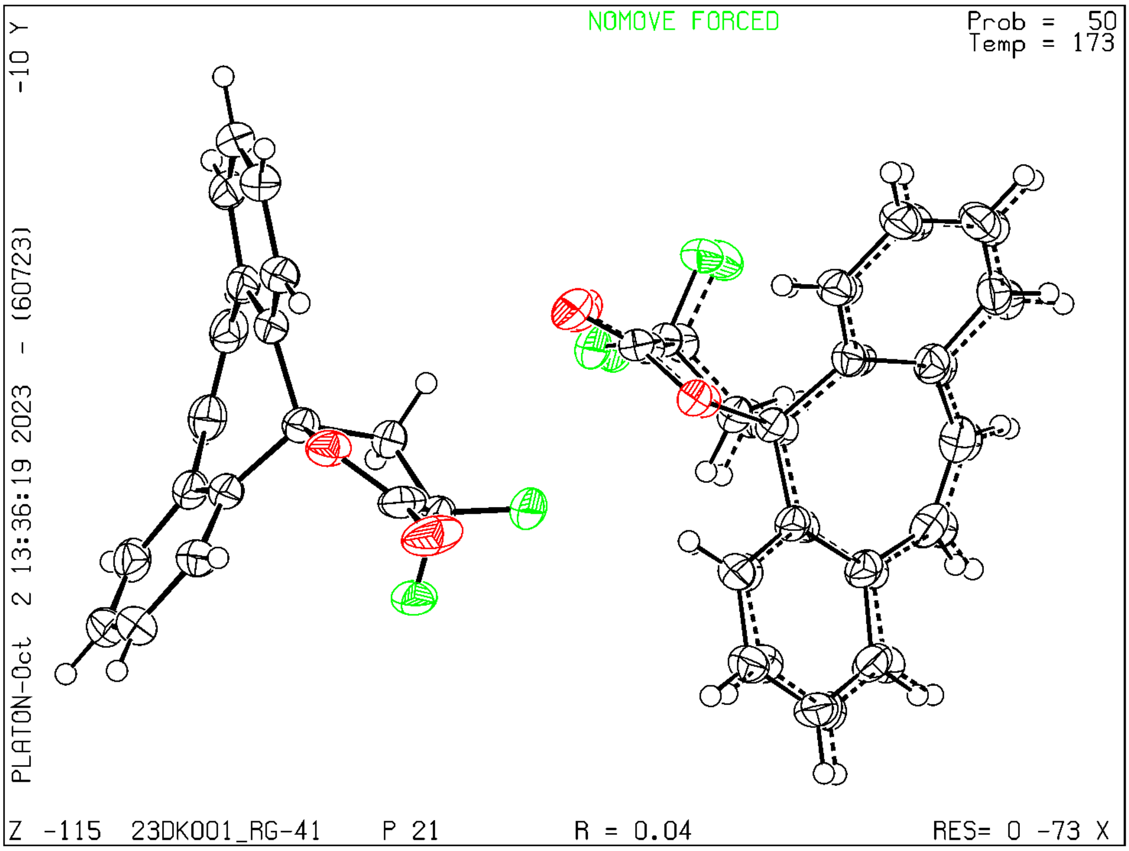

EXPERIMENTAL

Crystal-Structure Determination. A crystal of $C_{18}H_{12}F_2O_2$ was dissolved using acetone under the microscope, then immersed in parabar oil, mounted at ambient conditions and transferred into the stream of nitrogen (173 K). All measurements were made on a *RIGAKU Synergy S* area-detector diffractometer[22] using mirror optics monochromated Cu *K*α radiation (λ = 1.54184 Å). The unit cell constants and an orientation matrix for data collection were obtained from a least-squares refinement of the setting angles of reflections in the range 3.771° < θ < 80.116°. A total of 5736 frames were collected using ω scans, with 1.5 seconds exposure time (5 s for high-angle reflections), a rotation angle of 0.5° per frame, a crystal-detector distance of 31.0 mm, at T = 173.01(10) K.

Data reduction was performed using the *CrysAlisPro* program. The intensities were corrected for Lorentz and polarization effects, and a numerical absorption correction based on gaussian integration over a multifaceted crystal model with additional empirical absorption correction using spherical harmonics using SCALE3 ABSPACK in *CrysAlisPro* was applied. Data collection and refinement parameters are given in **Table S15**.

The structure was solved by intrinsic phasing using *SHELXT*[23], which revealed the positions of all non-hydrogen atoms of the title compound. All non-hydrogen atoms were refined anisotropically. H-atoms were assigned in geometrically calculated positions and refined using a riding model where each H-atom was assigned a fixed isotropic displacement parameter with a value equal to 1.2Ueq of its parent atom (1.5Ueq for methyl groups).

Refinement of the structure was carried out on F^2 using full-matrix least-squares procedures, which minimized the function $\Sigma w(F_o^2 - F_c^2)^2$. The weighting scheme was based on counting statistics and included a factor to downweight the intense reflections. All calculations were performed using the *SHELXL-2014/7* [24] program in OLEX2.[25]

Signs of a super structure could be detected during the data reduction, the structure is refined using the super cell, and therefore contains two independent molecules in the asymmetric unit. In addition, one of the molecules is fully disorder. A disorder model was used for this one molecule where the occupancies of each disorder component was refined through the use of a free variable. The sum of equivalent components was constrained to 1, i.e. 100%. The SAME restraints were used in the refinement to reduce the number of parameters on the refinement. Since checkcif is unable to detect this restrain, an incorrect alert is raised regarding the ratio of total number of reflections/parameter.

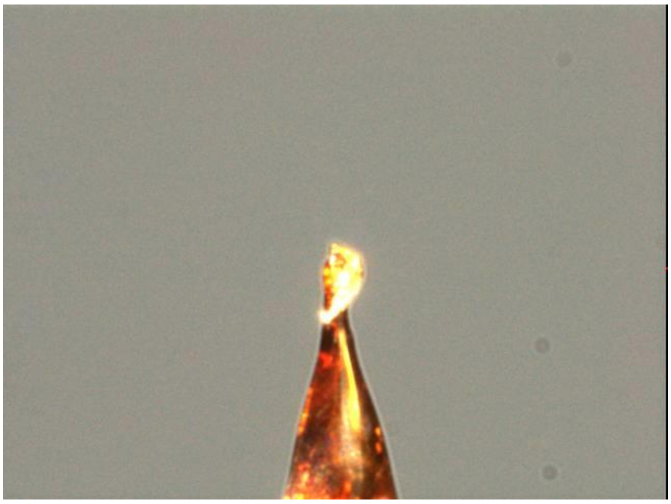

Figure S19: A single crystal of the compound mounted on a loop.

Table S15 Crystal data and structure refinement for 23DK001_*11*.

Identification code	23DK001_*10*
Empirical formula	$C_{18}H_{12}F_2O_2$
Formula weight	298.28
Temperature/K	173.01(10)
Crystal system	monoclinic
Space group	$P2_1$
a/Å	10.05628(17)
b/Å	5.83820(12)
c/Å	23.8712(4)
α/°	90
β/°	100.5648(17)
γ/°	90
Volume/Å^3	1377.74(4)
Z	4
ρ_{calc}g/cm^3	1.438
μ/mm^{-1}	0.938
F(000)	616.0
Crystal size/mm^3	0.118 × 0.084 × 0.056
Radiation	Cu Kα (λ = 1.54184)
2Θ range for data collection/°	7.534 to 161.372
Index ranges	$-12 \leq h \leq 12$, $-6 \leq k \leq 7$, $-30 \leq l \leq 30$
Reflections collected	28713
Independent reflections	5747 [R_{int} = 0.0277, R_{sigma} = 0.0221]
Data/restraints/parameters	5747/497/597
Goodness-of-fit on F^2	1.065
Final R indexes [I>=2σ (I)]	R_1 = 0.0386, wR_2 = 0.1097
Final R indexes [all data]	R_1 = 0.0412, wR_2 = 0.1114
Largest diff. peak/hole / e Å^{-3}	0.20/-0.17
Flack parameter	-0.01(11)

Fractional Atomic Coordinates (×10^4) and Equivalent Isotropic Displacement Parameters (Å^2×10^3) for 23DK001_*11*. U_{eq} is defined as 1/3 of of the trace of the orthogonalised U_{IJ} tensor.

Atom	*x*	*y*	*z*	U(eq)
C2B	9912(2)	8005(5)	1408.0(9)	32.2(5)
C3B	10471(2)	9840(6)	1741.1(10)	37.4(6)
C4B	11865(2)	10074(6)	1902.0(11)	42.4(6)
C5B	12715(2)	8423(7)	1749.1(11)	44.9(7)
C6B	12169(2)	6567(6)	1423.9(10)	41.1(6)
C7B	10766(2)	6339(5)	1235.5(10)	34.3(5)
C8B	10312(2)	4375(6)	871.6(11)	39.6(6)
C9B	9184(3)	4111(5)	481.5(10)	38.3(6)
C10B	8101(2)	5773(5)	302.3(10)	34.3(6)
C11B	7368(3)	5575(6)	-254.2(10)	39.9(6)
C12B	6405(3)	7167(6)	-482.3(10)	43.3(7)
C13B	6146(3)	9005(6)	-156.0(11)	42.1(6)
C14B	6827(2)	9209(6)	405.1(10)	36.7(6)
C15B	7780(2)	7579(5)	645.0(9)	32.0(5)
C1B	8386(2)	7658(5)	1280.8(9)	31.5(5)
C16B	7945(2)	5574(6)	1608.1(10)	36.4(6)
C17B	6617(2)	6368(6)	1742.3(10)	40.2(6)
C18B	6725(2)	8976(7)	1760.7(11)	43.3(6)
F1B	6326.4(15)	5556(6)	2235.9(6)	57.4(6)
F2B	5544.0(14)	5786(5)	1326.3(6)	47.3(5)
O1B	7779.5(16)	9617(4)	1524.7(7)	38.0(5)
O2B	6008.5(18)	10299(6)	1945.2(10)	61.4(7)
C1	6609(3)	3246(6)	3740.9(12)	28.5(7)
C2	5077(3)	3536(7)	3608.1(16)	30.6(8)
C3	4496(3)	5350(8)	3276.7(17)	36.3(9)
C4	3104(4)	5512(9)	3101(2)	41.2(11)

Fractional Atomic Coordinates (×10^4) and Equivalent Isotropic Displacement Parameters (Å^2×10^3) for 23DK001_*11*. U_{eq} is defined as 1/3 of of the trace of the orthogonalised U_{IJ} tensor.

Atom	*x*	*y*	*z*	U(eq)
C5	2275(4)	3815(9)	3245(3)	43.1(12)
C6	2836(4)	1977(9)	3572(2)	40.4(12)
C7	4238(4)	1822(7)	3774.5(18)	34.3(10)
C8	4711(4)	-125(8)	4136(2)	41.0(11)
C9	5839(4)	-371(8)	4529(2)	39.4(10)
C10	6912(4)	1292(7)	4713.0(15)	33.7(8)
C11	7666(5)	1073(8)	5266.3(16)	40.3(11)
C12	8622(5)	2664(9)	5498.2(17)	42.8(11)
C13	8865(4)	4528(9)	5175.8(16)	41.9(10)
C14	8172(4)	4753(7)	4620.6(15)	35.3(9)
C15	7222(4)	3138(7)	4375.1(12)	31.5(8)
C16	7076(3)	1228(7)	3398.7(13)	32.4(8)
C17	8391(3)	2105(8)	3264.7(11)	31.9(8)
C18	8251(3)	4704(7)	3270.4(12)	33.2(8)
F1	8664(2)	1398(5)	2759.1(8)	42.3(8)
F2	9479.1(19)	1496(5)	3668.6(9)	42.4(8)
O1	7187(2)	5270(5)	3509.0(11)	32.2(6)
O2	8951(2)	6092(6)	3094.2(12)	45.4(8)
C1A	6605(16)	2320(40)	3712(7)	31(3)
C2A	5080(16)	2670(40)	3593(9)	27(3)
C3A	4520(20)	4440(40)	3250(11)	36(5)
C4A	3110(20)	4700(50)	3120(15)	39(5)
C5A	2260(30)	3040(40)	3259(15)	31(4)
C6A	2820(20)	1190(40)	3587(15)	35(5)
C7A	4244(19)	1000(30)	3756(9)	25(4)
C8A	4680(20)	-860(40)	4142(12)	31(4)
C9A	5830(20)	-1200(40)	4519(10)	28(4)

Fractional Atomic Coordinates (×10^4) and Equivalent Isotropic Displacement Parameters (Å^2×10^3) for 23DK001_*11*. U_{eq} is defined as 1/3 of of the trace of the orthogonalised U_{IJ} tensor.

Atom	*x*	*y*	*z*	U(eq)
C10A	6890(20)	430(40)	4689(8)	28(4)
C11A	7580(30)	240(40)	5256(9)	35(5)
C12A	8570(30)	1760(40)	5473(10)	34(5)
C13A	8860(30)	3610(40)	5164(10)	37(5)
C14A	8180(20)	3860(40)	4588(8)	29(4)
C15A	7218(19)	2220(40)	4360(7)	26(3)
C16A	7036(17)	170(40)	3396(7)	32(3)
C17A	8366(16)	930(50)	3258(7)	37(3)
C18A	8267(16)	3540(50)	3229(8)	37(3)
F1A	8641(13)	80(50)	2767(5)	47(4)
F2A	9427(13)	310(40)	3675(6)	47(4)
O1A	7206(14)	4270(40)	3469(7)	36(3)
O2A	8991(15)	4860(50)	3043(8)	52(4)

Anisotropic Displacement Parameters (Å^2×10^3) for 23DK001_*10*. The Anisotropic displacement factor exponent takes the form: $-2\pi^2[h^2a^{*2}U_{11}+2hka^*b^*U_{12}+\ldots]$.

Atom	U_{11}	U_{22}	U_{33}	U_{23}	U_{13}	U_{12}
C2B	27.2(10)	42.0(15)	27.9(10)	4.2(9)	6.5(8)	1.7(9)
C3B	31.4(11)	49.1(16)	30.8(11)	-0.4(10)	3.5(9)	-0.2(10)
C4B	33.6(12)	56.8(18)	35.5(12)	2.7(11)	2.9(9)	-7.4(11)
C5B	26.5(11)	71(2)	37.0(12)	10.4(13)	5.1(9)	-2.6(11)
C6B	30.0(12)	58.1(18)	37.2(12)	9.5(12)	11.4(9)	8.1(11)
C7B	29.7(11)	44.5(15)	30.5(11)	8.5(10)	10.4(9)	6.4(9)
C8B	37.9(12)	43.5(15)	41.4(12)	4.0(11)	17.9(10)	9.5(10)
C9B	43.1(13)	38.5(15)	36.5(12)	-2.2(10)	15.3(10)	2.5(10)
C10B	32.4(11)	41.2(15)	30.9(11)	-1.8(9)	10.3(9)	-2.3(9)
C11B	42.7(13)	48.0(16)	31.0(11)	-4.9(10)	12.0(10)	-7.2(11)

Anisotropic Displacement Parameters (Å²×10³) for 23DK001_*10*. The Anisotropic displacement factor exponent takes the form: $-2\pi^2[h^2a^{*2}U_{11}+2hka^*b^*U_{12}+\ldots]$.

Atom	U_{11}	U_{22}	U_{33}	U_{23}	U_{13}	U_{12}
C12B	38.8(13)	61.4(19)	28.7(11)	0.1(11)	3.3(9)	-12.3(12)
C13B	36.6(12)	53.8(17)	34.0(12)	5.8(11)	1.2(10)	0.6(11)
C14B	31.3(11)	42.7(15)	35.1(11)	0.3(10)	3.6(9)	1.7(10)
C15B	27.6(10)	40.9(14)	28.1(10)	-0.8(9)	6.4(8)	-0.9(9)
C1B	27.2(10)	38.8(13)	29.2(10)	-4.3(8)	7.0(8)	3.0(8)
C16B	30.2(10)	51.8(15)	28.6(10)	0.7(10)	9.2(8)	0.1(10)
C17B	25.2(10)	70.3(16)	25.4(10)	-6.5(10)	5.7(8)	-6.1(10)
C18B	22.9(10)	70.2(16)	35.7(11)	-17.9(11)	2.4(8)	0.9(10)
F1B	36.2(8)	108.5(16)	30.4(7)	1.5(8)	13.4(6)	-7.7(9)
F2B	30.0(7)	72.9(13)	36.8(7)	-9.9(7)	-0.1(6)	-10.4(7)
O1B	28.3(8)	49.3(12)	35.9(8)	-12.0(7)	4.9(6)	4.9(7)
O2B	30.3(9)	88.5(18)	66.6(13)	-34.9(13)	11.7(9)	4.8(10)
C1	25.6(12)	34(2)	26.9(12)	0.5(13)	7.3(10)	-3.8(12)
C2	26.6(12)	39(2)	27.2(14)	-4.9(16)	8.4(10)	-0.8(13)
C3	31.0(14)	49(3)	28.2(14)	2.1(18)	4.0(10)	1.2(16)
C4	32.5(15)	58(3)	32.5(17)	3(2)	3.5(12)	8.4(17)
C5	25.2(14)	67(4)	37.2(19)	-6(3)	4.6(12)	1.3(19)
C6	29.4(14)	56(3)	38(2)	-6(2)	12.5(13)	-8.1(17)
C7	29.4(14)	45(3)	30.8(15)	-8.7(18)	11.2(11)	-3.8(15)
C8	38.5(17)	44(3)	45(2)	-3(2)	19.1(13)	-9.3(18)
C9	44.1(18)	38(3)	39.9(17)	5.0(18)	17.5(13)	1.2(17)
C10	32.7(15)	38(2)	32.8(15)	0.7(15)	11.7(11)	3.4(16)
C11	42(2)	52(3)	29.5(14)	7.7(17)	11.9(13)	8(2)
C12	39.1(18)	60(3)	27.4(15)	-1.3(19)	2.2(12)	13(2)
C13	35.7(17)	58(3)	30.5(14)	-4.7(17)	1.6(11)	1(2)
C14	31.3(15)	42(3)	31.3(14)	-3.5(15)	3.3(11)	-0.3(16)
C15	29.1(15)	40(2)	25.6(12)	1.3(13)	7.3(10)	3.0(15)

Anisotropic Displacement Parameters (Å^2×10^3) for 23DK001_*10*. The Anisotropic displacement factor exponent takes the form: -2π^2[h^{2}a*^{2}U$_{11}$+2hka*b*U$_{12}$+…].

Atom	U_{11}	U_{22}	U_{33}	U_{23}	U_{13}	U_{12}
C16	28.0(13)	41(2)	29.6(14)	-0.8(14)	8.9(10)	-1.7(13)
C17	24.7(12)	48(2)	23.4(12)	1.7(14)	5.3(9)	0.9(13)
C18	22.9(13)	48(2)	28.1(13)	3.4(13)	2.8(10)	-4.1(13)
F1	36.8(10)	62(2)	31.8(9)	-1.6(10)	15.7(7)	-0.2(10)
F2	26.7(9)	61(2)	37.6(9)	7.0(11)	0.6(7)	5.8(10)
O1	28.4(10)	38.1(17)	30.9(10)	4.0(11)	6.8(8)	-4.2(10)
O2	32.9(12)	54(2)	50.6(13)	8.1(14)	12.0(9)	-10.9(12)
C1A	32(4)	33(6)	28(4)	-1(4)	3(3)	-2(4)
C2A	32(4)	27(6)	21(6)	-5(5)	1(3)	-3(4)
C3A	33(5)	37(8)	36(8)	7(7)	6(5)	0(5)
C4A	33(5)	40(9)	44(10)	8(8)	5(5)	2(5)
C5A	33(5)	36(8)	26(8)	-3(6)	9(5)	2(5)
C6A	33(4)	36(9)	33(8)	0(7)	4(5)	-2(5)
C7A	33(4)	23(7)	19(6)	-9(5)	5(4)	-3(4)
C8A	33(5)	32(8)	29(6)	0(6)	9(4)	-1(5)
C9A	31(5)	30(7)	26(6)	2(6)	12(4)	0(5)
C10A	30(6)	30(7)	27(4)	-2(4)	11(4)	2(5)
C11A	36(7)	39(9)	30(5)	2(5)	7(4)	-3(7)
C12A	32(7)	36(8)	33(6)	-2(5)	5(5)	2(6)
C13A	37(8)	38(8)	34(5)	-3(6)	-1(4)	-6(7)
C14A	25(6)	31(7)	32(5)	-3(5)	4(4)	0(6)
C15A	21(5)	30(6)	28(4)	-2(4)	6(3)	1(5)
C16A	35(4)	39(6)	23(5)	0(5)	7(4)	1(4)
C17A	33(4)	48(6)	30(4)	6(4)	7(3)	3(4)
C18A	25(5)	48(6)	35(7)	8(5)	0(4)	-1(4)
F1A	41(5)	69(9)	34(4)	2(5)	12(3)	8(6)
F2A	40(4)	55(9)	42(4)	6(6)	-1(4)	8(5)

Anisotropic Displacement Parameters (Å^2×10^3) for 23DK001_*10*. The Anisotropic displacement factor exponent takes the form: $-2\pi^2[h^2a^{*2}U_{11}+2hka^*b^*U_{12}+\dots]$.

Atom	U_{11}	U_{22}	U_{33}	U_{23}	U_{13}	U_{12}
O1A	29(4)	40(6)	36(5)	8(5)	2(4)	0(4)
O2A	36(6)	56(8)	65(9)	18(8)	12(6)	-3(6)

Bond Lengths for 23DK001_*11*.

Atom	Atom	Length/Å	Atom	Atom	Length/Å
C2B	C3B	1.390(4)	C10	C11	1.403(4)
C2B	C7B	1.408(3)	C10	C15	1.415(4)
C2B	C1B	1.522(3)	C11	C12	1.378(6)
C3B	C4B	1.390(3)	C12	C13	1.380(6)
C4B	C5B	1.381(4)	C13	C14	1.386(4)
C5B	C6B	1.386(4)	C14	C15	1.393(5)
C6B	C7B	1.407(3)	C16	C17	1.506(4)
C7B	C8B	1.460(4)	C17	C18	1.524(5)
C8B	C9B	1.337(4)	C17	F1	1.351(3)
C9B	C10B	1.464(3)	C17	F2	1.367(3)
C10B	C11B	1.401(3)	C18	O1	1.342(3)
C10B	C15B	1.408(3)	C18	O2	1.199(4)
C11B	C12B	1.380(4)	C1A	C2A	1.521(18)
C12B	C13B	1.379(4)	C1A	C15A	1.557(18)
C13B	C14B	1.393(3)	C1A	C16A	1.57(2)
C14B	C15B	1.397(3)	C1A	O1A	1.456(18)
C15B	C1B	1.529(3)	C2A	C3A	1.38(2)
C1B	C16B	1.554(4)	C2A	C7A	1.386(19)
C1B	O1B	1.466(3)	C3A	C4A	1.40(2)
C16B	C17B	1.502(3)	C4A	C5A	1.37(2)
C17B	C18B	1.526(5)	C5A	C6A	1.40(2)

Bond Lengths for 23DK001_*11*.

Atom	Atom	Length/Å	Atom	Atom	Length/Å
C17B	F1B	1.351(3)	C6A	C7A	1.41(2)
C17B	F2B	1.369(3)	C7A	C8A	1.44(2)
C18B	O1B	1.342(3)	C8A	C9A	1.340(19)
C18B	O2B	1.194(3)	C9A	C10A	1.44(2)
C1	C2	1.524(4)	C10A	C11A	1.406(19)
C1	C15	1.528(4)	C10A	C15A	1.384(19)
C1	C16	1.555(4)	C11A	C12A	1.37(2)
C1	O1	1.470(3)	C12A	C13A	1.37(2)
C2	C3	1.385(5)	C13A	C14A	1.43(2)
C2	C7	1.412(4)	C14A	C15A	1.396(19)
C3	C4	1.389(4)	C16A	C17A	1.503(18)
C4	C5	1.378(6)	C17A	C18A	1.52(2)
C5	C6	1.385(6)	C17A	F1A	1.348(17)
C6	C7	1.407(4)	C17A	F2A	1.370(17)
C7	C8	1.453(5)	C18A	O1A	1.369(18)
C8	C9	1.341(5)	C18A	O2A	1.200(19)
C9	C10	1.458(5)			

Bond Angles for 23DK001_*11*.

Atom	Atom	Atom	Angle/°	Atom	Atom	Atom	Angle/°
C3B	C2B	C7B	119.7(2)	C15	C10	C9	124.3(3)
C3B	C2B	C1B	120.2(2)	C12	C11	C10	122.2(4)
C7B	C2B	C1B	119.8(2)	C11	C12	C13	119.3(3)
C4B	C3B	C2B	121.0(2)	C12	C13	C14	119.9(4)
C5B	C4B	C3B	120.0(3)	C13	C14	C15	121.6(3)
C4B	C5B	C6B	119.5(2)	C10	C15	C1	120.3(3)
C5B	C6B	C7B	121.6(2)	C14	C15	C1	120.7(3)

Bond Angles for 23DK001_*11*.

Atom	Atom	Atom	Angle/°	Atom	Atom	Atom	Angle/°
C2B	C7B	C8B	125.1(2)	C14	C15	C10	118.8(3)
C6B	C7B	C2B	118.1(2)	C17	C16	C1	102.4(3)
C6B	C7B	C8B	116.8(2)	C16	C17	C18	104.5(3)
C9B	C8B	C7B	129.6(2)	F1	C17	C16	114.6(3)
C8B	C9B	C10B	128.1(2)	F1	C17	C18	110.3(3)
C11B	C10B	C9B	117.1(2)	F1	C17	F2	106.4(2)
C11B	C10B	C15B	118.5(2)	F2	C17	C16	112.5(3)
C15B	C10B	C9B	124.4(2)	F2	C17	C18	108.4(3)
C12B	C11B	C10B	121.8(2)	O1	C18	C17	109.3(3)
C13B	C12B	C11B	119.5(2)	O2	C18	C17	127.5(3)
C12B	C13B	C14B	119.9(2)	O2	C18	O1	123.2(4)
C13B	C14B	C15B	121.0(2)	C18	O1	C1	111.3(3)
C10B	C15B	C1B	120.5(2)	C2A	C1A	C15A	113.1(14)
C14B	C15B	C10B	119.0(2)	C2A	C1A	C16A	112.0(13)
C14B	C15B	C1B	120.3(2)	C15A	C1A	C16A	110.7(13)
C2B	C1B	C15B	113.93(18)	O1A	C1A	C2A	107.5(14)
C2B	C1B	C16B	111.96(19)	O1A	C1A	C15A	107.9(13)
C15B	C1B	C16B	112.1(2)	O1A	C1A	C16A	105.2(12)
O1B	C1B	C2B	107.14(19)	C3A	C2A	C1A	120.0(16)
O1B	C1B	C15B	107.39(18)	C3A	C2A	C7A	119.4(15)
O1B	C1B	C16B	103.48(17)	C7A	C2A	C1A	119.9(15)
C17B	C16B	C1B	102.3(2)	C2A	C3A	C4A	120.1(18)
C16B	C17B	C18B	104.7(2)	C5A	C4A	C3A	121(2)
F1B	C17B	C16B	114.9(2)	C4A	C5A	C6A	119(2)
F1B	C17B	C18B	110.5(2)	C5A	C6A	C7A	120(2)
F1B	C17B	F2B	106.1(2)	C2A	C7A	C6A	120.0(16)
F2B	C17B	C16B	112.5(2)	C2A	C7A	C8A	125.4(17)
F2B	C17B	C18B	108.1(2)	C6A	C7A	C8A	114.3(18)

Bond Angles for 23DK001_*11*.

Atom	Atom	Atom	Angle/°	Atom	Atom	Atom	Angle/°
O1B	C18B	C17B	108.8(2)	C9A	C8A	C7A	132(2)
O2B	C18B	C17B	127.7(3)	C8A	C9A	C10A	126.9(19)
O2B	C18B	O1B	123.5(3)	C11A	C10A	C9A	115.5(17)
C18B	O1B	C1B	111.6(2)	C15A	C10A	C9A	125.5(16)
C2	C1	C15	114.8(2)	C15A	C10A	C11A	118.8(16)
C2	C1	C16	111.5(2)	C12A	C11A	C10A	120.6(19)
C15	C1	C16	112.6(3)	C11A	C12A	C13A	121.4(19)
O1	C1	C2	106.7(2)	C12A	C13A	C14A	119.2(18)
O1	C1	C15	106.9(2)	C15A	C14A	C13A	118.8(17)
O1	C1	C16	103.5(2)	C10A	C15A	C1A	120.1(14)
C3	C2	C1	120.5(3)	C10A	C15A	C14A	120.9(15)
C3	C2	C7	119.4(3)	C14A	C15A	C1A	118.8(15)
C7	C2	C1	119.7(3)	C17A	C16A	C1A	101.7(13)
C2	C3	C4	121.3(3)	C16A	C17A	C18A	104.7(13)
C5	C4	C3	119.9(4)	F1A	C17A	C16A	114.3(15)
C4	C5	C6	119.7(3)	F1A	C17A	C18A	110.8(15)
C5	C6	C7	121.5(3)	F1A	C17A	F2A	106.0(13)
C2	C7	C8	125.0(3)	F2A	C17A	C16A	111.5(14)
C6	C7	C2	118.1(3)	F2A	C17A	C18A	109.6(15)
C6	C7	C8	116.9(3)	O1A	C18A	C17A	109.9(13)
C9	C8	C7	129.9(4)	O2A	C18A	C17A	128.2(18)
C8	C9	C10	128.6(4)	O2A	C18A	O1A	122(2)
C11	C10	C9	117.7(3)	C18A	O1A	C1A	109.6(14)
C11	C10	C15	117.9(3)				

Torsion Angles for 23DK001_*11*.

A	B	C	D	Angle/˚	A	B	C	D	Angle/˚
C2B	C3B	C4B	C5B	-2.5(4)	C11	C10	C15	C14	-5.4(5)
C2B	C7B	C8B	C9B	25.7(4)	C11	C12	C13	C14	-1.5(7)
C2B	C1B	C16B	C17B	144.72(19)	C12	C13	C14	C15	0.2(6)
C2B	C1B	O1B	C18B	-140.96(19)	C13	C14	C15	C1	-171.8(4)
C3B	C2B	C7B	C6B	1.8(3)	C13	C14	C15	C10	3.4(6)
C3B	C2B	C7B	C8B	-177.9(2)	C15	C1	C2	C3	123.8(4)
C3B	C2B	C1B	C15B	123.2(2)	C15	C1	C2	C7	-62.6(4)
C3B	C2B	C1B	C16B	-108.2(3)	C15	C1	C16	C17	-85.6(3)
C3B	C2B	C1B	O1B	4.6(3)	C15	C1	O1	C18	96.2(3)
C3B	C4B	C5B	C6B	1.5(4)	C15	C10	C11	C12	4.3(6)
C4B	C5B	C6B	C7B	1.2(4)	C16	C1	C2	C3	-106.7(4)
C5B	C6B	C7B	C2B	-2.8(4)	C16	C1	C2	C7	66.9(4)
C5B	C6B	C7B	C8B	177.0(2)	C16	C1	C15	C10	-63.8(4)
C6B	C7B	C8B	C9B	-154.1(3)	C16	C1	C15	C14	111.3(4)
C7B	C2B	C3B	C4B	0.7(4)	C16	C1	O1	C18	-22.8(3)
C7B	C2B	C1B	C15B	-63.1(3)	C16	C17	C18	O1	13.5(3)
C7B	C2B	C1B	C16B	65.5(3)	C16	C17	C18	O2	-166.4(3)
C7B	C2B	C1B	O1B	178.29(19)	C17	C18	O1	C1	6.3(3)
C7B	C8B	C9B	C10B	1.7(4)	F1	C17	C18	O1	137.1(2)
C8B	C9B	C10B	C11B	152.2(3)	F1	C17	C18	O2	-42.7(4)
C8B	C9B	C10B	C15B	-25.8(4)	F2	C17	C18	O1	-106.7(3)
C9B	C10B	C11B	C12B	-174.3(2)	F2	C17	C18	O2	73.4(4)
C9B	C10B	C15B	C14B	172.8(2)	O1	C1	C2	C3	5.6(4)
C9B	C10B	C15B	C1B	-11.7(3)	O1	C1	C2	C7	179.2(3)
C10B	C11B	C12B	C13B	-0.3(4)	O1	C1	C15	C10	-176.7(3)
C10B	C15B	C1B	C2B	66.0(3)	O1	C1	C15	C14	-1.7(4)
C10B	C15B	C1B	C16B	-62.5(3)	O1	C1	C16	C17	29.4(3)
C10B	C15B	C1B	O1B	-175.5(2)	O2	C18	O1	C1	-173.8(3)

Torsion Angles for 23DK001_*11*.

A	B	C	D	Angle/°	A	B	C	D	Angle/°
C11B	C10B	C15B	C14B	-5.2(3)	C1A	C2A	C3A	C4A	177(2)
C11B	C10B	C15B	C1B	170.3(2)	C1A	C2A	C7A	C6A	-173(2)
C11B	C12B	C13B	C14B	-1.8(4)	C1A	C2A	C7A	C8A	14(3)
C12B	C13B	C14B	C15B	0.2(4)	C1A	C16A	C17A	C18A	-26.1(16)
C13B	C14B	C15B	C10B	3.3(4)	C1A	C16A	C17A	F1A	-147.5(15)
C13B	C14B	C15B	C1B	-172.2(2)	C1A	C16A	C17A	F2A	92.4(16)
C14B	C15B	C1B	C2B	-118.6(2)	C2A	C1A	C15A	C10A	66(2)
C14B	C15B	C1B	C16B	112.9(3)	C2A	C1A	C15A	C14A	-119.2(19)
C14B	C15B	C1B	O1B	-0.1(3)	C2A	C1A	C16A	C17A	145.8(14)
C15B	C10B	C11B	C12B	3.8(4)	C2A	C1A	O1A	C18A	-141.1(16)
C15B	C1B	C16B	C17B	-85.8(2)	C2A	C3A	C4A	C5A	-9(5)
C15B	C1B	O1B	C18B	96.3(2)	C2A	C7A	C8A	C9A	17(5)
C1B	C2B	C3B	C4B	174.5(2)	C3A	C2A	C7A	C6A	-3(3)
C1B	C2B	C7B	C6B	-171.9(2)	C3A	C2A	C7A	C8A	-176(2)
C1B	C2B	C7B	C8B	8.3(3)	C3A	C4A	C5A	C6A	7(5)
C1B	C16B	C17B	C18B	-26.6(2)	C4A	C5A	C6A	C7A	-2(5)
C1B	C16B	C17B	F1B	-148.0(2)	C5A	C6A	C7A	C2A	0(4)
C1B	C16B	C17B	F2B	90.5(3)	C5A	C6A	C7A	C8A	174(3)
C16B	C1B	O1B	C18B	-22.5(2)	C6A	C7A	C8A	C9A	-157(3)
C16B	C17B	C18B	O1B	14.4(3)	C7A	C2A	C3A	C4A	7(4)
C16B	C17B	C18B	O2B	-166.2(3)	C7A	C8A	C9A	C10A	9(5)
C17B	C18B	O1B	C1B	5.6(3)	C8A	C9A	C10A	C11A	147(3)
F1B	C17B	C18B	O1B	138.60(19)	C8A	C9A	C10A	C15A	-27(4)
F1B	C17B	C18B	O2B	-42.0(4)	C9A	C10A	C11A	C12A	-177(2)
F2B	C17B	C18B	O1B	-105.7(2)	C9A	C10A	C15A	C1A	-13(3)
F2B	C17B	C18B	O2B	73.7(3)	C9A	C10A	C15A	C14A	173(2)
O1B	C1B	C16B	C17B	29.7(2)	C10A	C11A	C12A	C13A	5(4)
O2B	C18B	O1B	C1B	-173.9(2)	C11A	C10A	C15A	C1A	173(2)

Torsion Angles for 23DK001_*11*.

A	B	C	D	Angle/˚	A	B	C	D	Angle/˚
C1	C2	C3	C4	173.1(4)	C11A	C10A	C15A	C14A	-2(3)
C1	C2	C7	C6	-170.7(4)	C11A	C12A	C13A	C14A	-5(4)
C1	C2	C7	C8	8.5(6)	C12A	C13A	C14A	C15A	1(3)
C1	C16	C17	C18	-25.9(3)	C13A	C14A	C15A	C1A	-173(2)
C1	C16	C17	F1	-146.7(3)	C13A	C14A	C15A	C10A	2(3)
C1	C16	C17	F2	91.5(3)	C15A	C1A	C2A	C3A	126(2)
C2	C1	C15	C10	65.2(4)	C15A	C1A	C2A	C7A	-64(2)
C2	C1	C15	C14	-119.7(4)	C15A	C1A	C16A	C17A	-86.9(15)
C2	C1	C16	C17	143.7(3)	C15A	C1A	O1A	C18A	96.6(17)
C2	C1	O1	C18	-140.5(3)	C15A	C10A	C11A	C12A	-2(4)
C2	C3	C4	C5	-1.6(7)	C16A	C1A	C2A	C3A	-108(2)
C2	C7	C8	C9	25.3(8)	C16A	C1A	C2A	C7A	62(2)
C3	C2	C7	C6	2.9(6)	C16A	C1A	C15A	C10A	-61(2)
C3	C2	C7	C8	-177.9(4)	C16A	C1A	C15A	C14A	114.2(18)
C3	C4	C5	C6	1.1(8)	C16A	C1A	O1A	C18A	-21.6(17)
C4	C5	C6	C7	1.5(8)	C16A	C17A	C18A	O1A	15(2)
C5	C6	C7	C2	-3.5(7)	C16A	C17A	C18A	O2A	-167(2)
C5	C6	C7	C8	177.3(5)	C17A	C18A	O1A	C1A	5(2)
C6	C7	C8	C9	-155.6(6)	F1A	C17A	C18A	O1A	138.5(15)
C7	C2	C3	C4	-0.4(6)	F1A	C17A	C18A	O2A	-43(3)
C7	C8	C9	C10	1.1(9)	F2A	C17A	C18A	O1A	-104.9(16)
C8	C9	C10	C11	153.7(5)	F2A	C17A	C18A	O2A	74(3)
C8	C9	C10	C15	-24.8(7)	O1A	C1A	C2A	C3A	7(2)
C9	C10	C11	C12	-174.4(4)	O1A	C1A	C2A	C7A	177.0(17)
C9	C10	C15	C1	-11.7(5)	O1A	C1A	C15A	C10A	-175.3(16)
C9	C10	C15	C14	173.1(4)	O1A	C1A	C15A	C14A	0(2)
C10	C11	C12	C13	-0.8(7)	O1A	C1A	C16A	C17A	29.4(15)
C11	C10	C15	C1	169.7(4)	O2A	C18A	O1A	C1A	-173.8(18)

Hydrogen Atom Coordinates (Å×10^4) and Isotropic Displacement Parameters (Å^2×10^3) for 23DK001_*11*.

**Atom**	***x***	***y***	***z***	**U(eq)**
H3B	9892.18	10951.83	1860.67	45
H4B	12232.25	11369.06	2117.11	51
H5B	13666.62	8559.15	1866.02	54
H6B	12757.62	5421.37	1325.99	49
H8B	10905.19	3095.69	918.89	47
H9B	9073.43	2661.74	297.72	46
H11B	7539.16	4309.55	-480.96	48
H12B	5924.89	6998.26	-861.55	52
H13B	5502.63	10130.83	-313.53	51
H14B	6640.5	10476.49	628.02	44
H16A	7829.5	4183.21	1366.79	44
H16B	8607.97	5256.39	1960.14	44
H3	5061.76	6507.35	3167.25	44
H4	2723.9	6789.81	2882.44	49
H5	1323.43	3904.96	3120.27	52
H6	2261.03	793.95	3662.29	48
H8	4132.18	-1423.38	4085.23	49
H9	5954.02	-1820.8	4711.68	47
H11	7511.96	-216.28	5488.67	48
H12	9109.28	2480.62	5875.95	51
H13	9506.91	5654.7	5334.12	50
H14	8349.46	6040	4402.22	42
H16C	7215.36	-186	3631.01	39
H16D	6411.99	919.09	3046.21	39
H3A	5086.35	5490.93	3100.67	43
H4A	2737.4	6050.76	2931.36	47

Hydrogen Atom Coordinates (Å×10^4) and Isotropic Displacement Parameters (Å^2×10^3) for 23DK001_*11*.

Atom	*x*	*y*	*z*	U(eq)
H5A	1313.18	3158.26	3134.11	37
H6A	2252.42	44.52	3697.8	41
H8A	4038.98	-2065.9	4126.98	37
H9A	5943.08	-2666.96	4691.33	34
H11A	7351.84	-958.82	5490.42	42
H12A	9077.04	1528.88	5846.11	41
H13A	9501.46	4722.31	5331.45	44
H14A	8369.38	5121.36	4364.28	35
H16E	7146.74	-1196.68	3645.88	39
H16F	6371.27	-174.54	3045.63	39

Atomic Occupancy for 23DK001_*11*.

Atom	*Occupancy*	Atom	*Occupancy*	Atom	*Occupancy*
C1	0.851(9)	C2	0.851(9)	C3	0.851(9)
H3	0.851(9)	C4	0.851(9)	H4	0.851(9)
C5	0.851(9)	H5	0.851(9)	C6	0.851(9)
H6	0.851(9)	C7	0.851(9)	C8	0.851(9)
H8	0.851(9)	C9	0.851(9)	H9	0.851(9)
C10	0.851(9)	C11	0.851(9)	H11	0.851(9)
C12	0.851(9)	H12	0.851(9)	C13	0.851(9)
H13	0.851(9)	C14	0.851(9)	H14	0.851(9)
C15	0.851(9)	C16	0.851(9)	H16C	0.851(9)
H16D	0.851(9)	C17	0.851(9)	C18	0.851(9)
F1	0.851(9)	F2	0.851(9)	O1	0.851(9)
O2	0.851(9)	C1A	0.149(9)	C2A	0.149(9)
C3A	0.149(9)	H3A	0.149(9)	C4A	0.149(9)

Atomic Occupancy for 23DK001_*11*.

**Atom**	***Occupancy***	**Atom**	***Occupancy***	**Atom**	***Occupancy***
H4A	0.149(9)	C5A	0.149(9)	H5A	0.149(9)
C6A	0.149(9)	H6A	0.149(9)	C7A	0.149(9)
C8A	0.149(9)	H8A	0.149(9)	C9A	0.149(9)
H9A	0.149(9)	C10A	0.149(9)	C11A	0.149(9)
H11A	0.149(9)	C12A	0.149(9)	H12A	0.149(9)
C13A	0.149(9)	H13A	0.149(9)	C14A	0.149(9)
H14A	0.149(9)	C15A	0.149(9)	C16A	0.149(9)
H16E	0.149(9)	H16F	0.149(9)	C17A	0.149(9)
C18A	0.149(9)	F1A	0.149(9)	F2A	0.149(9)
O1A	0.149(9)	O2A	0.149(9)		

12. NMR spectra of isolated compounds

^{1}H NMR (500 MHz, $CDCl_3$) of **4**

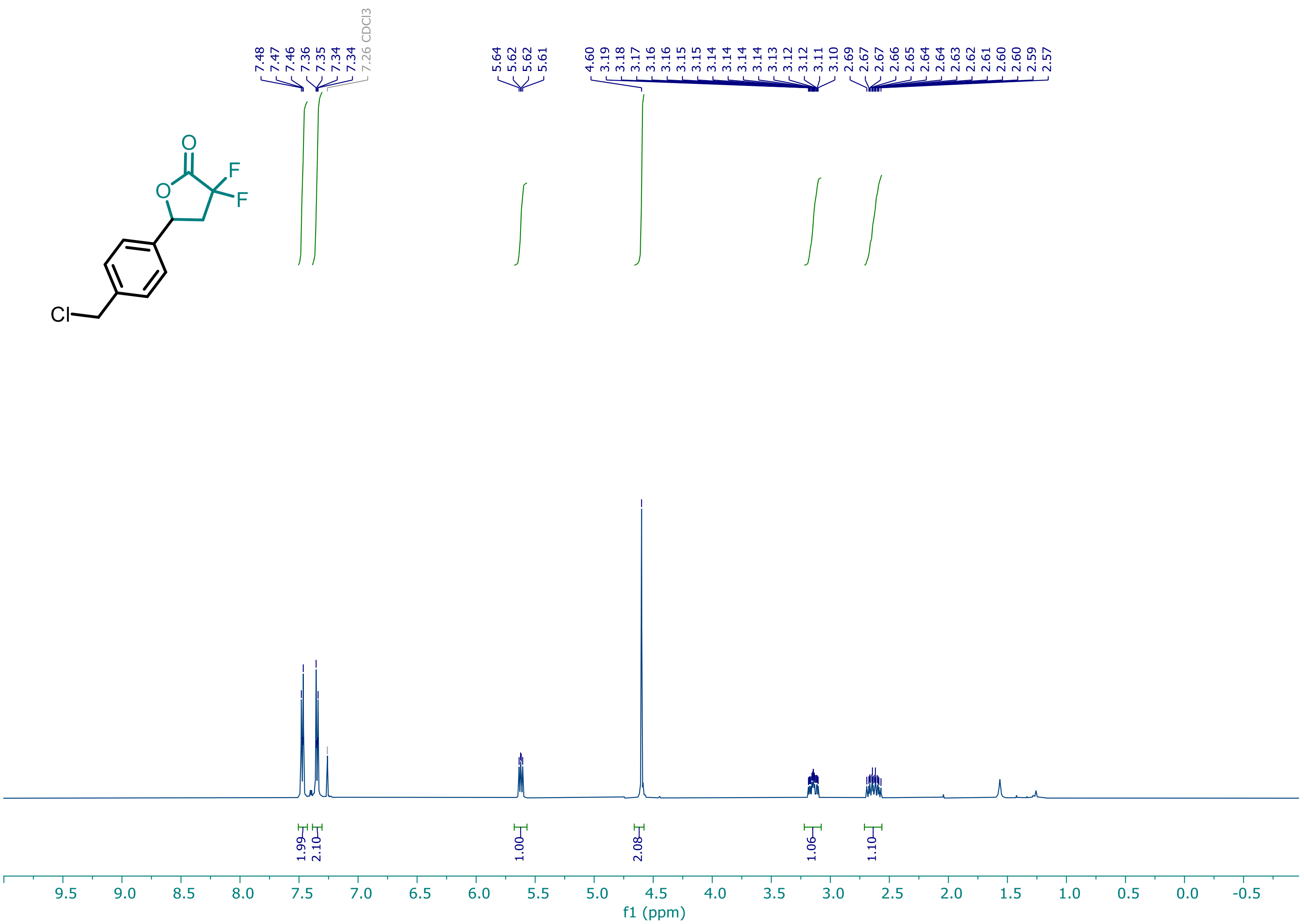

^{13}C NMR (126 MHz, $CDCl_3$) of **4**

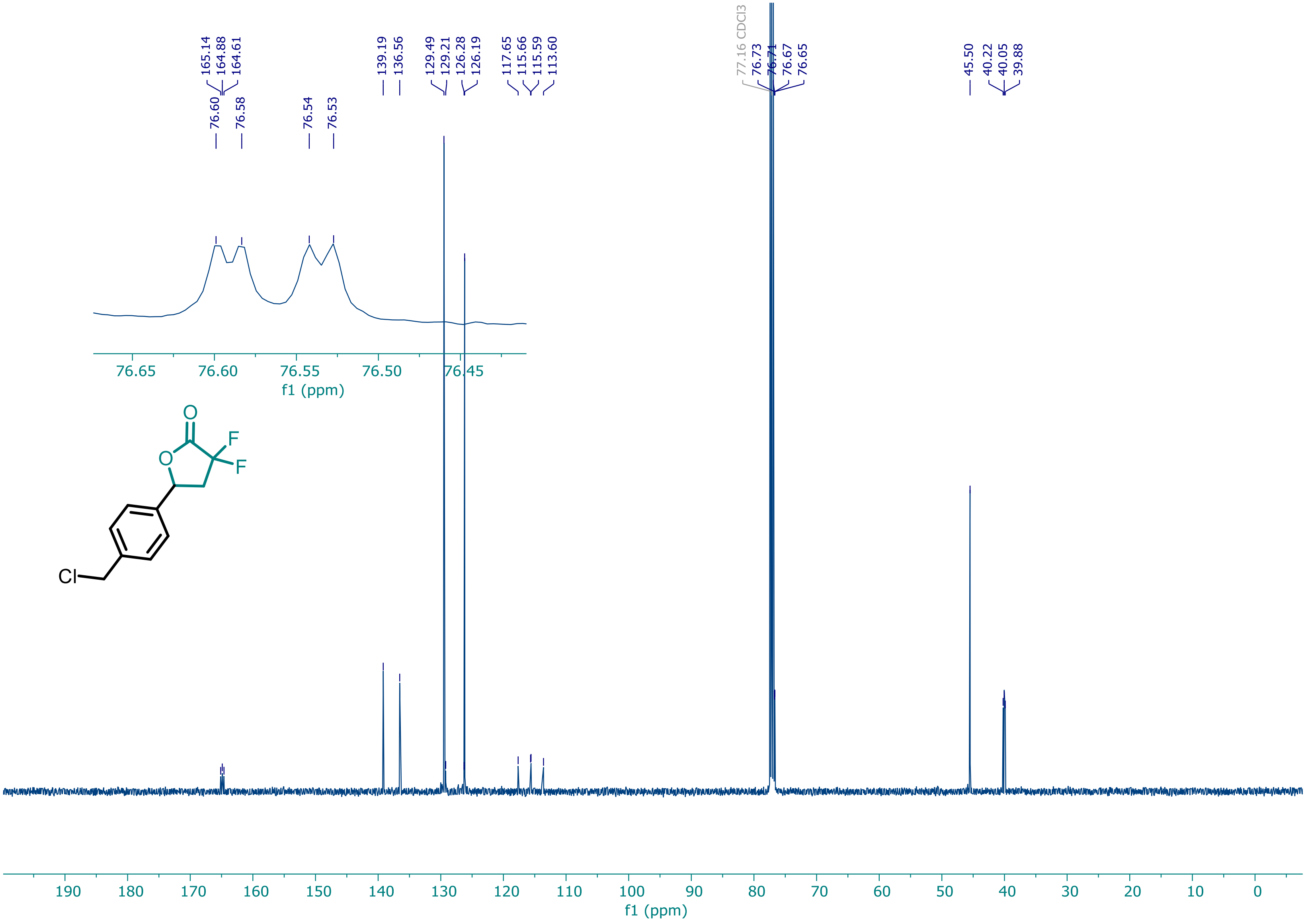

^{19}F NMR (282 MHz, $CDCl_3$) of **4**

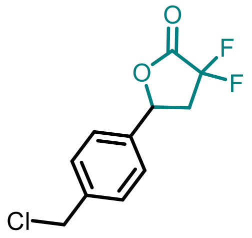

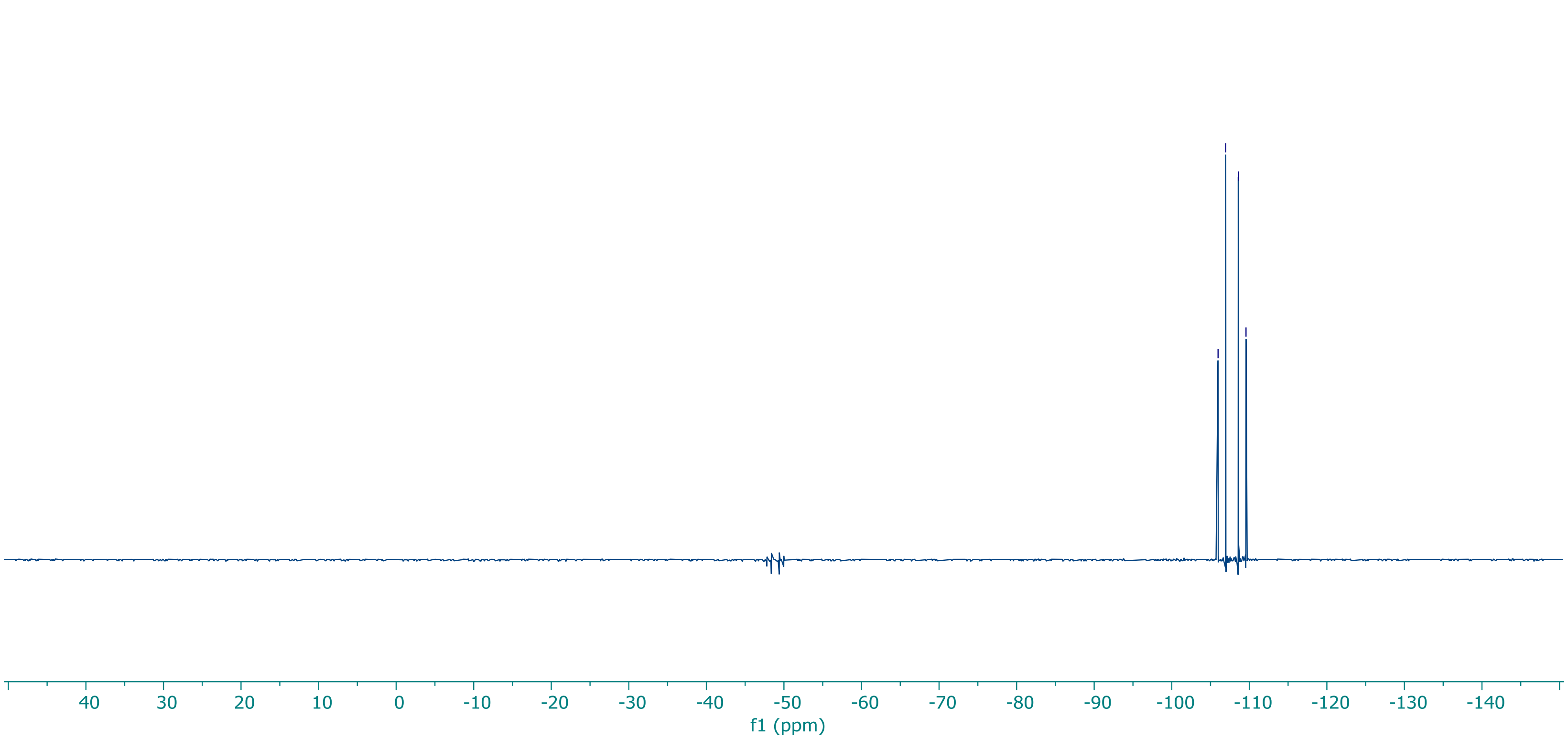

^{1}H NMR (300 MHz, $CDCl_3$) of **5**

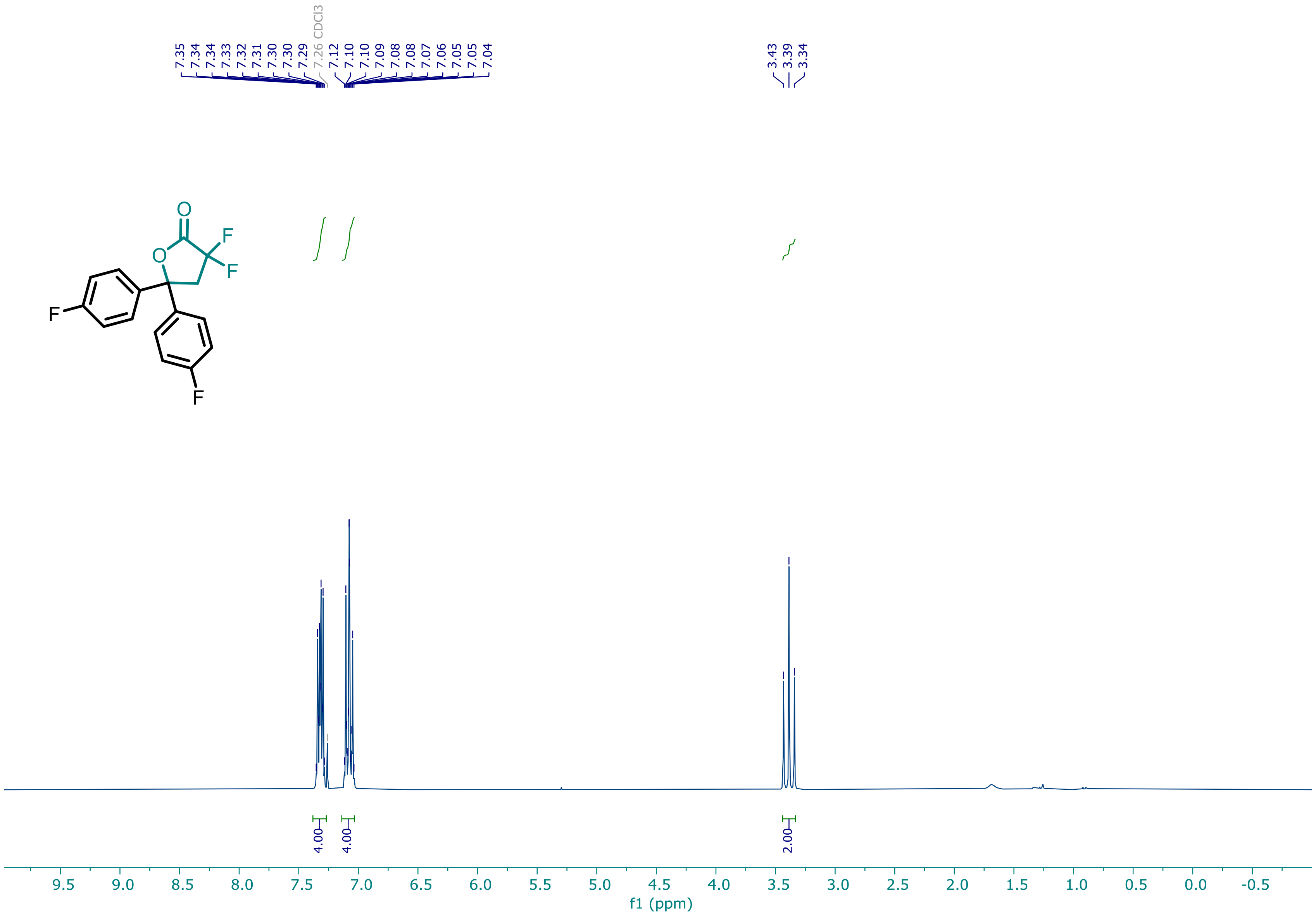

^{13}C NMR (75 MHz, $CDCl_3$) of **5**

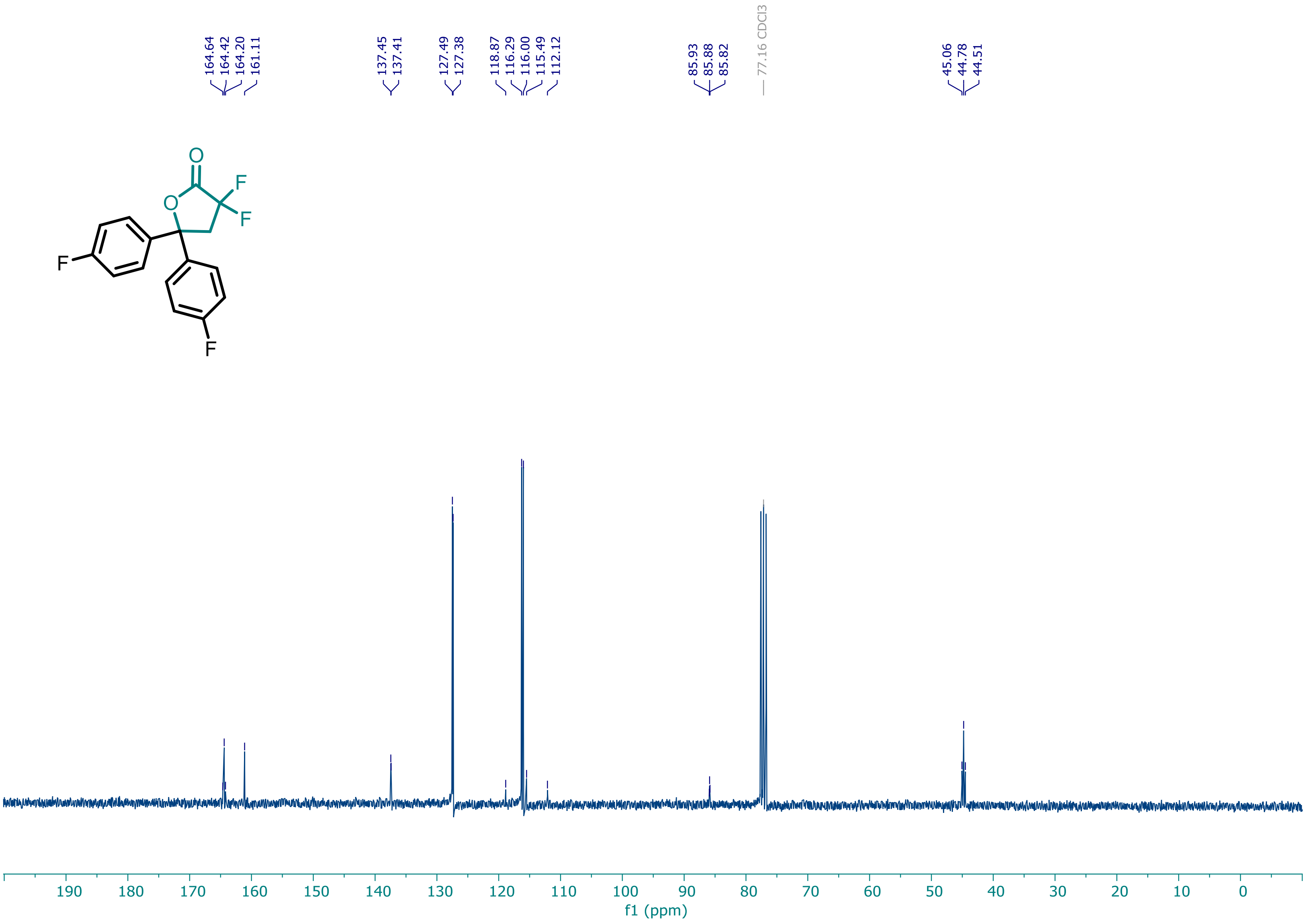

^{19}F NMR (282 MHz, $CDCl_3$) of **5**

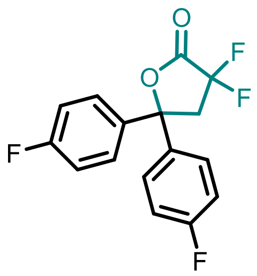

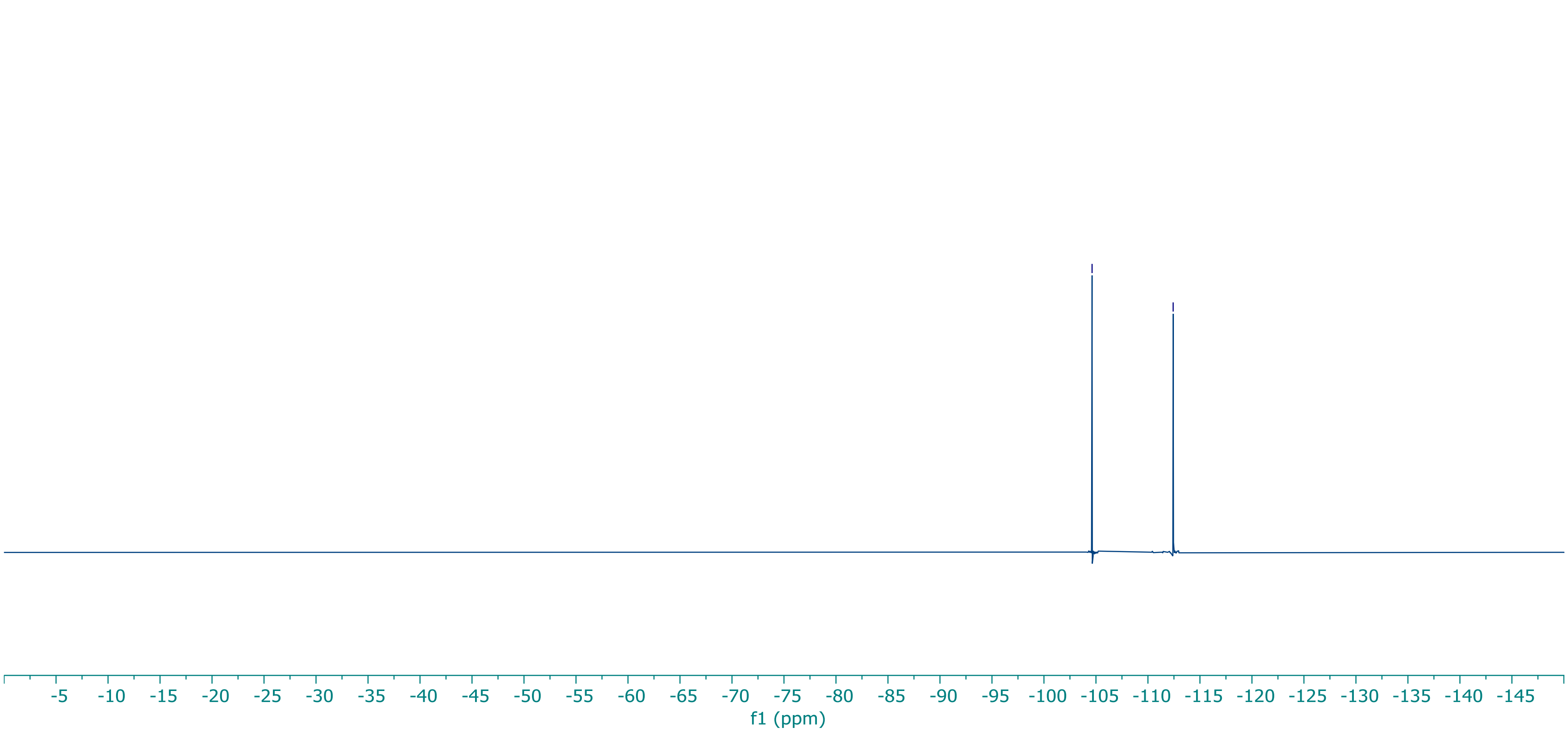

^{1}H NMR (300 MHz, $CDCl_3$) of **6**

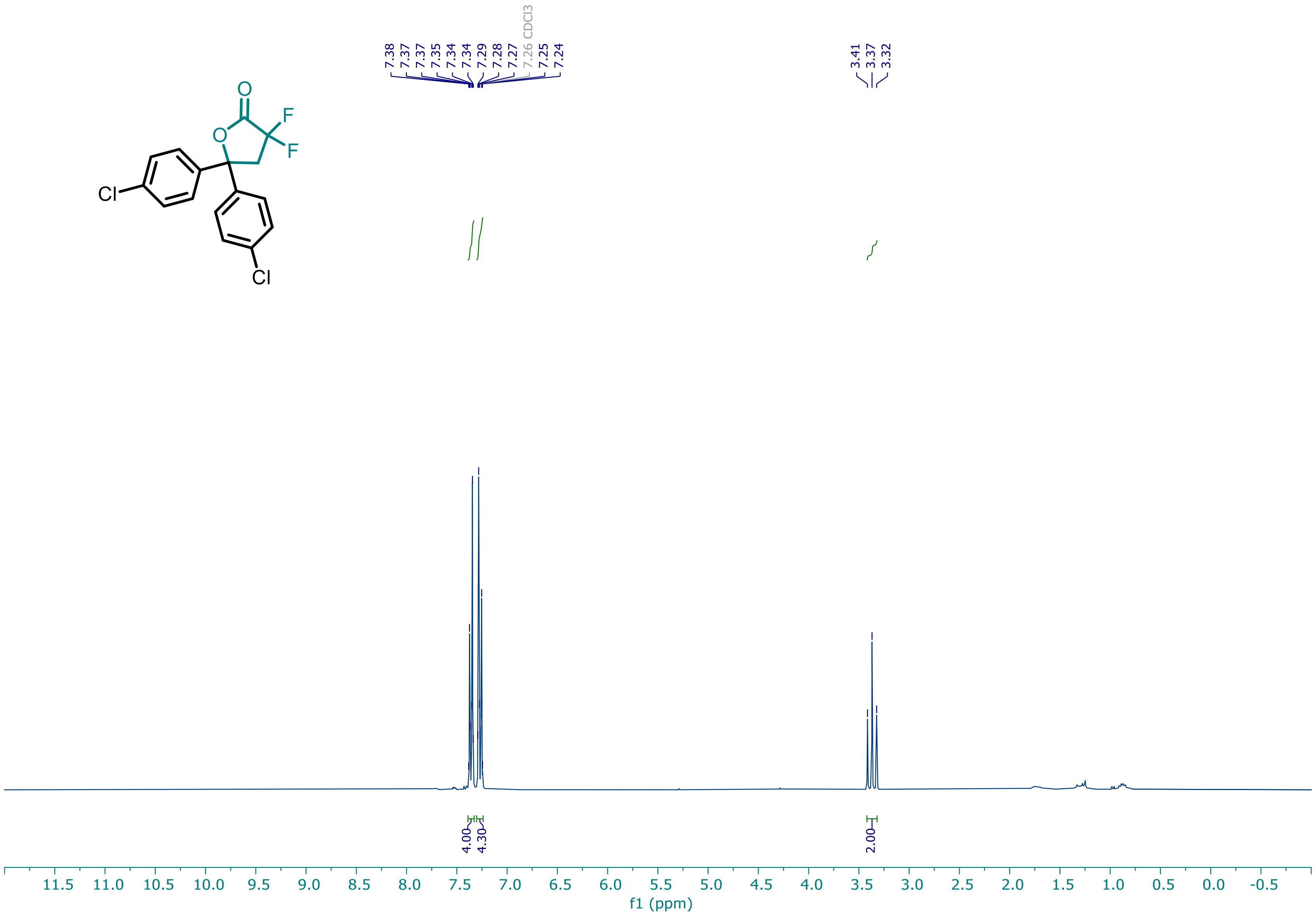

^{13}C NMR (75 MHz, $CDCl_3$) of **6**

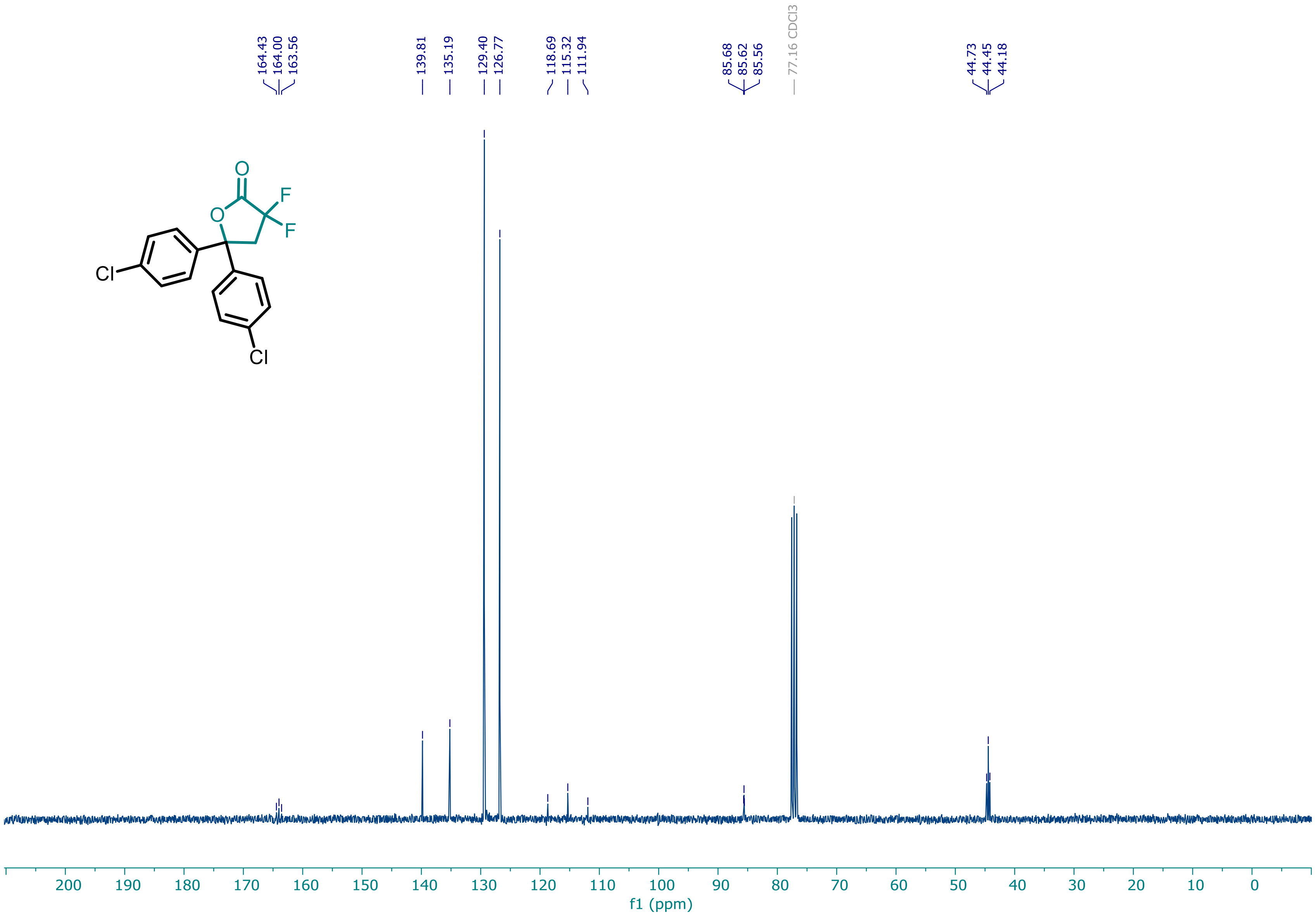

^{19}F NMR (282 MHz, $CDCl_3$) of **6**

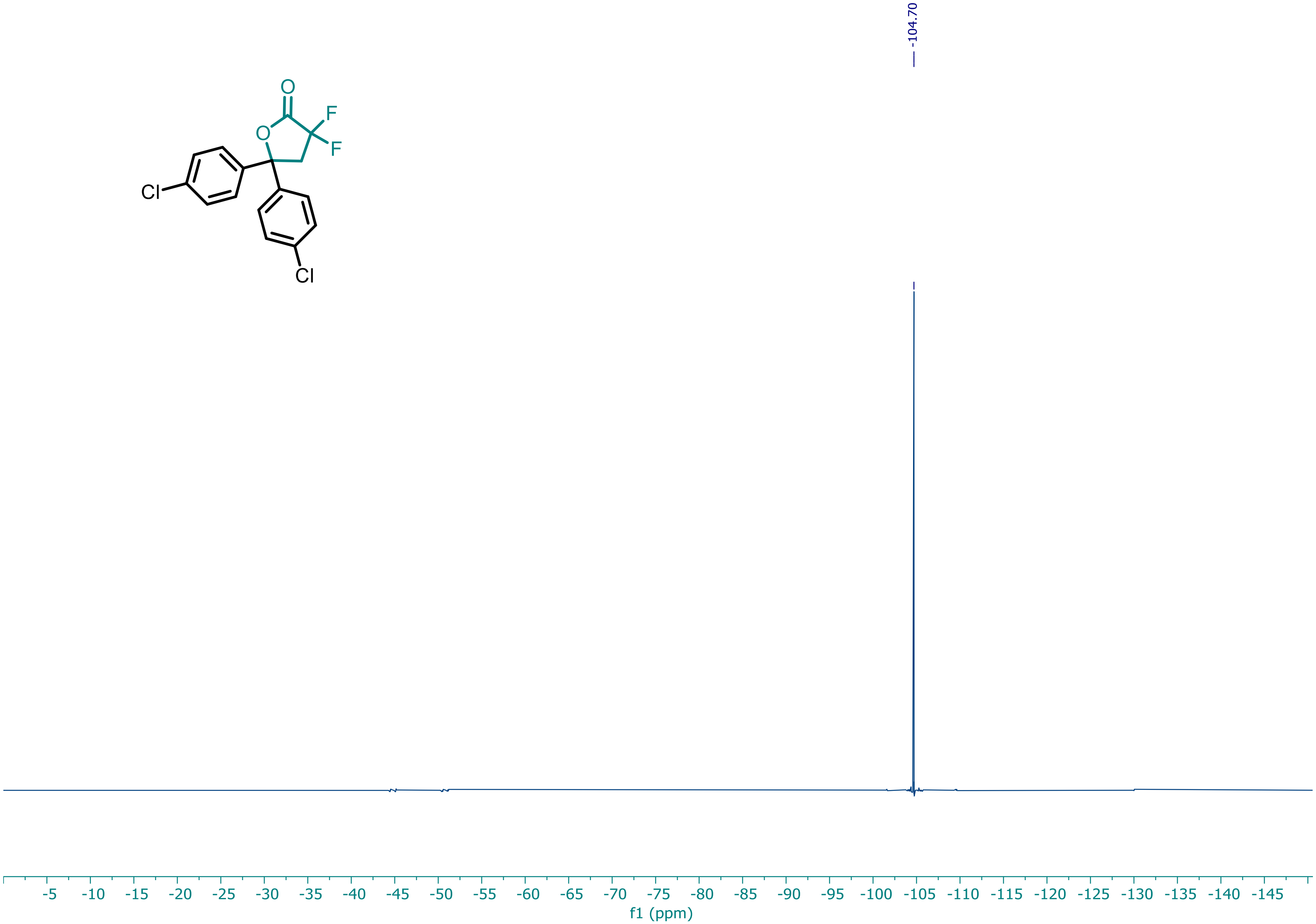

^{1}H NMR (300 MHz, $CDCl_3$) of **7**

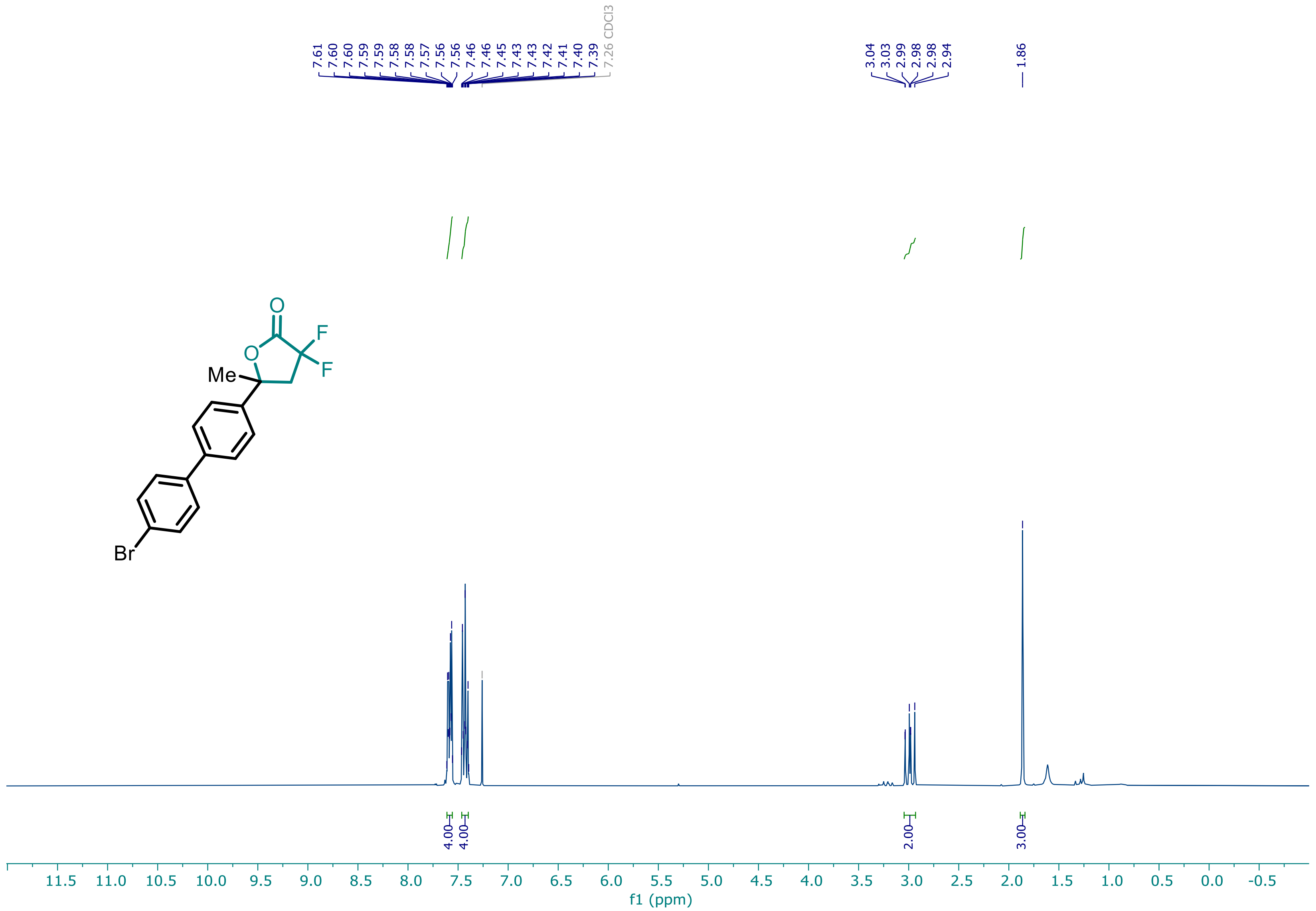

^{13}C NMR (126MHz, $CDCl_3$) of **7**

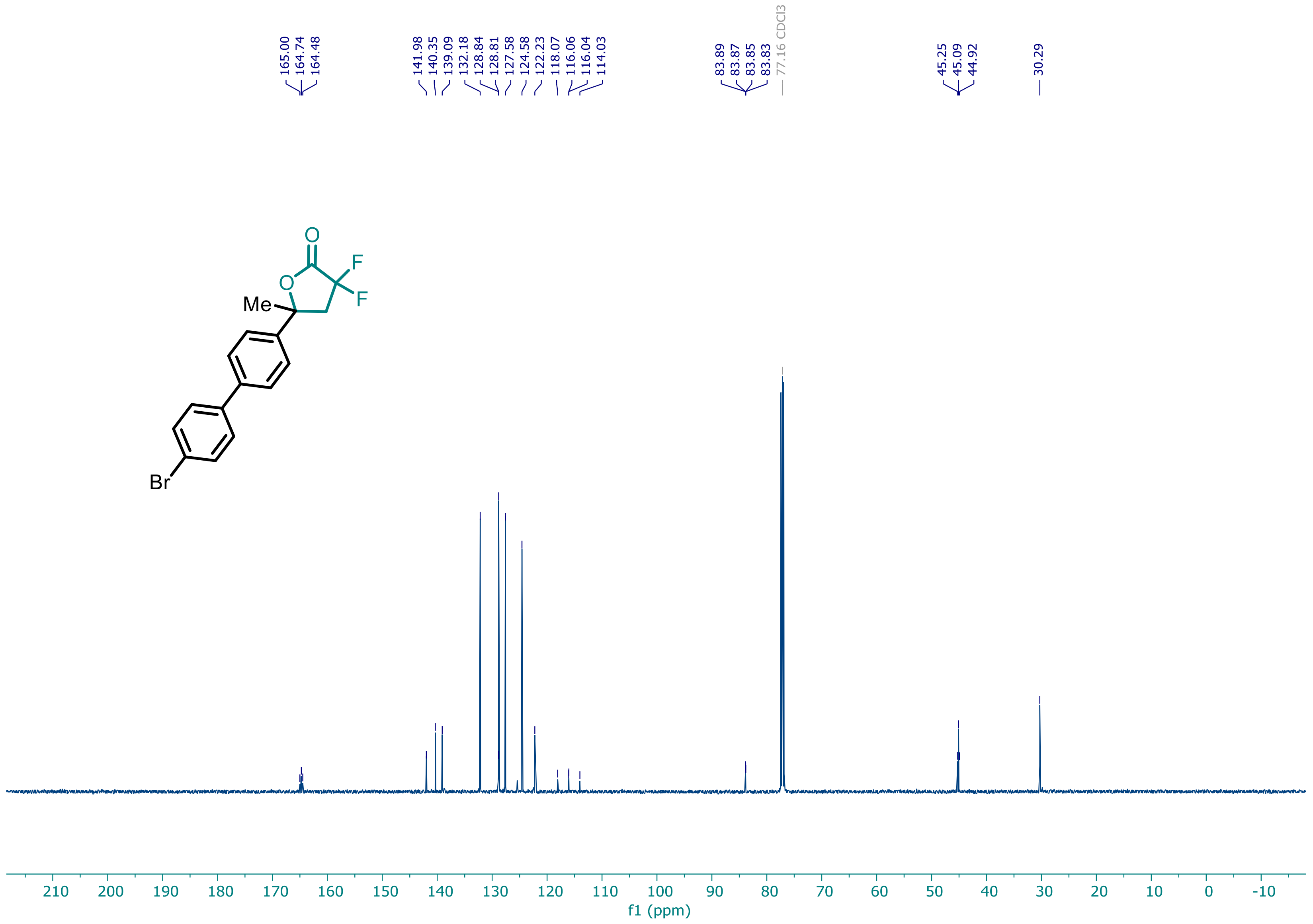

^{19}F NMR (282 MHz, $CDCl_3$) of **7**

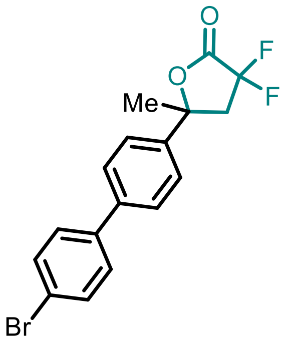

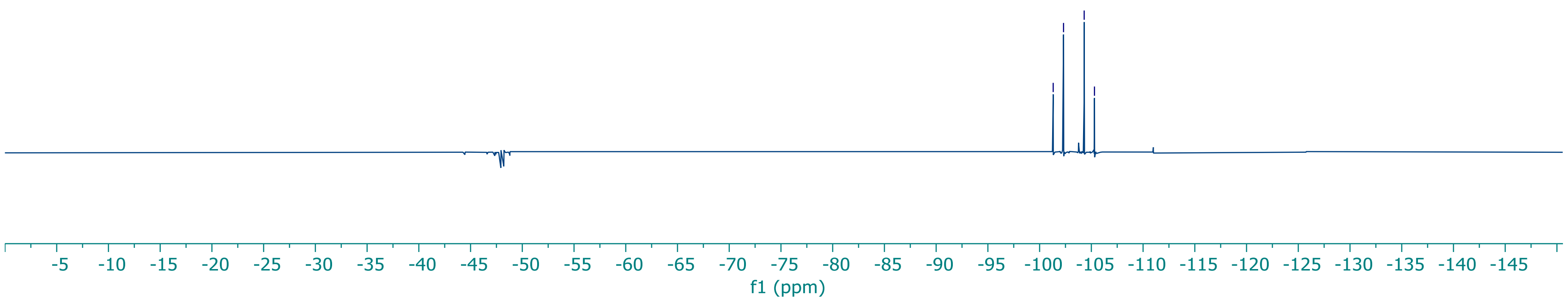

^{1}H NMR (500 MHz, $CDCl_3$) of **8**

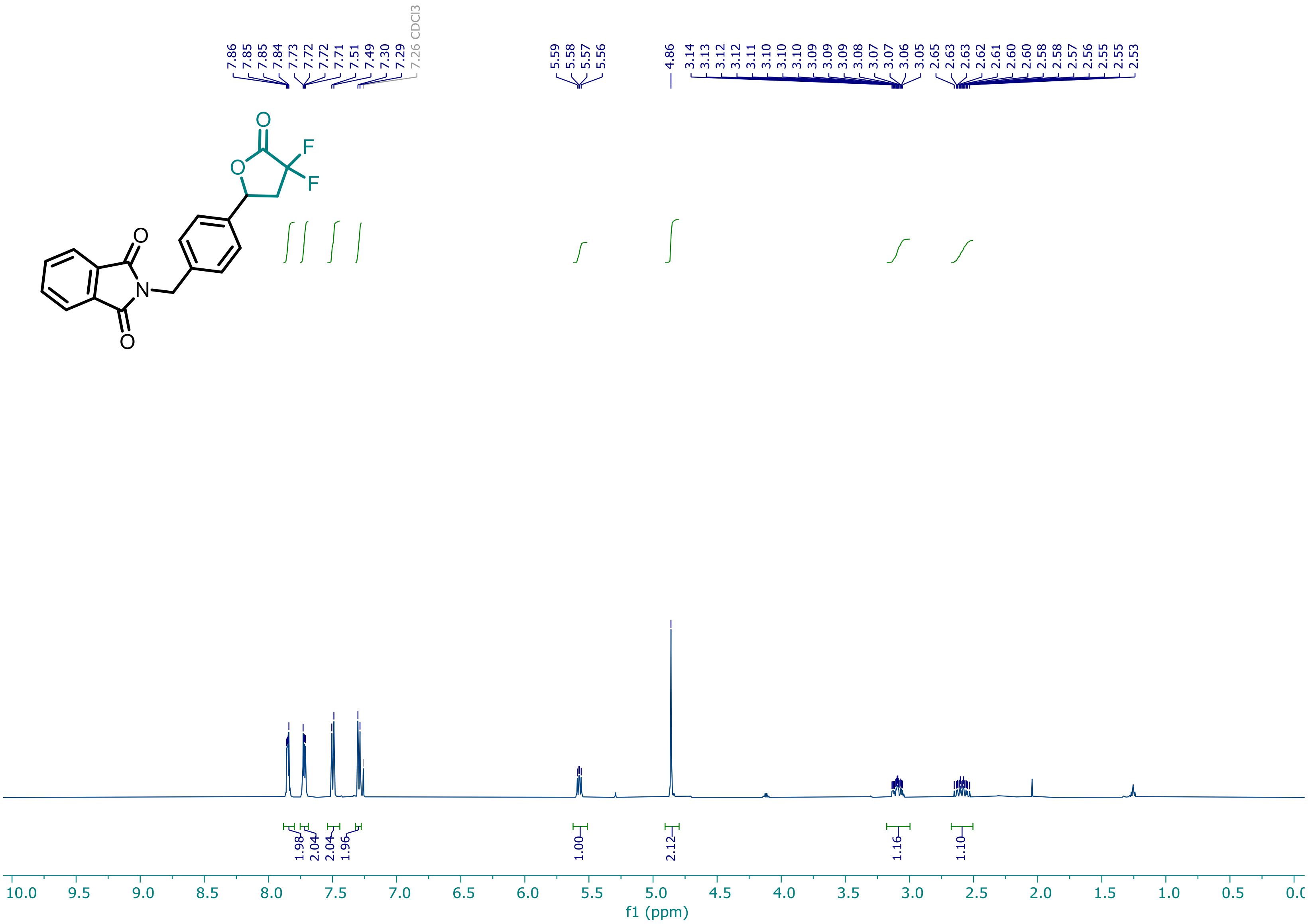

^{13}C NMR (126 MHz, $CDCl_3$) of **8**

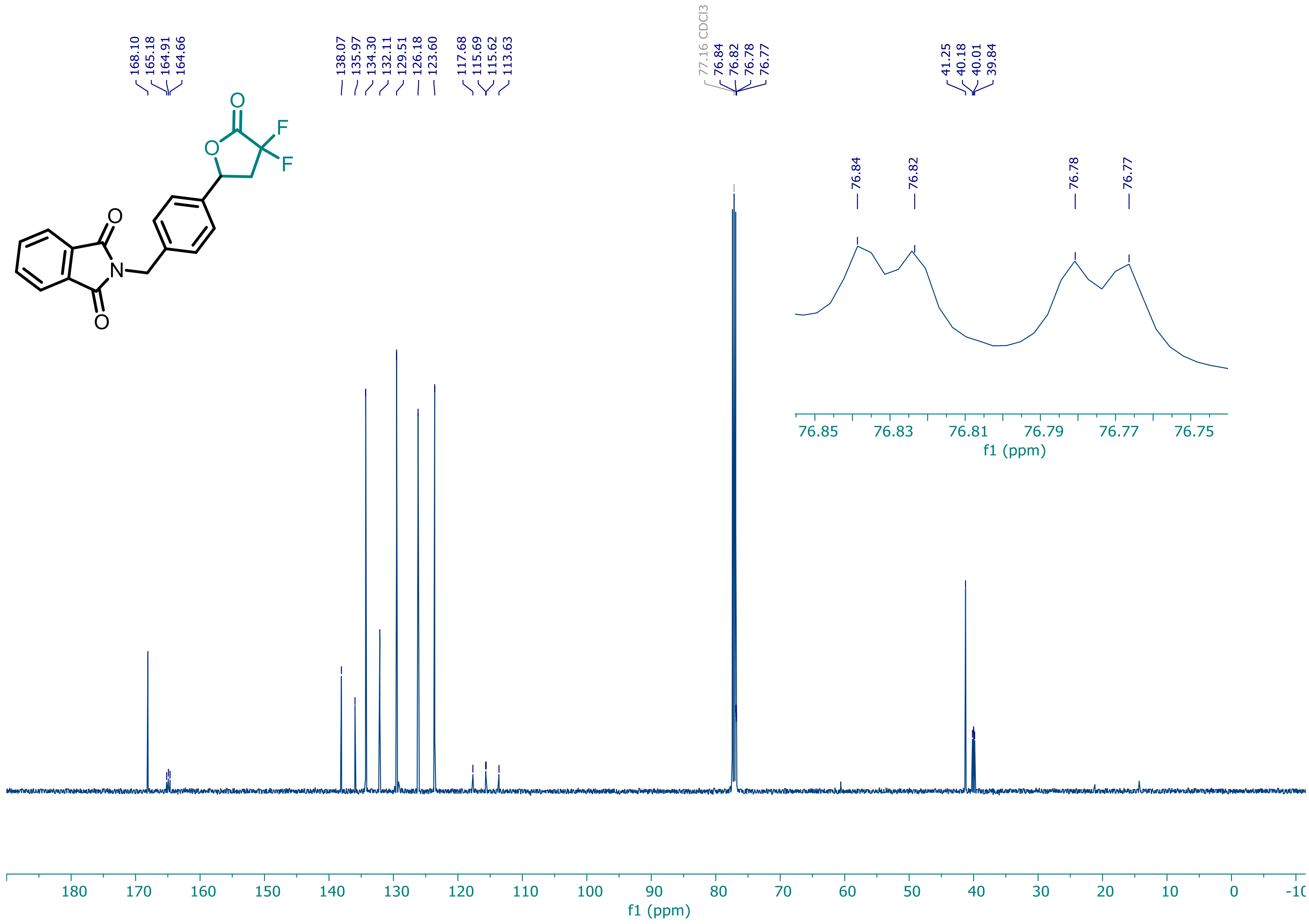

^{19}F NMR (282 MHz, $CDCl_3$) of **8**

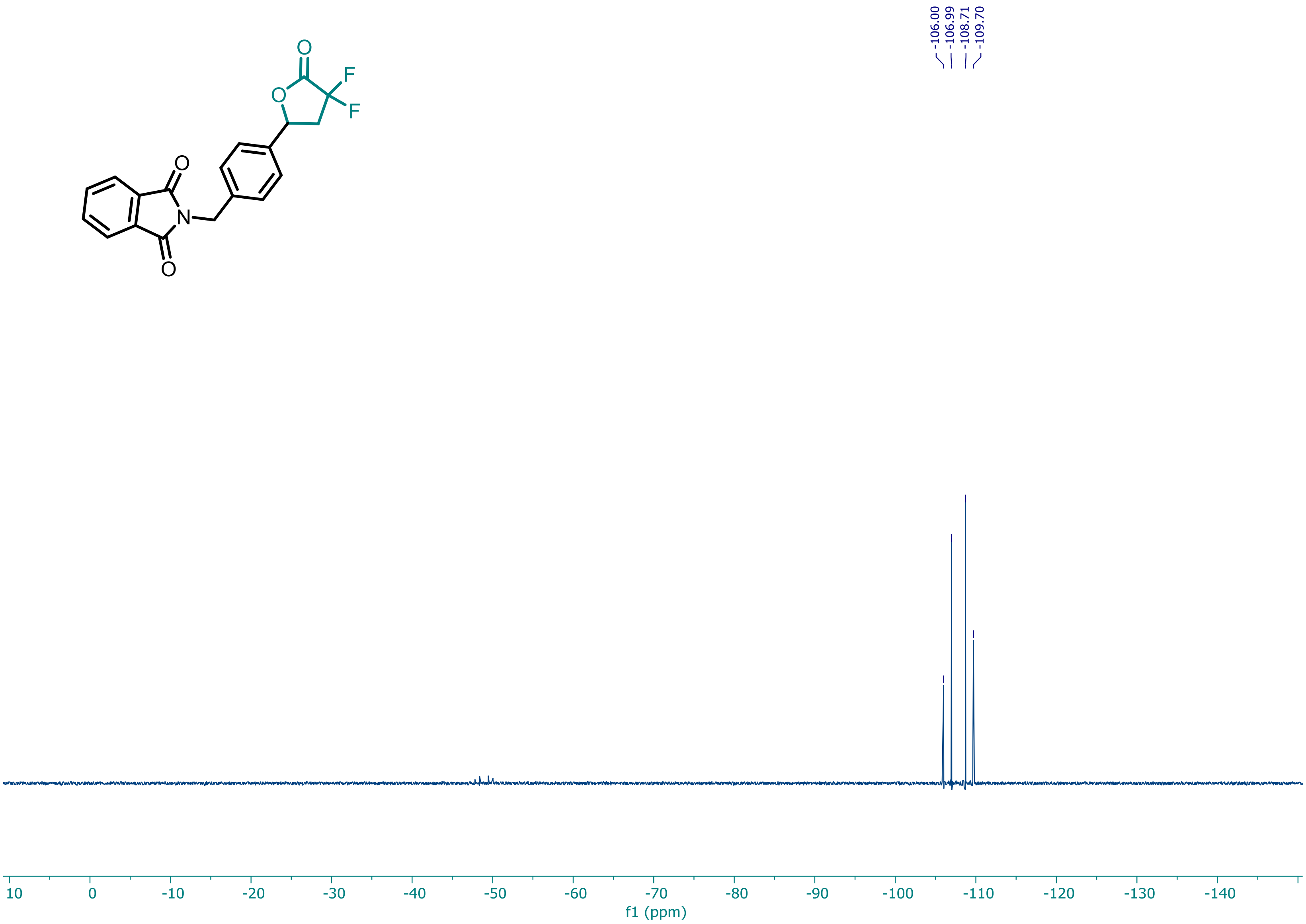

^{1}H NMR (300 MHz, $CDCl_3$) of **9**

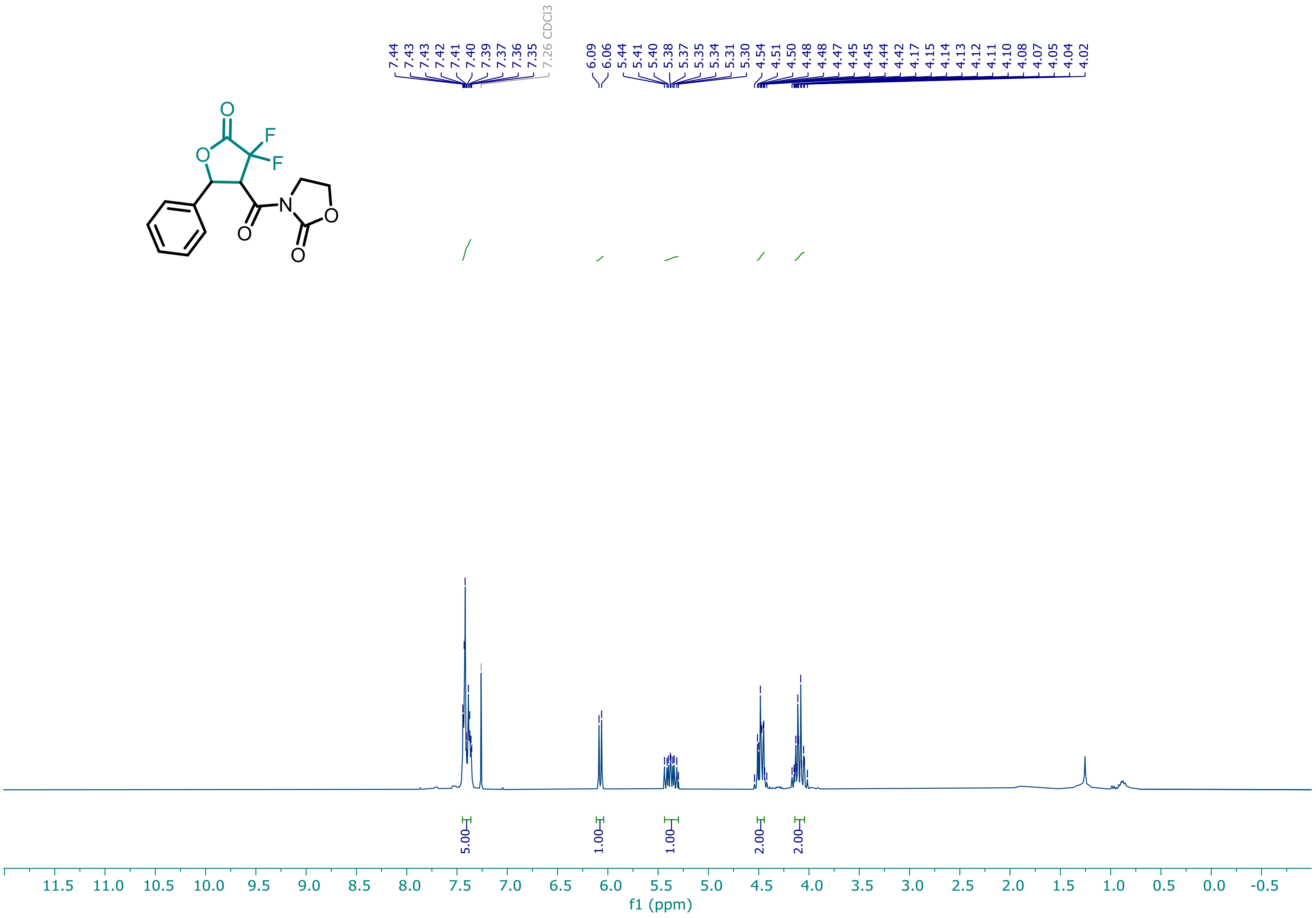

^{13}C NMR (101 MHz, $CDCl_3$) of **9**

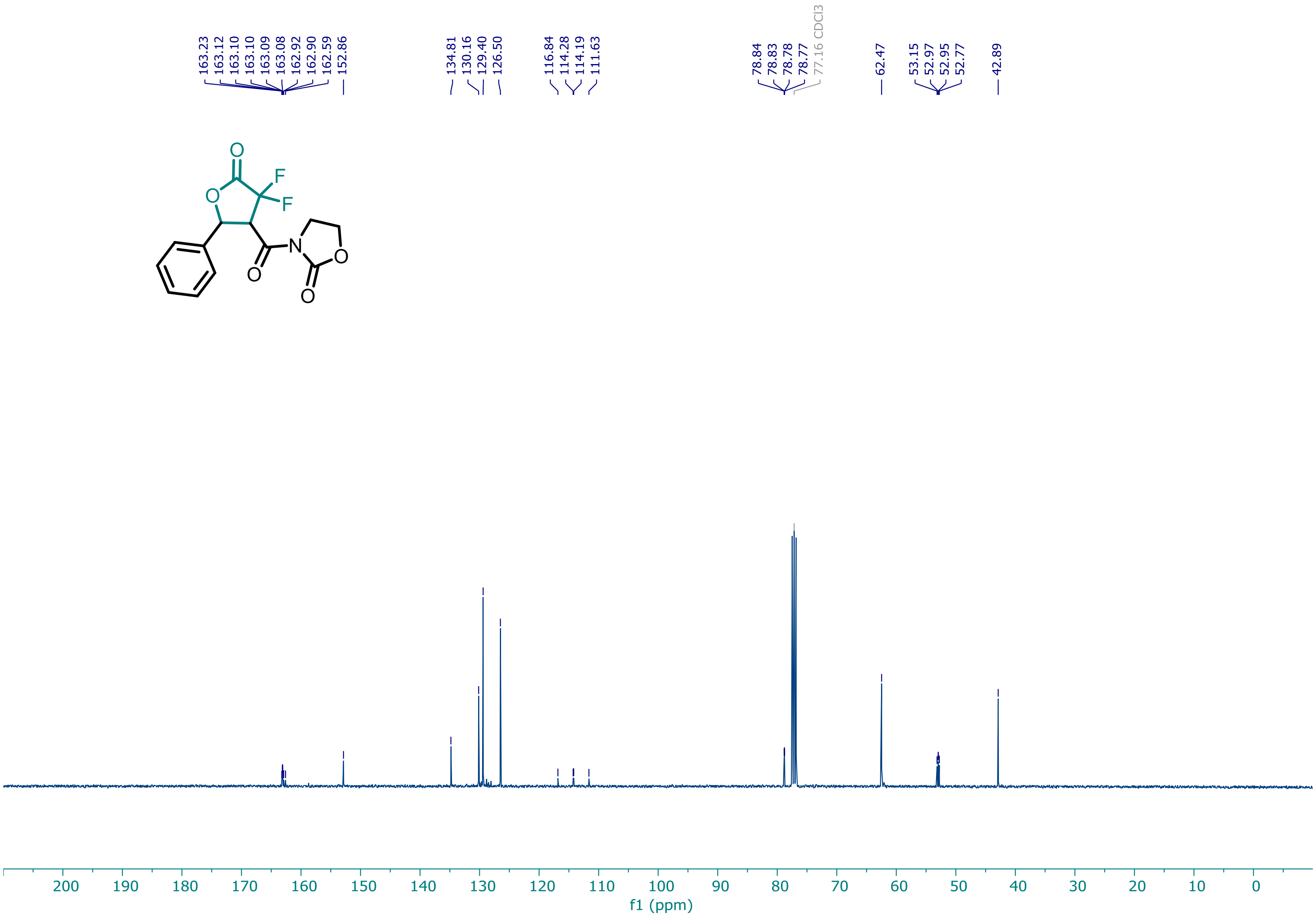

^{19}F NMR (282 MHz, $CDCl_3$) of **9**

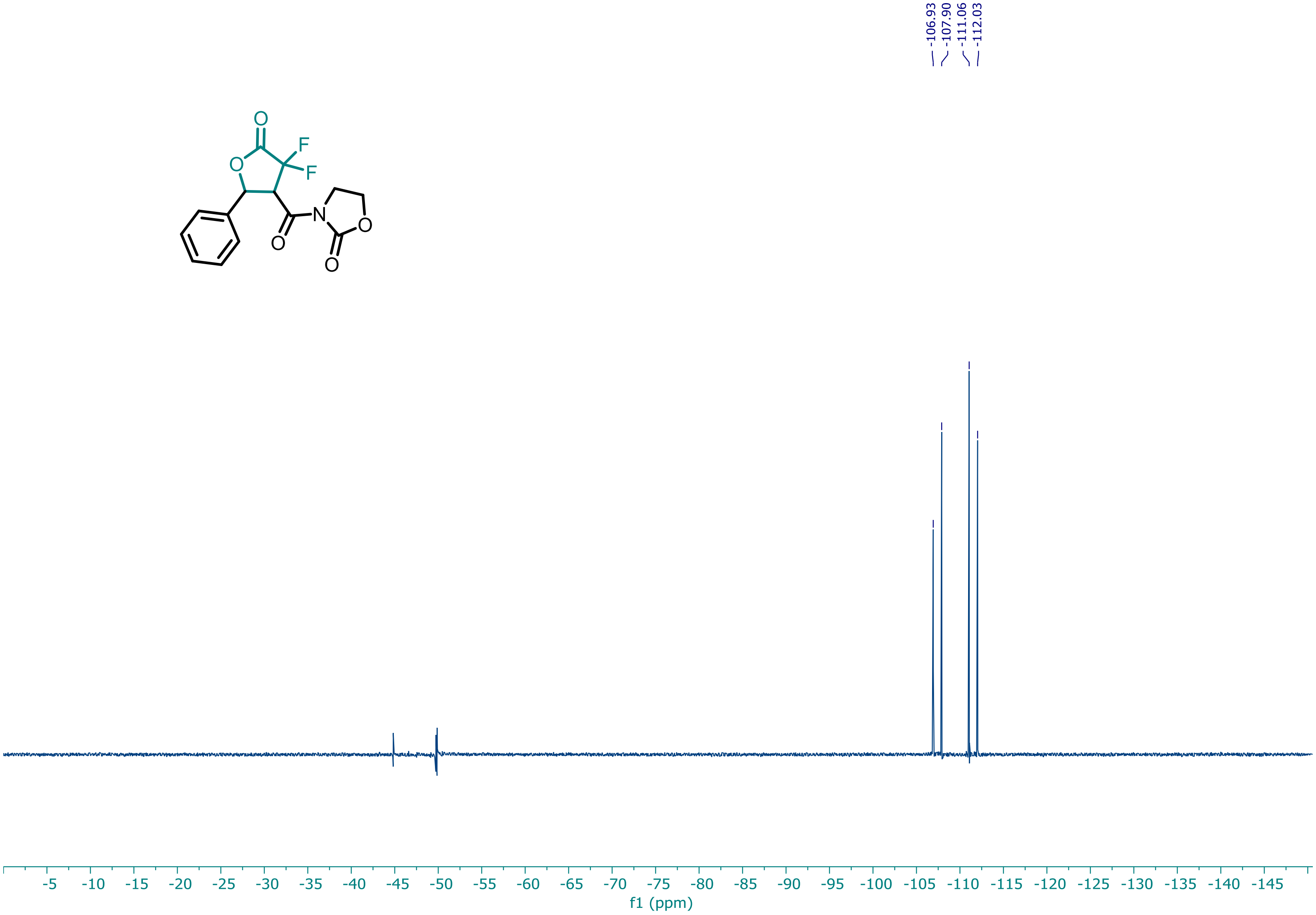

^{1}H NMR (300 MHz, $CDCl_3$) of **10**

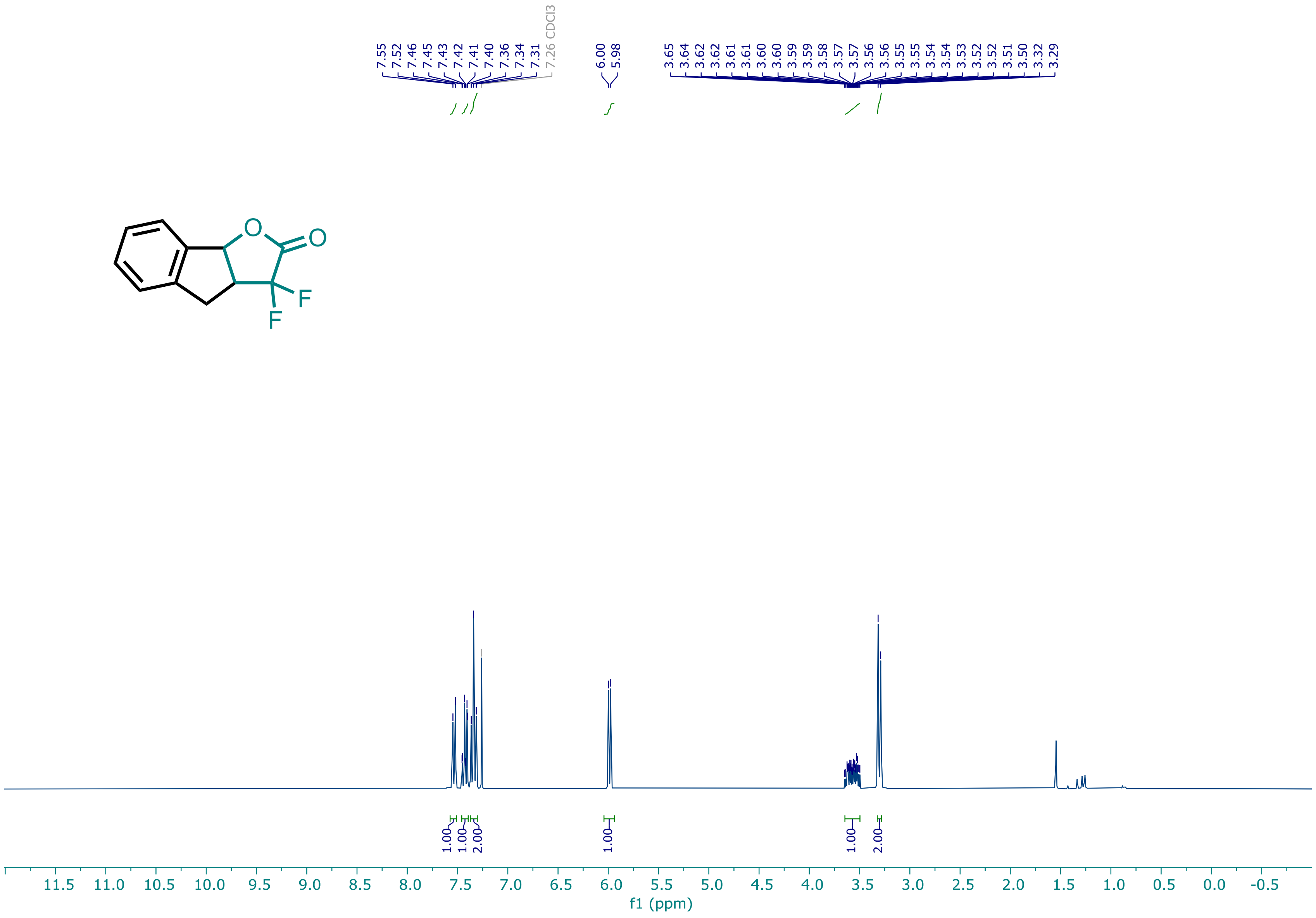

^{13}C NMR (75 MHz, $CDCl_3$) of **10**

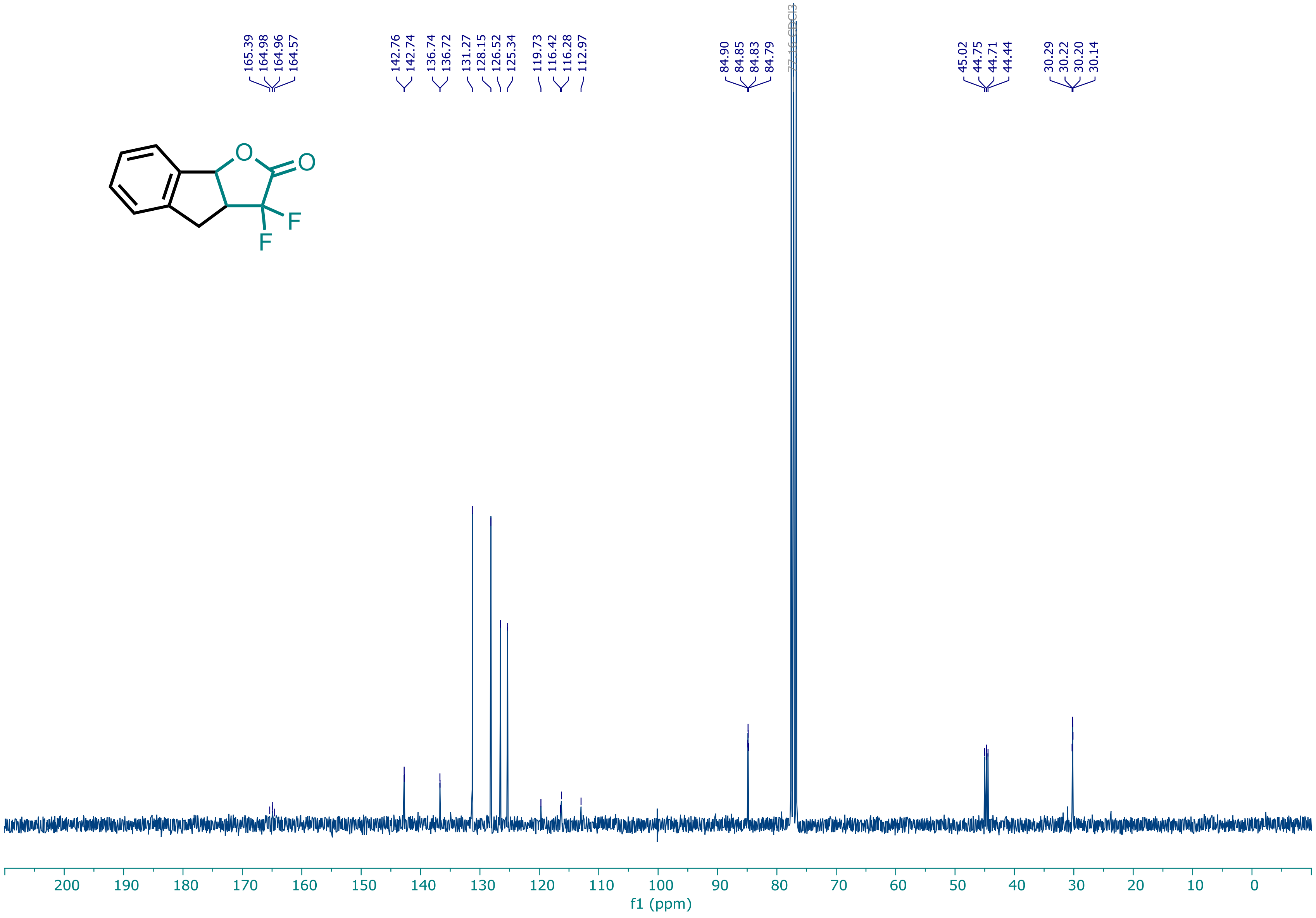

^{19}F NMR (282 MHz, $CDCl_3$) of **10**

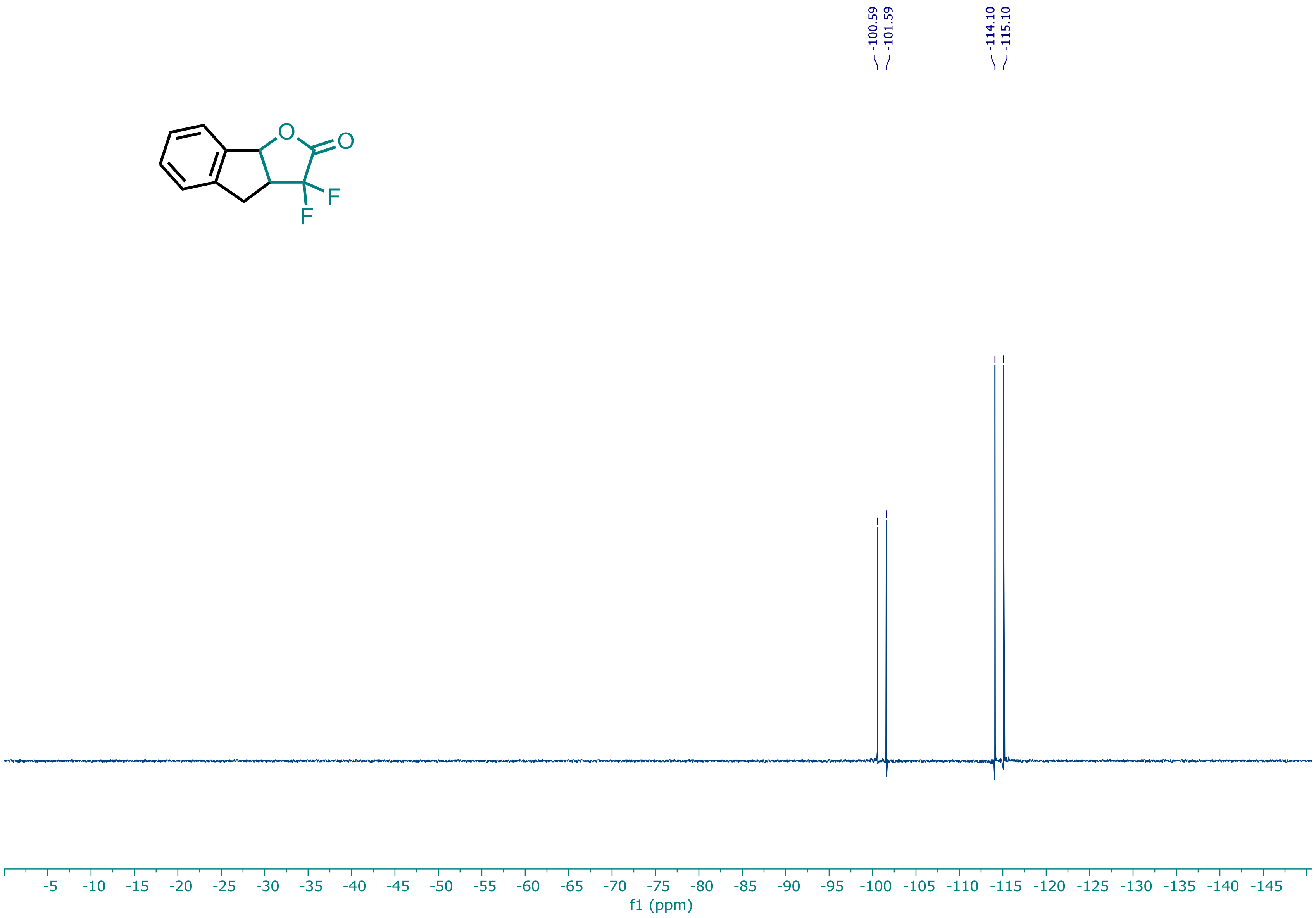

^{1}H NMR (300 MHz, $CDCl_3$) of **11**

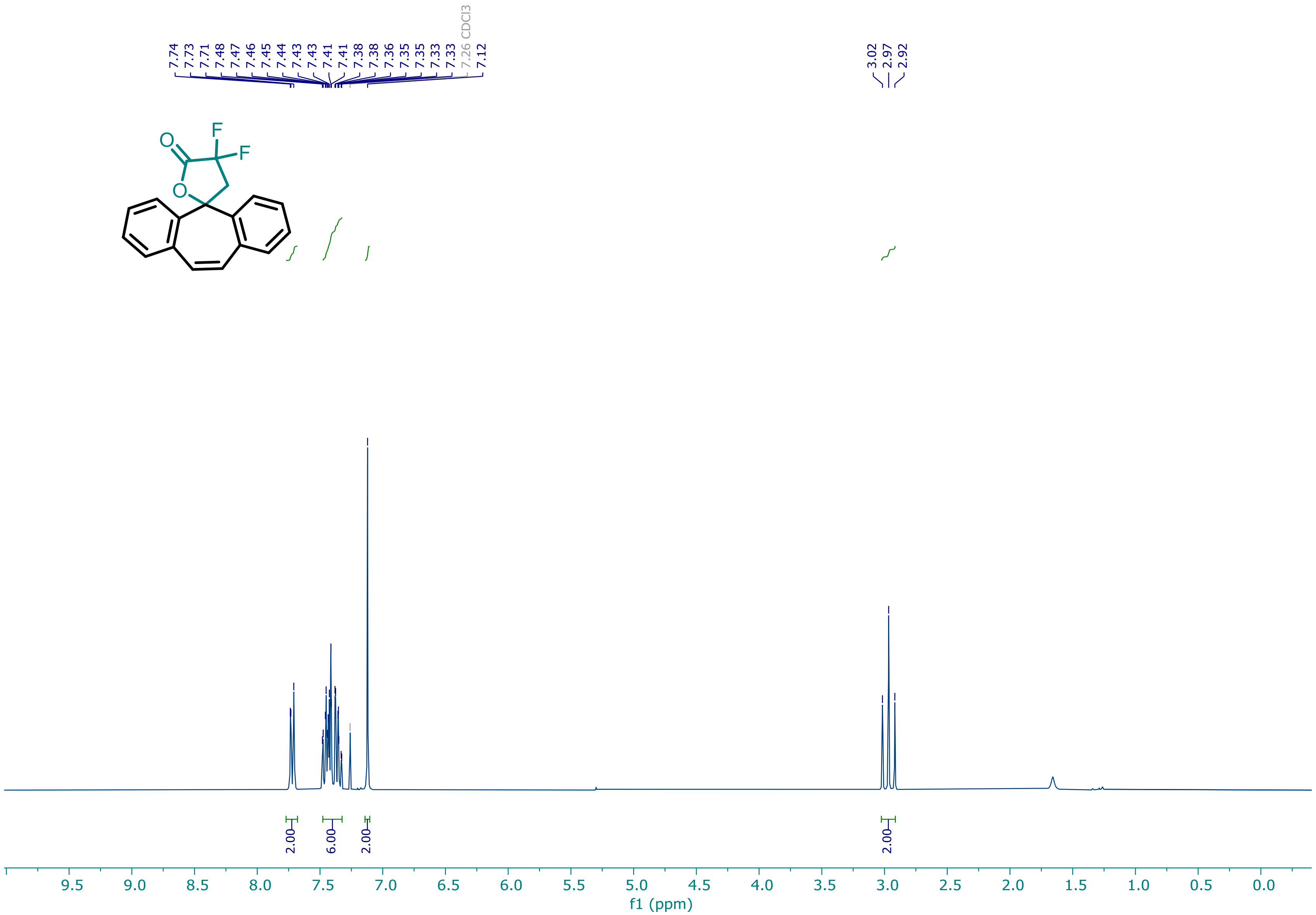

^{13}C NMR (75 MHz, $CDCl_3$) of **11**

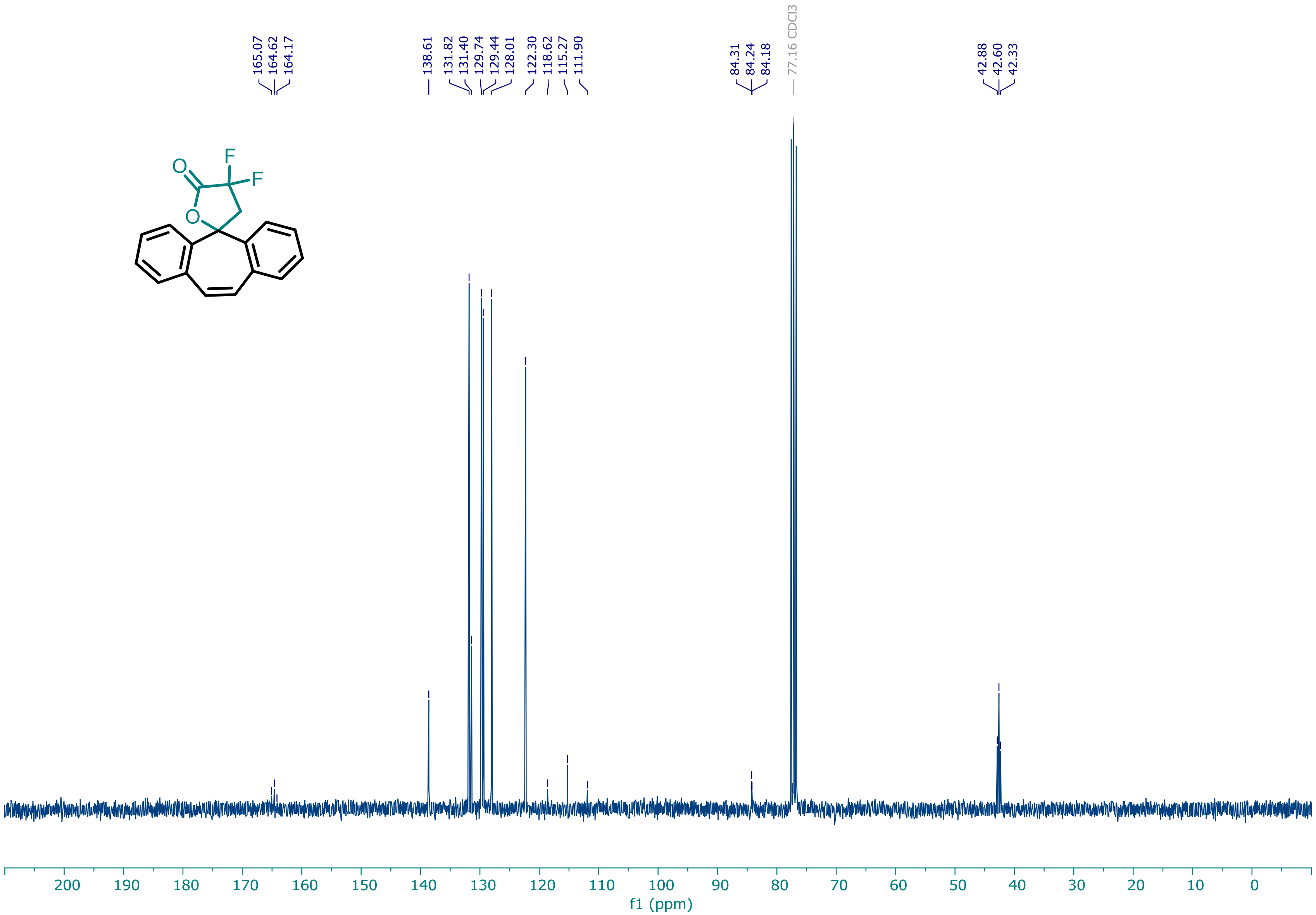

^{19}F NMR (282 MHz, $CDCl_3$) of **11**

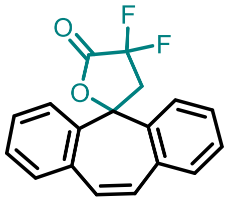

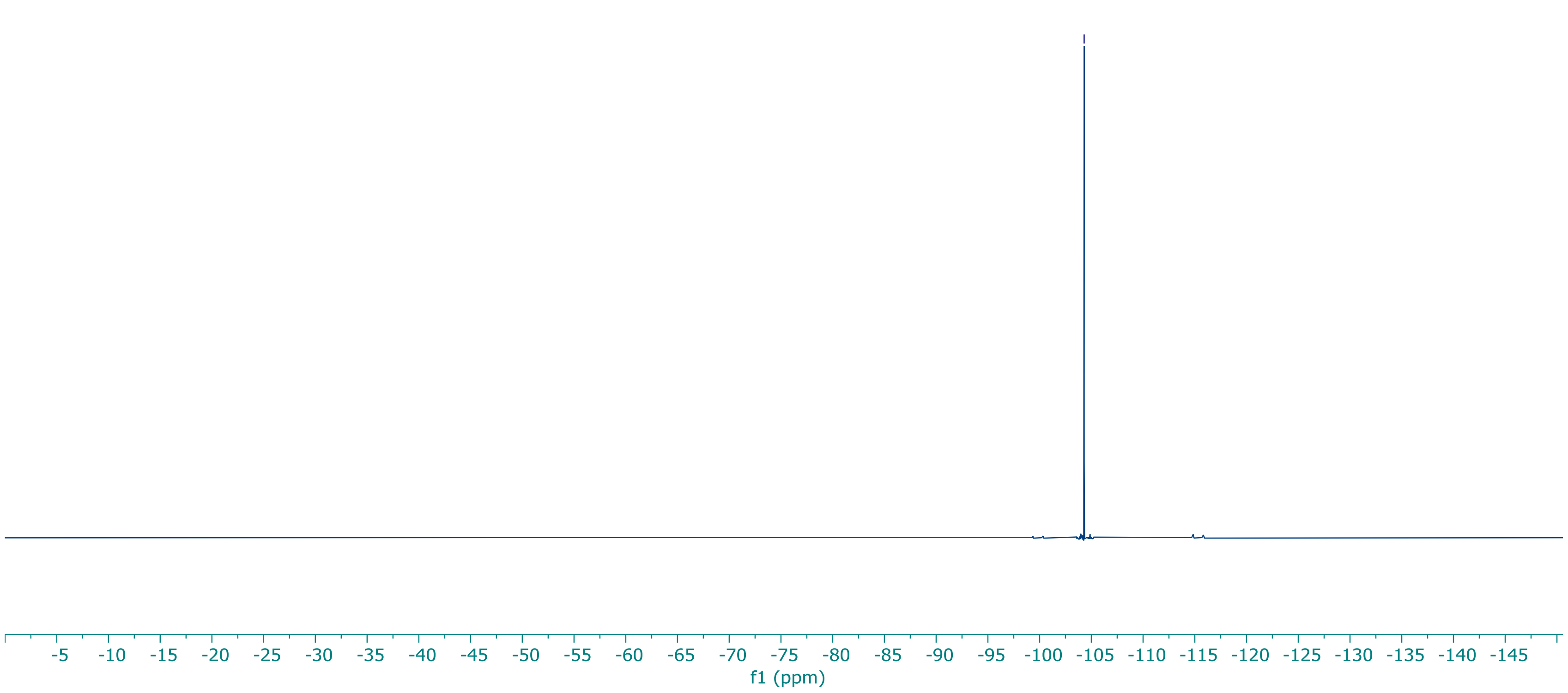

^{1}H NMR (300 MHz, $CDCl_3$) of **12**

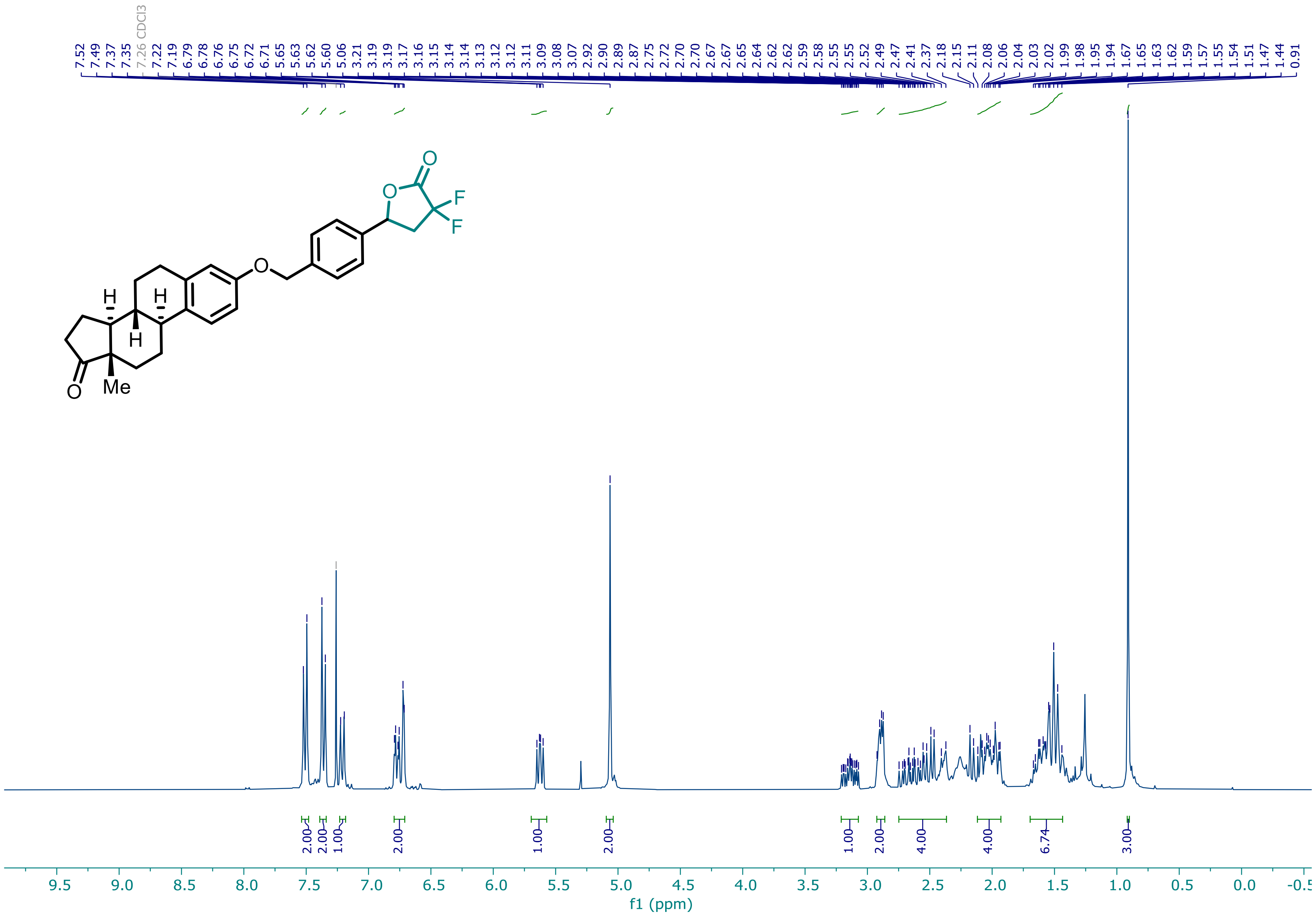

^{13}C NMR (101 MHz, CDCl$_3$) of **12**

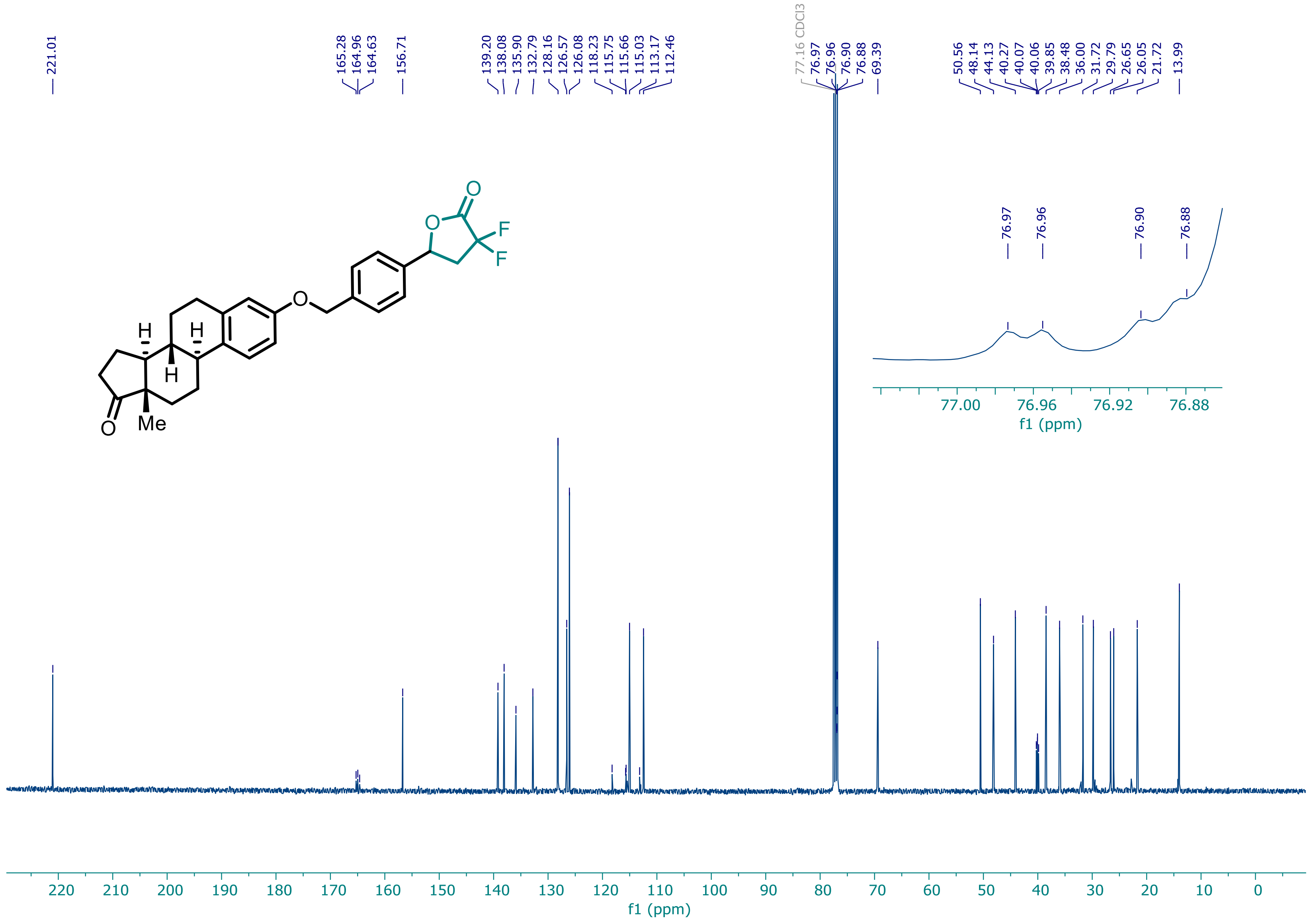

^{19}F NMR (282 MHz, $CDCl_3$) of **12**

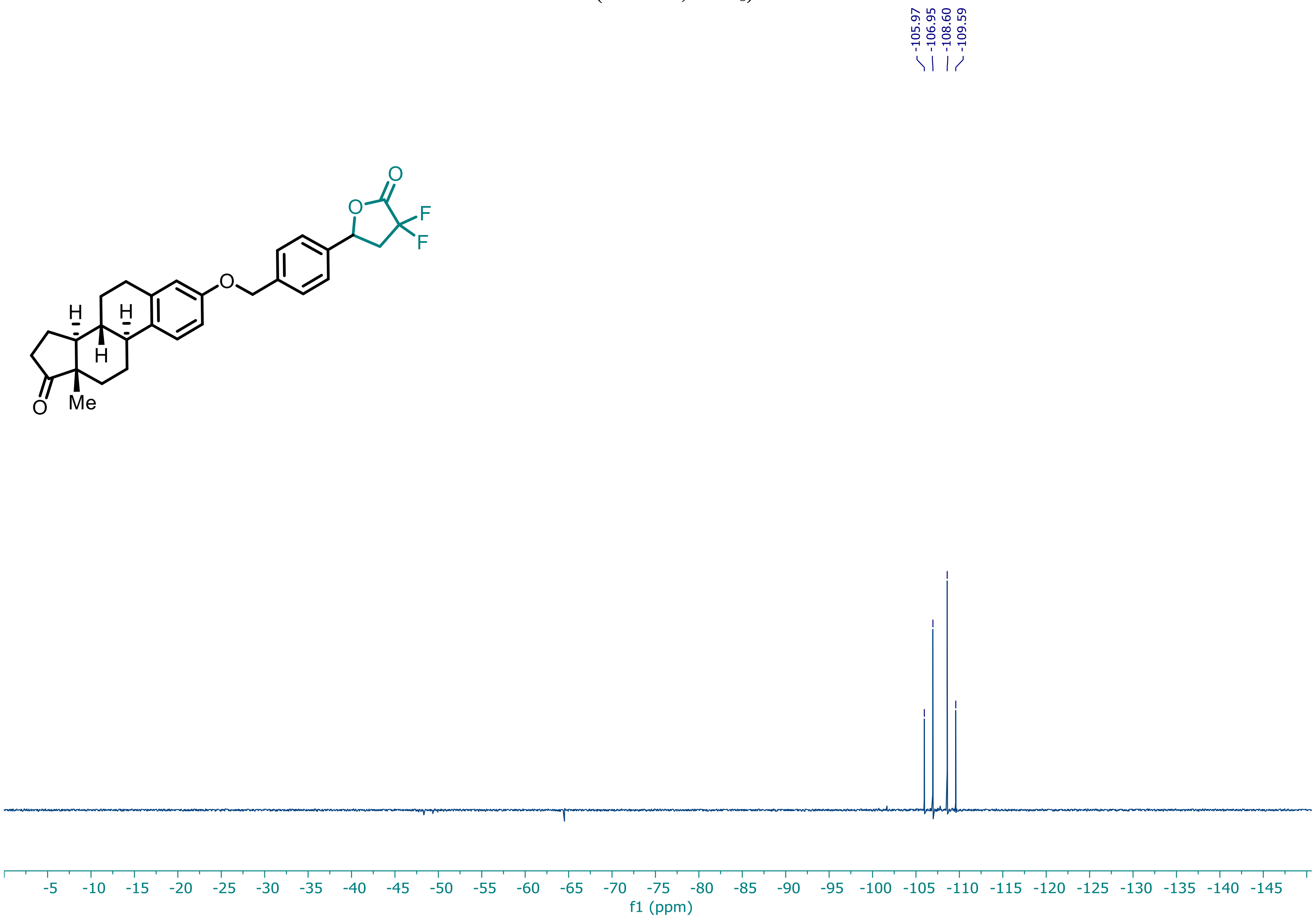

^{1}H NMR (400 MHz, $CDCl_3$) of **13**

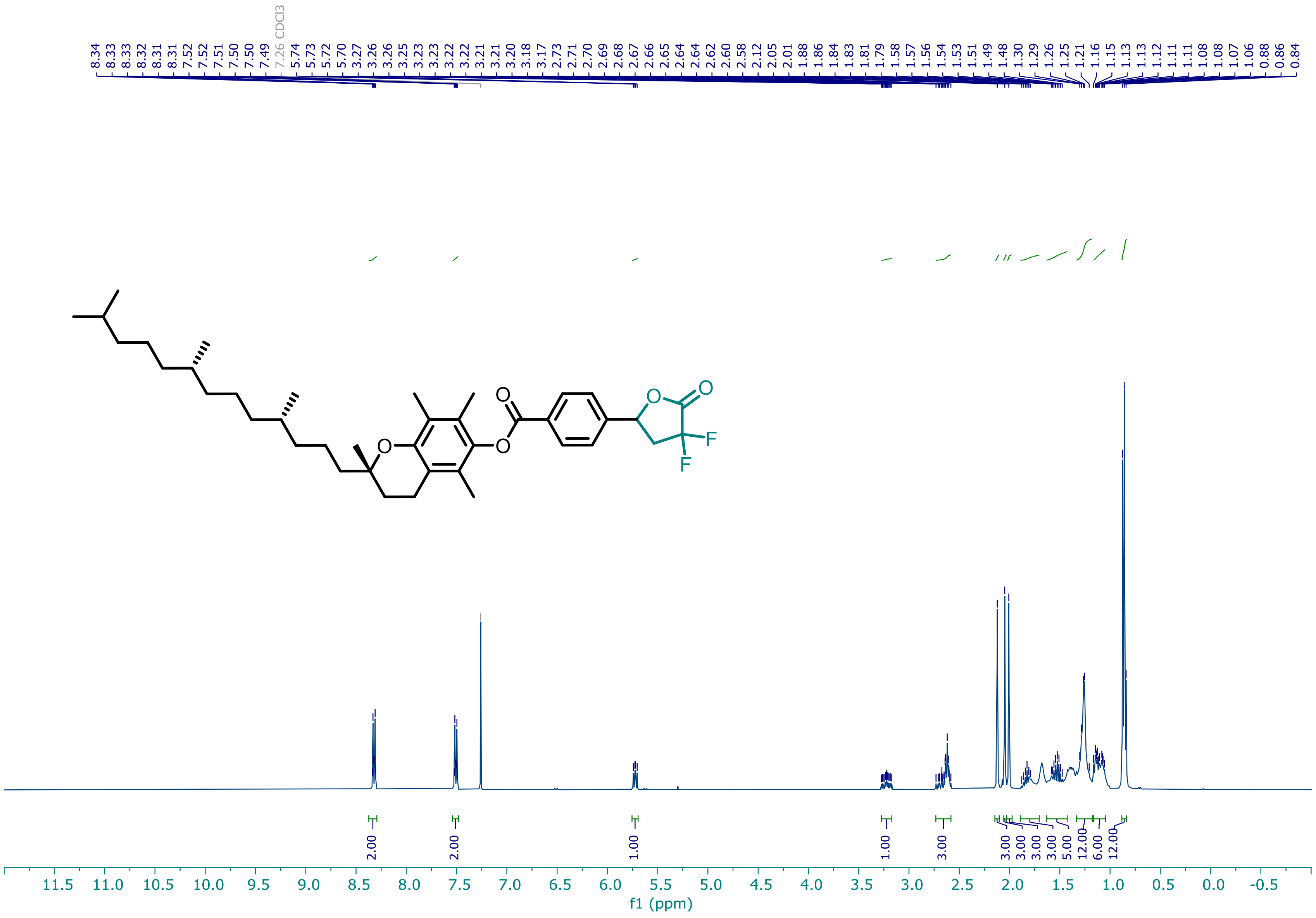

^{13}C NMR (101 MHz, $CDCl_3$) of **13**

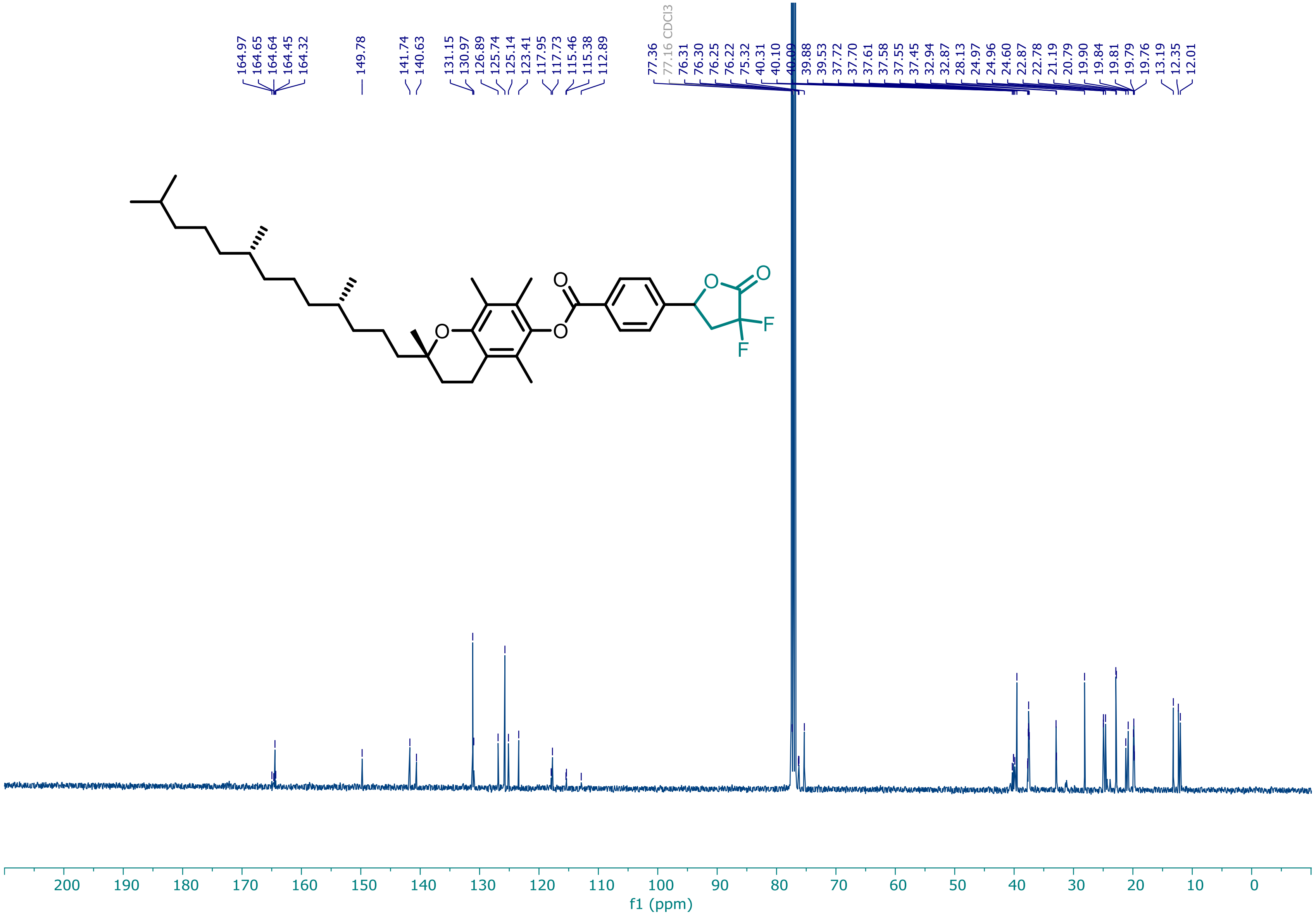

^{19}F NMR (376 MHz, $CDCl_3$) of **13**

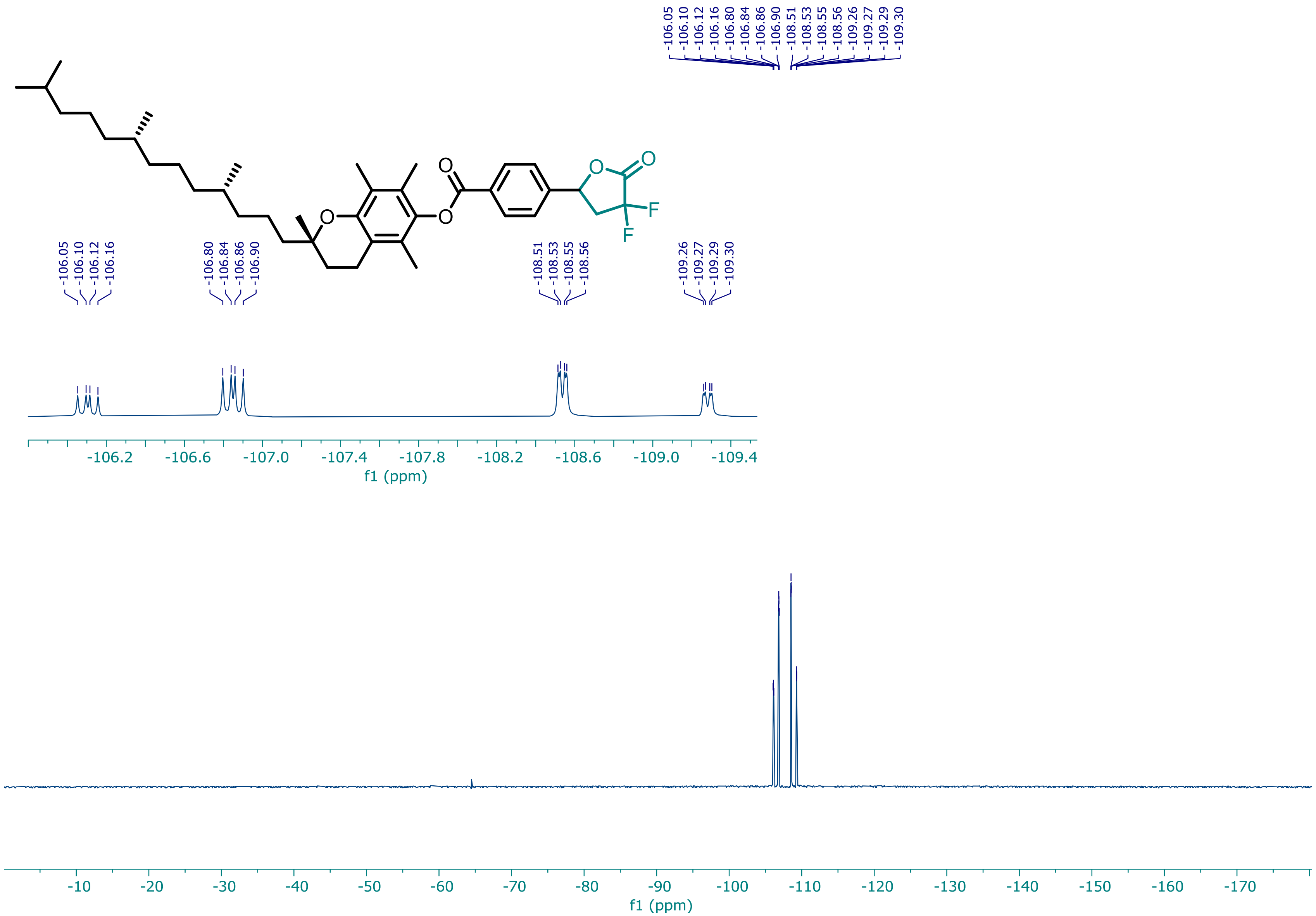

^{1}H NMR (300 MHz, $CDCl_3$) of **14**

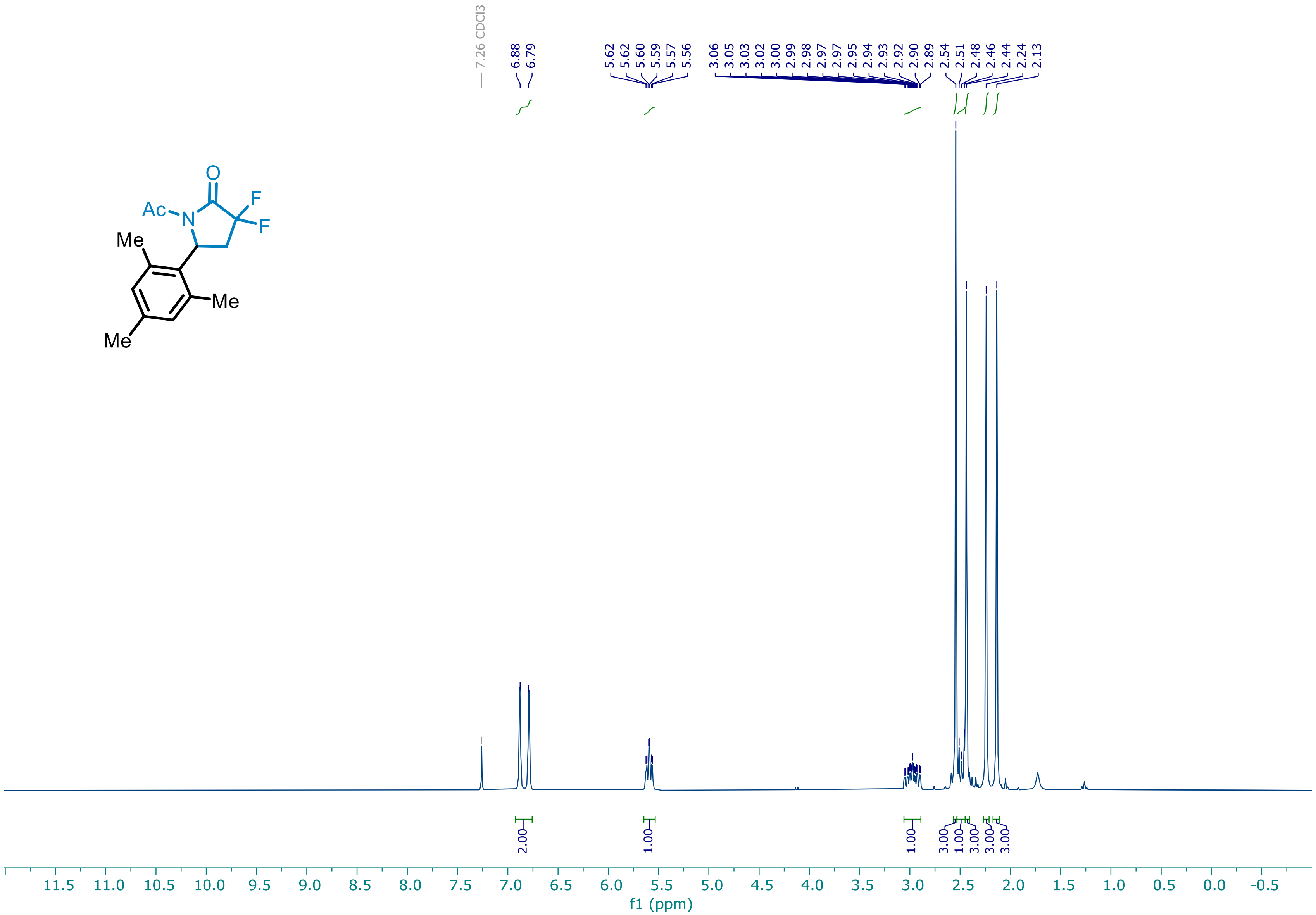

^{13}C NMR (75 MHz, $CDCl_3$) of **14**

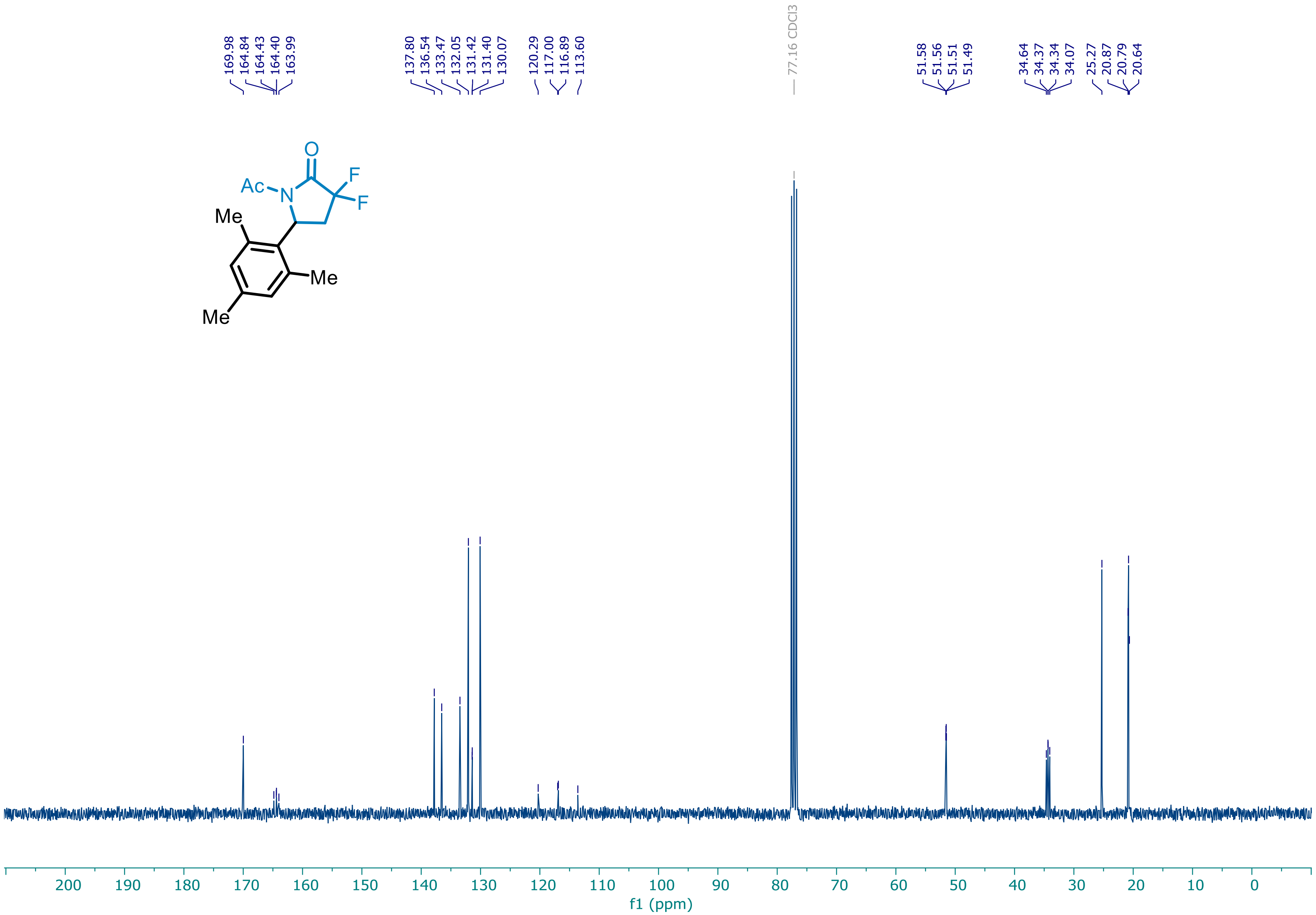

^{19}F NMR (282 MHz, $CDCl_3$) of **14**

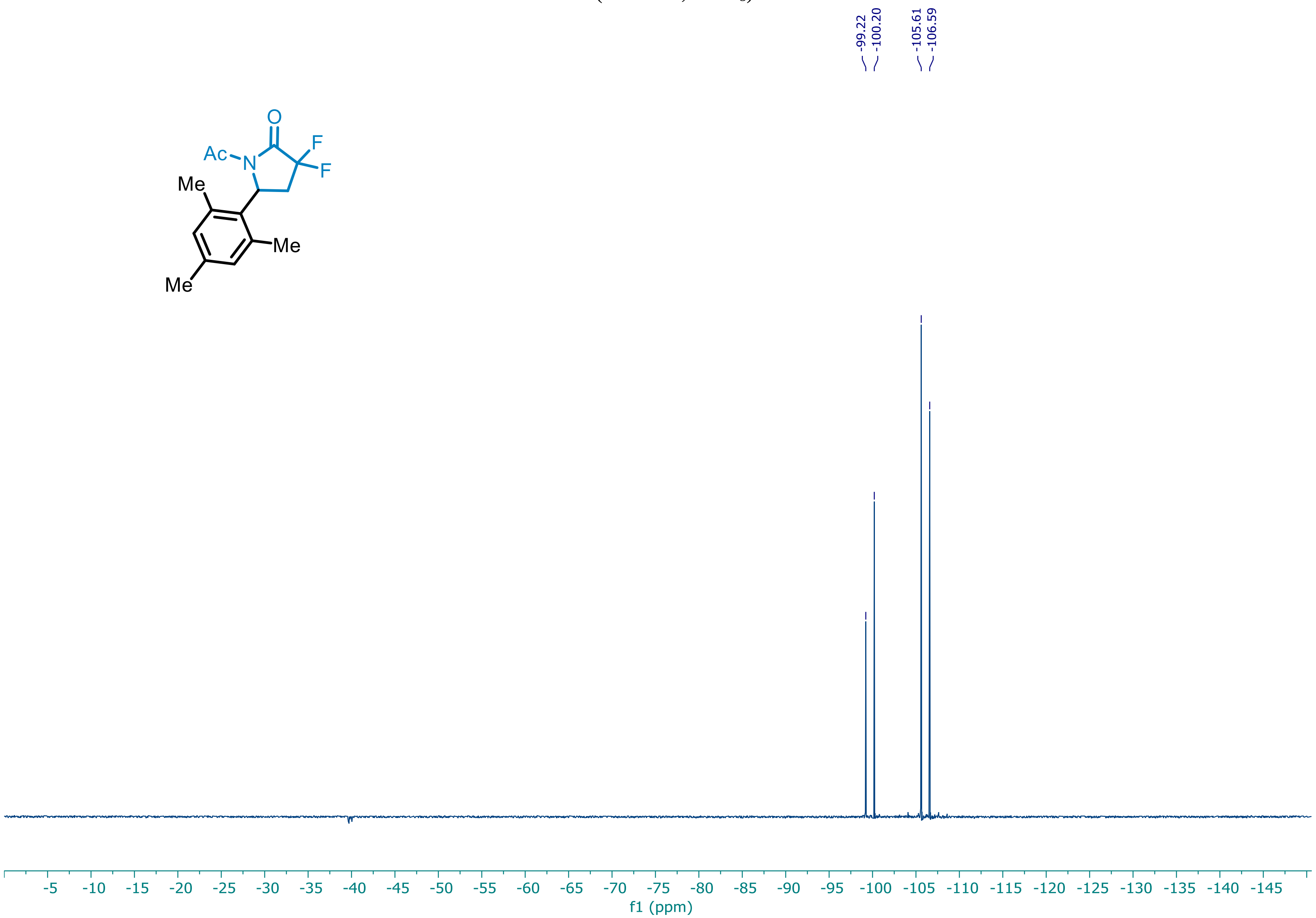

^{1}H NMR (300 MHz, $CDCl_3$) of **15**

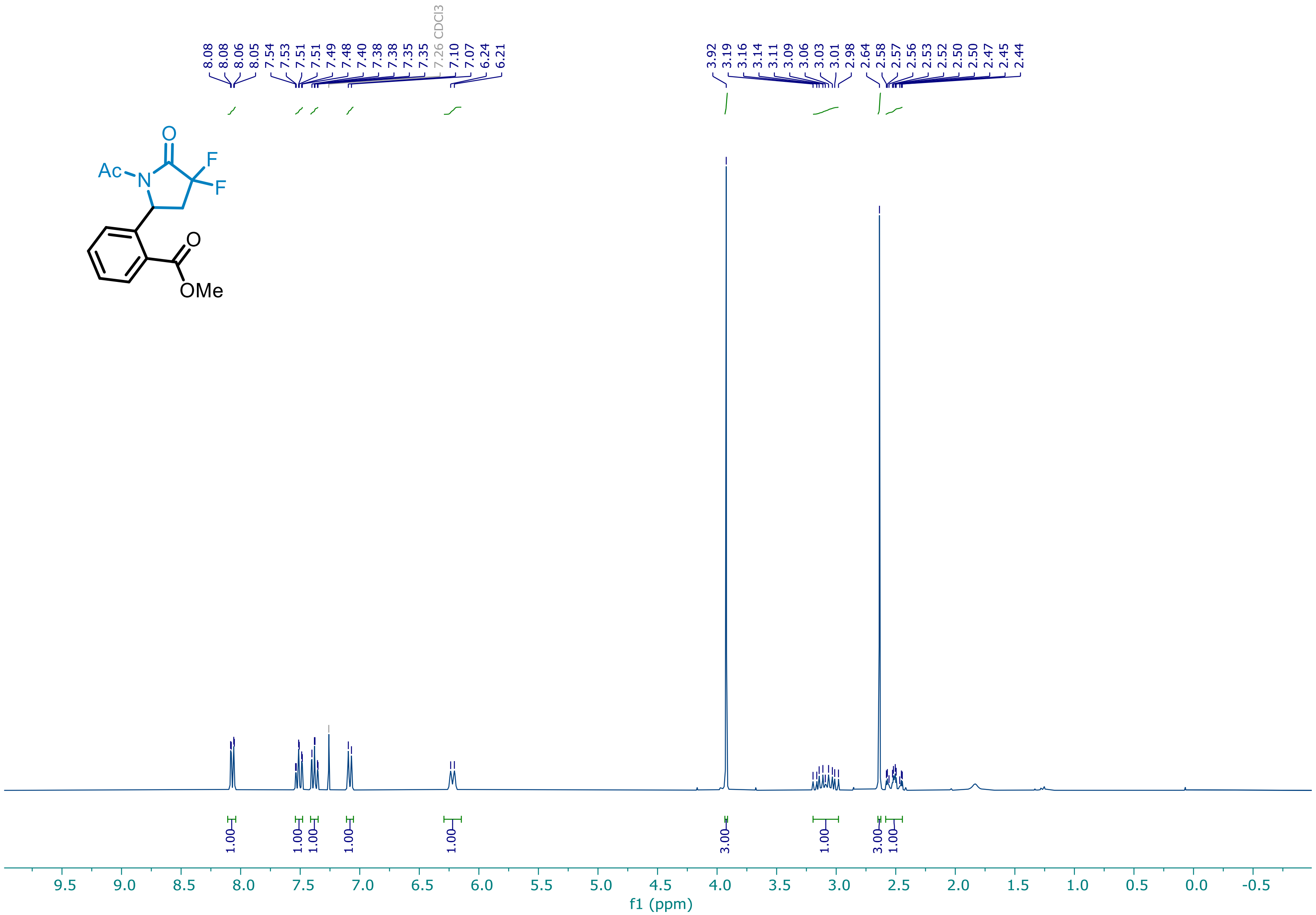

^{13}C NMR (75 MHz, $CDCl_3$) of **15**

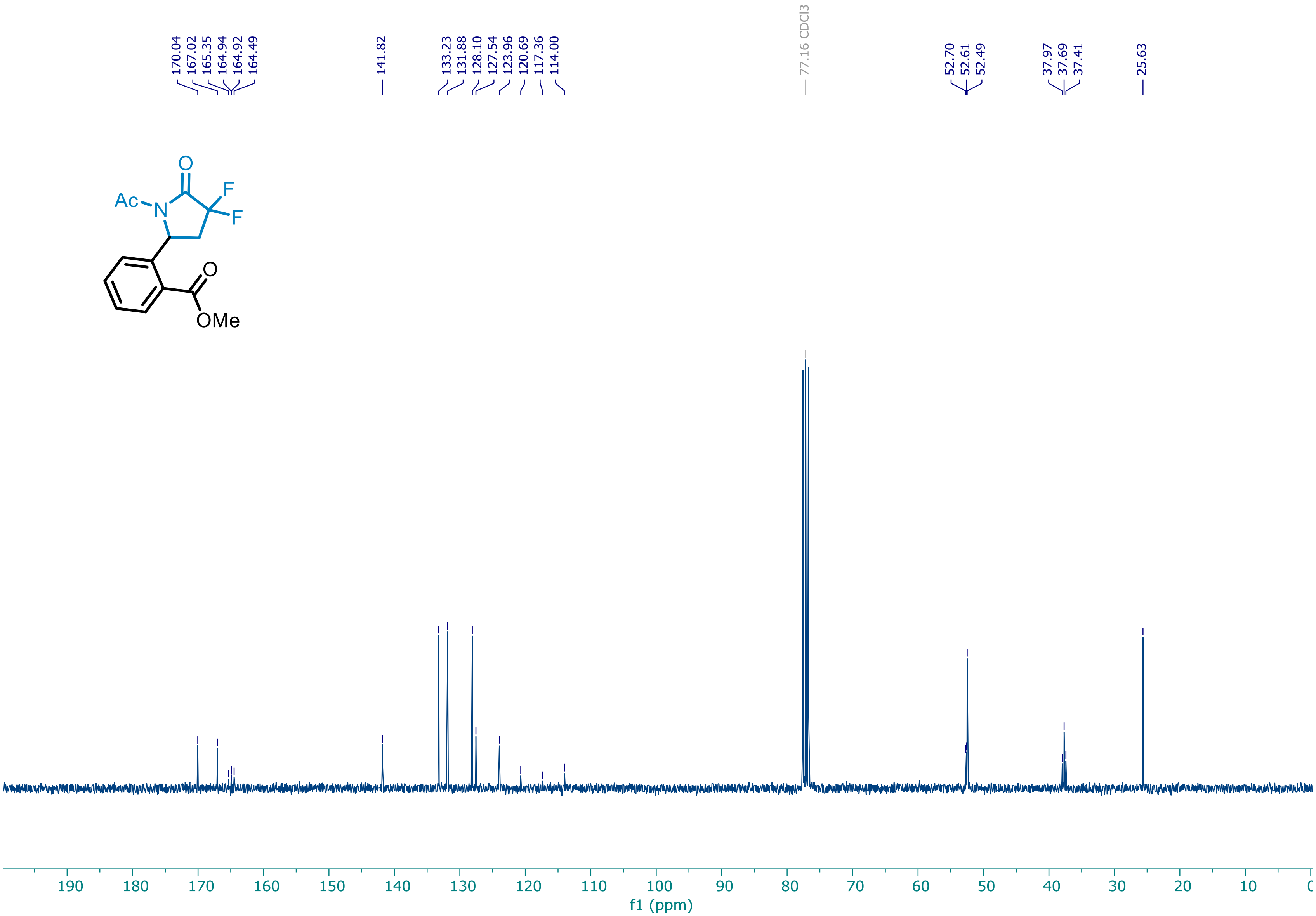

^{19}F NMR (282 MHz, $CDCl_3$) of **15**

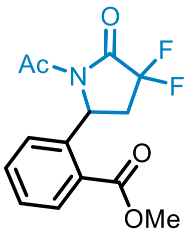

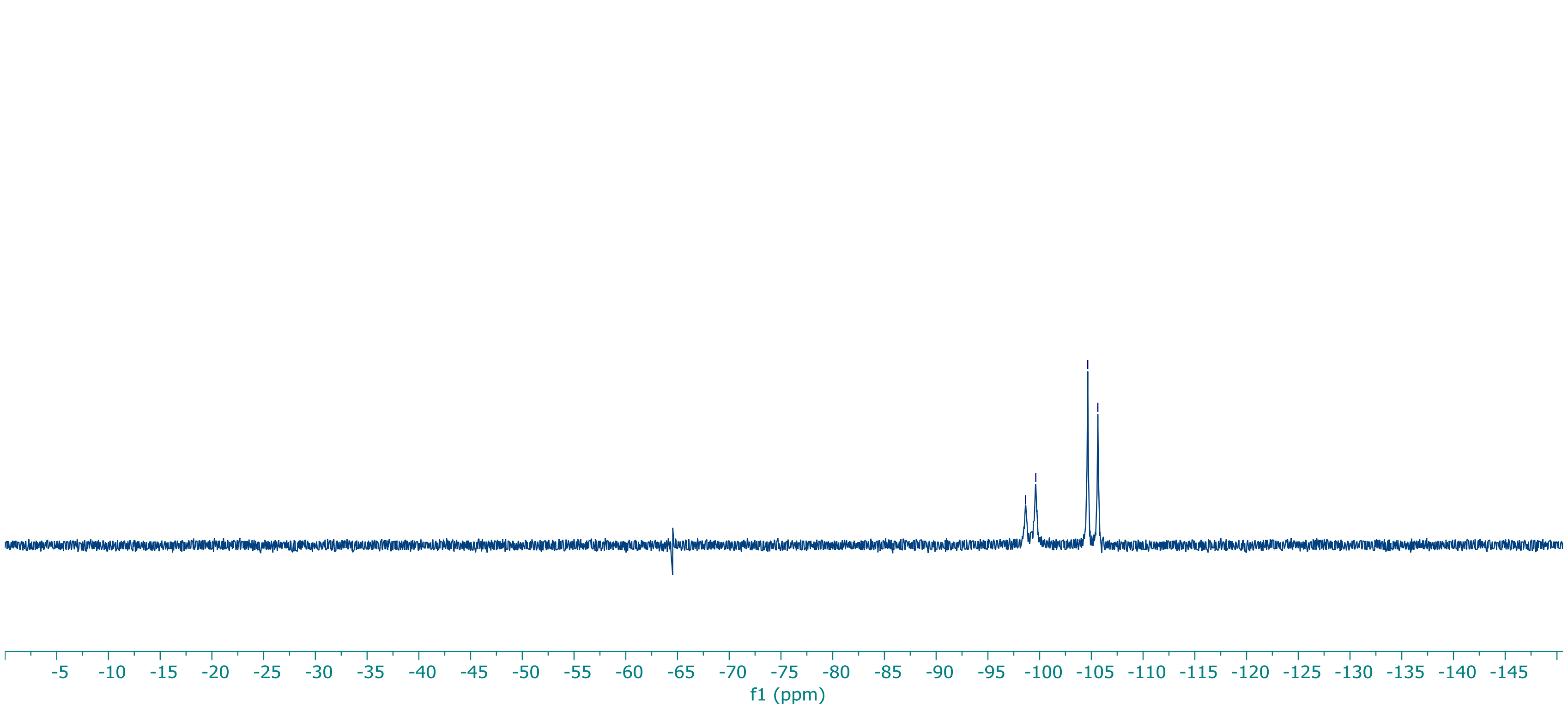

^{1}H NMR (400 MHz, $CDCl_3$) of **16**

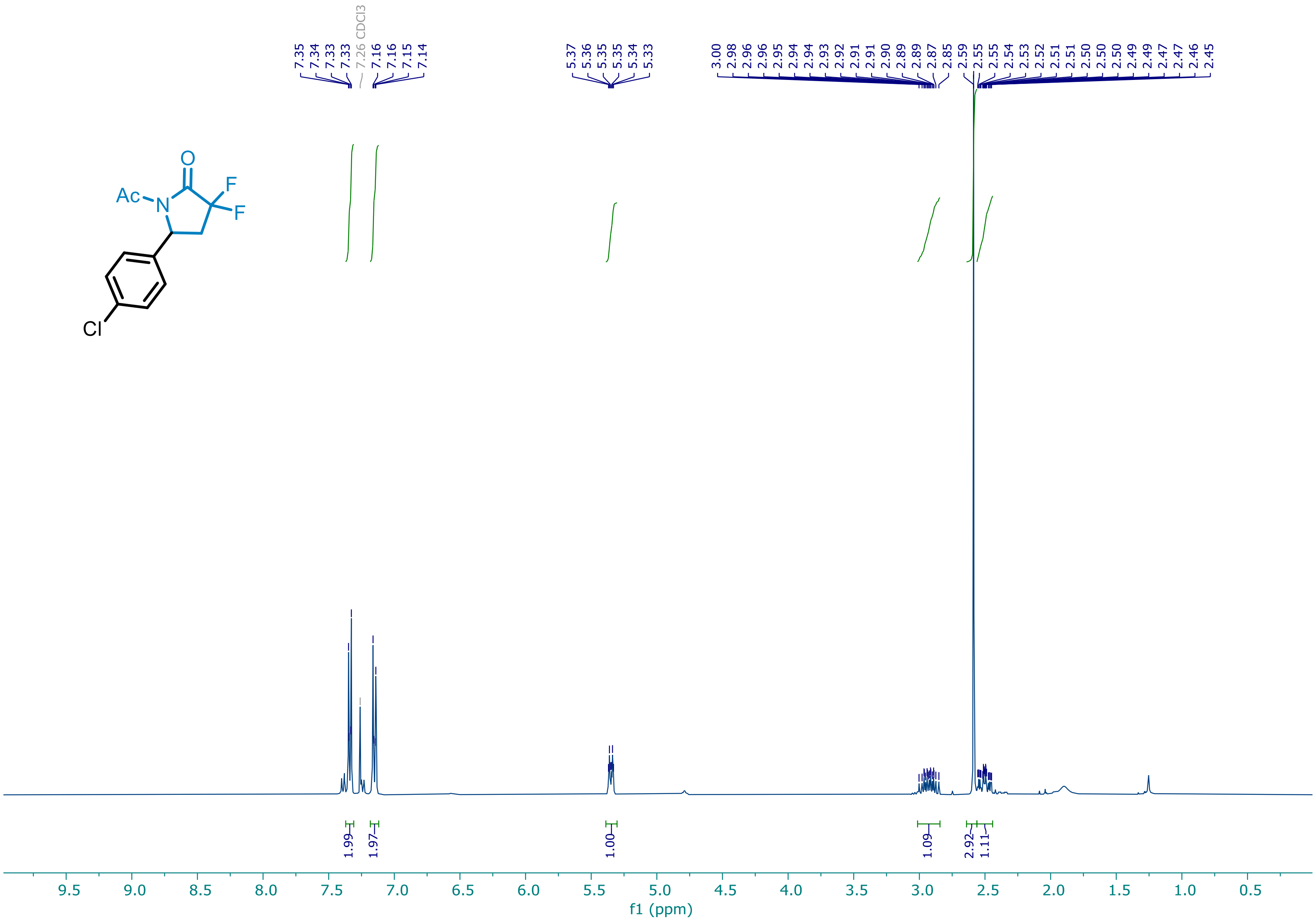

^{13}C NMR (101 MHz, $CDCl_3$) of **16**

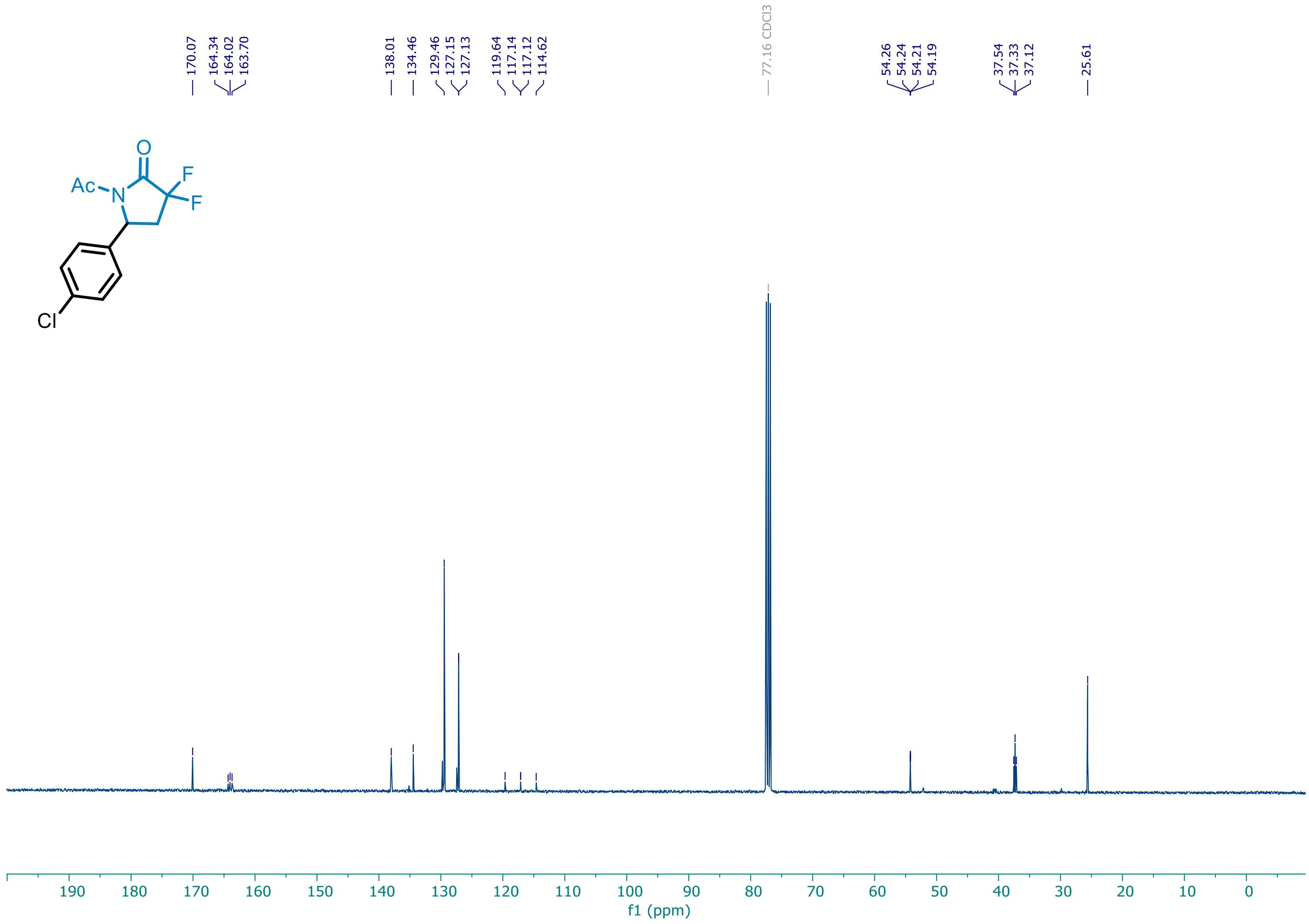

^{19}F NMR (471 MHz, $CDCl_3$) of **16**

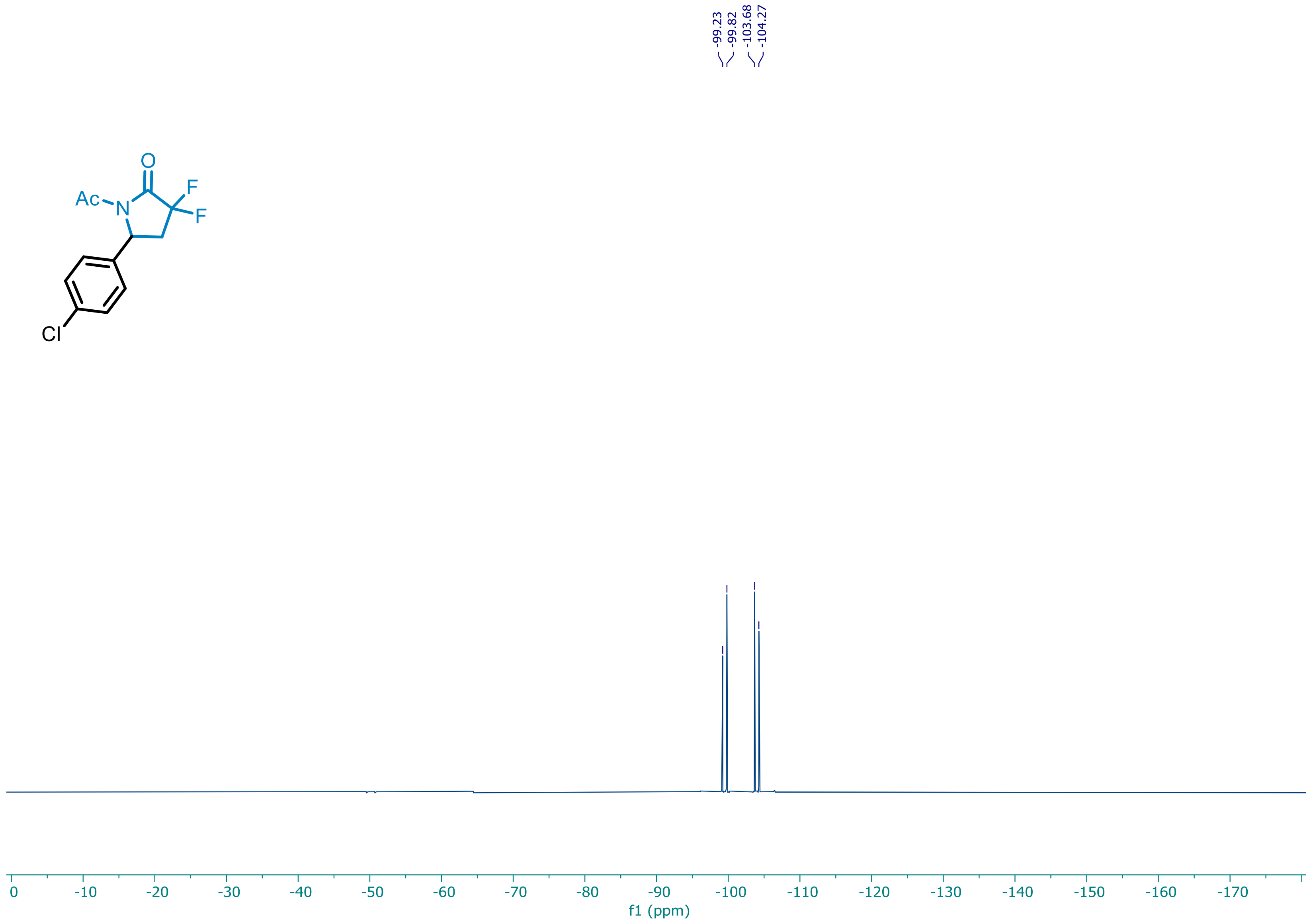

^{1}H NMR (500 MHz, $CDCl_3$) of **17**

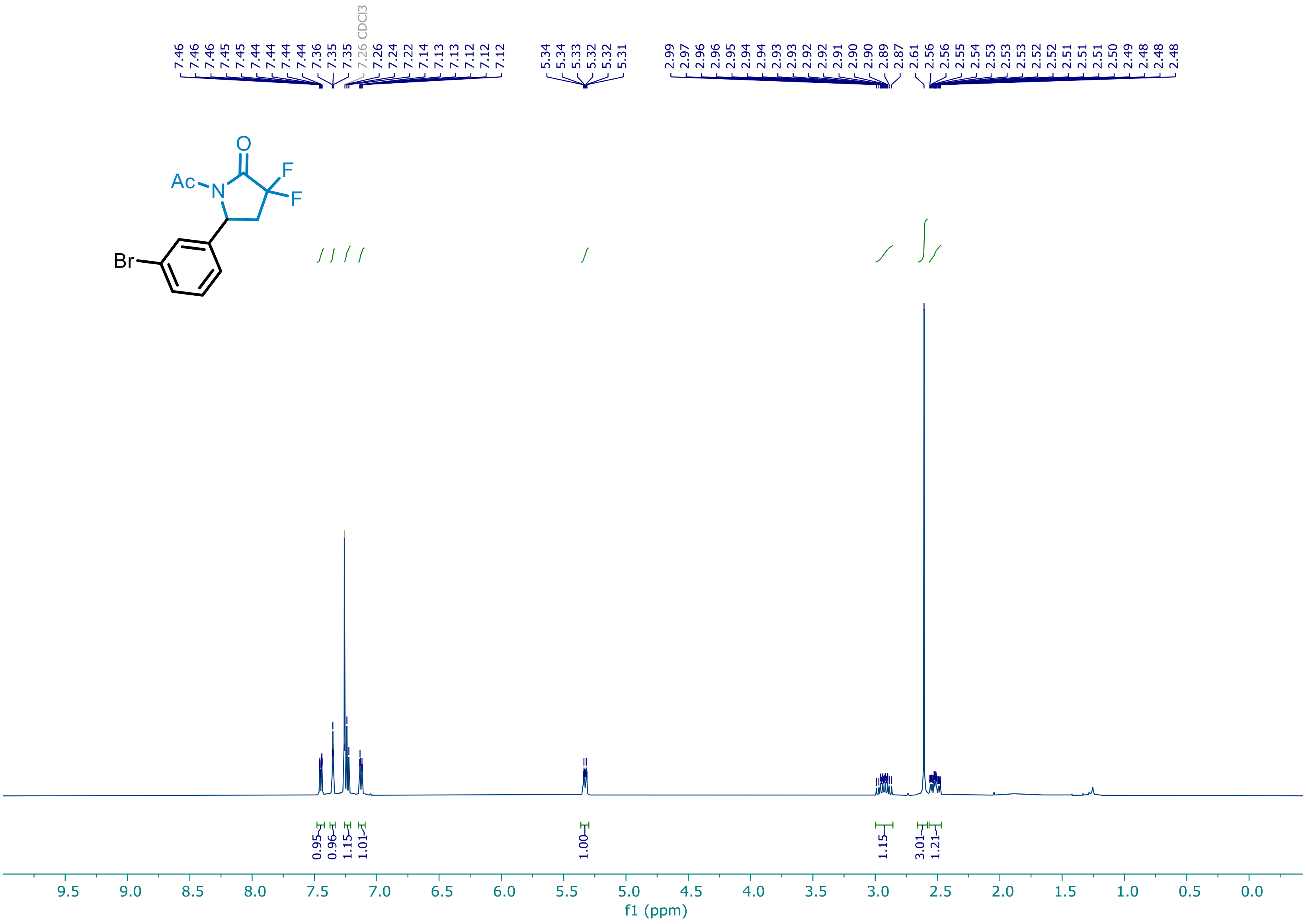

^{13}C NMR (126 MHz, $CDCl_3$) of **17**

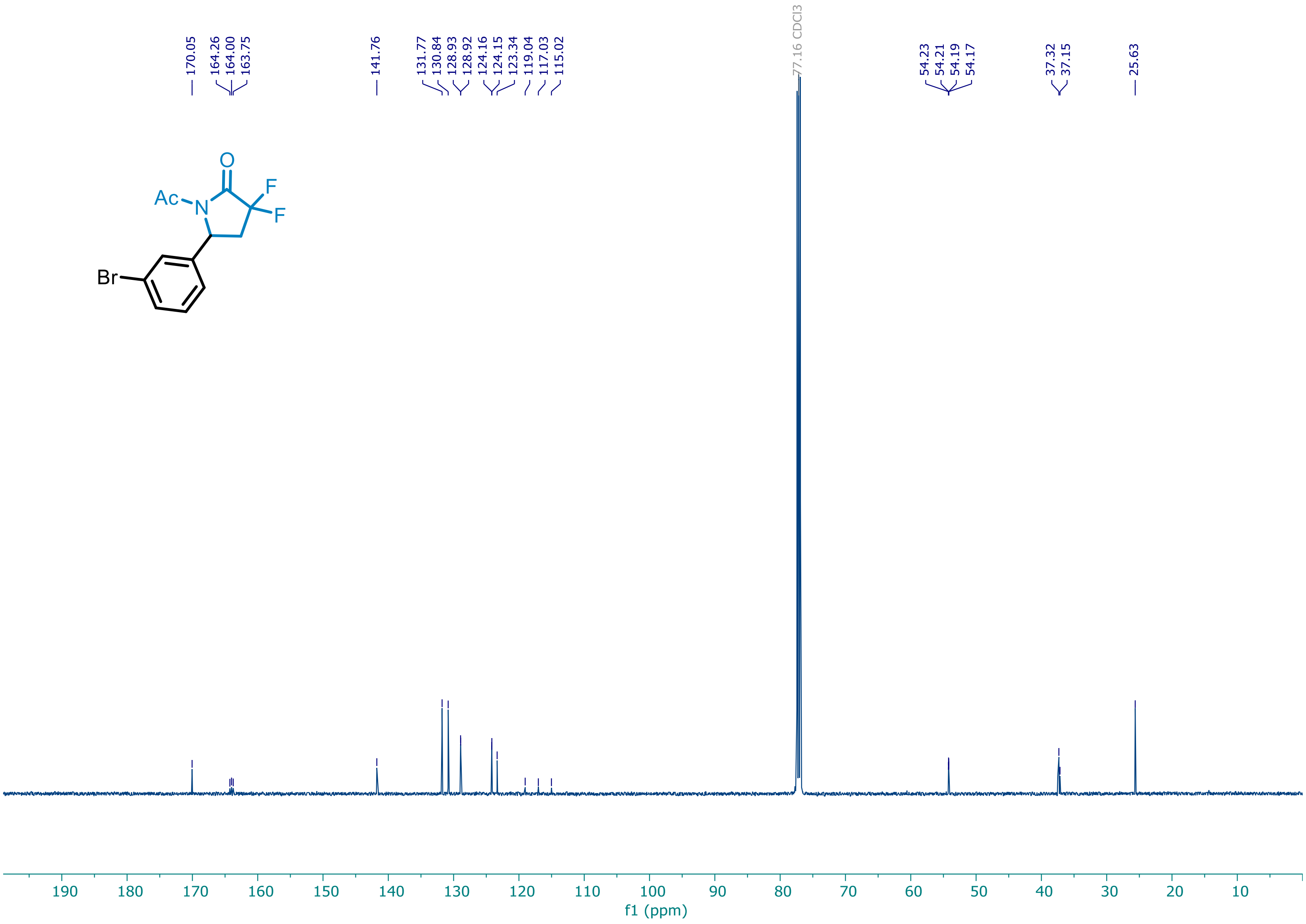

^{19}F NMR (471 MHz, $CDCl_3$) of **17**

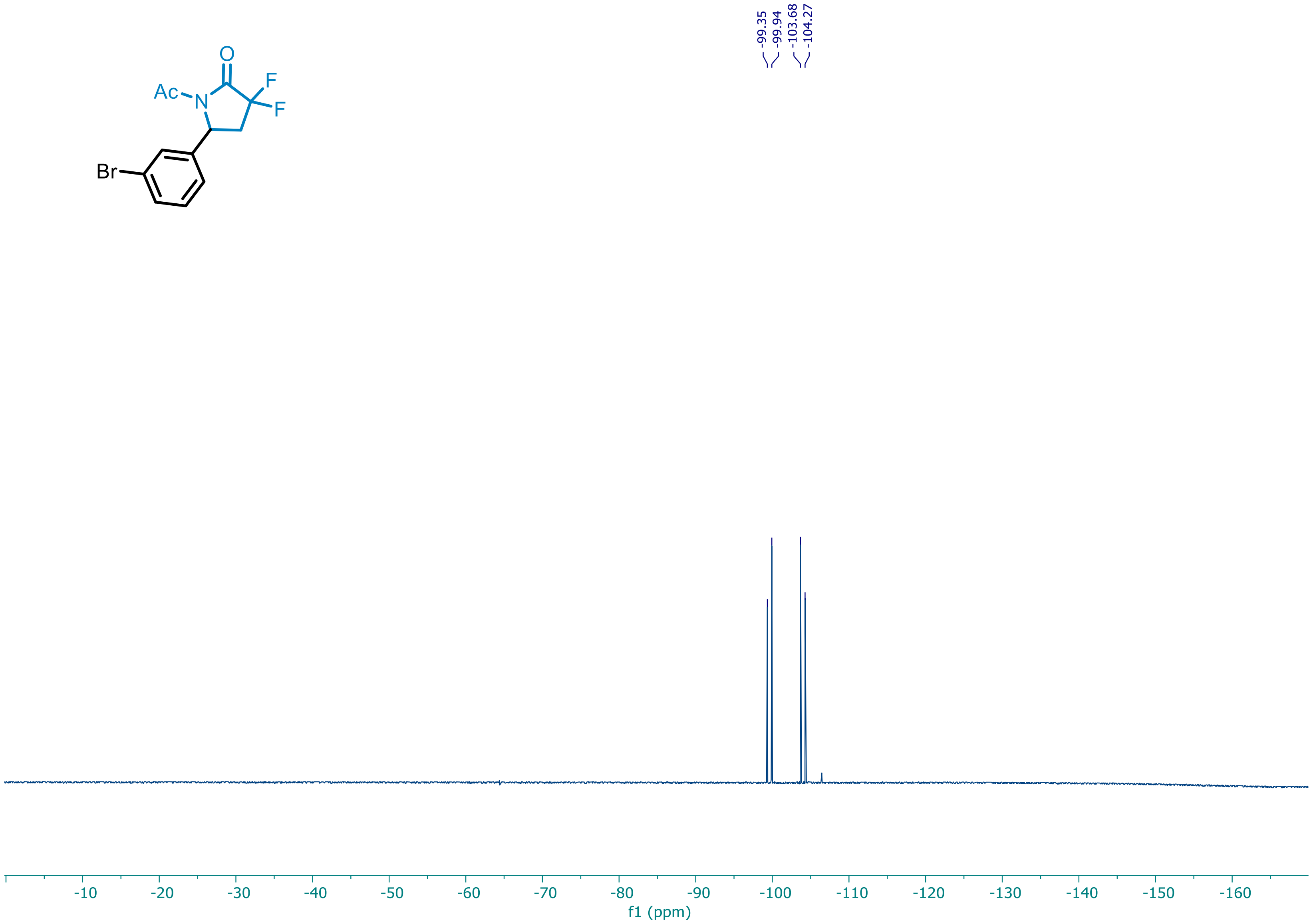

^{1}H NMR (500 MHz, $CDCl_3$) of **18**

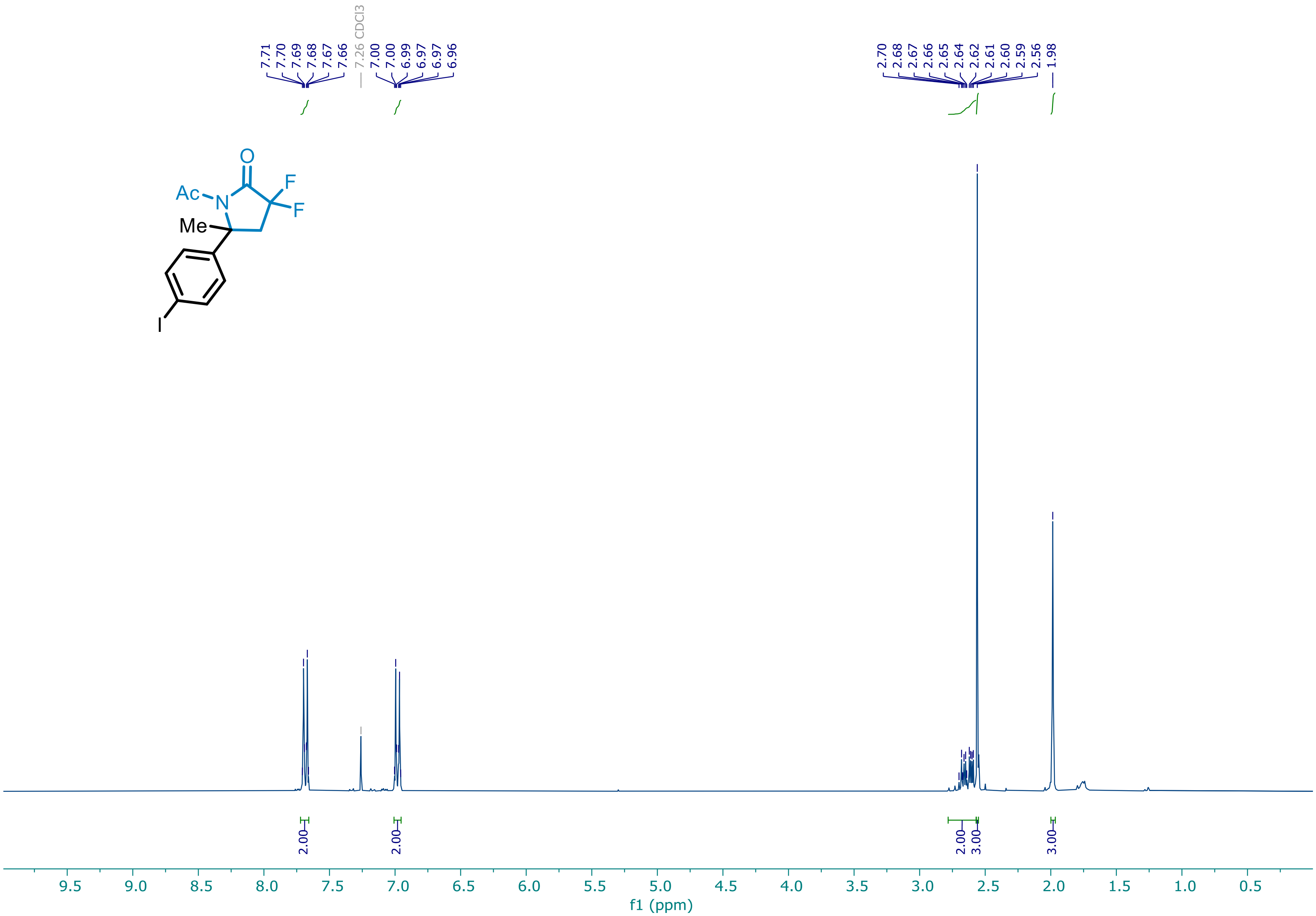

^{13}C NMR (75 MHz, $CDCl_3$) of **18**

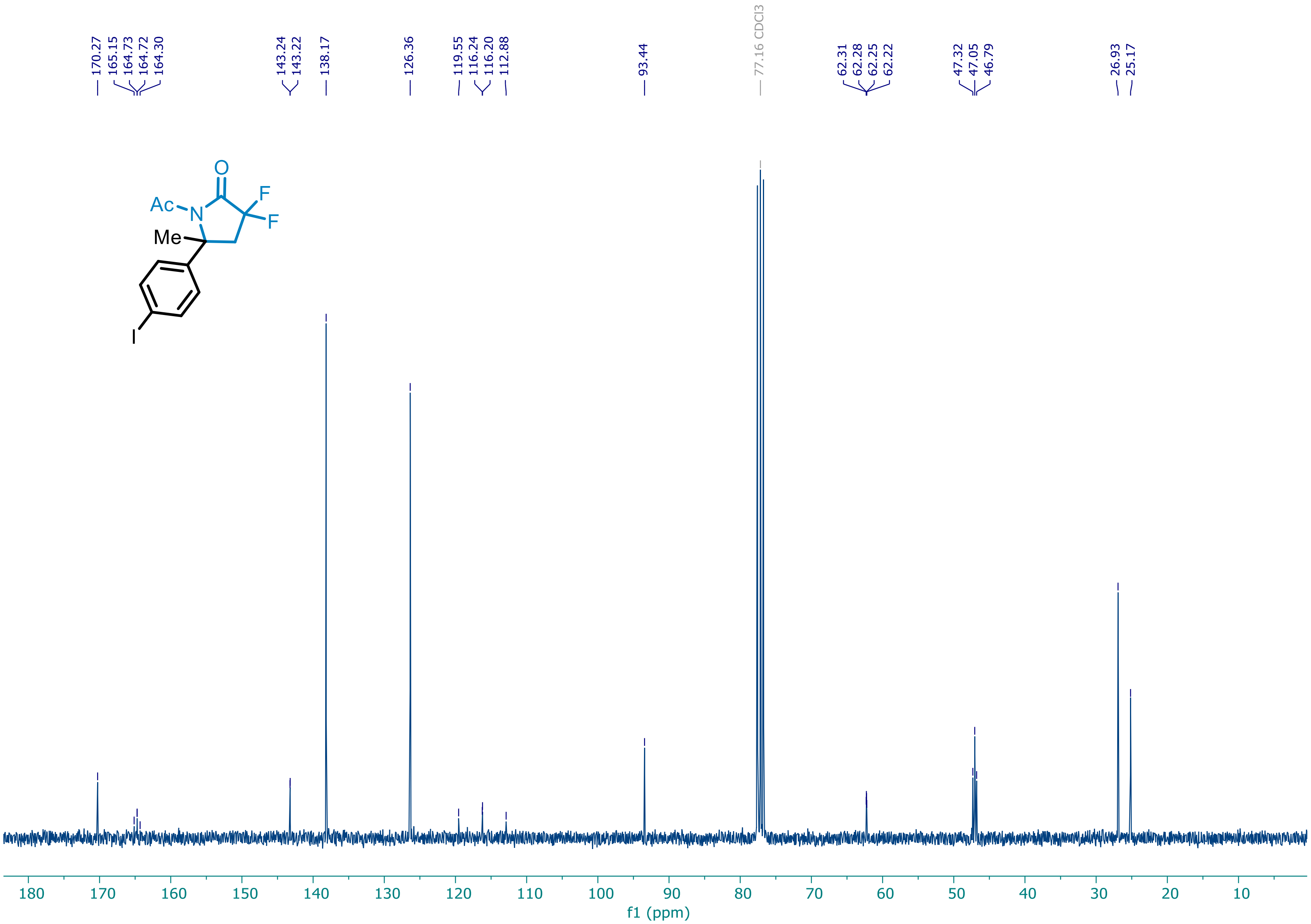

^{19}F NMR (282 MHz, $CDCl_3$) of **18**

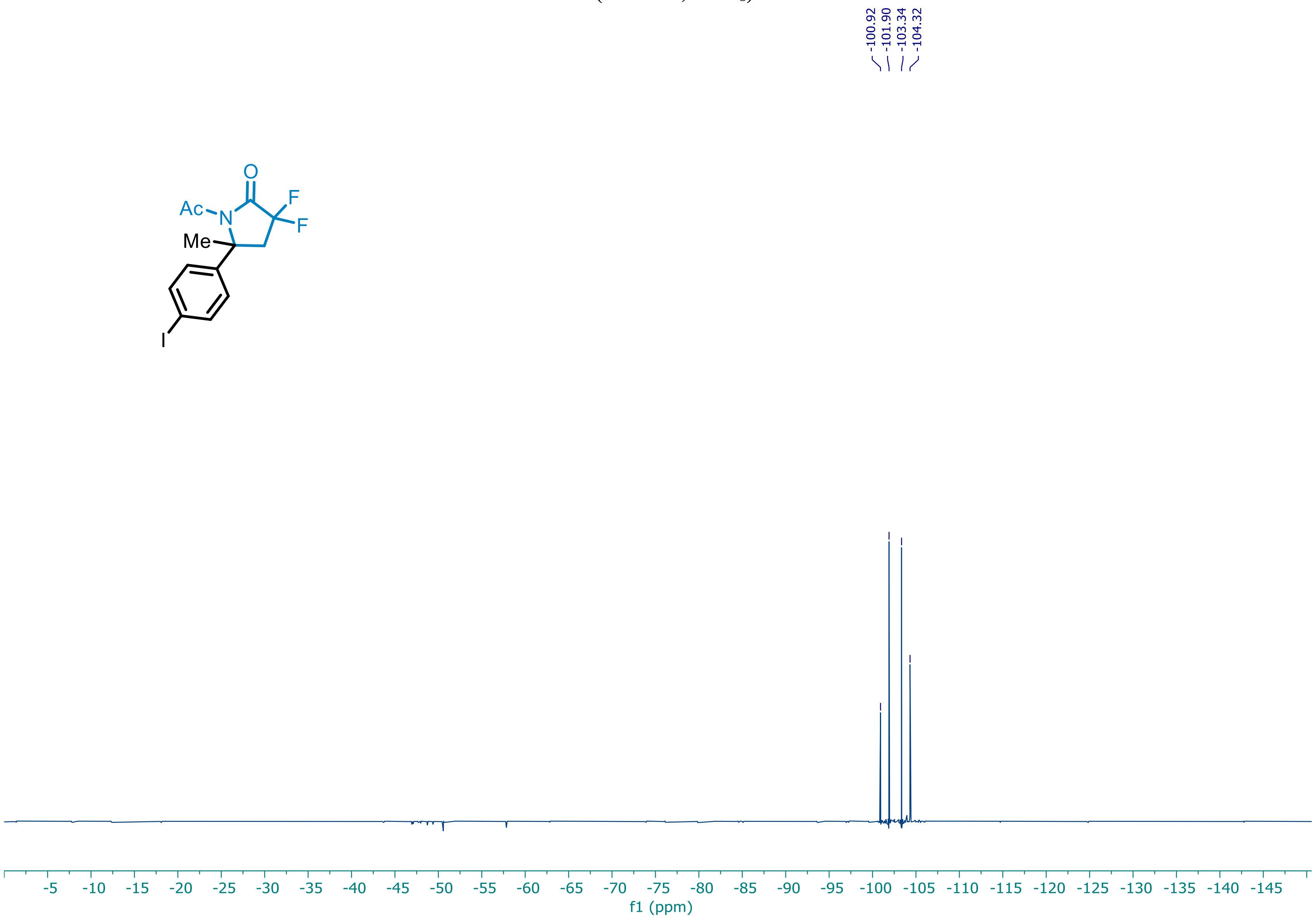

^{1}H NMR (300 MHz, $CDCl_3$) of **19**

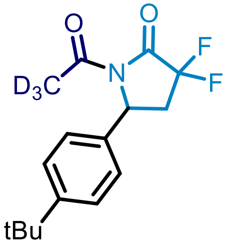

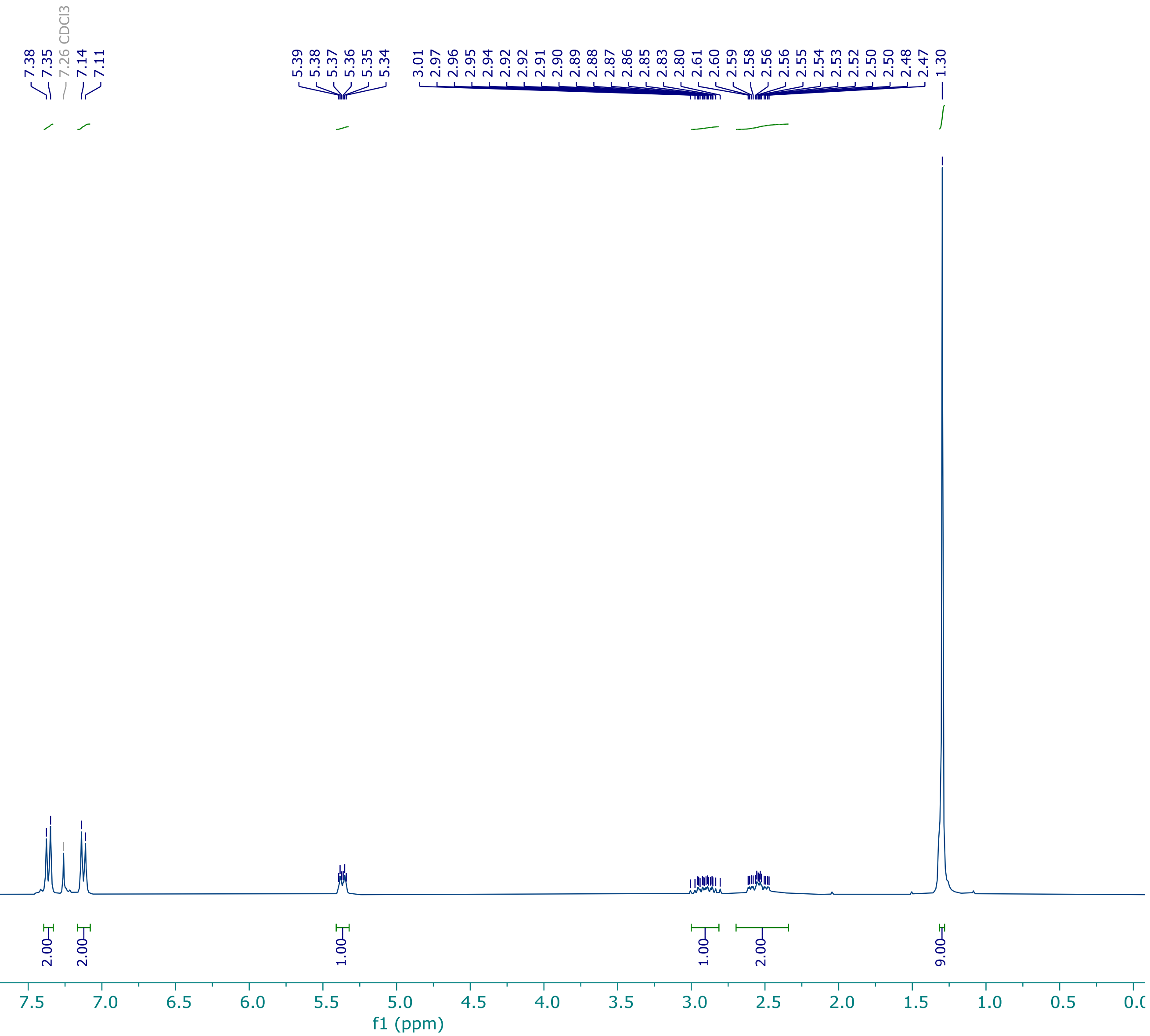

^{13}C NMR (75 MHz, $CDCl_3$) of **19**

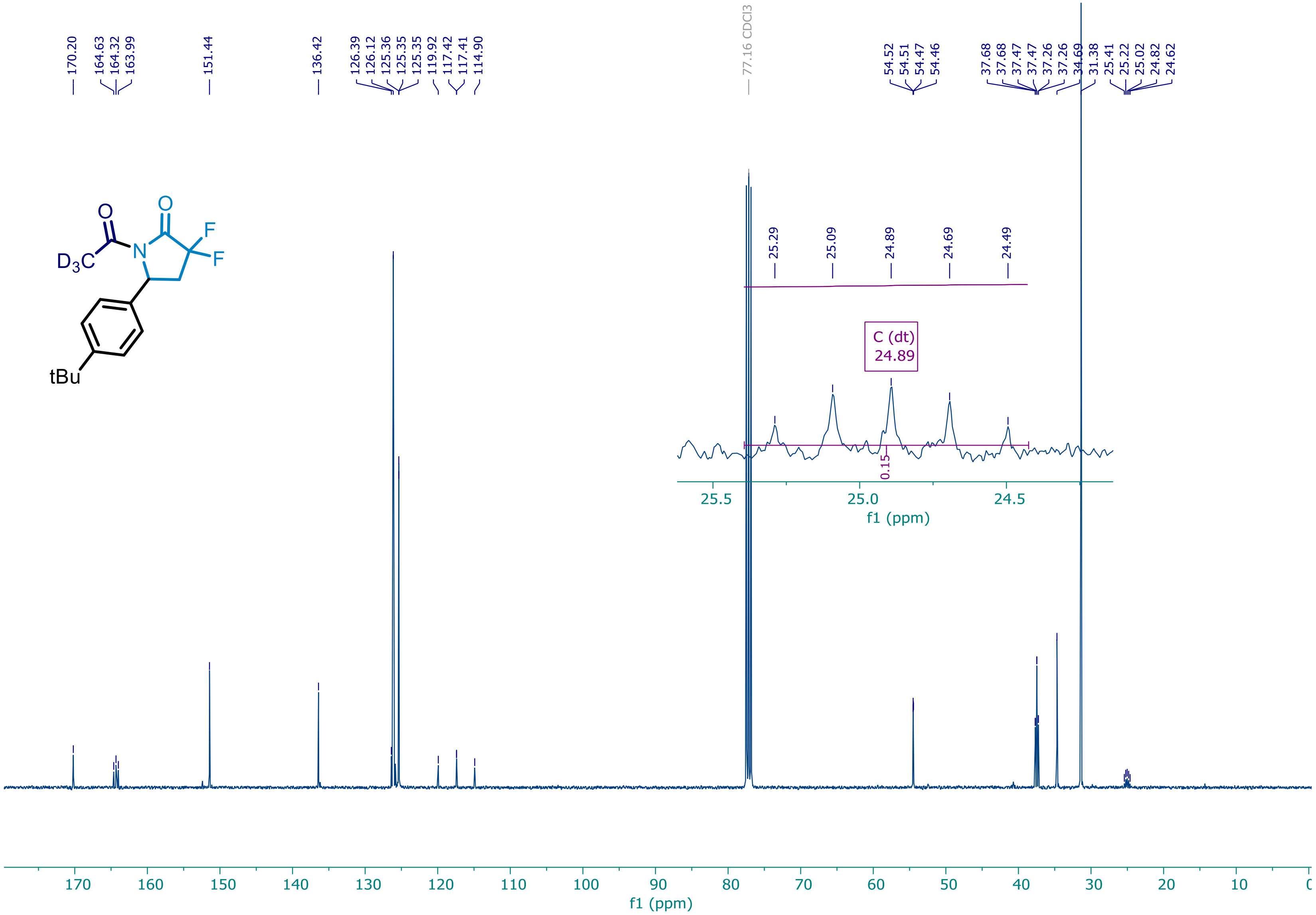

^{19}F NMR (282 MHz, $CDCl_3$) of **19**

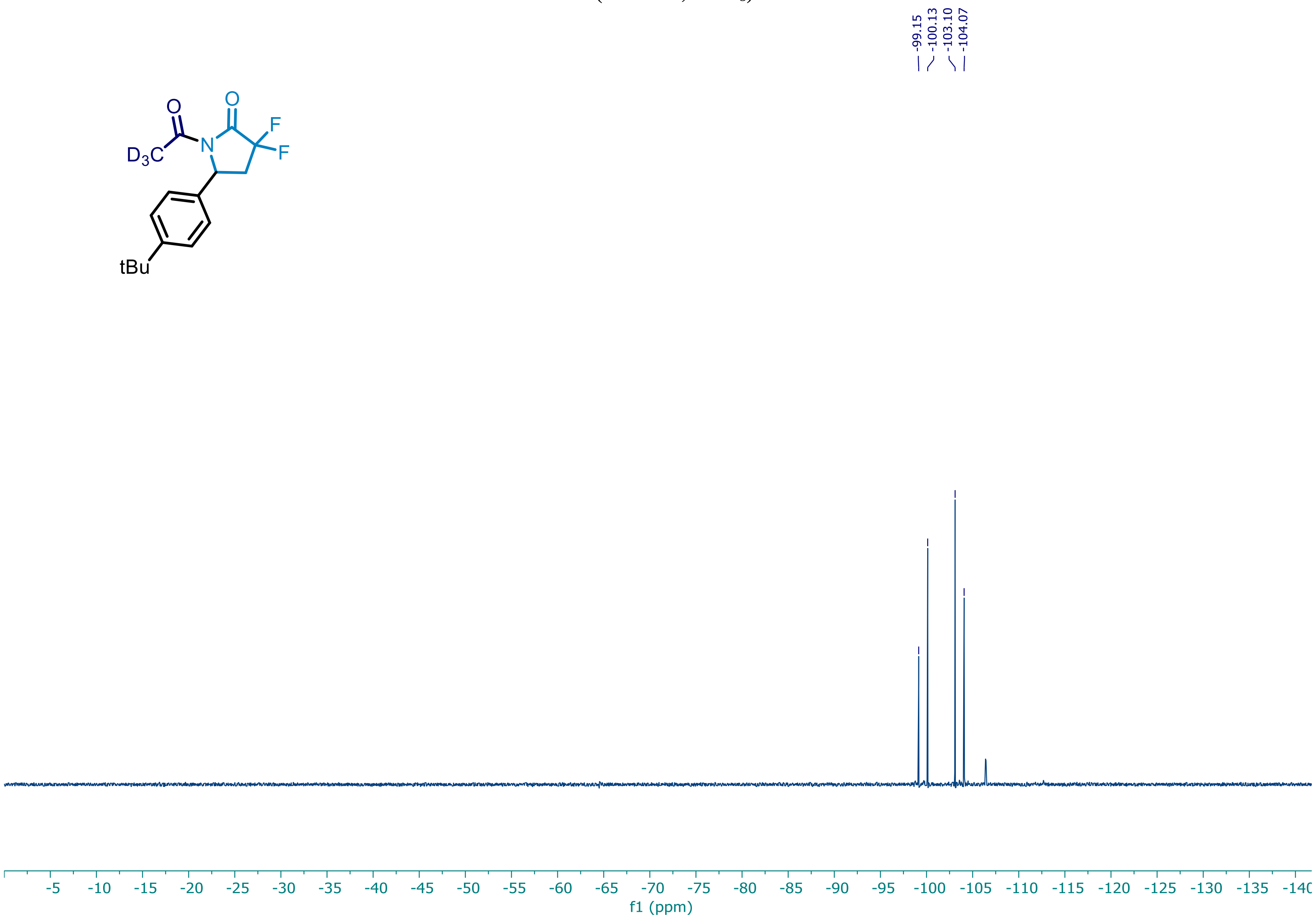

^{1}H NMR (400 MHz, $CDCl_3$) of **20**

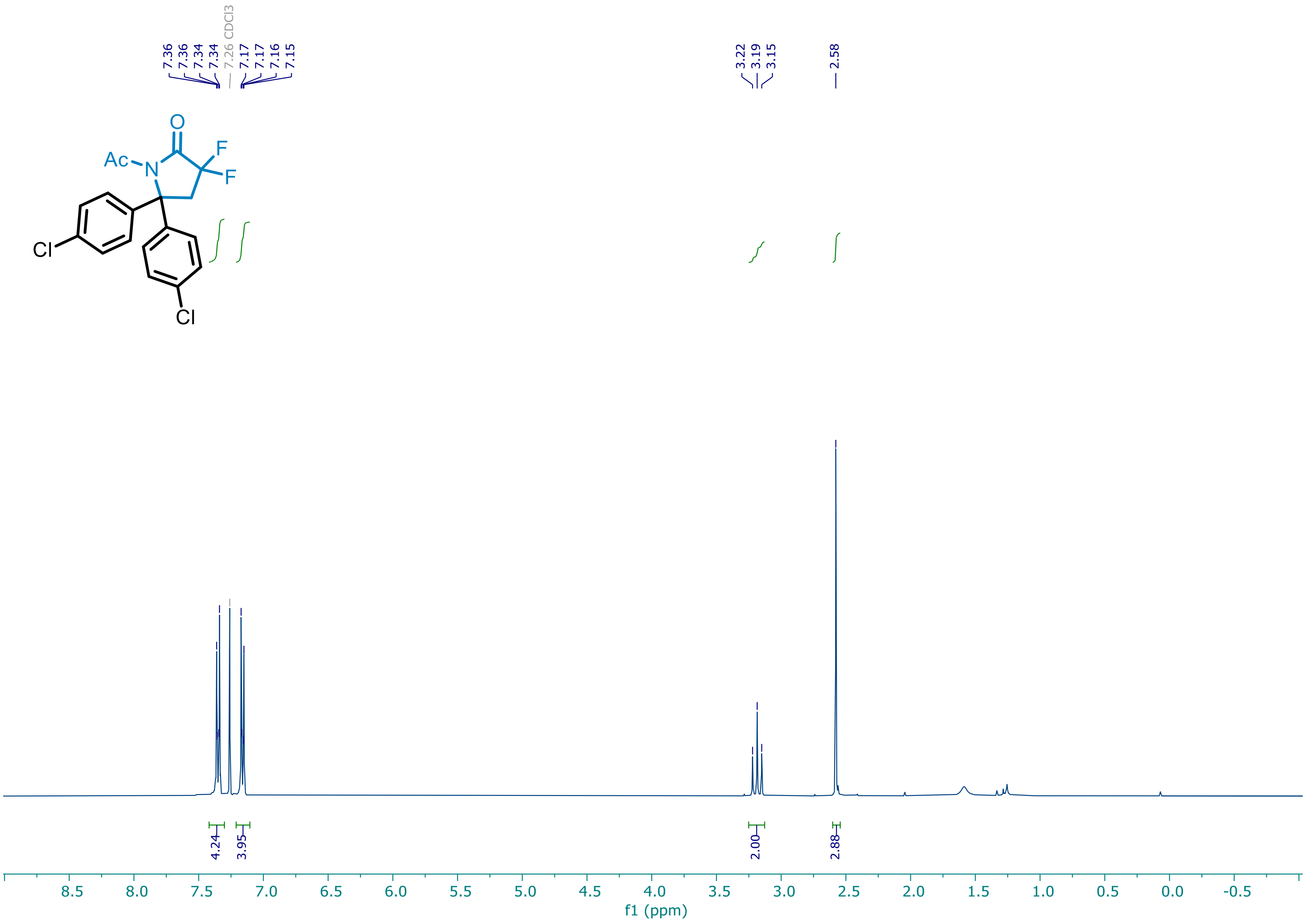

^{13}C NMR (101 MHz, CDCl$_3$) of **20**

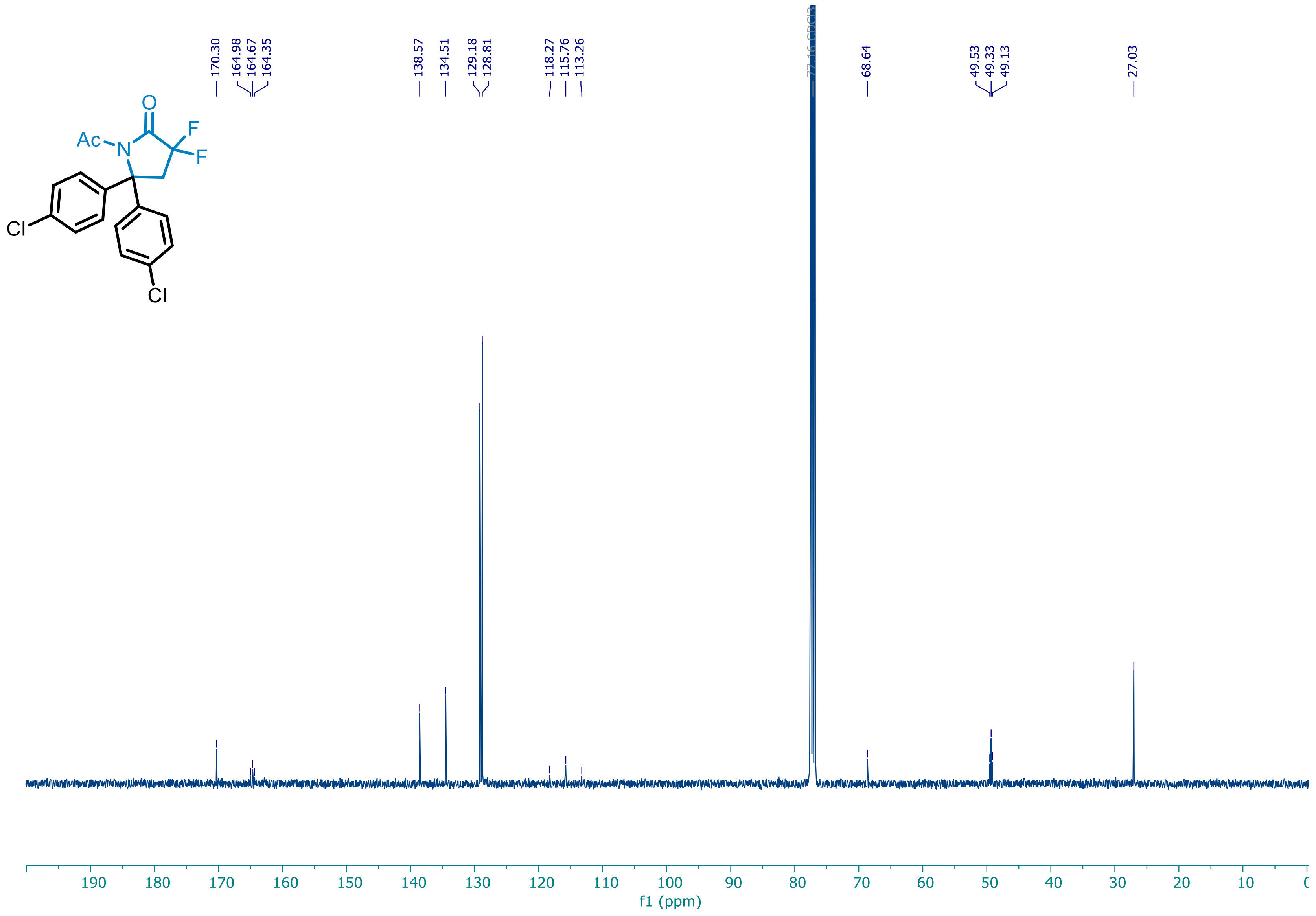

^{19}F NMR (376 MHz, $CDCl_3$) of **20**

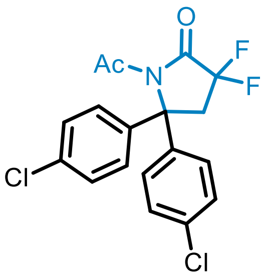

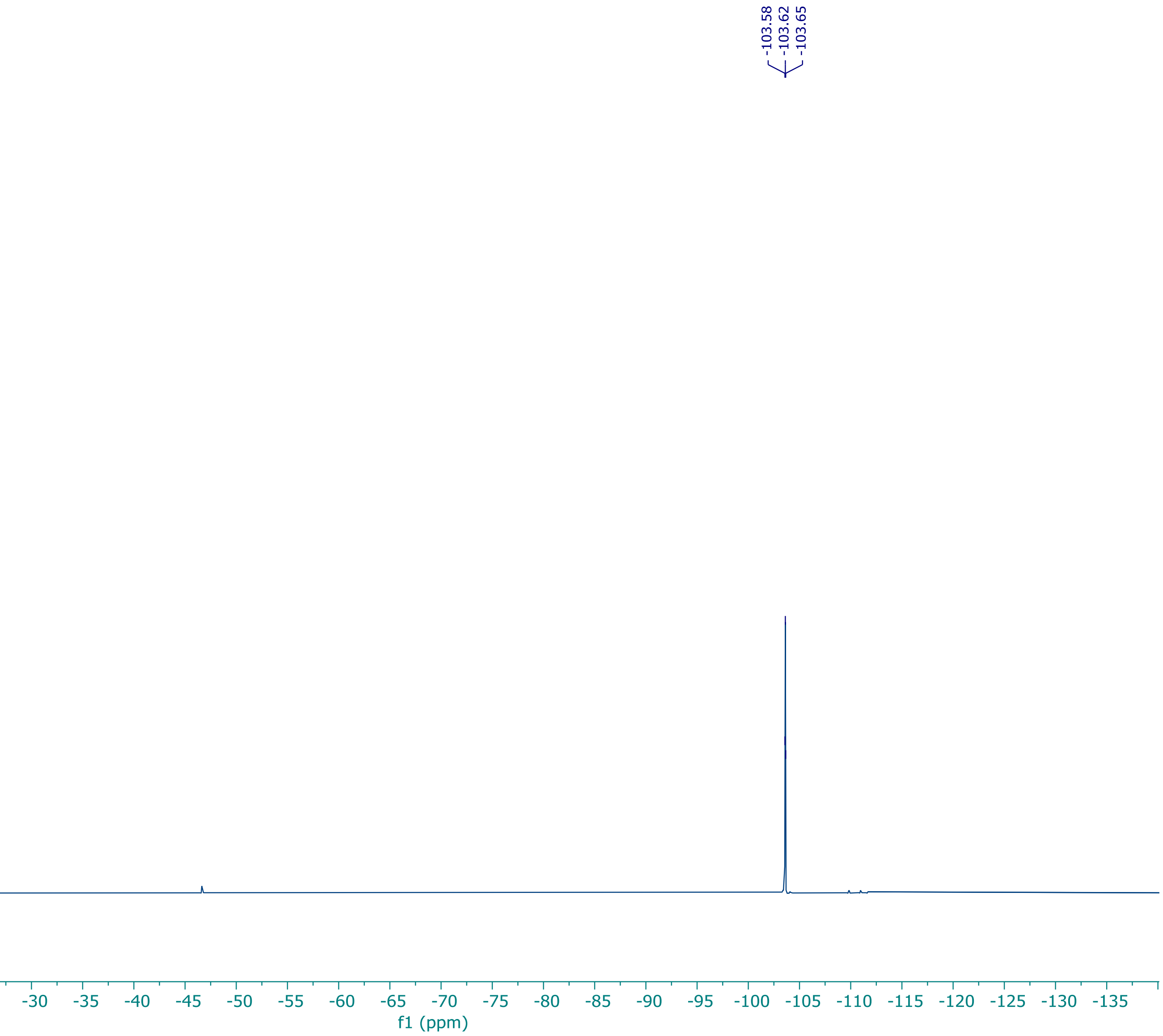

^1H NMR (400 MHz, $CDCl_3$) of **21**

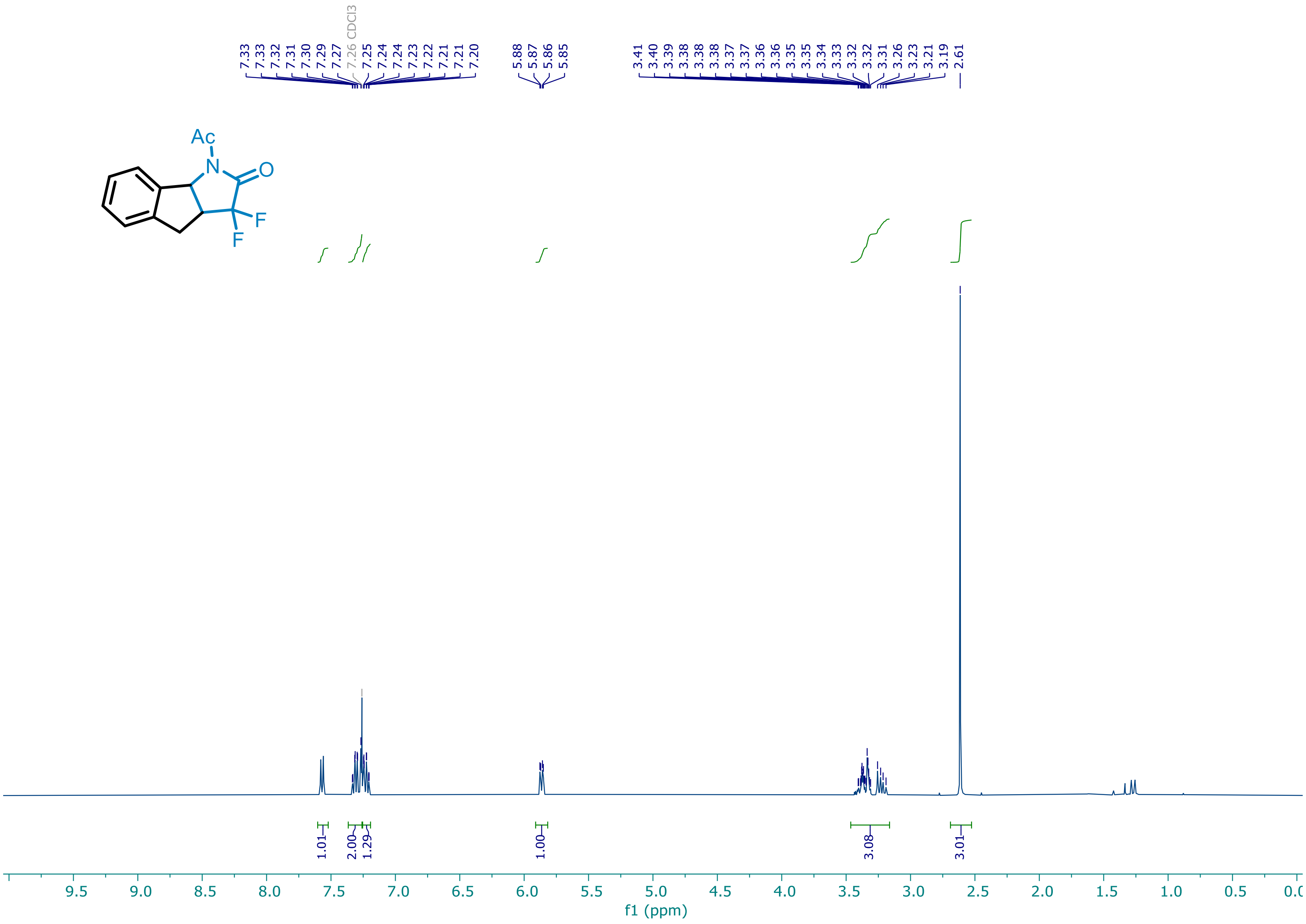

^{13}C NMR (101 MHz, $CDCl_3$) of **21**

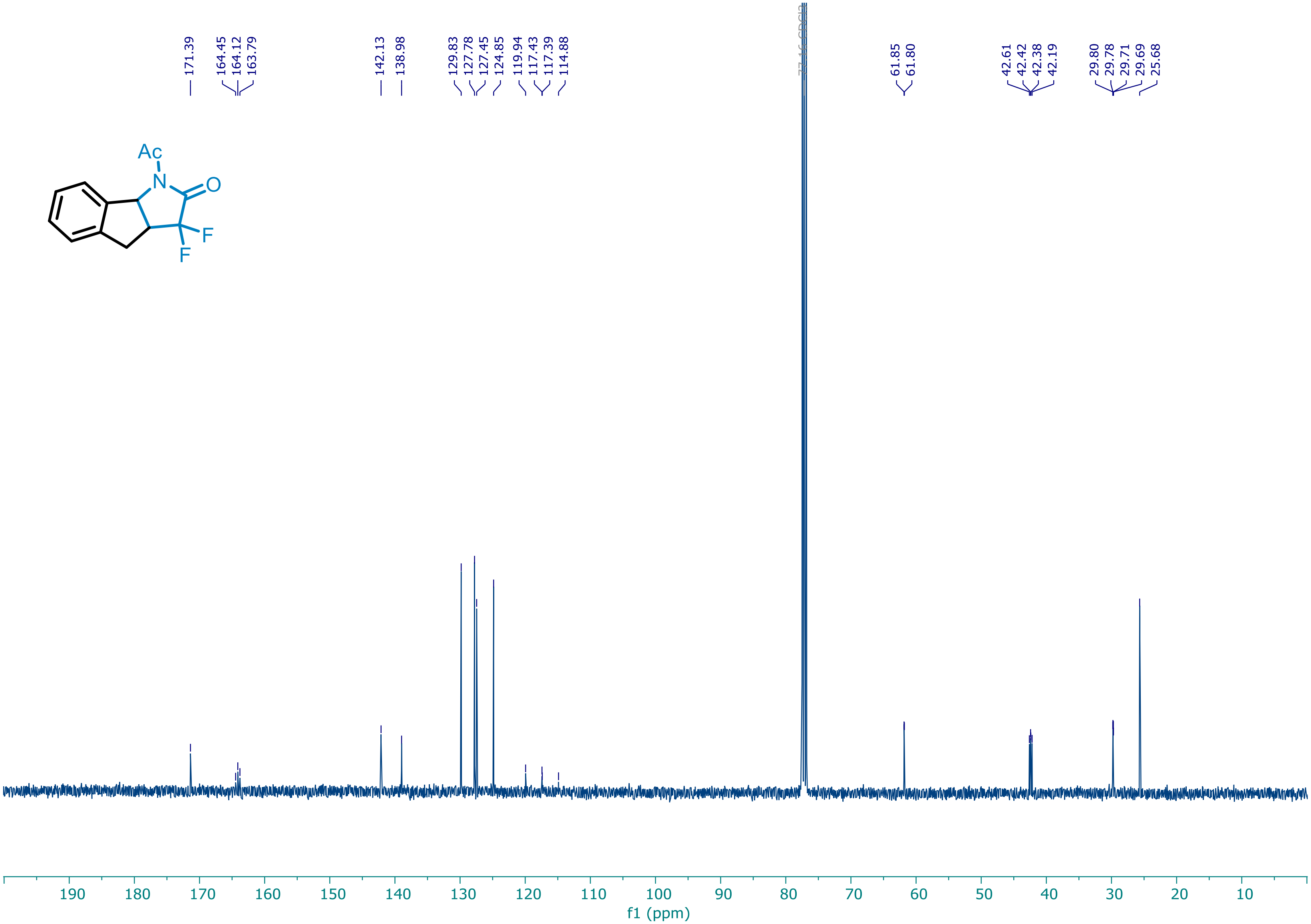

^{19}F NMR (376 MHz, $CDCl_3$) of **21**

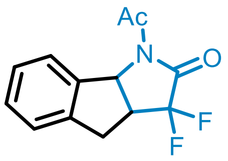

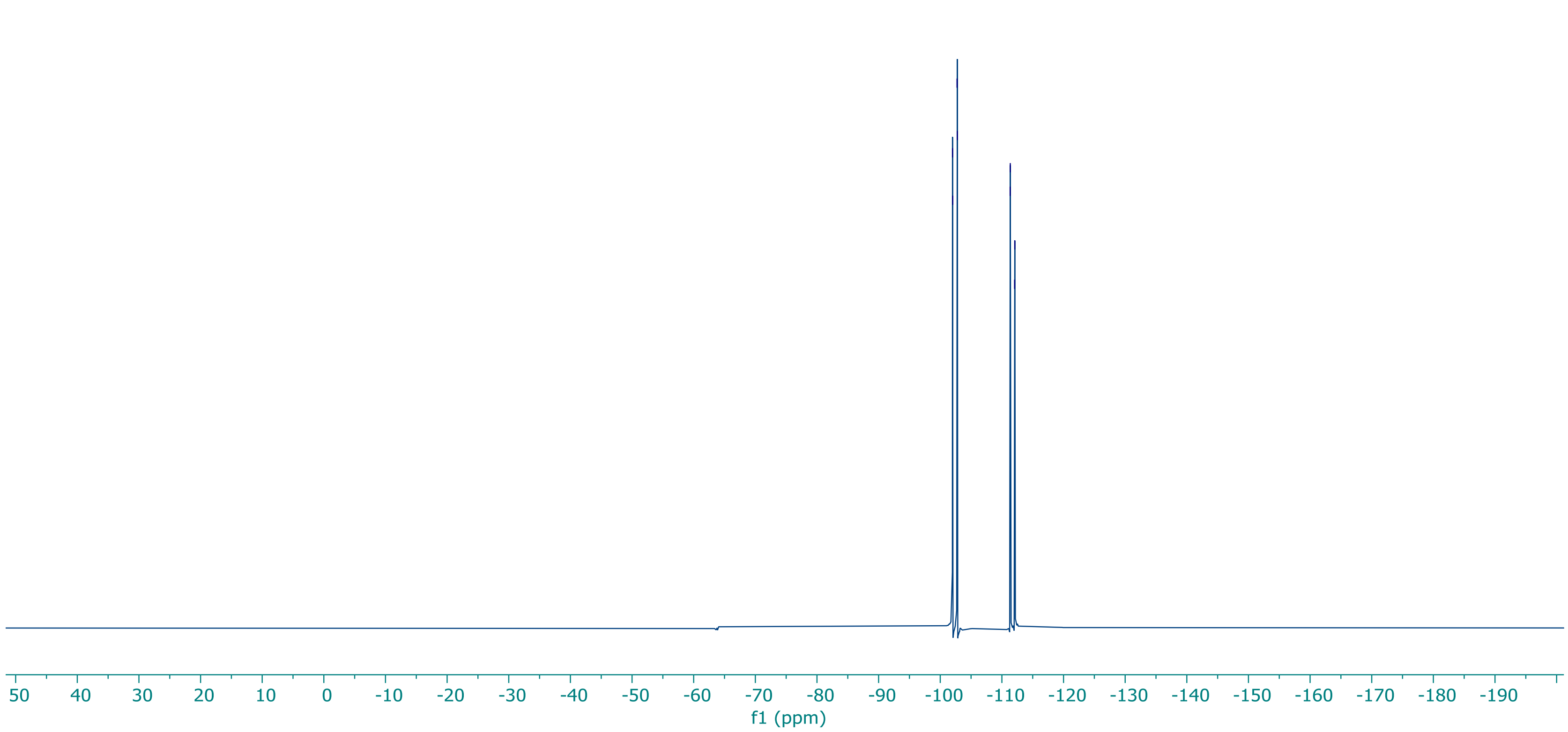

^{1}H NMR (300 MHz, $CDCl_3$) of **22**

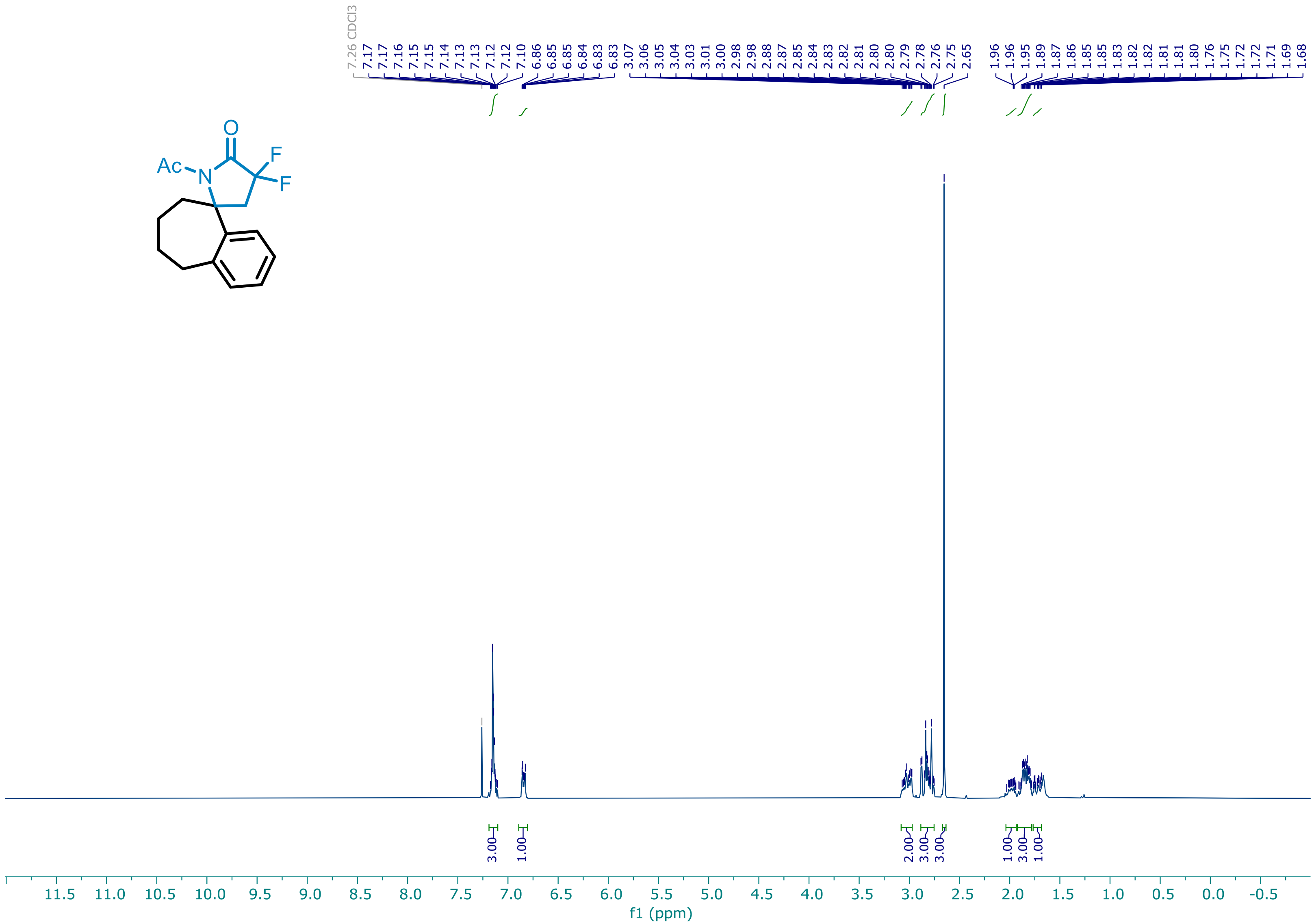

^{13}C NMR (75 MHz, CDCl$_3$) of **22**

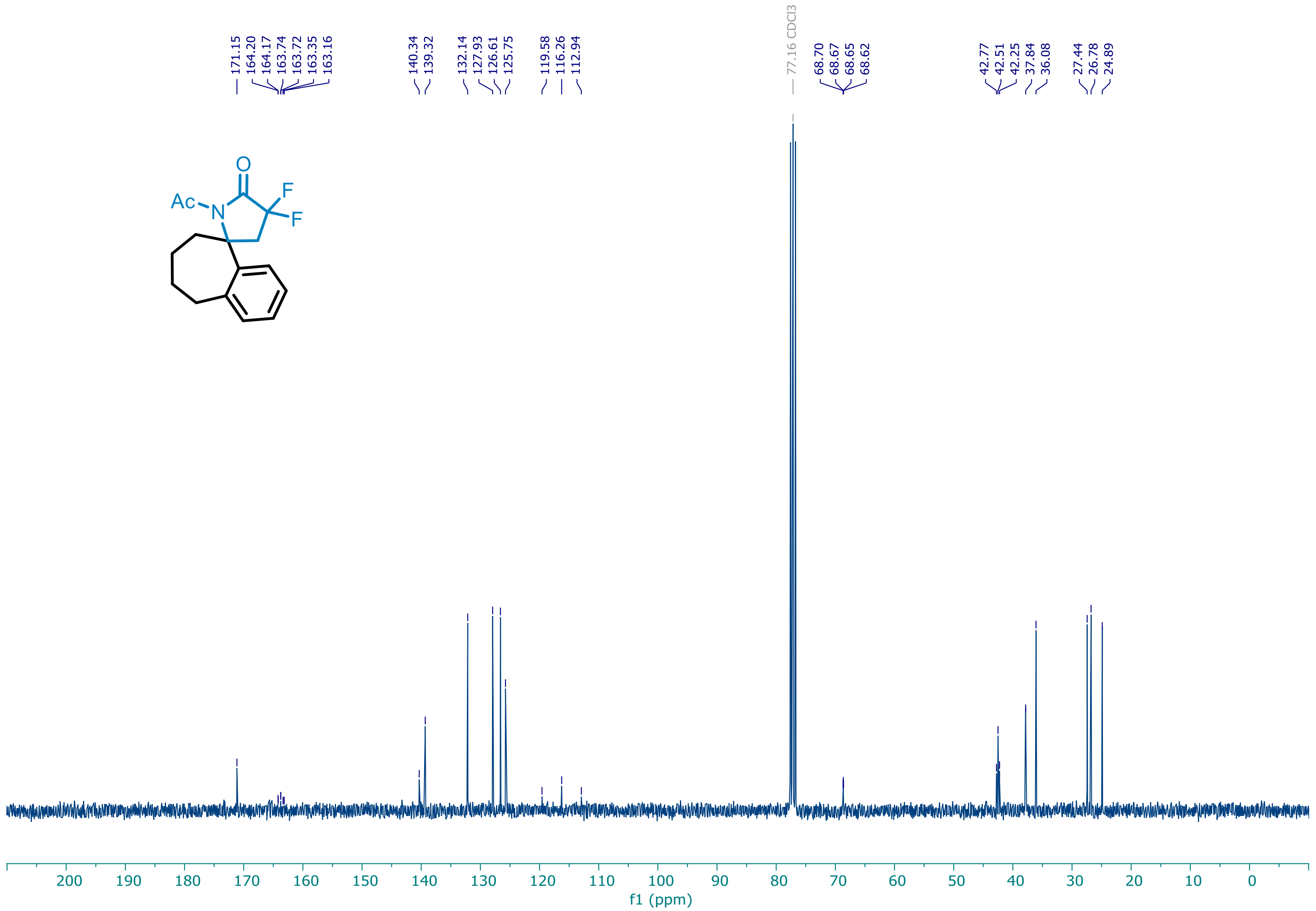

^{19}F NMR (282 MHz, $CDCl_3$) of **22**

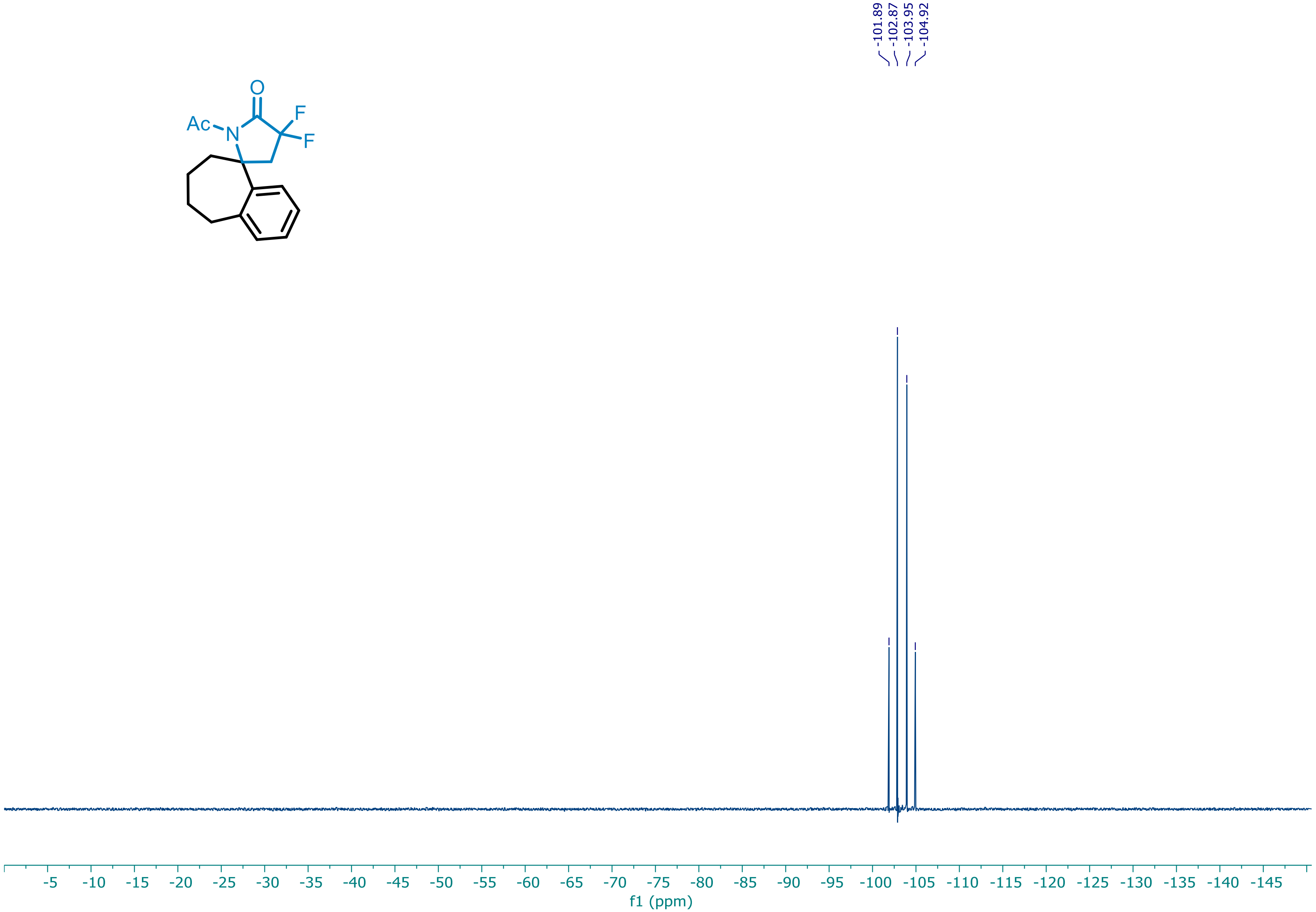

^{1}H NMR (300 MHz, $CDCl_3$) of **23**

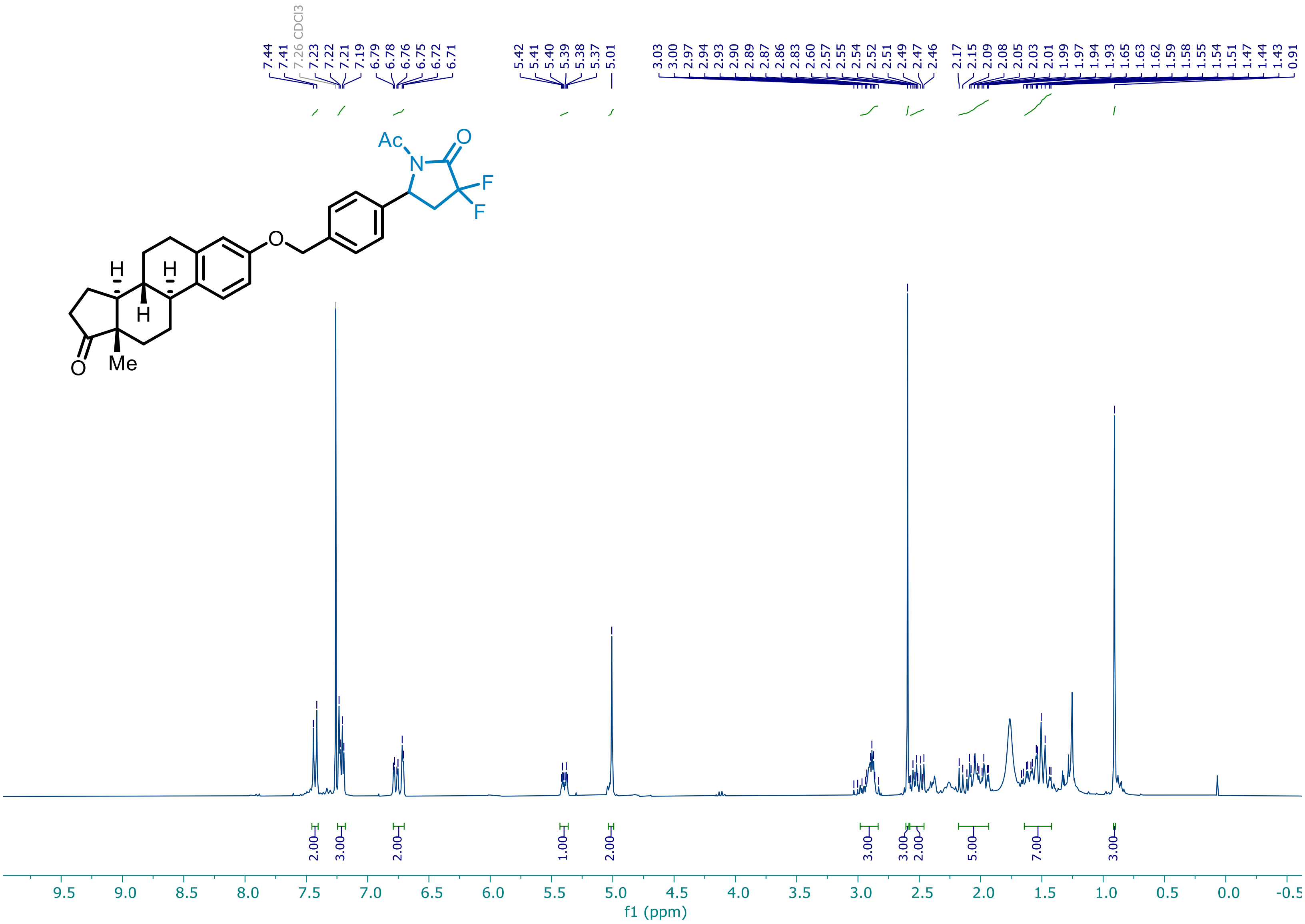

^{13}C NMR (126 MHz, $CDCl_3$) of **23**

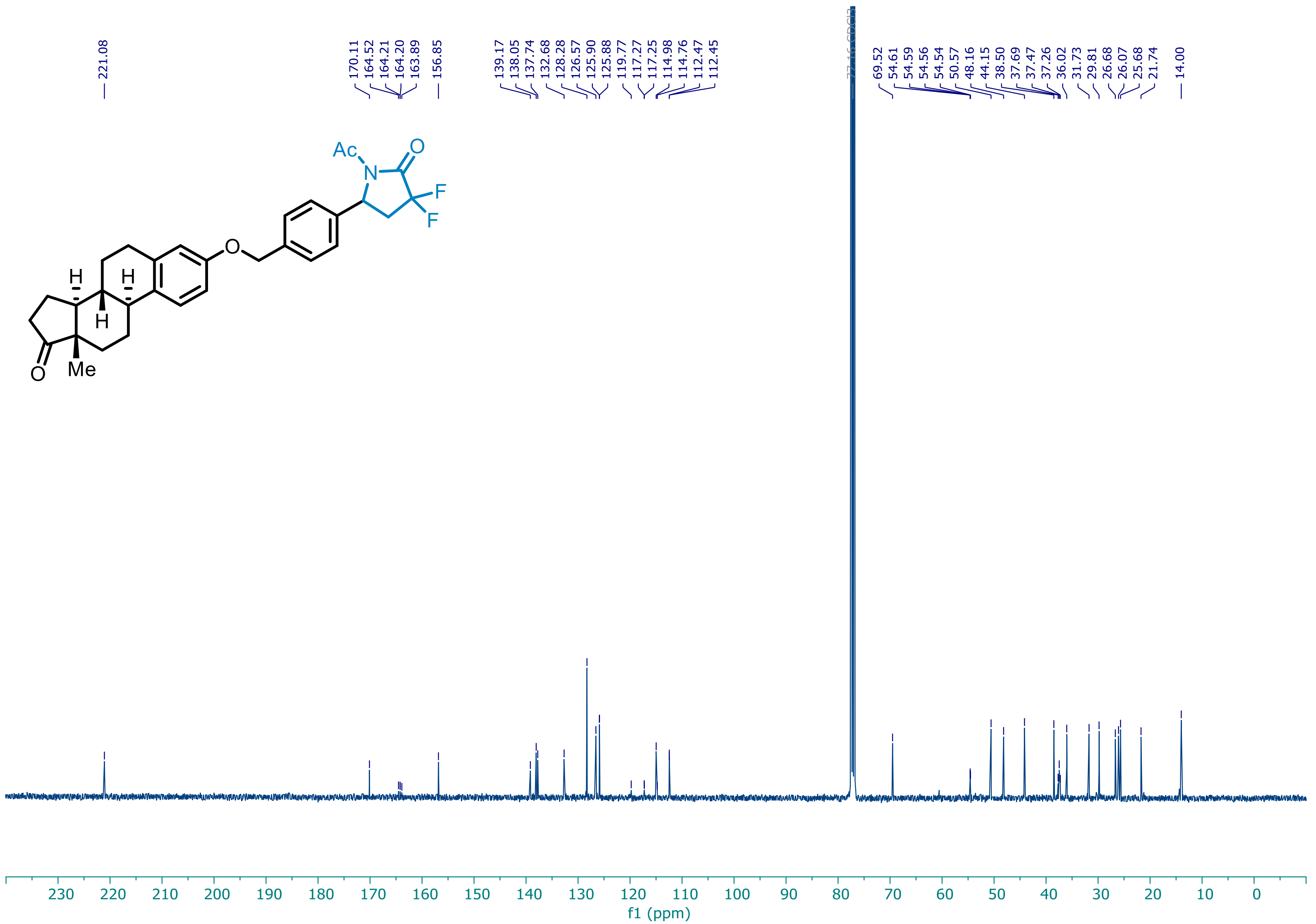

^{19}F NMR (282 MHz, $CDCl_3$) of **23**

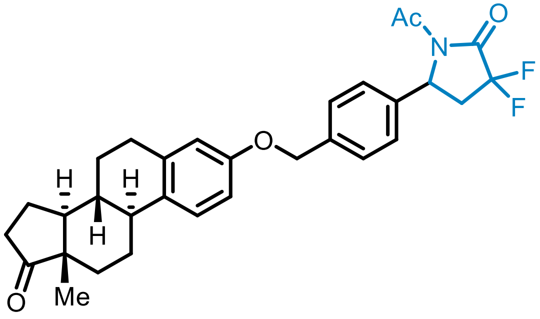

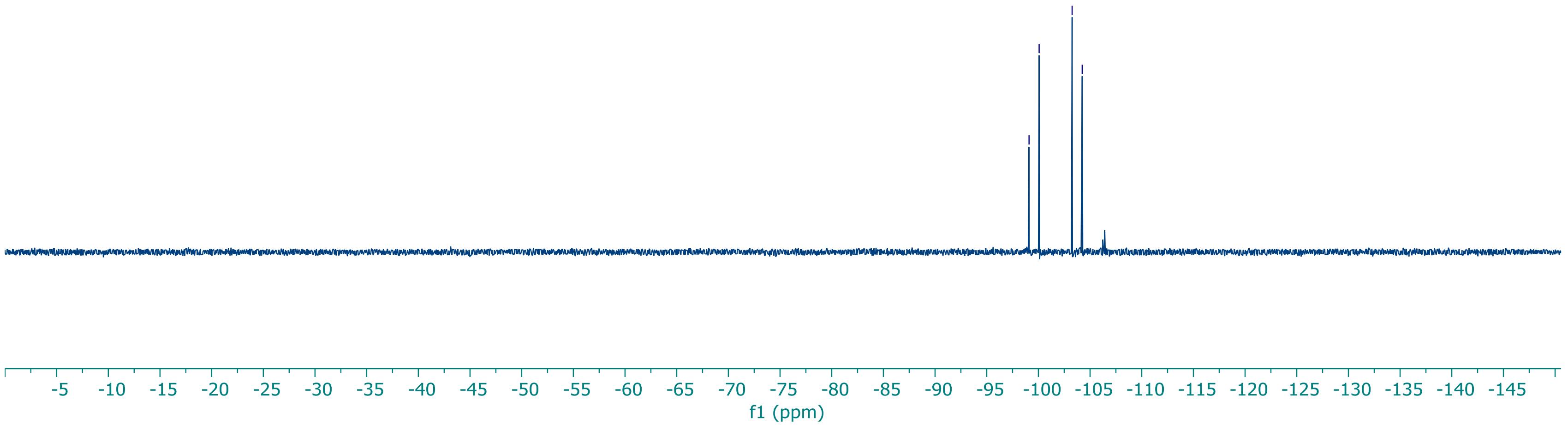

^{1}H NMR (300 MHz, $CDCl_3$) of **3**

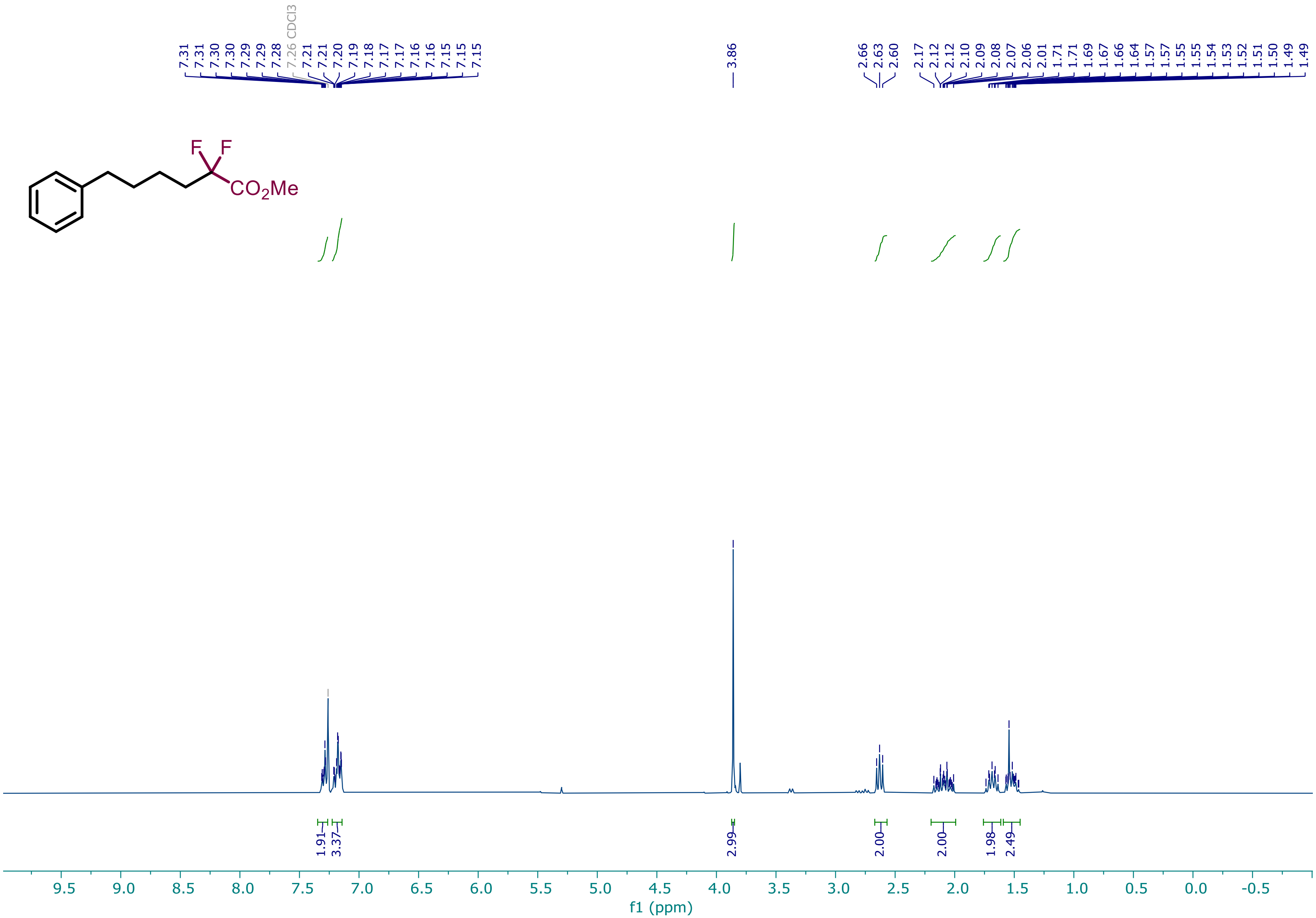

^{19}F NMR (282 MHz, $CDCl_3$) of **3**

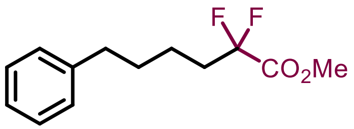

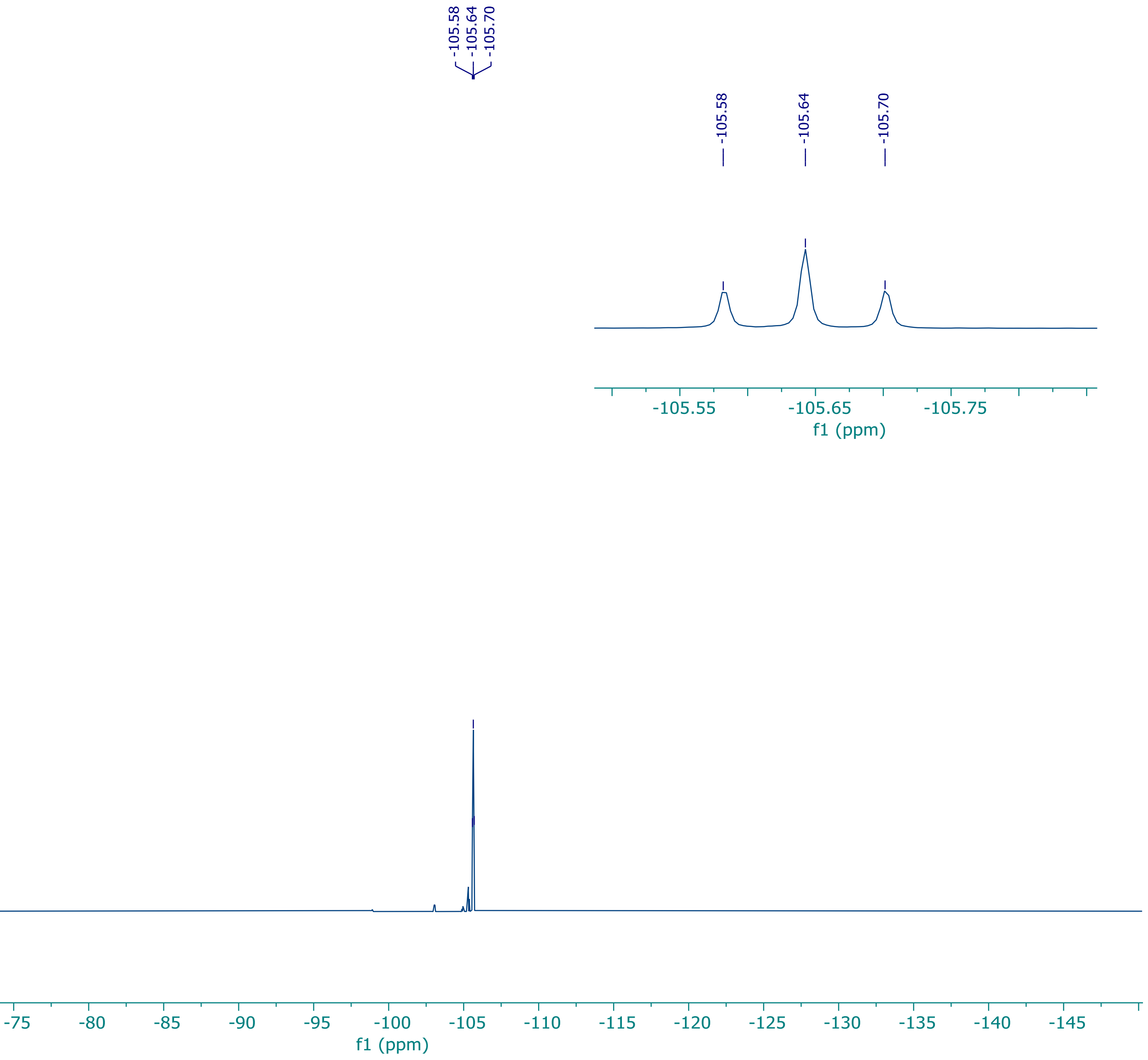

^{1}H NMR (300 MHz, $CDCl_3$) of **24**

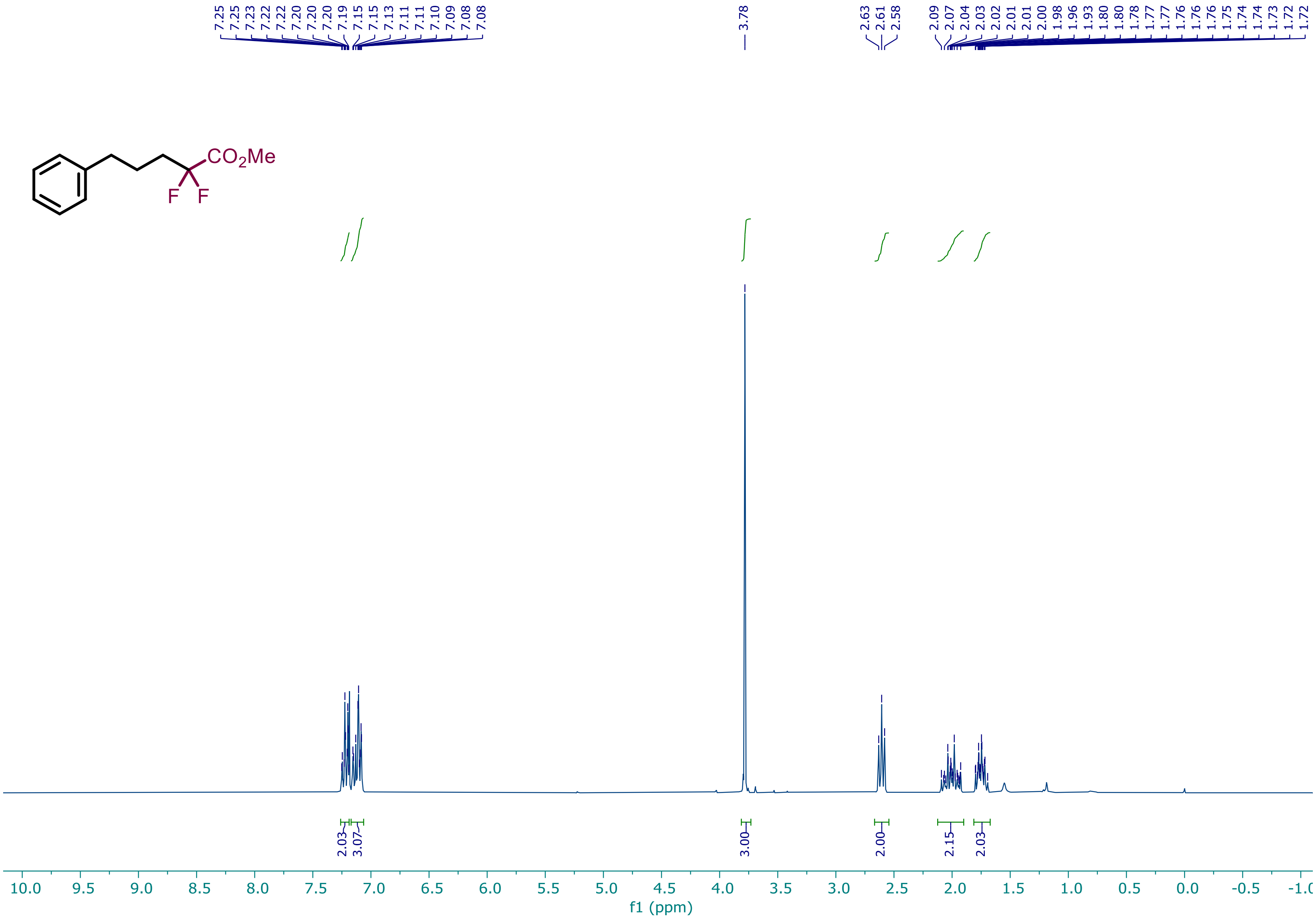

^{13}C NMR (75 MHz, $CDCl_3$) of **24**

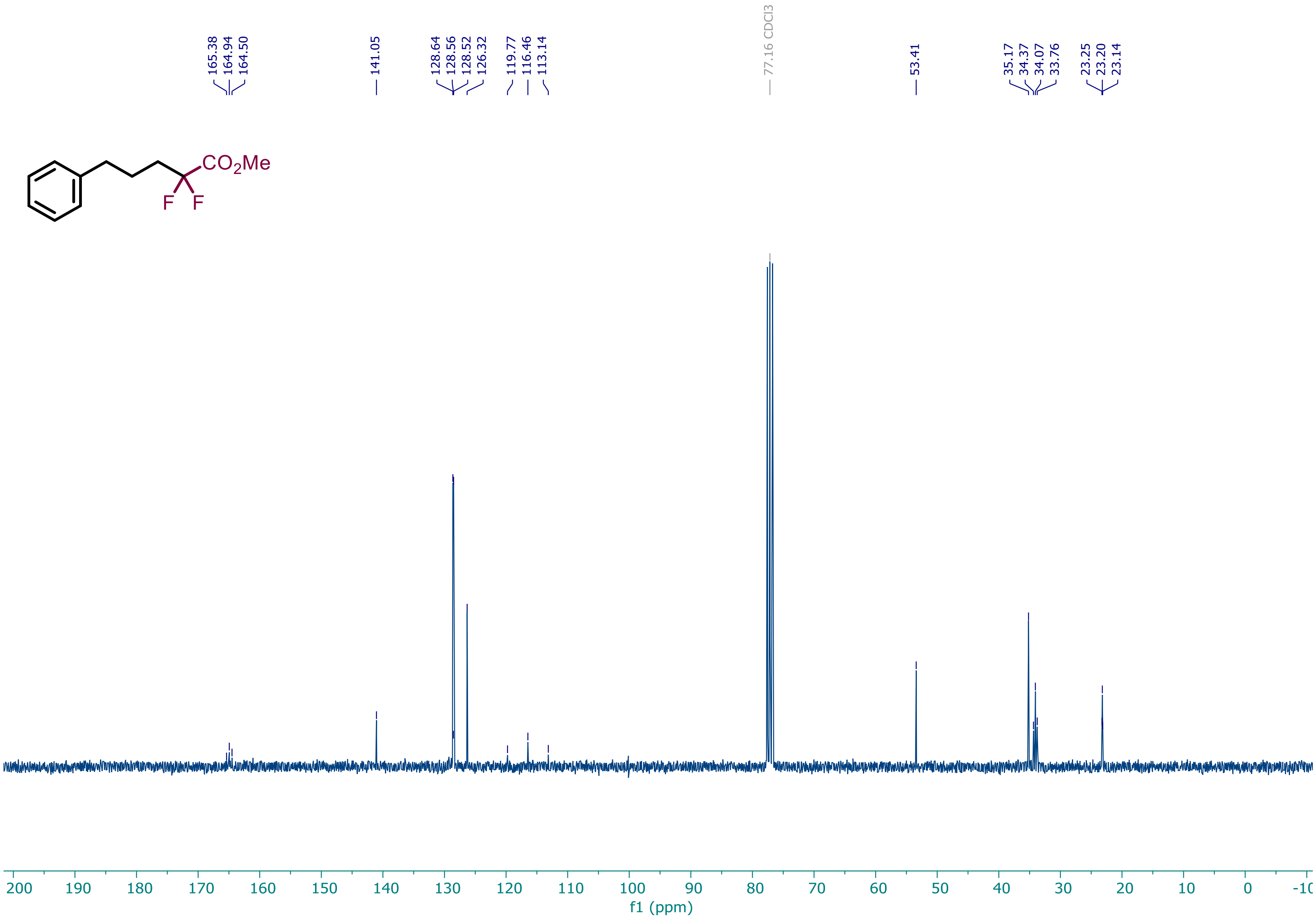

^{19}F NMR (282 MHz, $CDCl_3$) of **24**

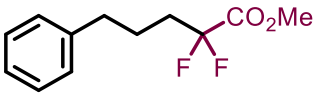

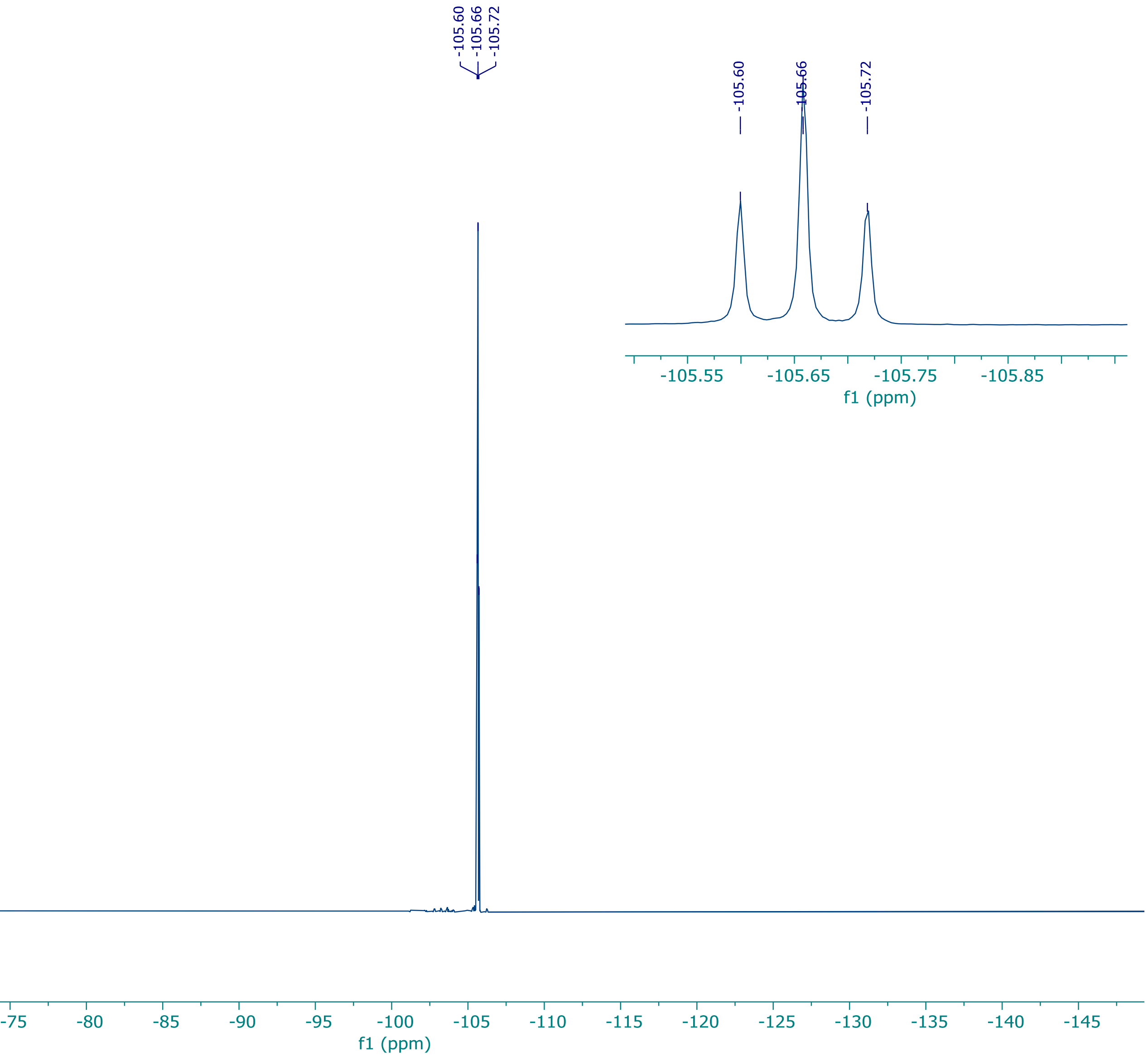

^{1}H NMR (400 MHz, $CDCl_3$) of **25**

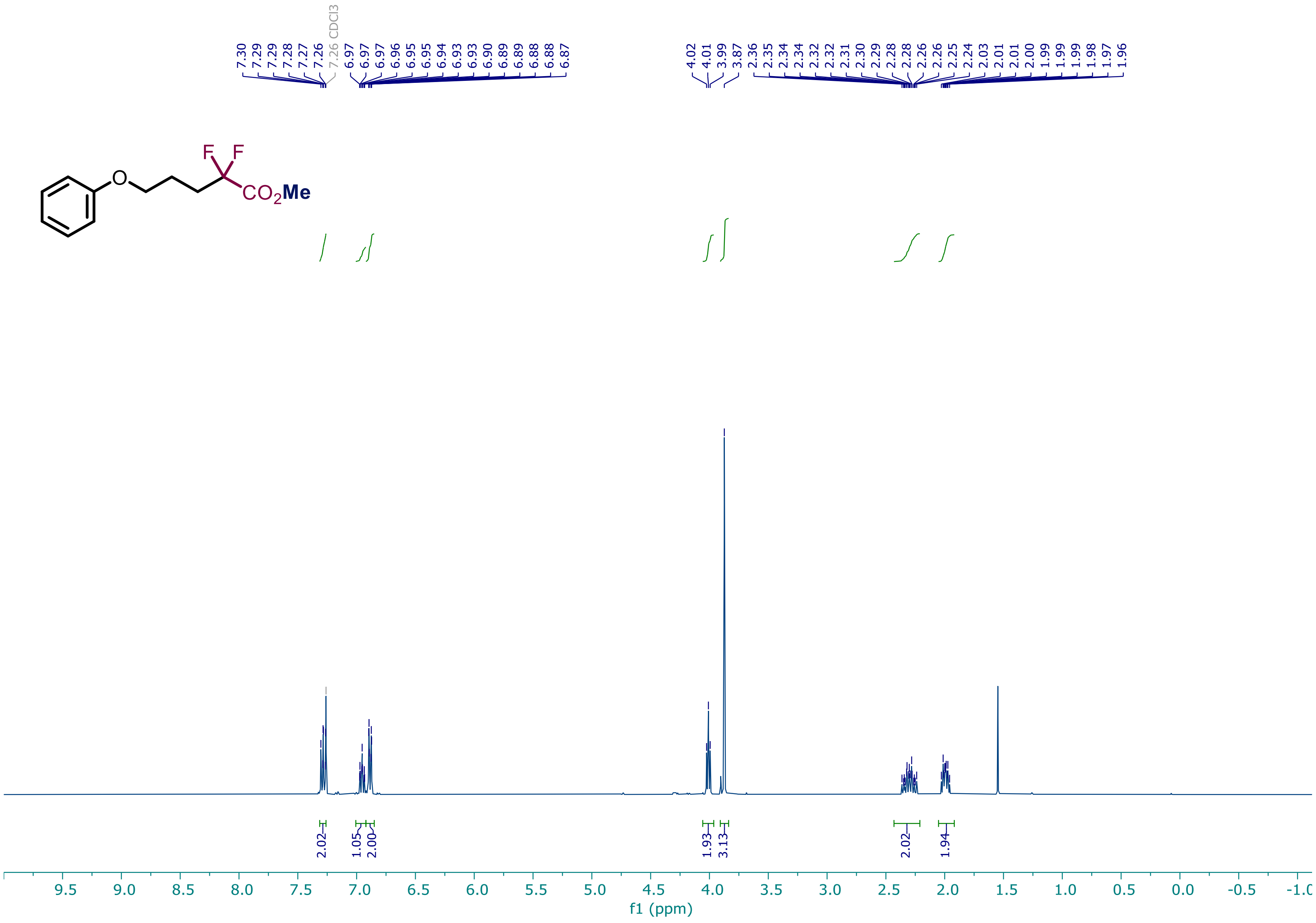

^{13}C NMR (101 MHz, $CDCl_3$) of **25**

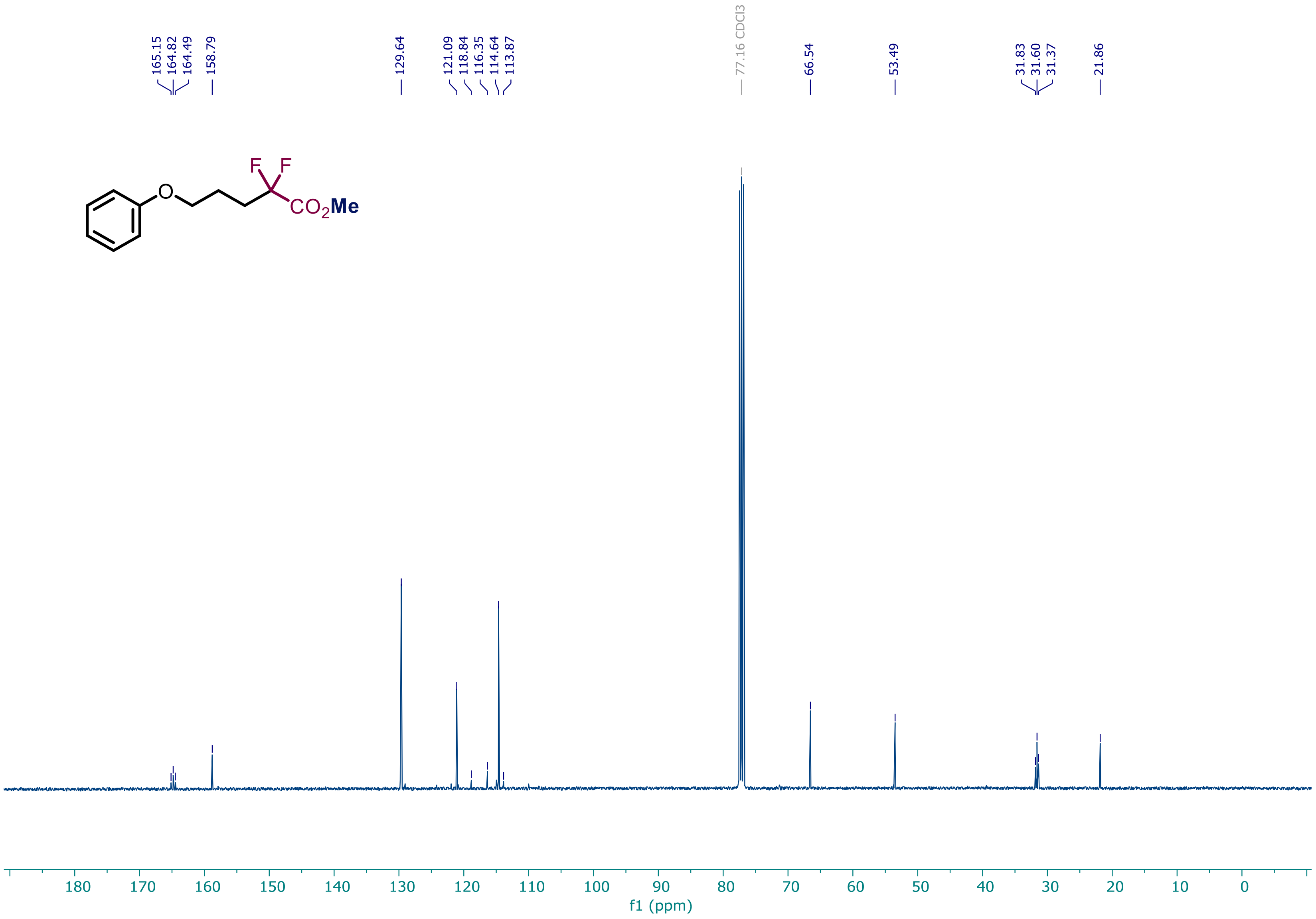

^{19}F NMR (376 MHz, $CDCl_3$) of **25**

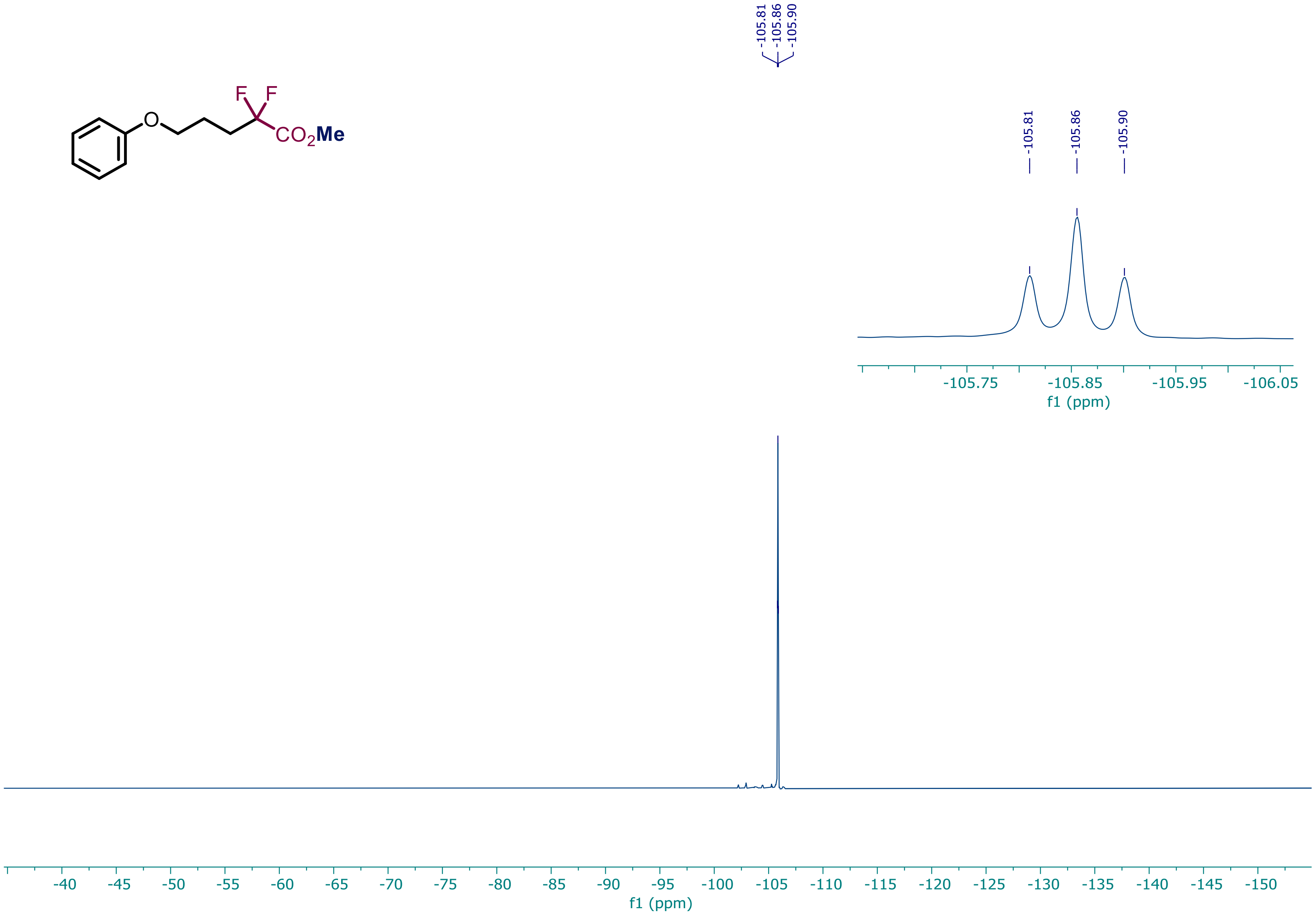

^{1}H NMR (300 MHz, $CDCl_3$) of **26**

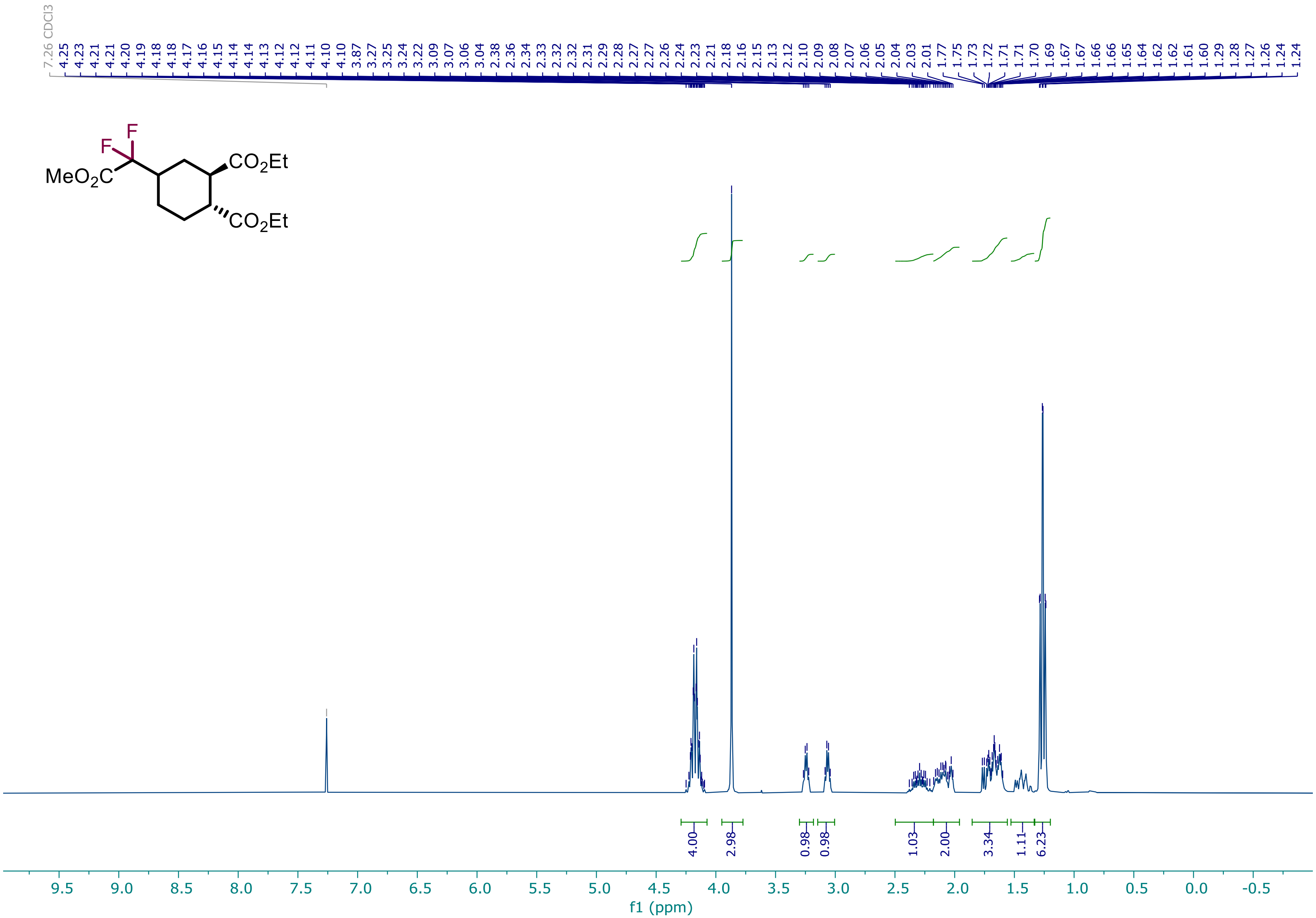

^{13}C NMR (101 MHz, $CDCl_3$) of **26**

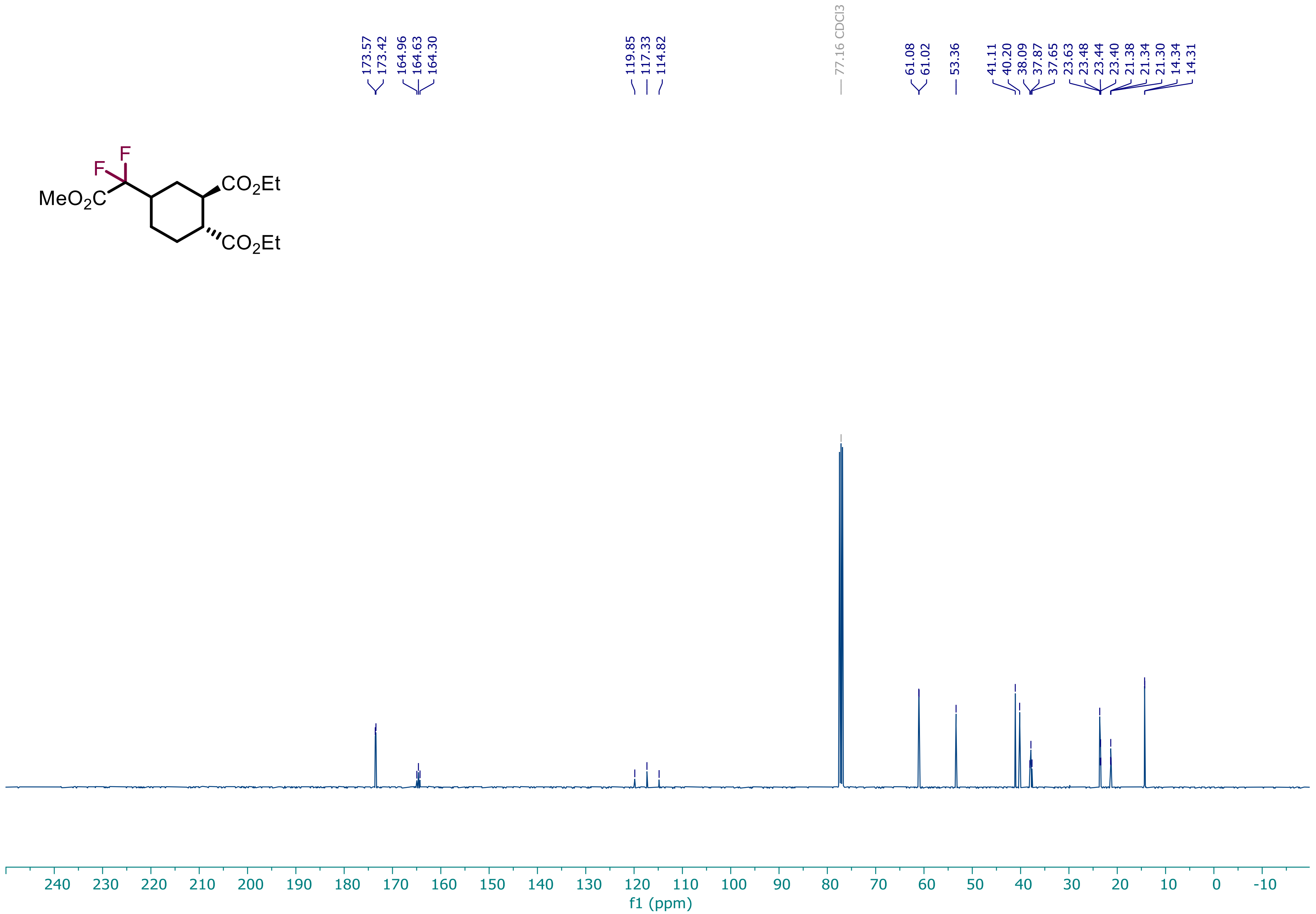

^{19}F NMR (282 MHz, $CDCl_3$) of **26**

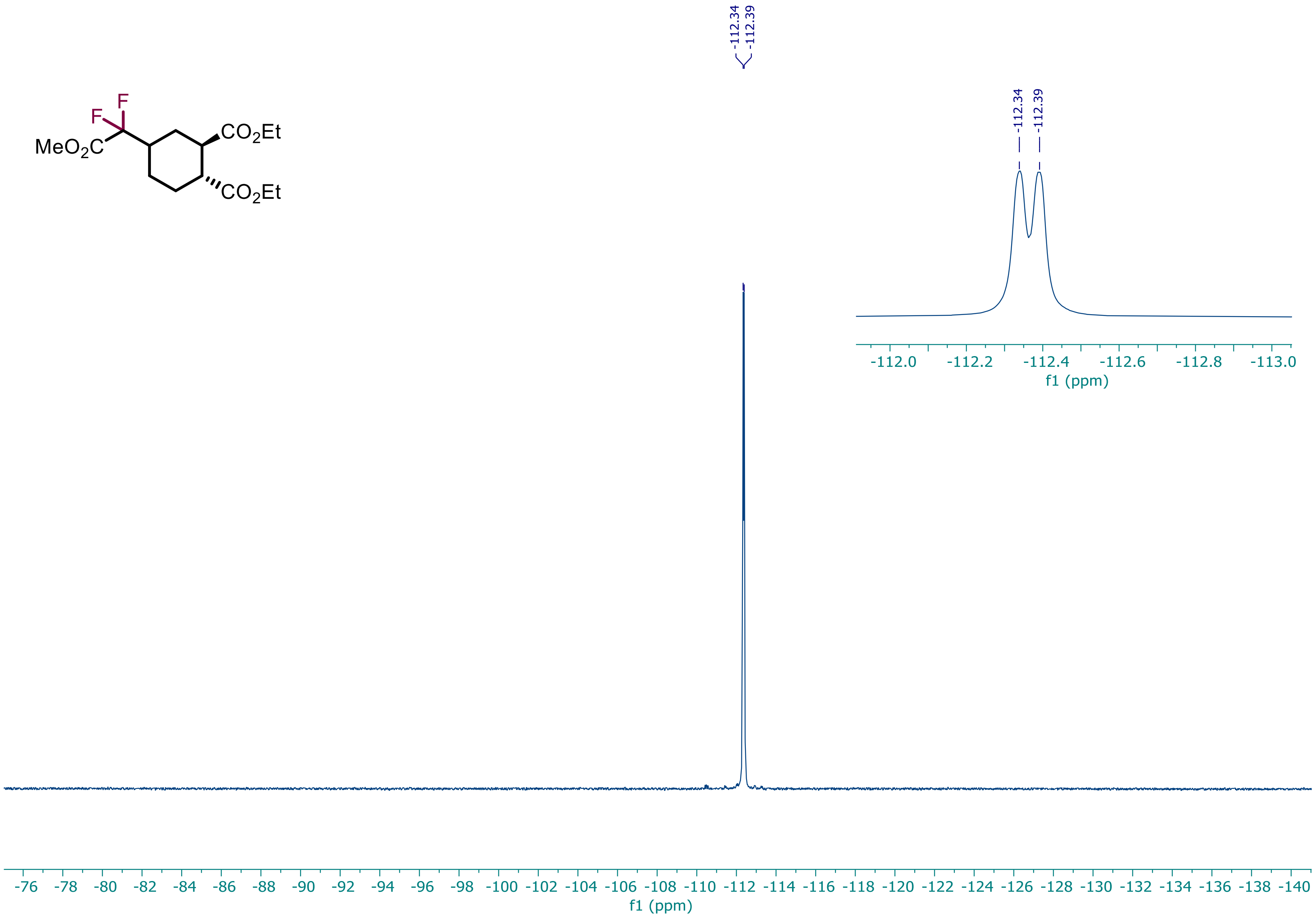

^{1}H NMR (400 MHz, $CDCl_3$) of **27**

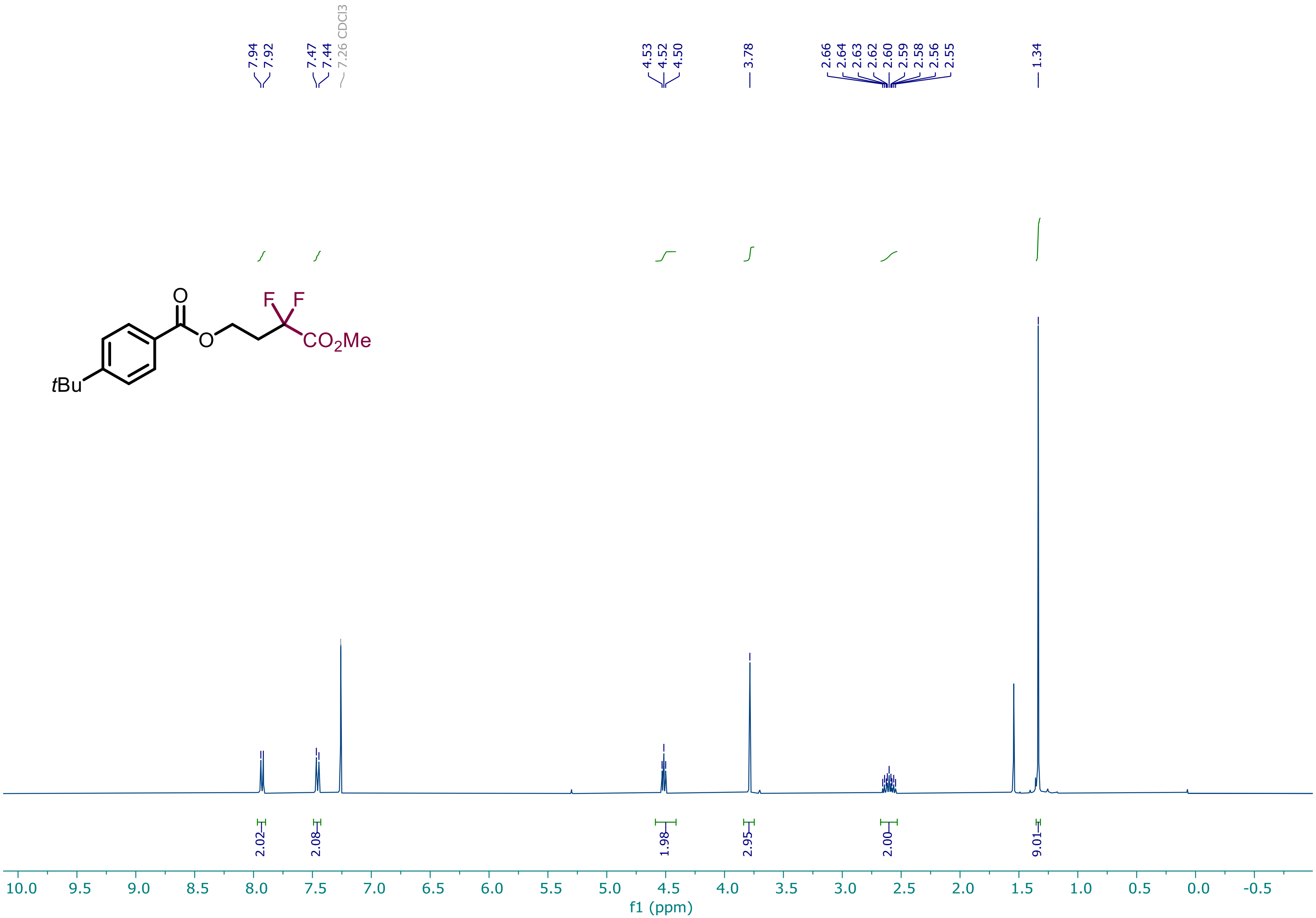

^{13}C NMR (101 MHz, $CDCl_3$) of **27**

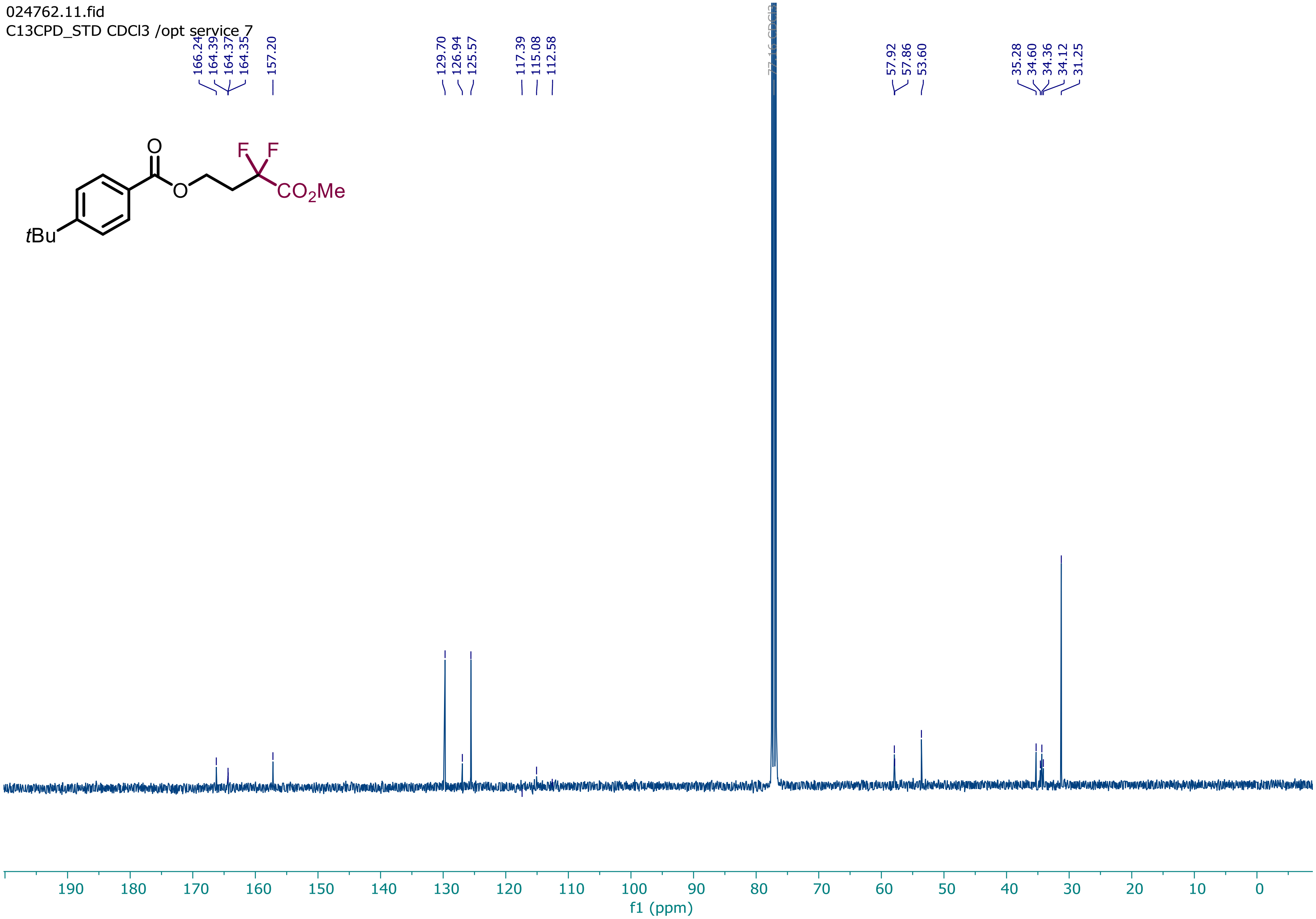

^{19}F NMR (376 MHz, $CDCl_3$) of **27**

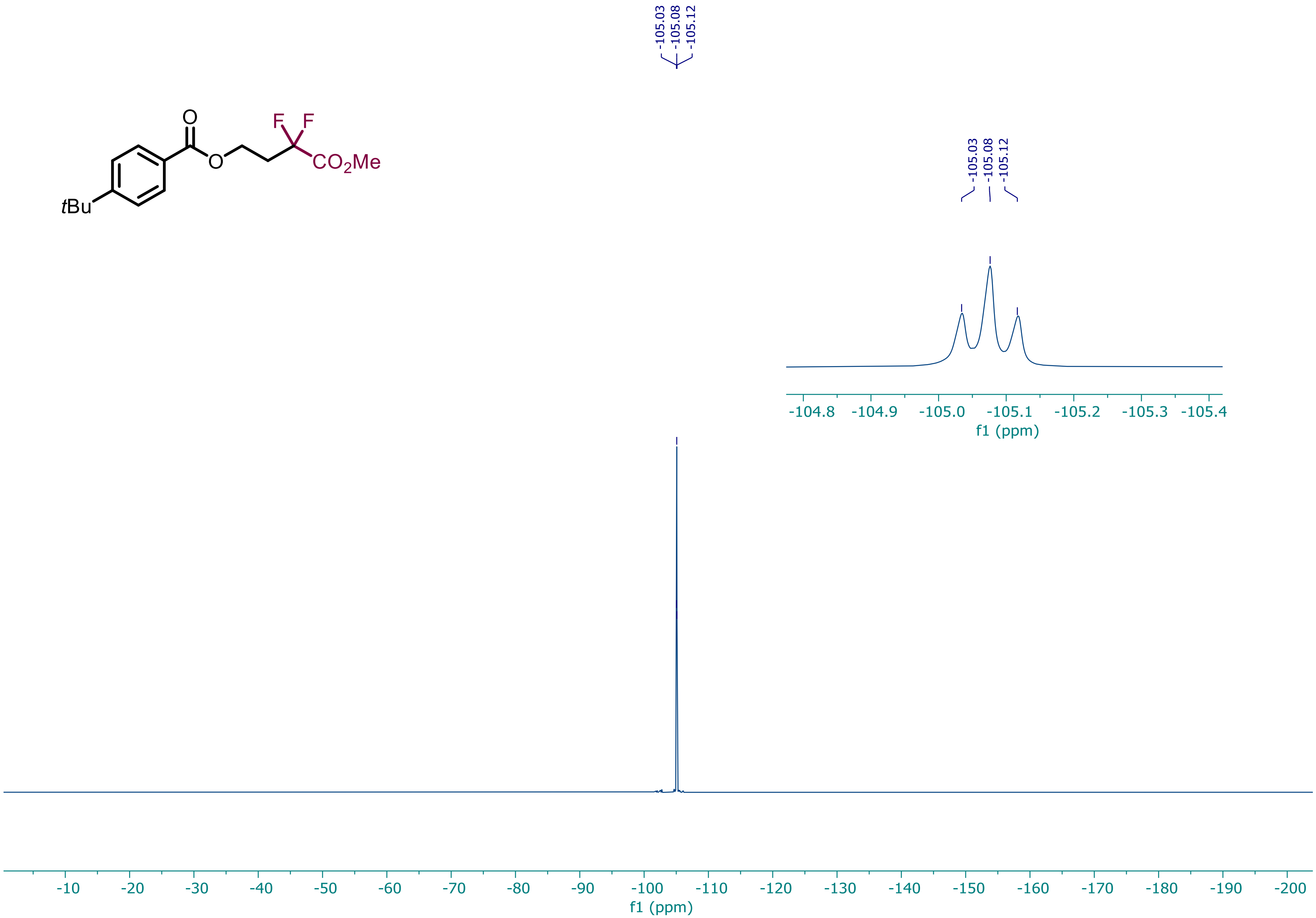

^1H NMR (400 MHz, $CDCl_3$) of **28**

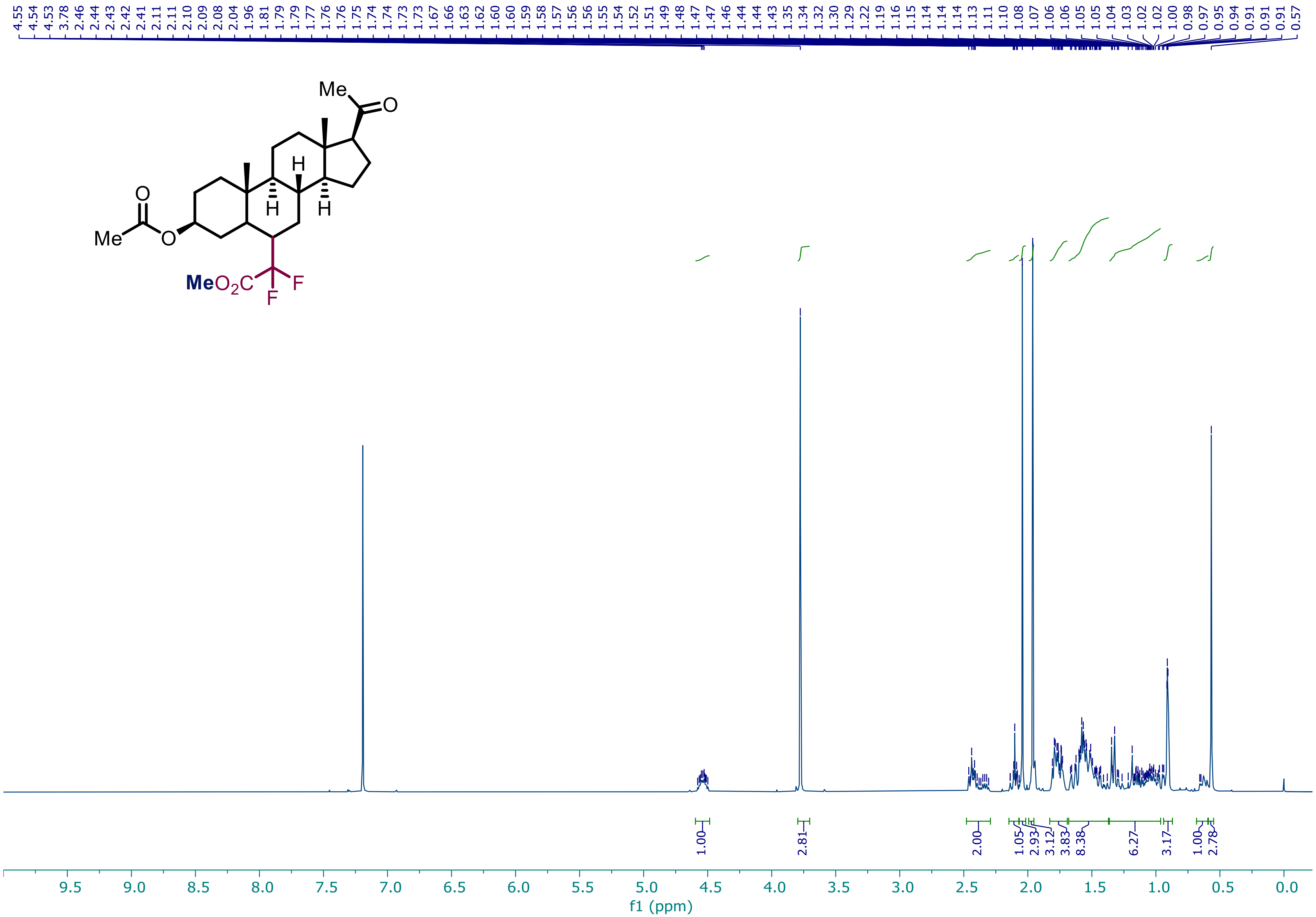

^{13}C NMR (101 MHz, $CDCl_3$) of **28**

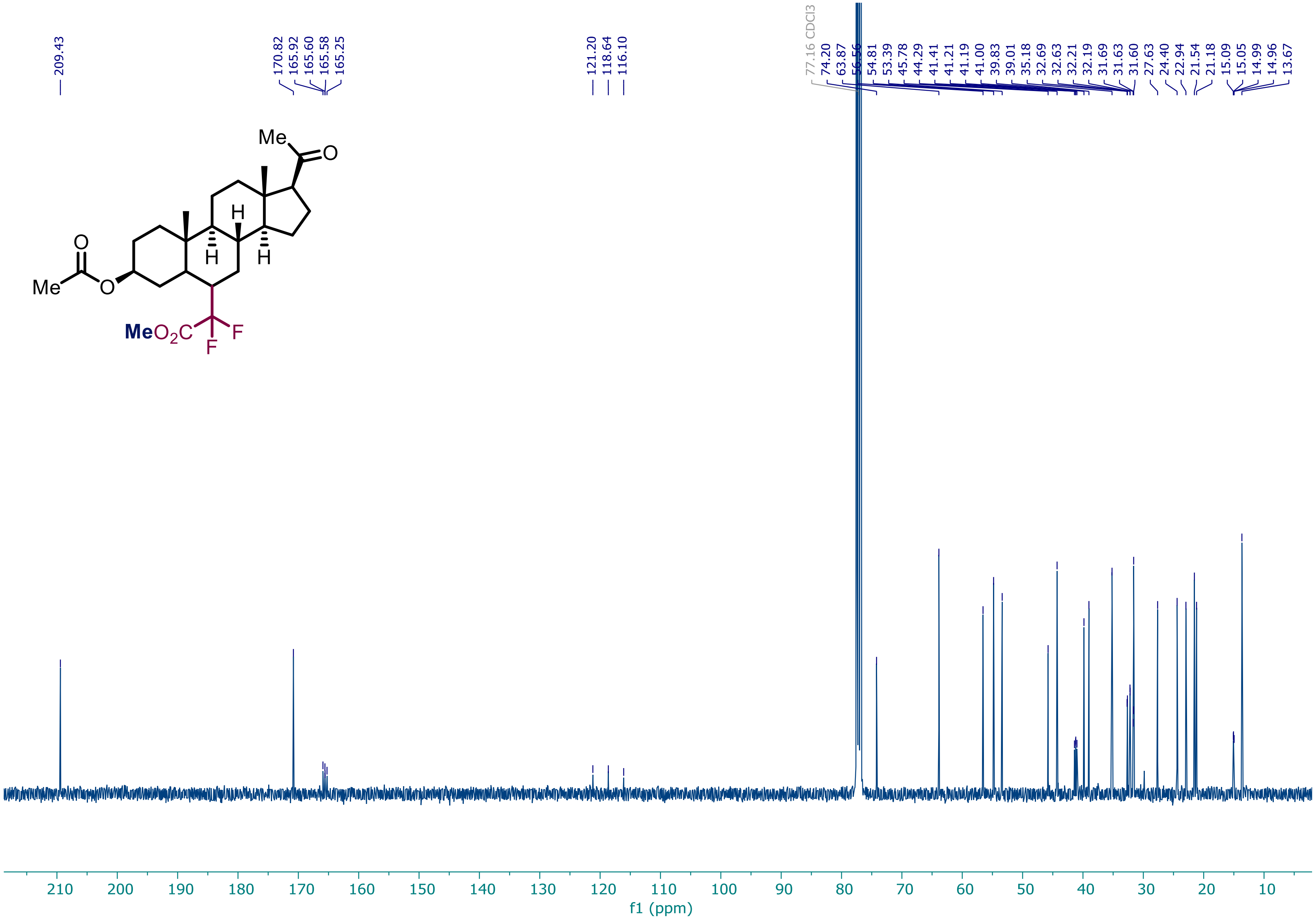

^{19}F NMR (376 MHz, $CDCl_3$) of **28**

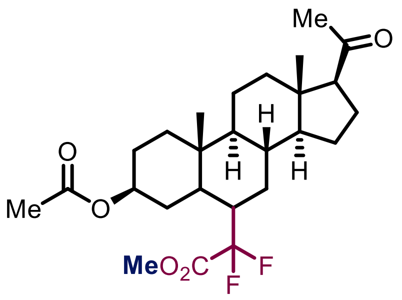

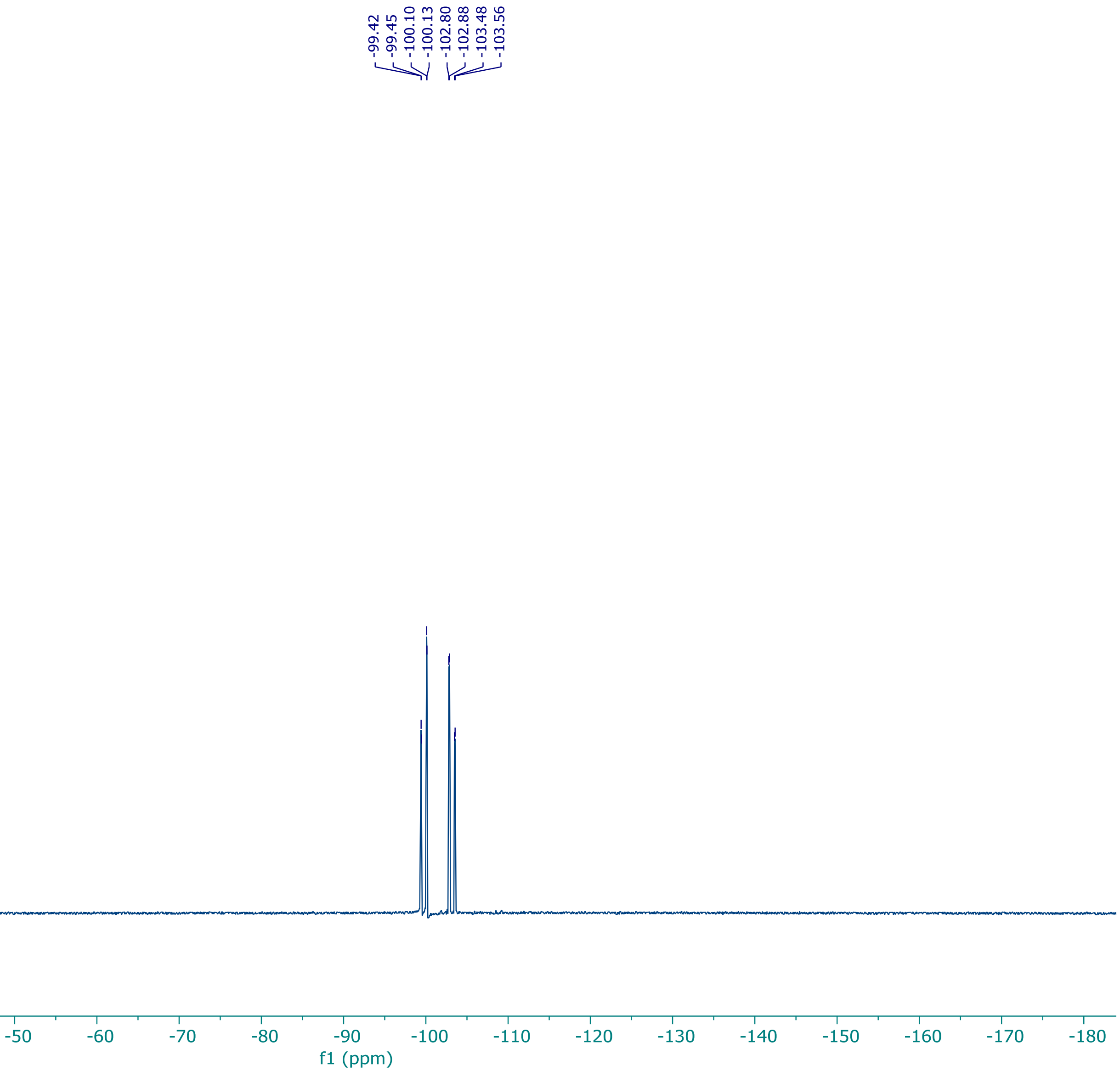

^{1}H NMR (400 MHz, $CDCl_3$) of **29**

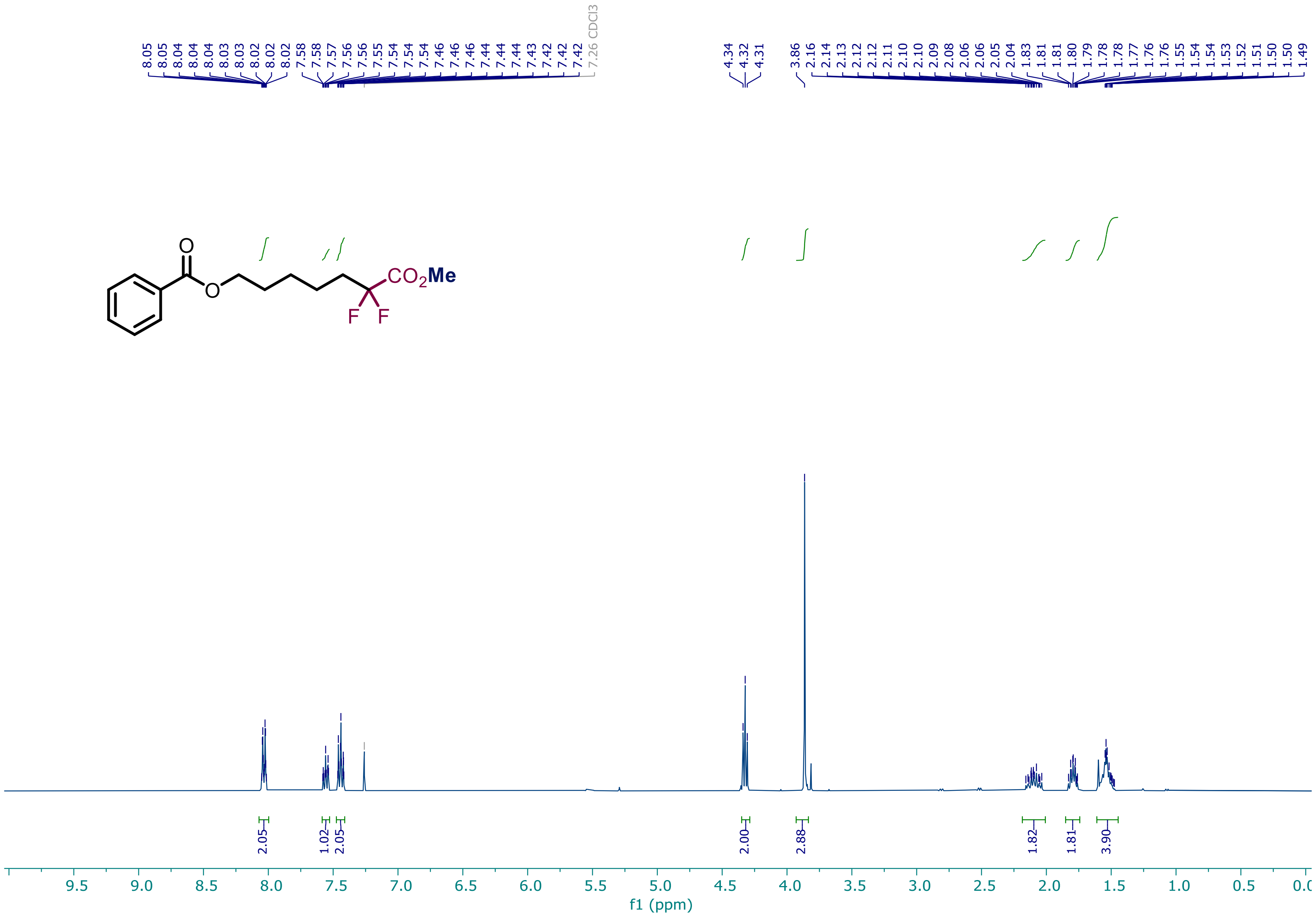

^{13}C NMR (101 MHz, $CDCl_3$) of **29**

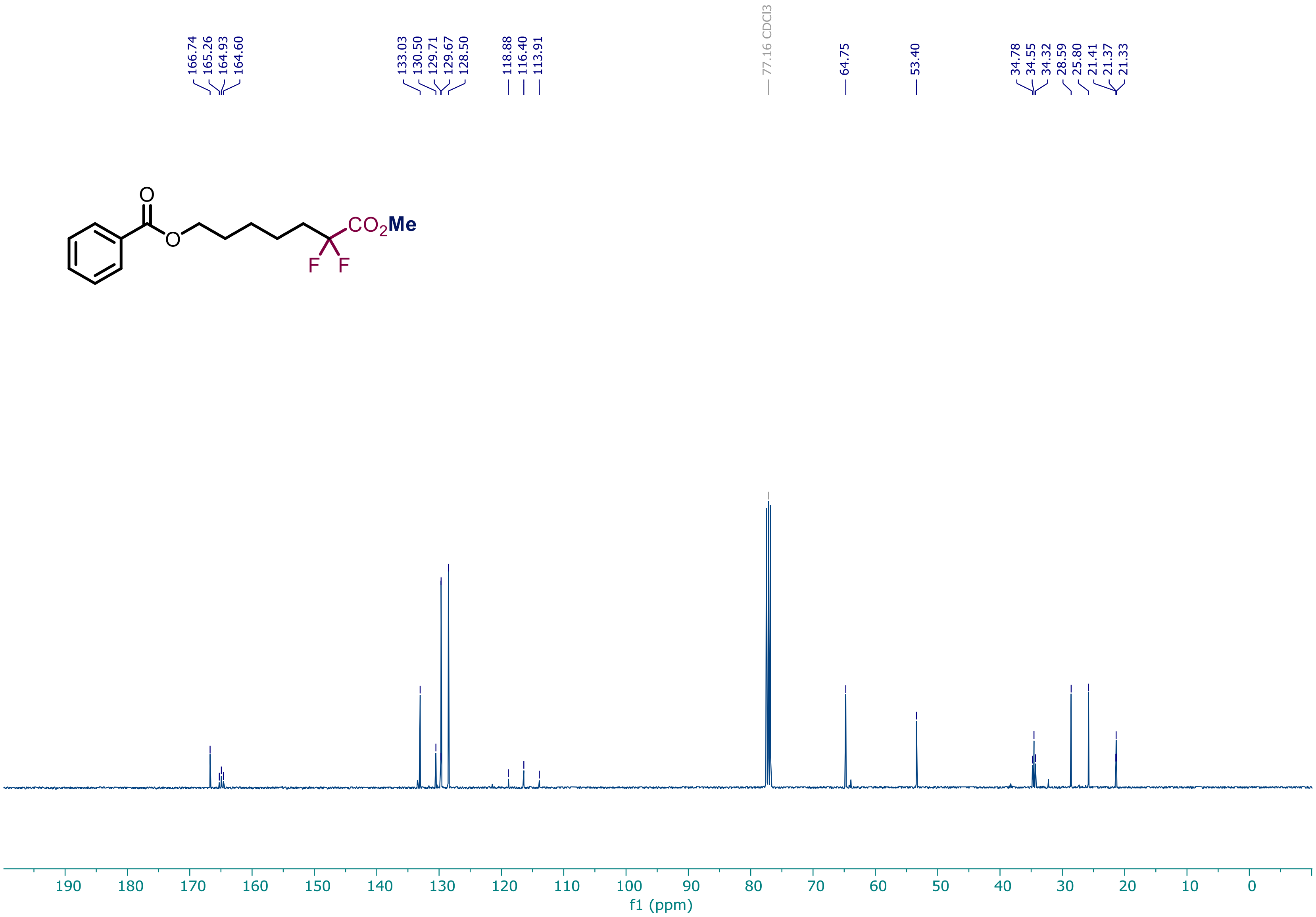

^{19}F NMR (376 MHz, $CDCl_3$) of **29**

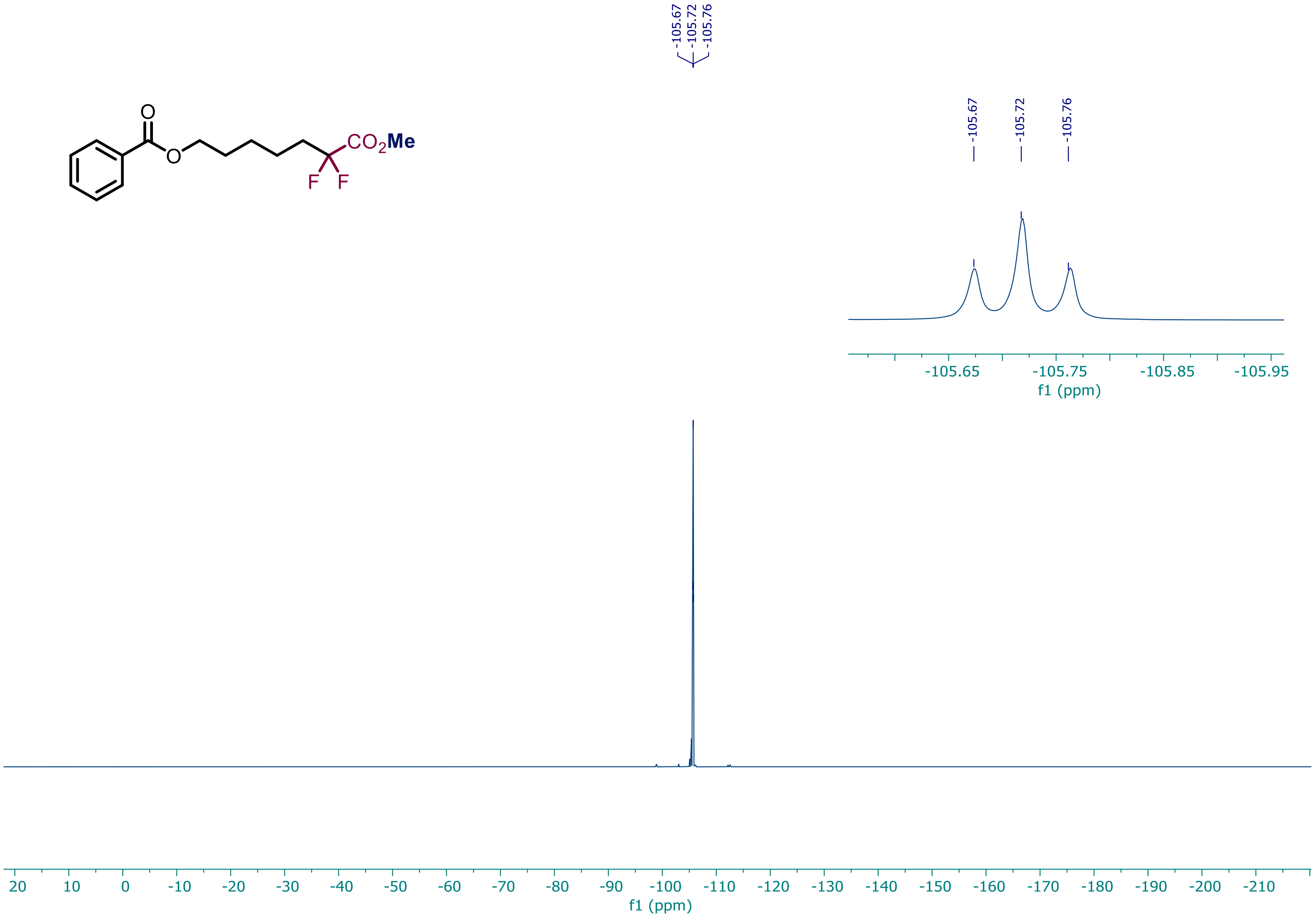

^{1}H NMR (300 MHz, $CDCl_3$) of **30**

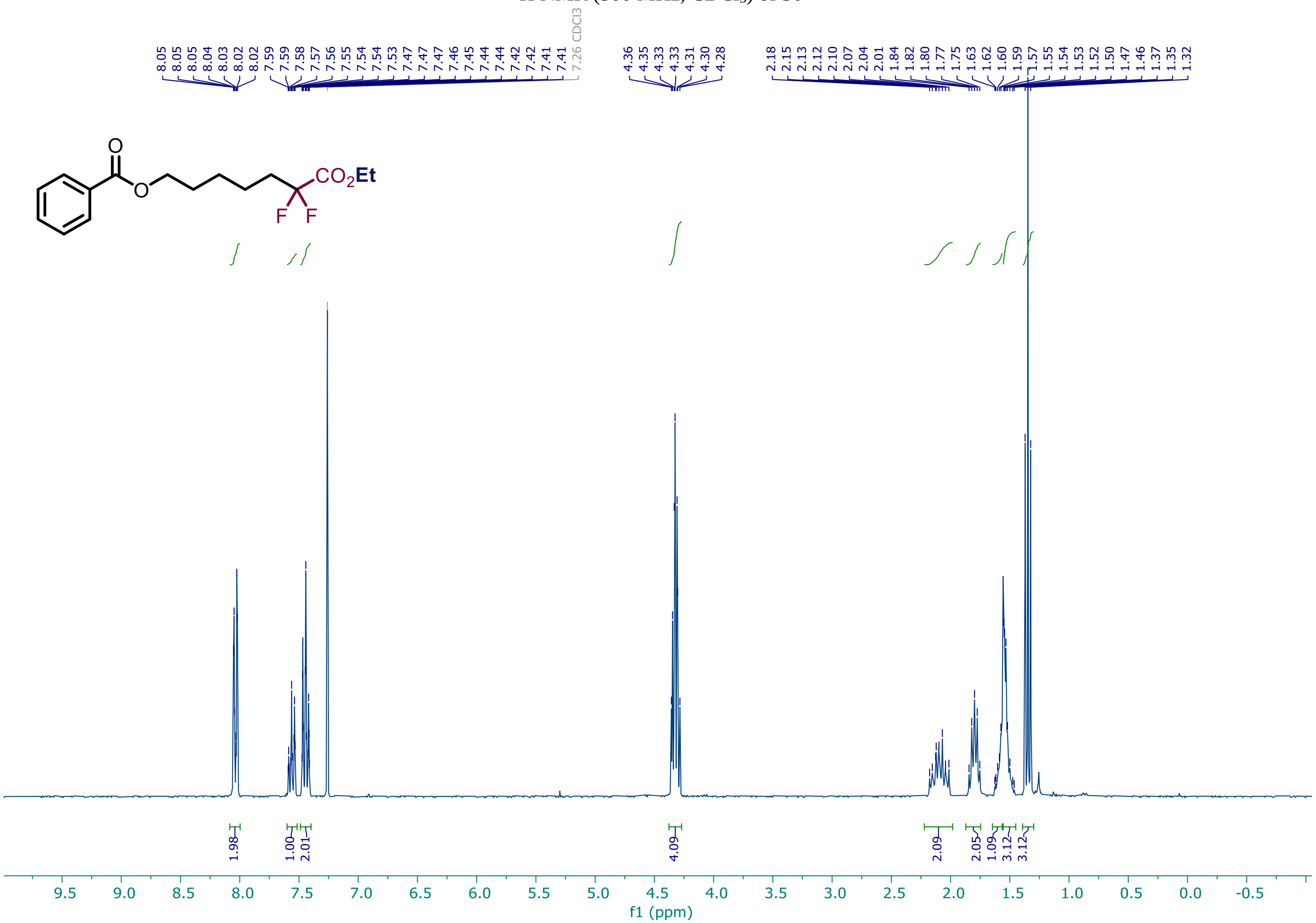

^{19}F NMR (282 MHz, $CDCl_3$) of **30**

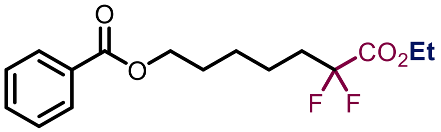

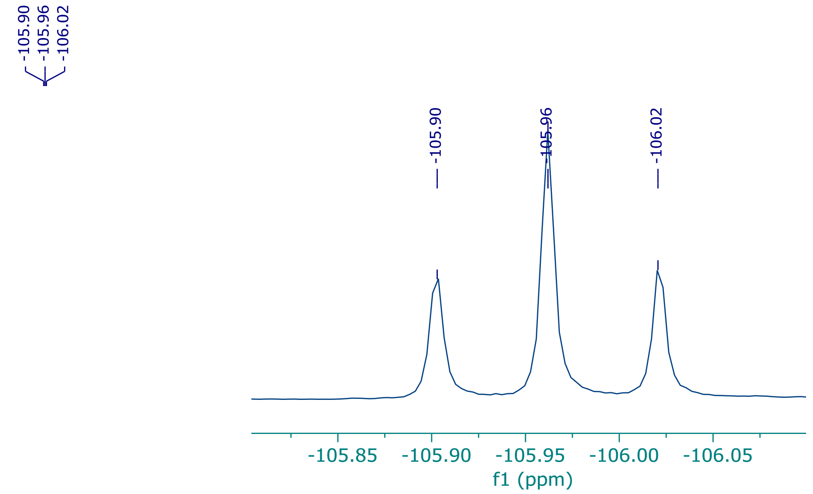

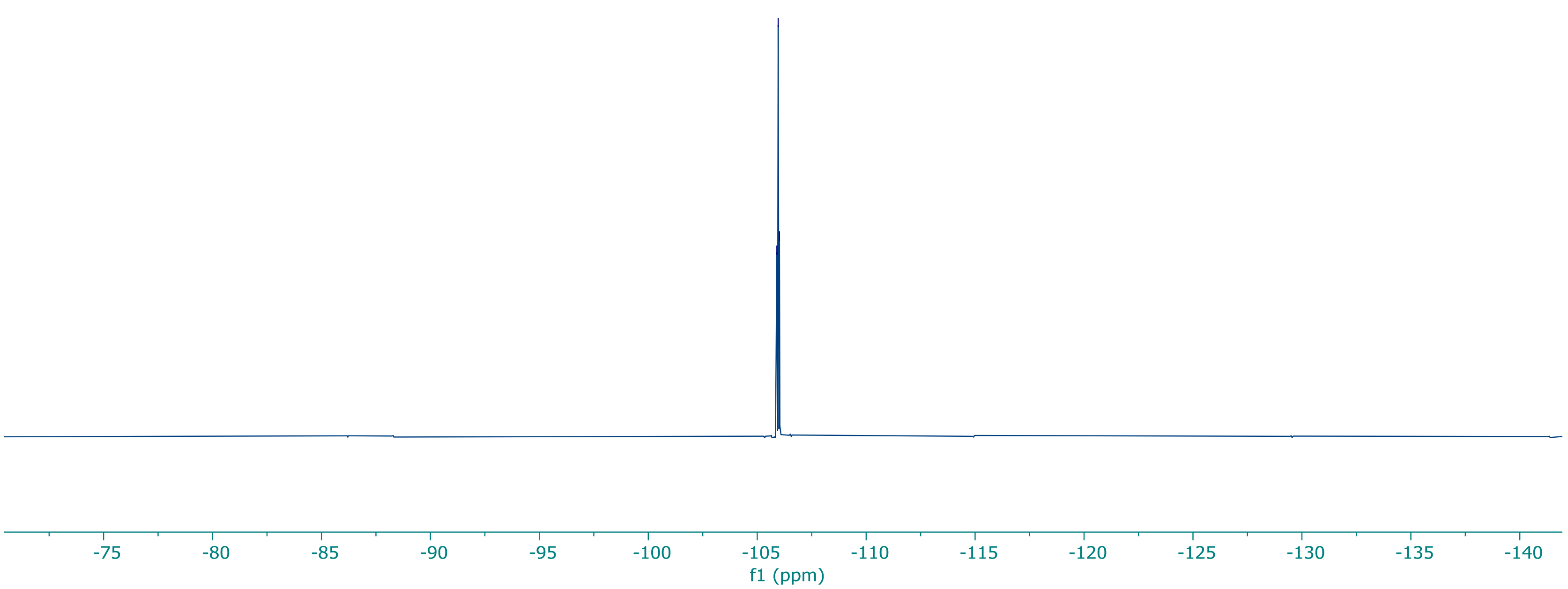

^{1}H NMR (400 MHz, $CDCl_3$) of **31**

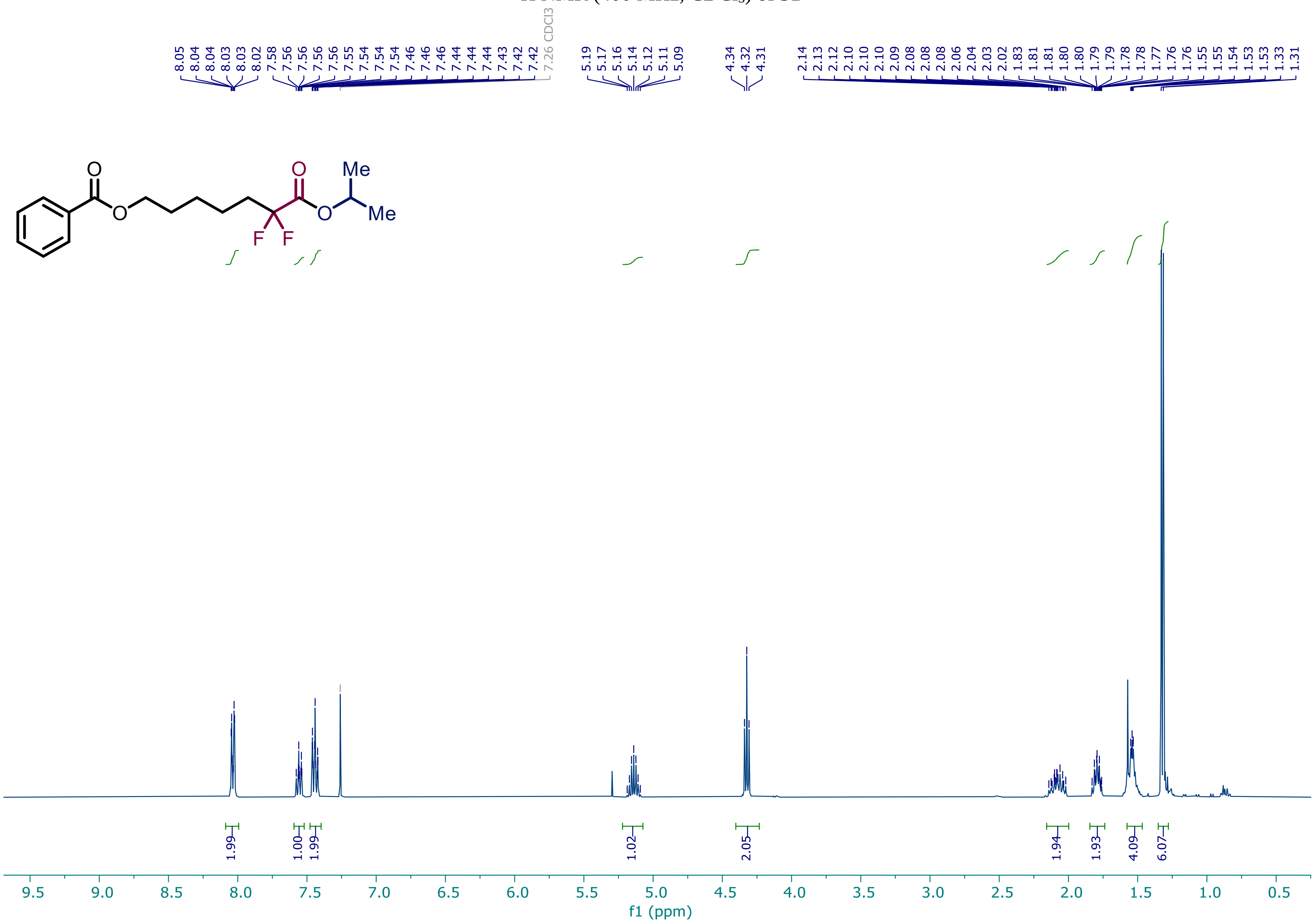

^{13}C NMR (101 MHz, $CDCl_3$) of **31**

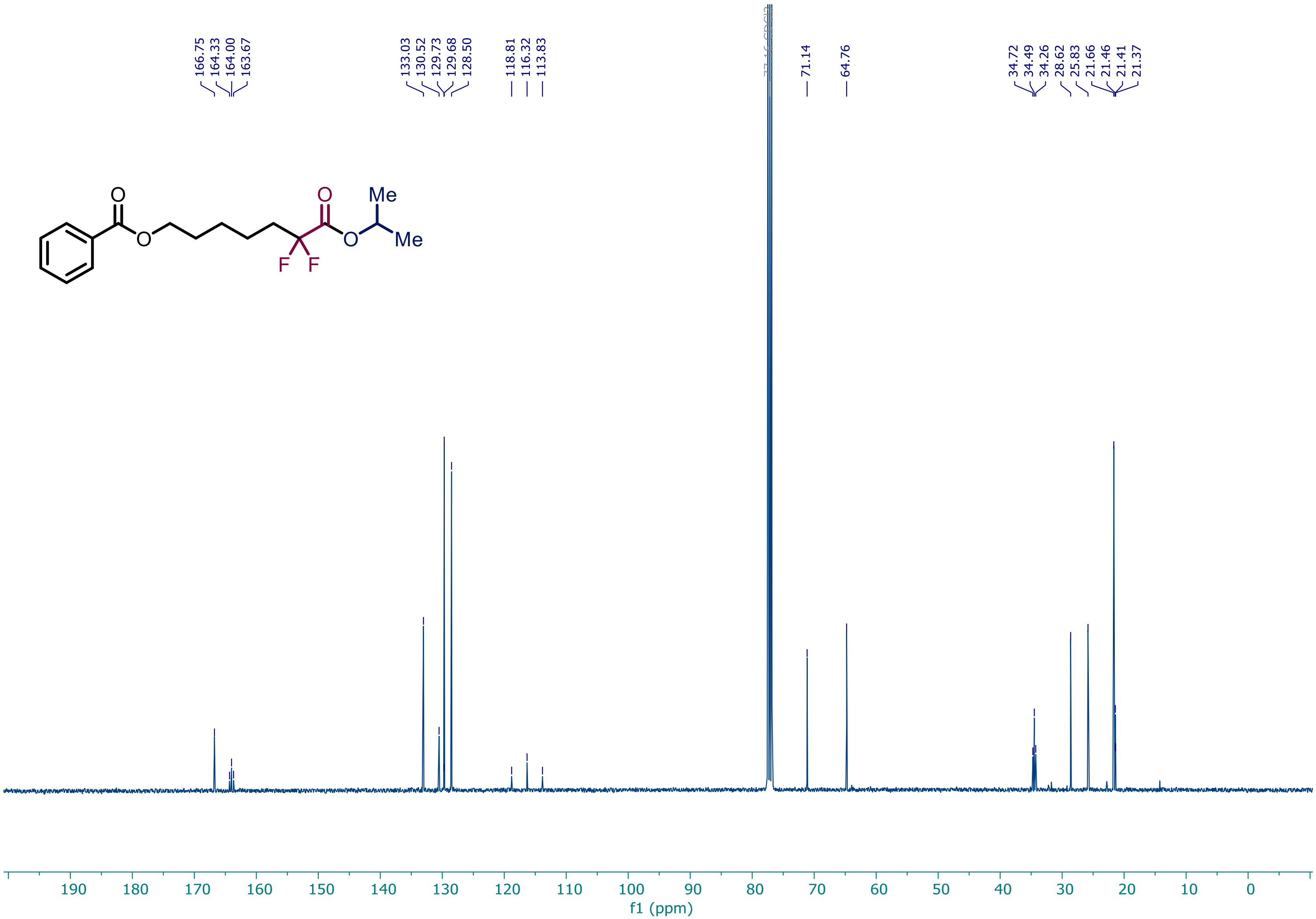

^{19}F NMR (376 MHz, $CDCl_3$) of **31**

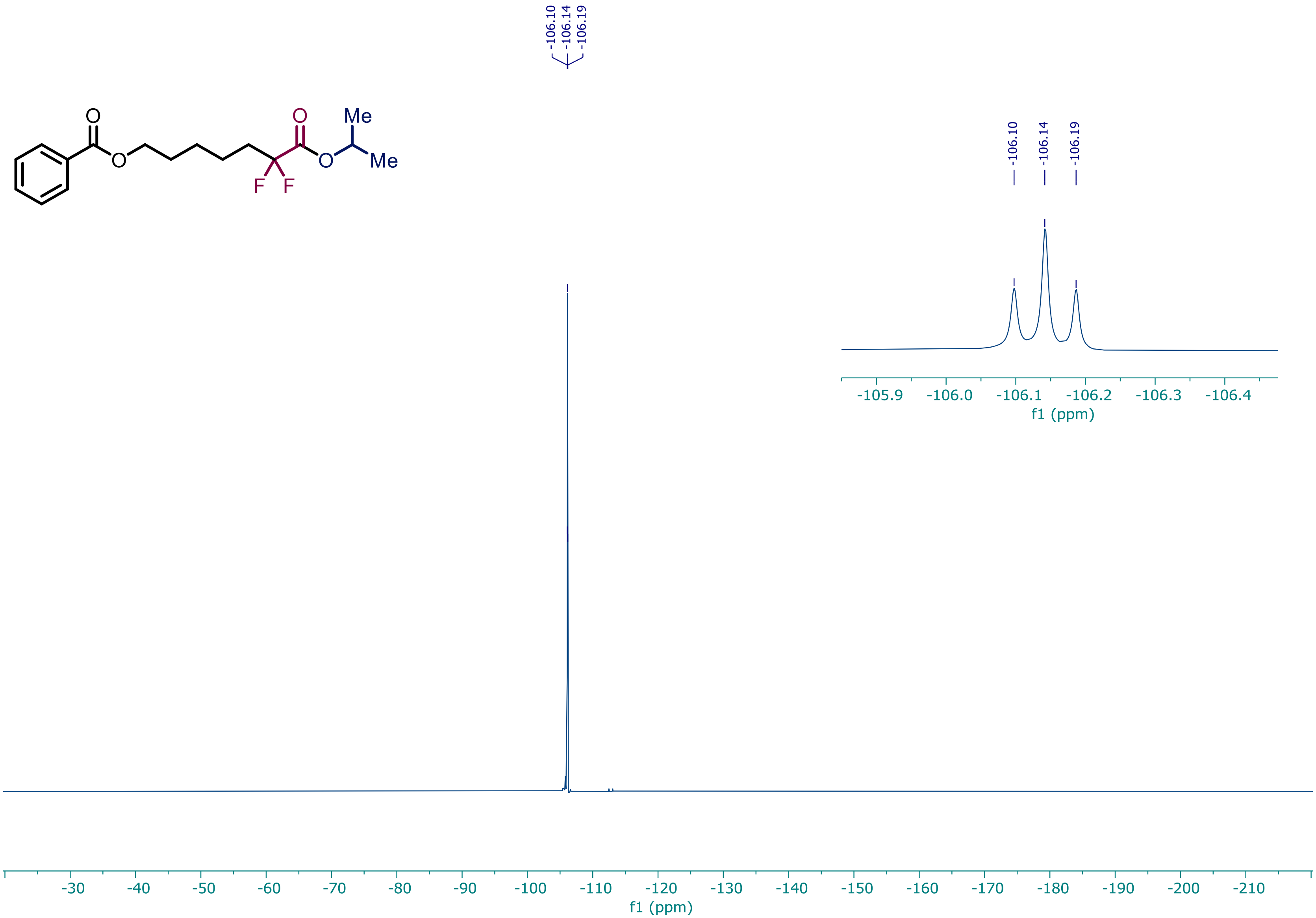

^{1}H NMR (400 MHz, $CDCl_3$) of **32**

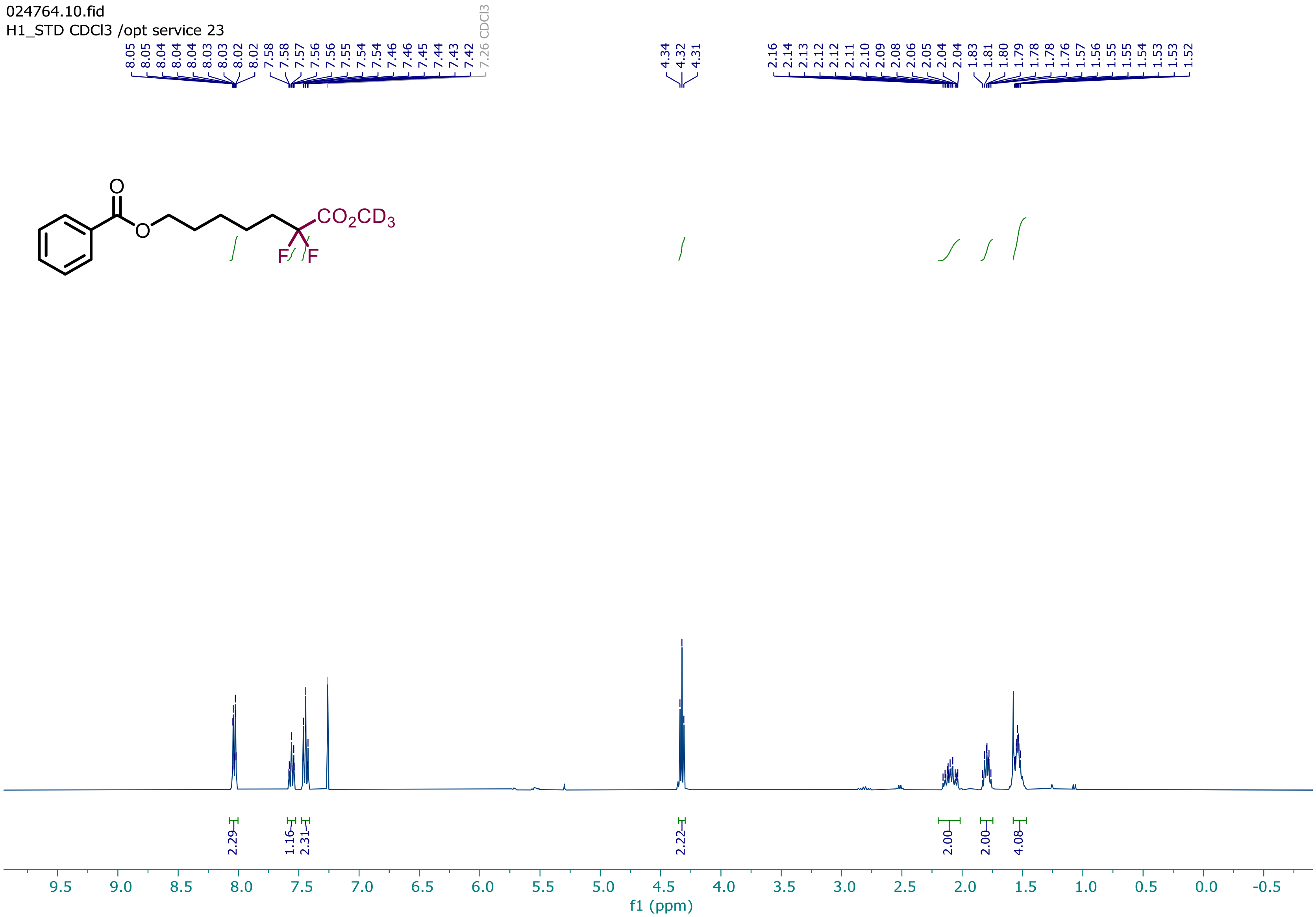

^{13}C NMR (101 MHz, $CDCl_3$) of **32**

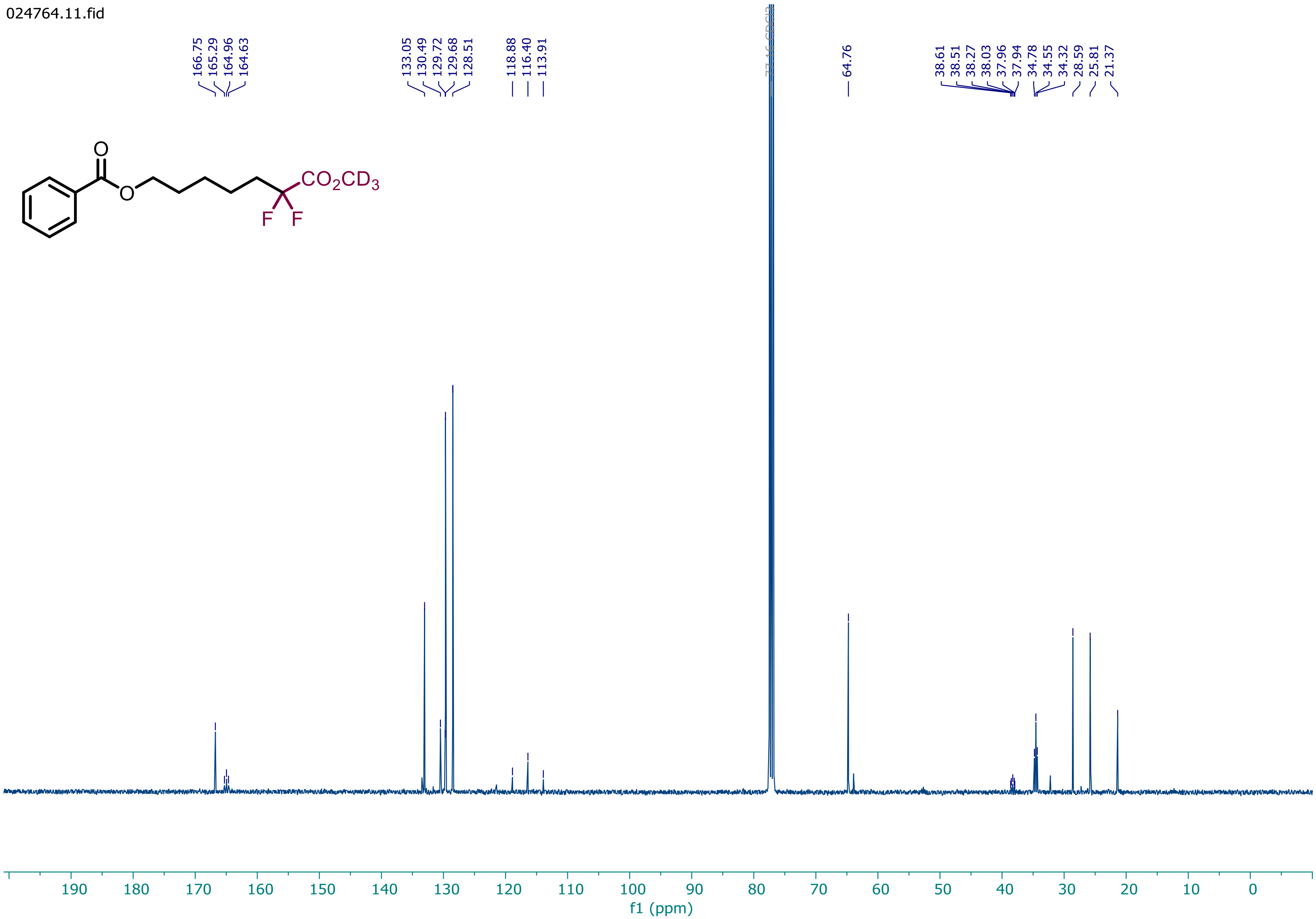

^{19}F NMR (282 MHz, $CDCl_3$) of **32**

GC_085195.3.fid
rg-sn-619-tt5,6

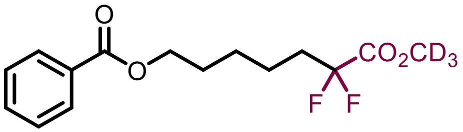

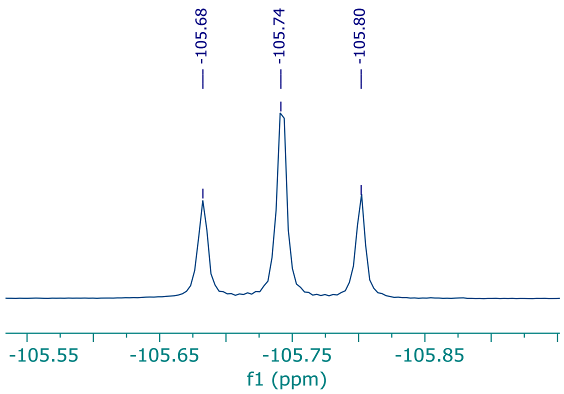

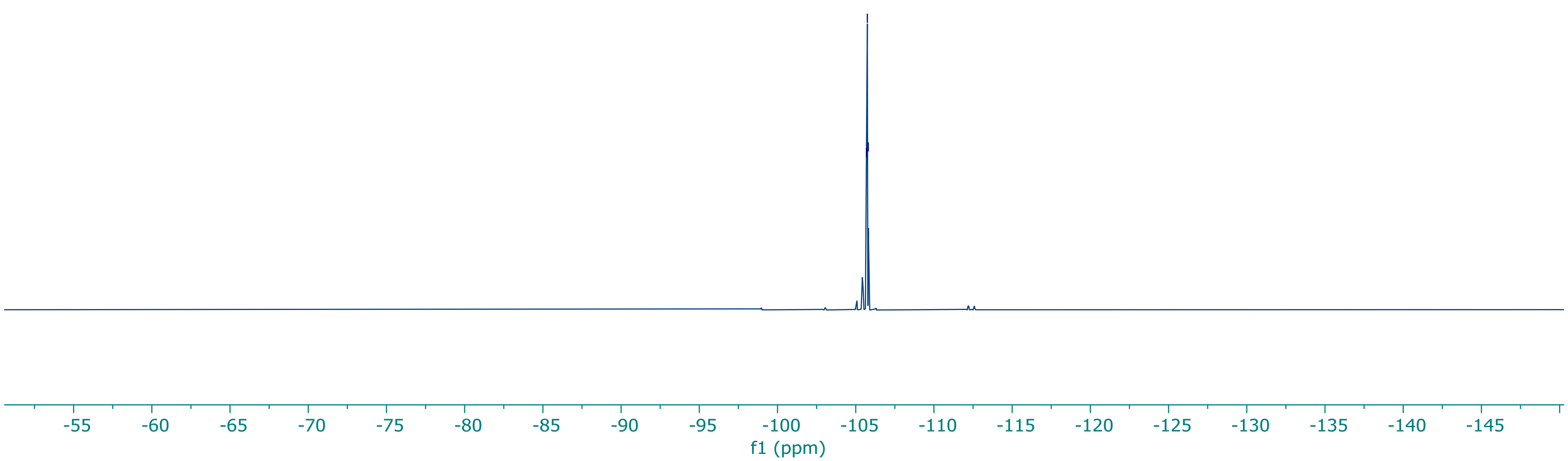

^{1}H NMR (300 MHz, $CDCl_3$) of **33**

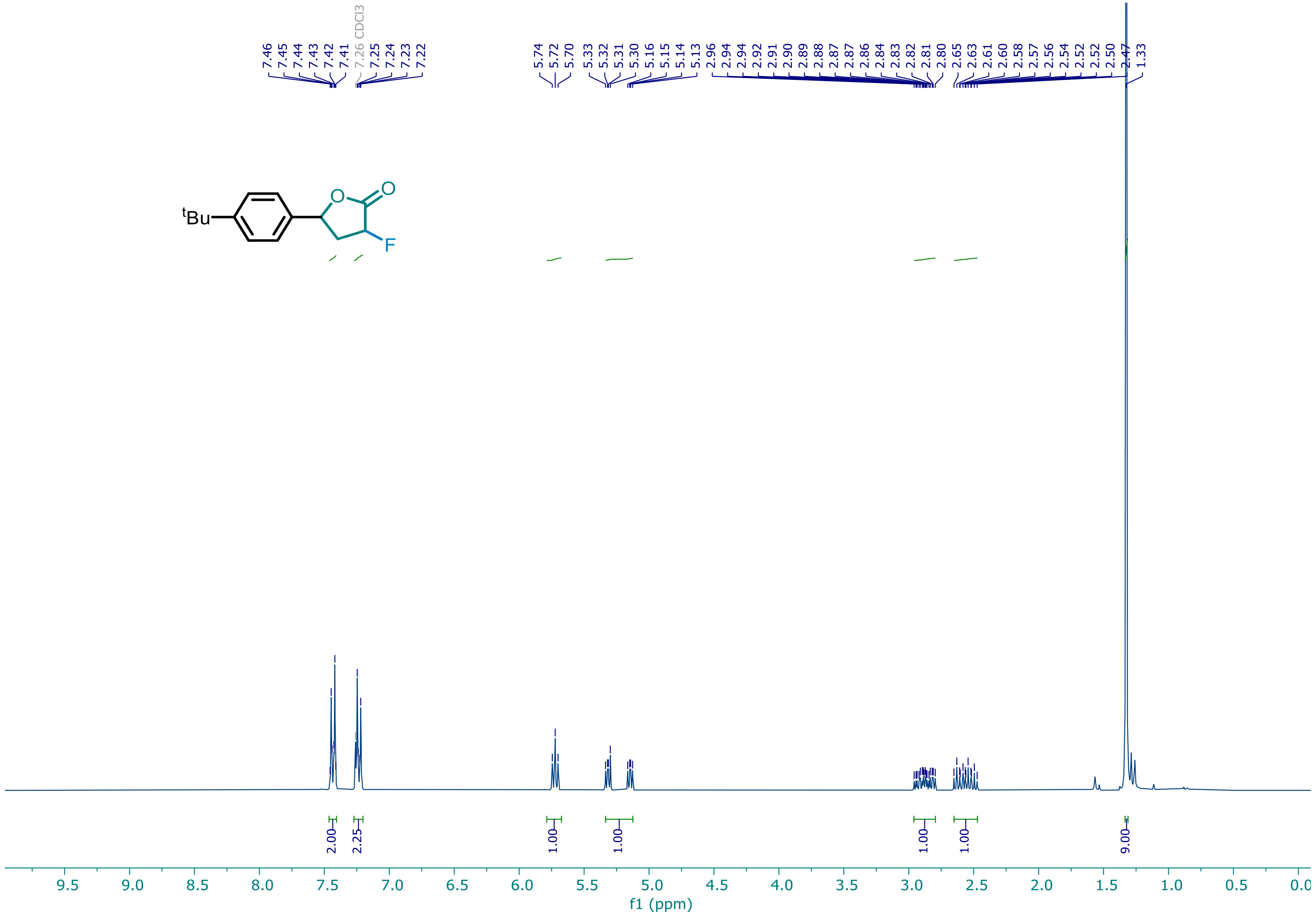

^{13}C NMR (75 MHz, $CDCl_3$) of **33**

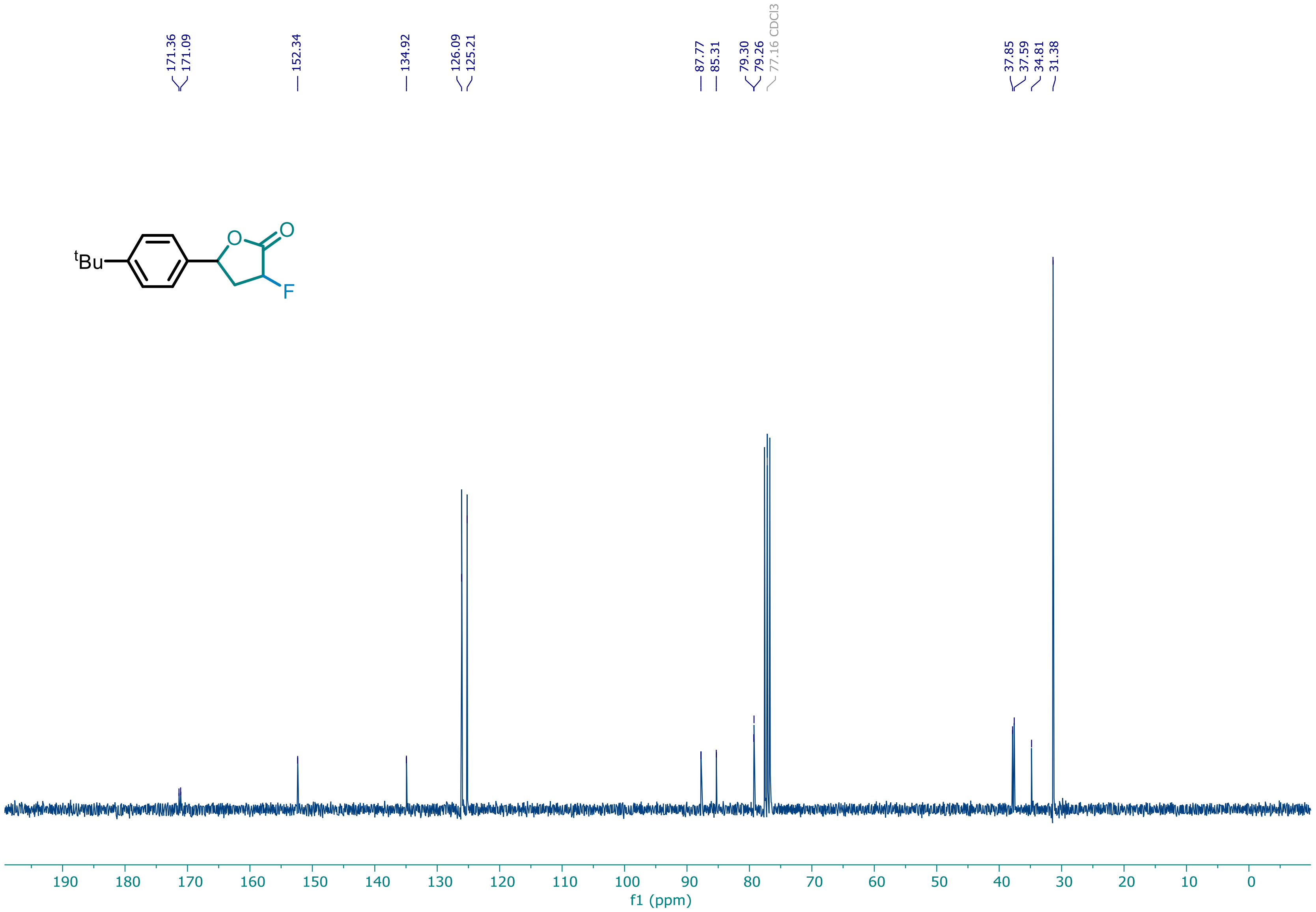

^{19}F NMR (282 MHz, $CDCl_3$) of **33**

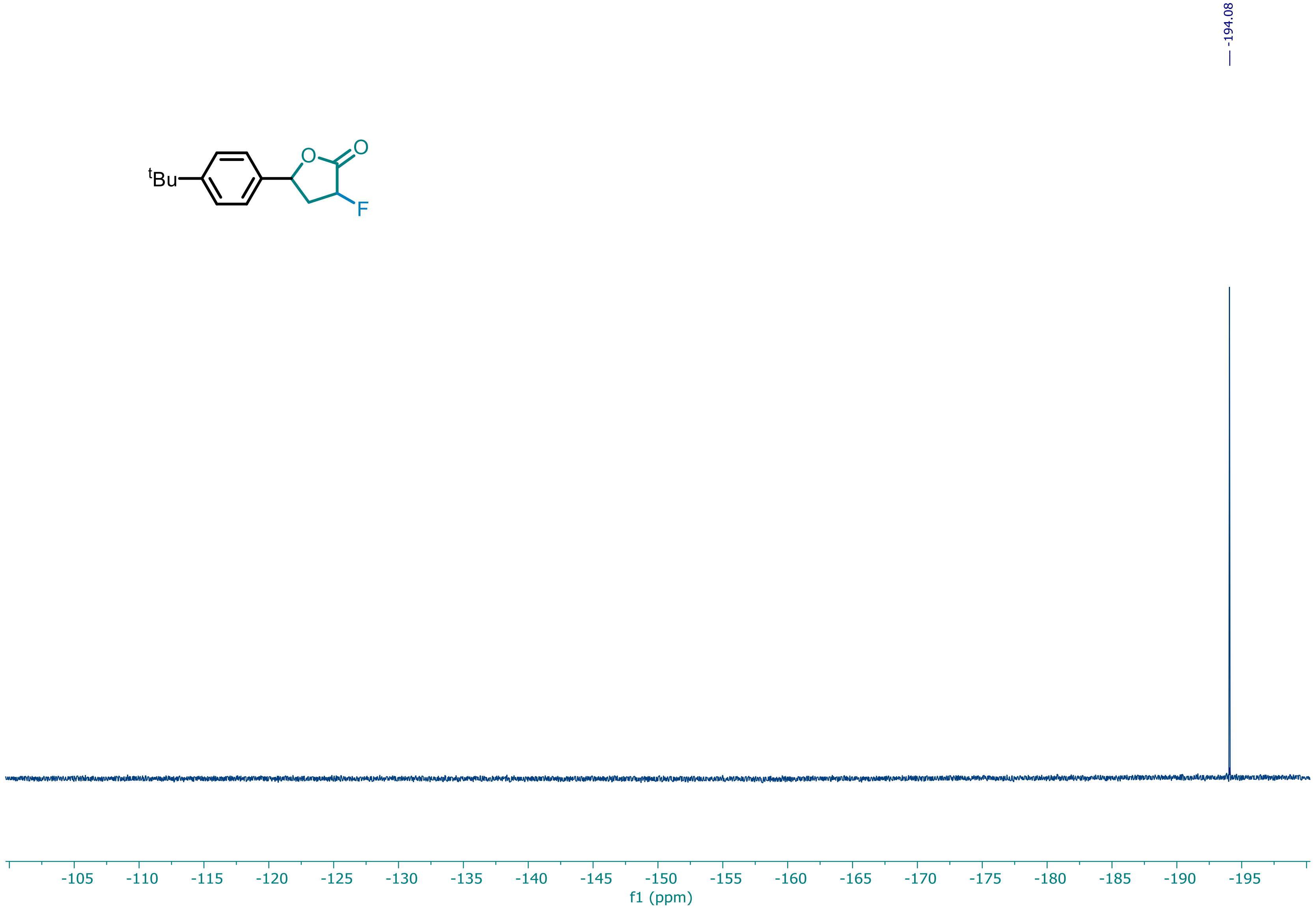

^{1}H NMR (300 MHz, $CDCl_3$) of **1**

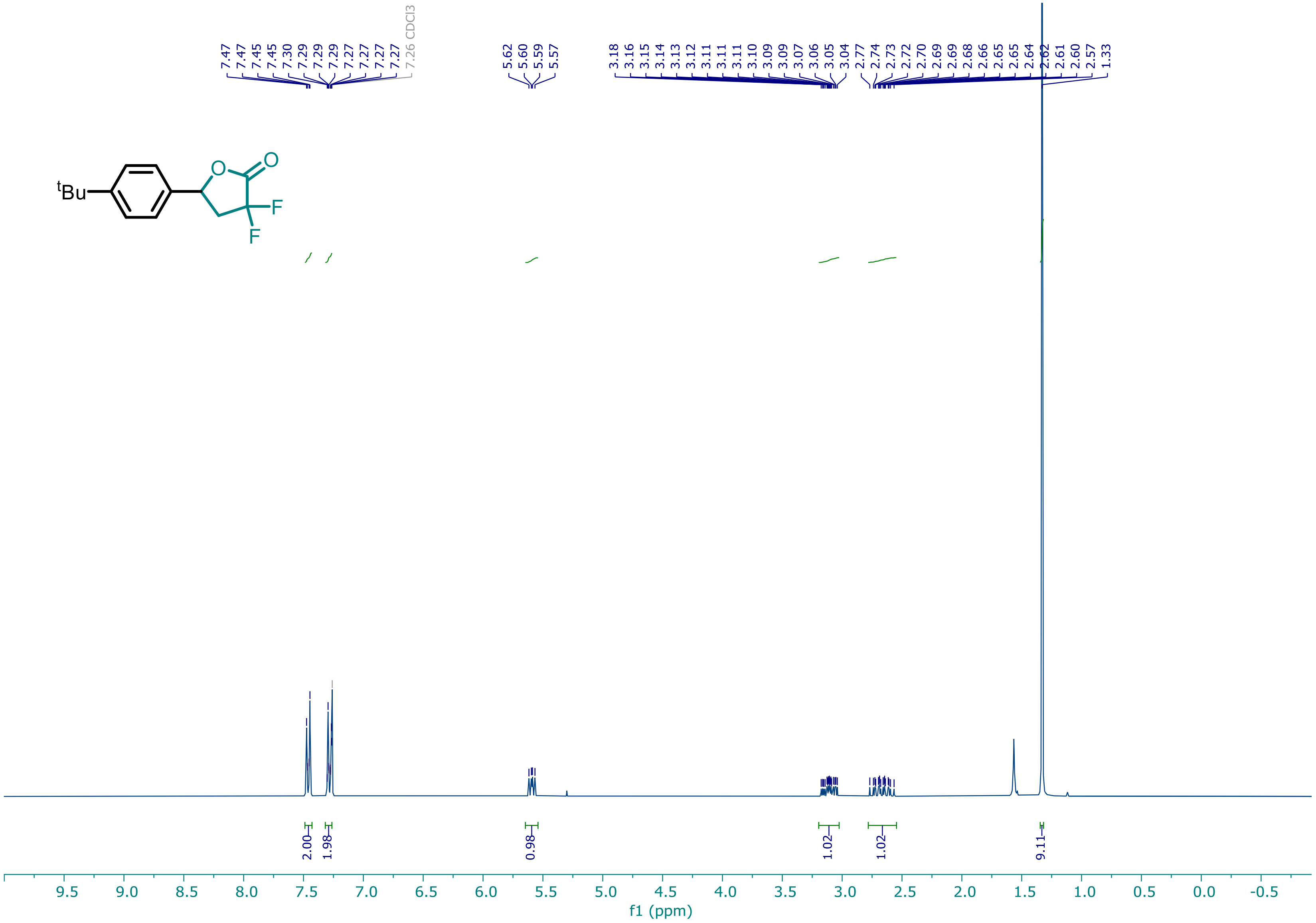

^{13}C NMR (126 MHz, $CDCl_3$) of **1**

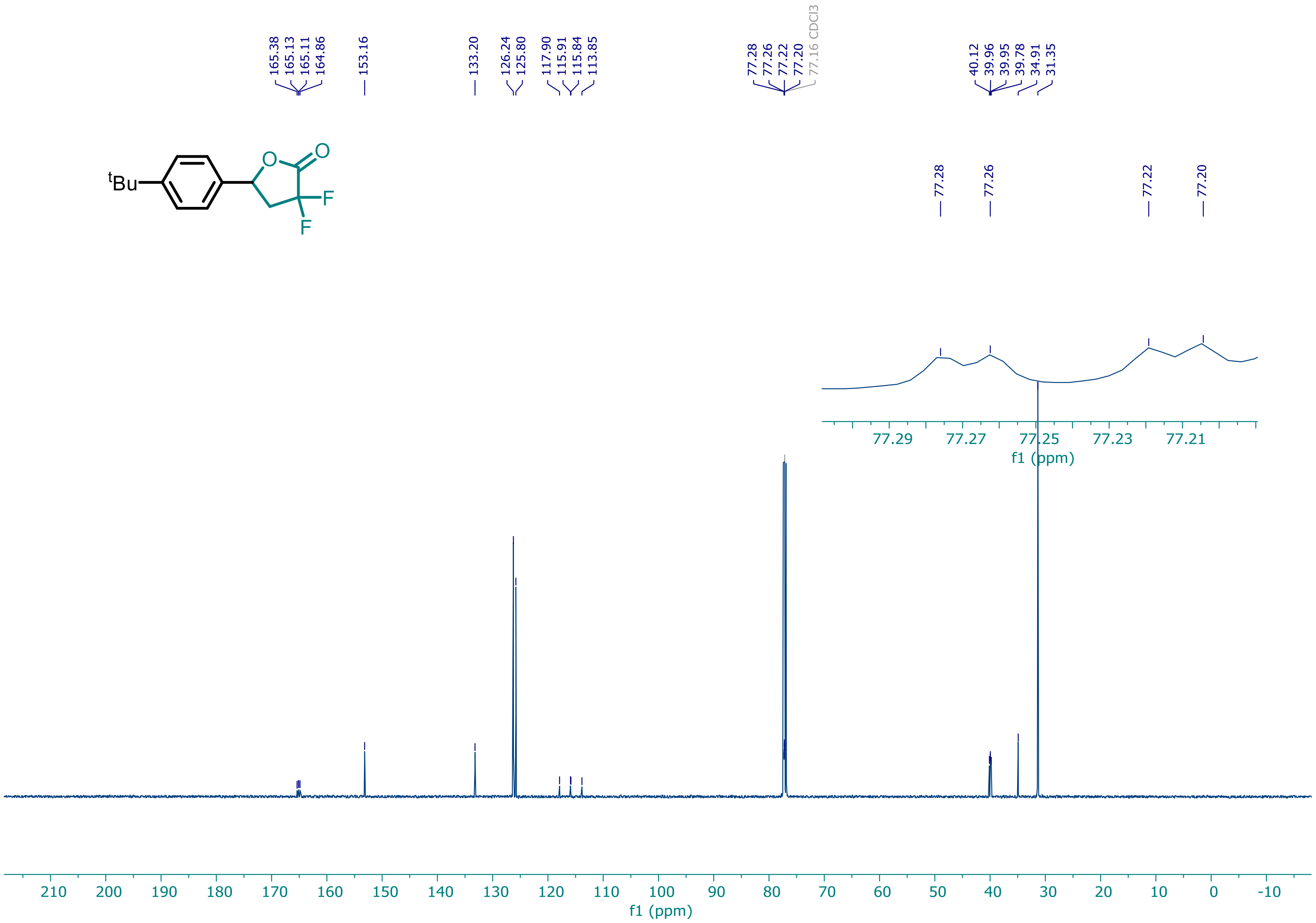

^{19}F NMR (282 MHz, $CDCl_3$) of **1**

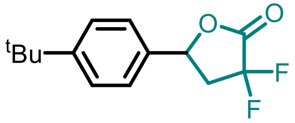

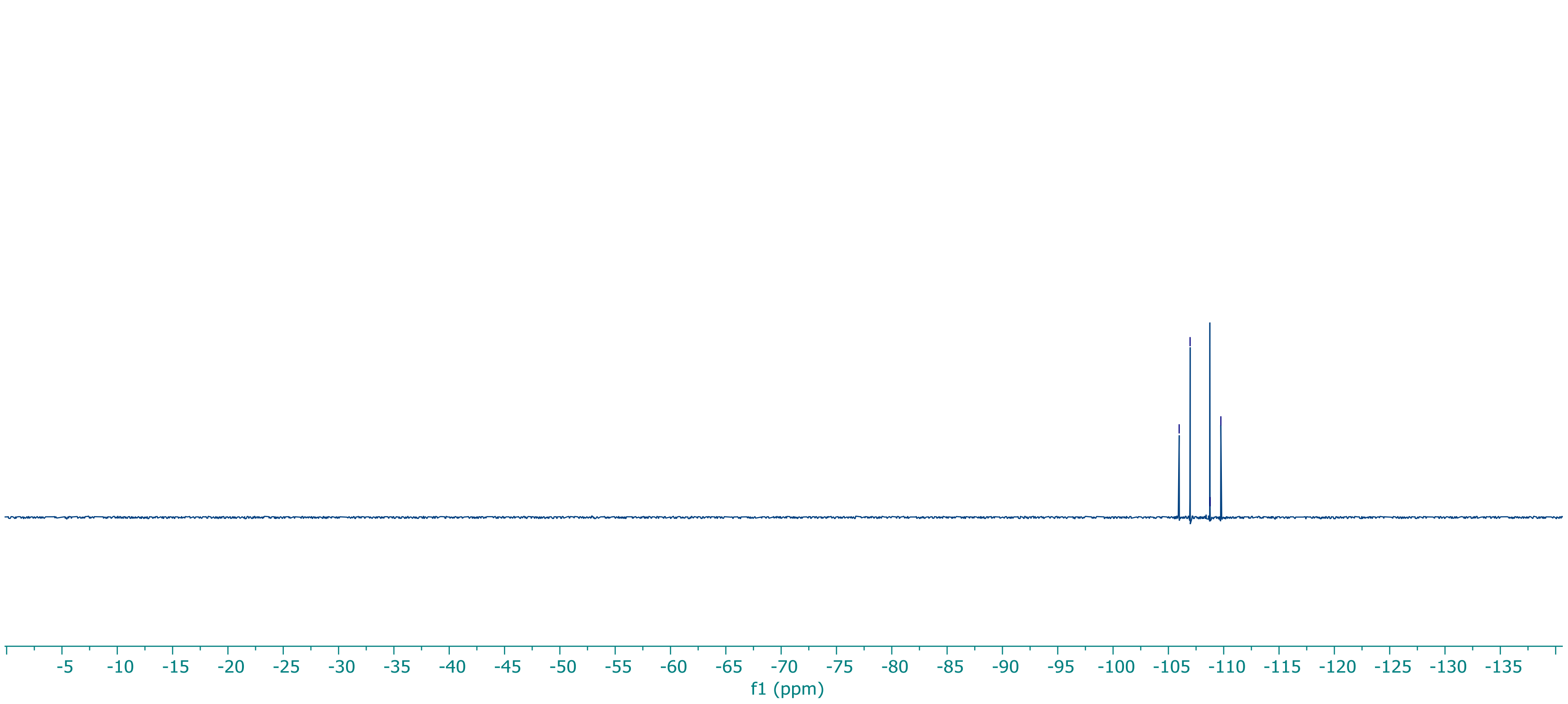

^{1}H NMR (500 MHz, $CDCl_3$) of **34**

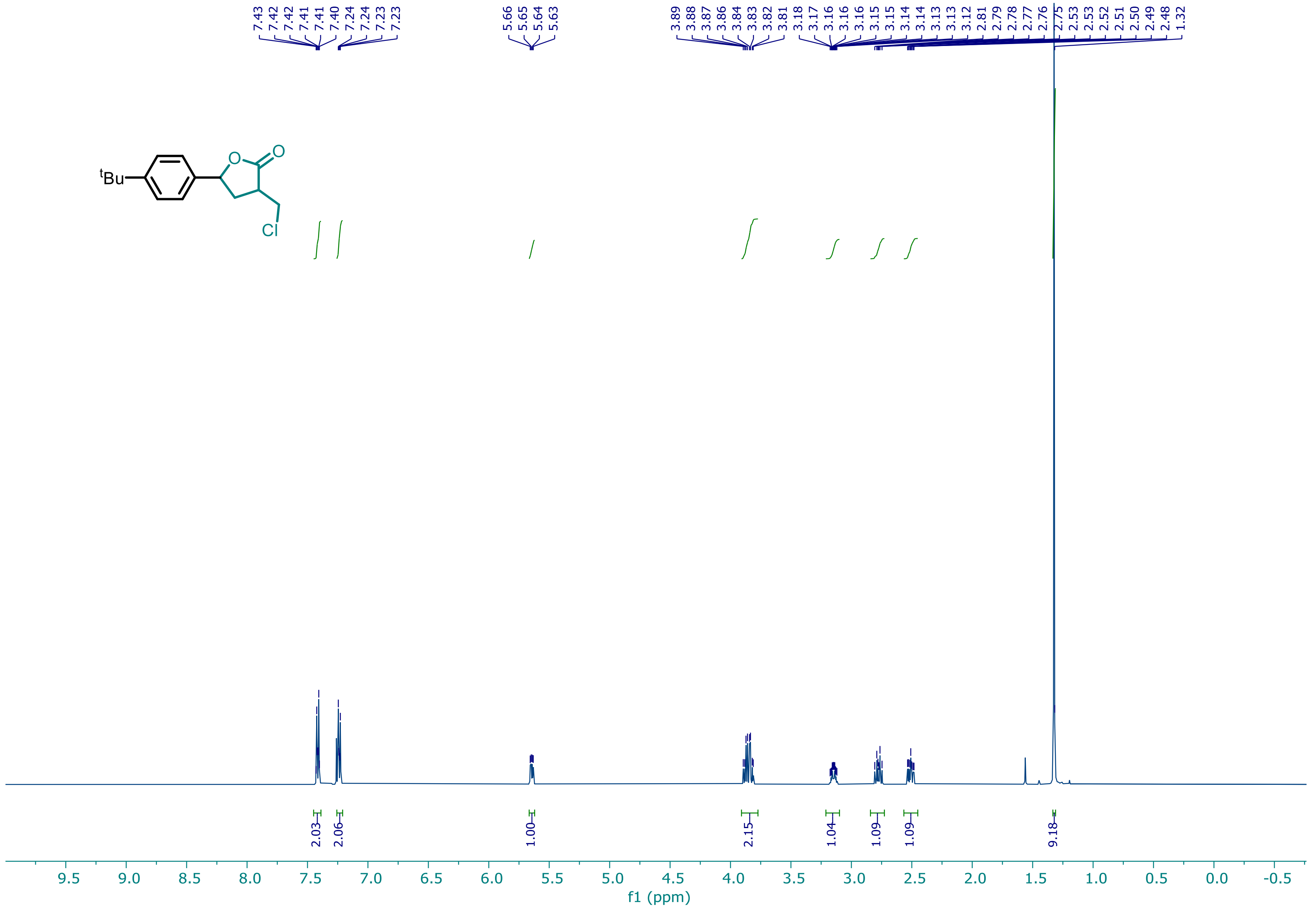

^{13}C NMR (126 MHz, $CDCl_3$) of **34**

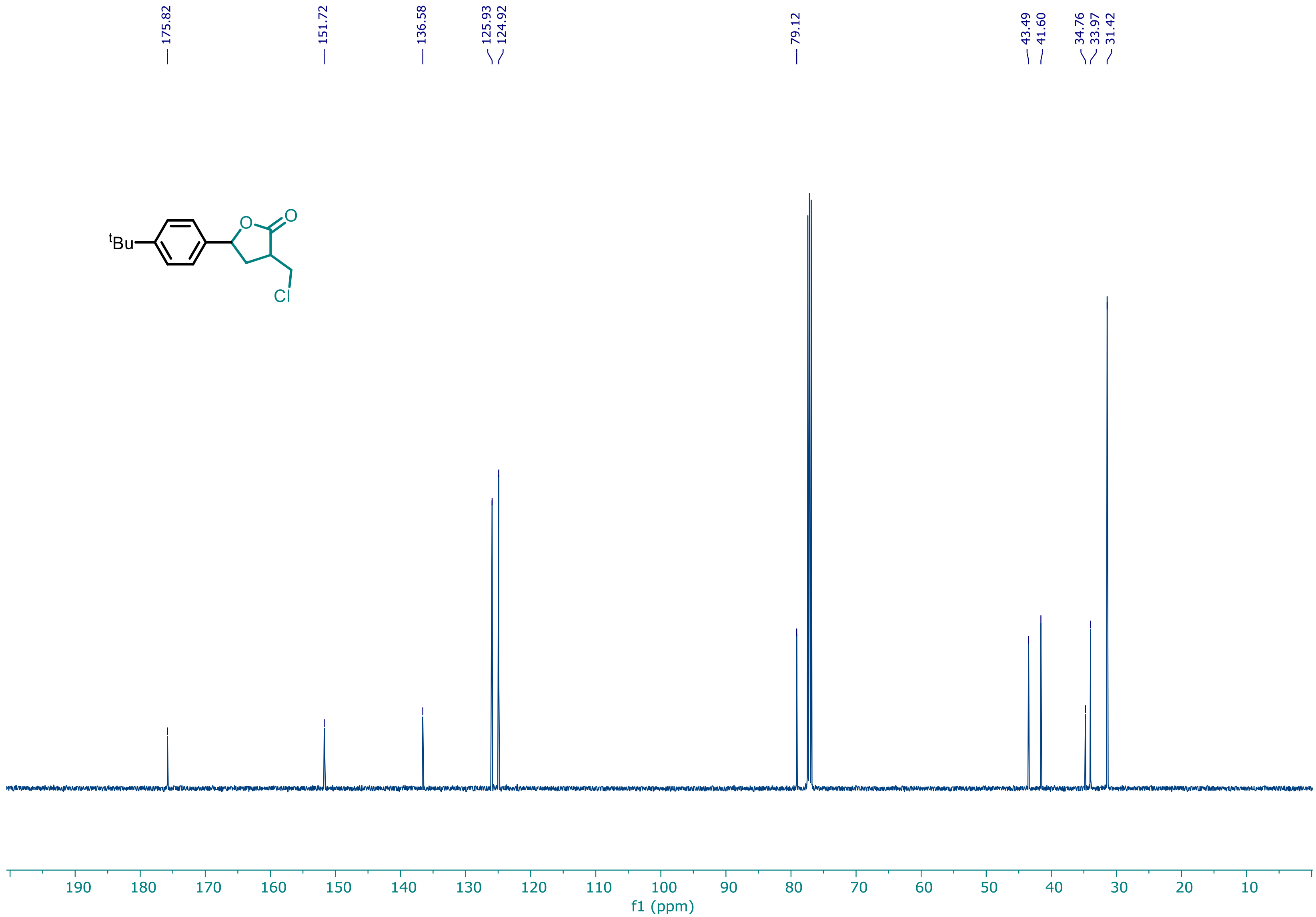

^{1}H NMR (500 MHz, $CDCl_3$) of **35**

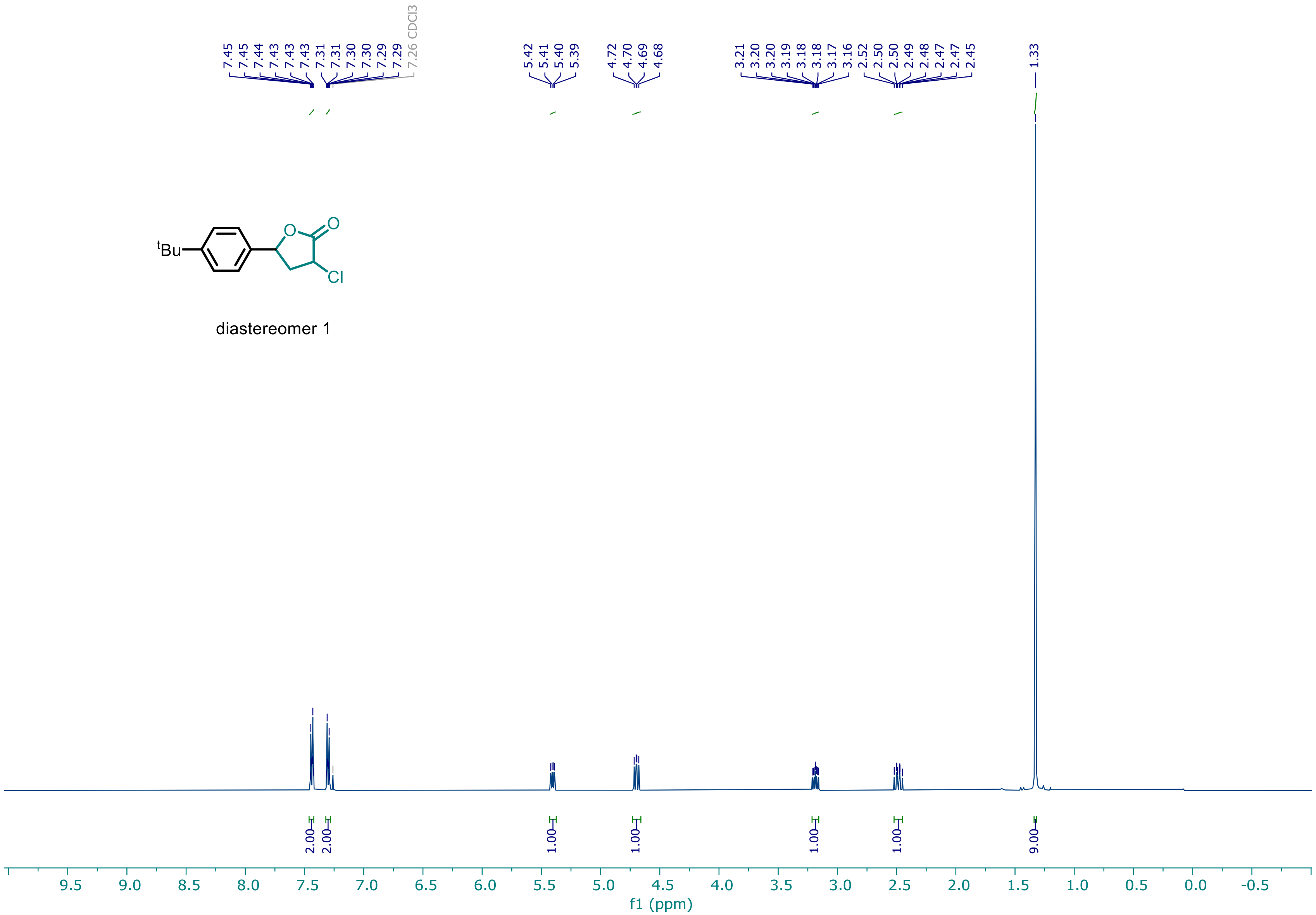

^{13}C NMR (126 MHz, $CDCl_3$) of **35**

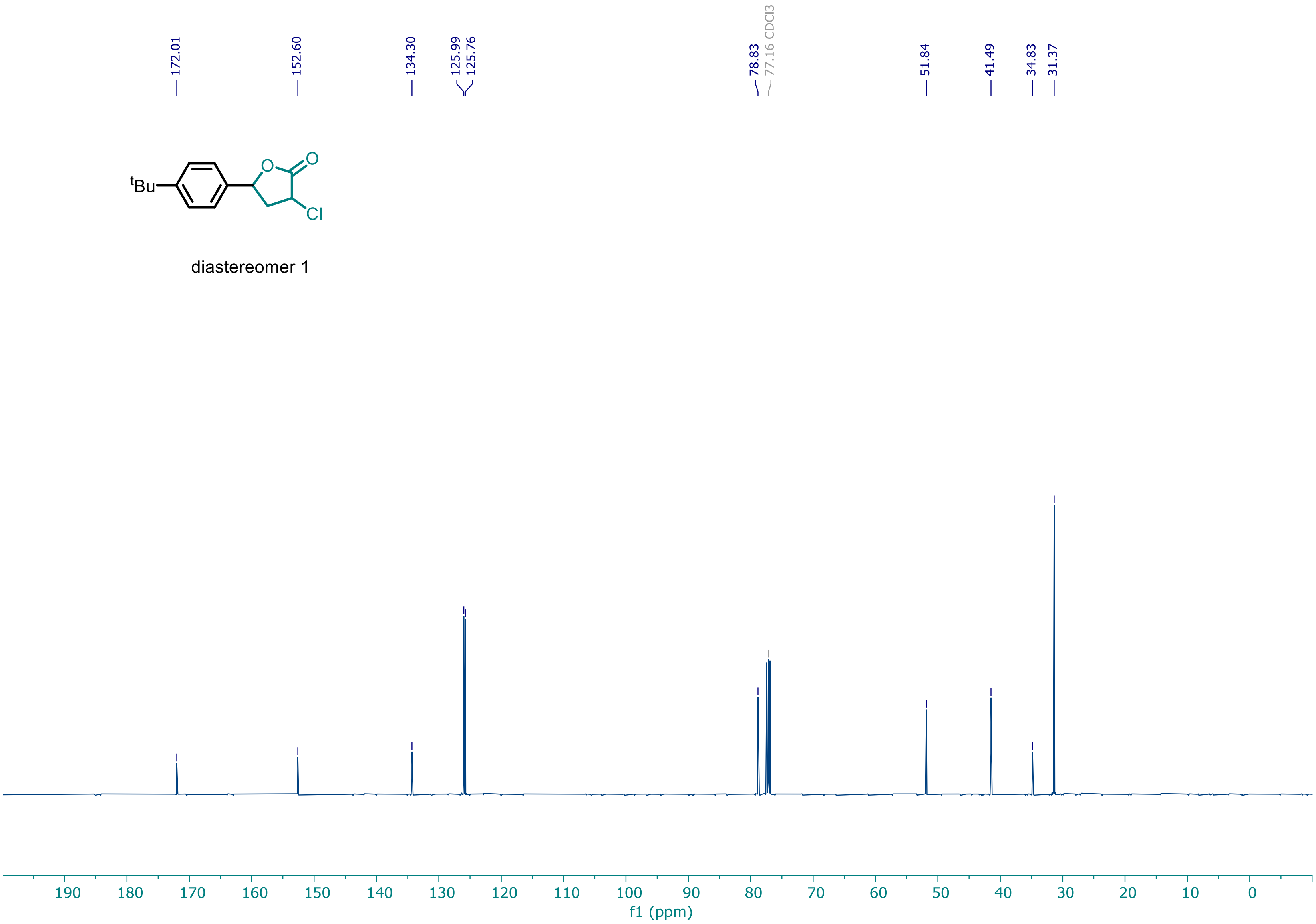

^{1}H NMR (500 MHz, $CDCl_3$) of **35**

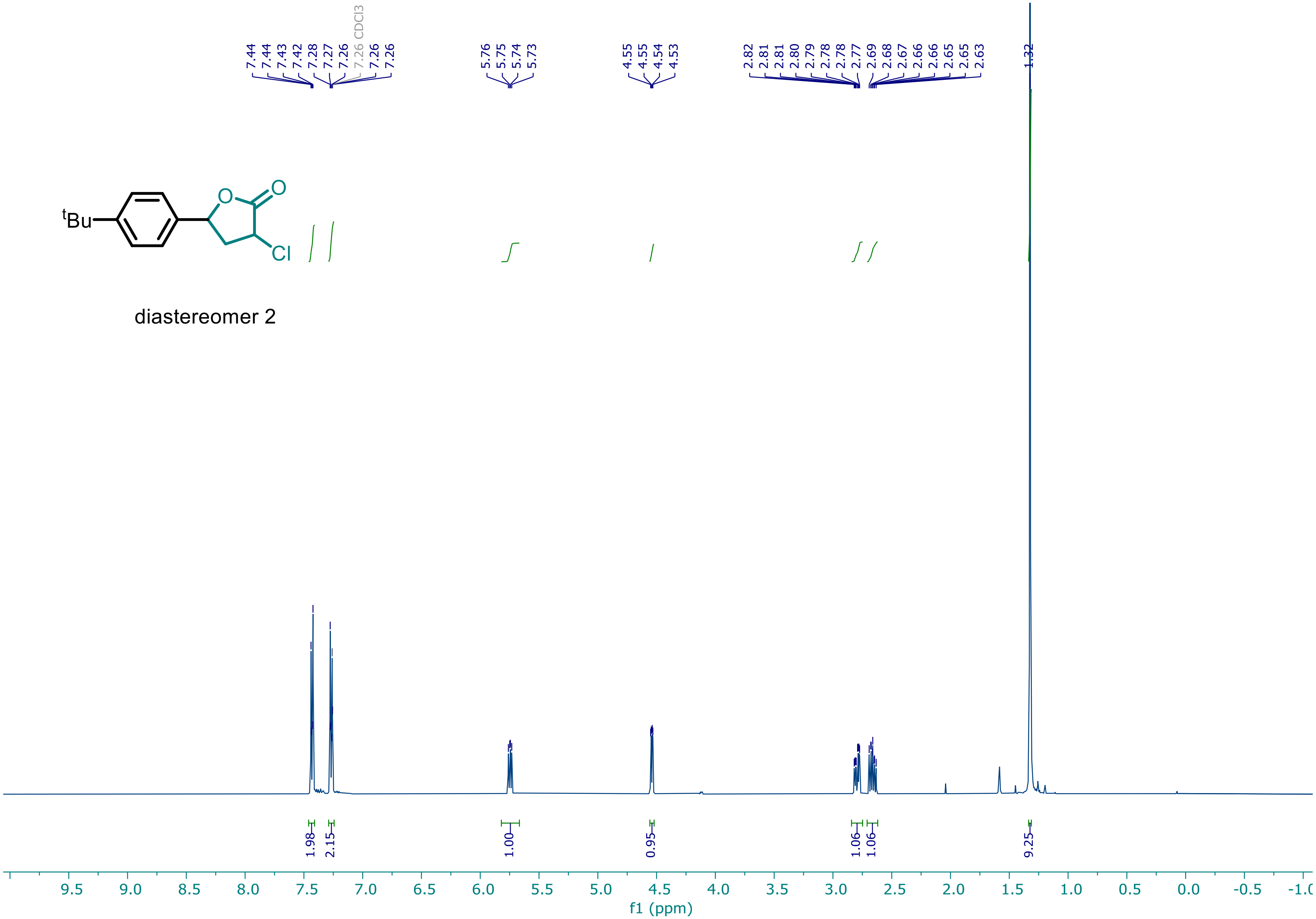

^{13}C NMR (126 MHz, $CDCl_3$) of **35**

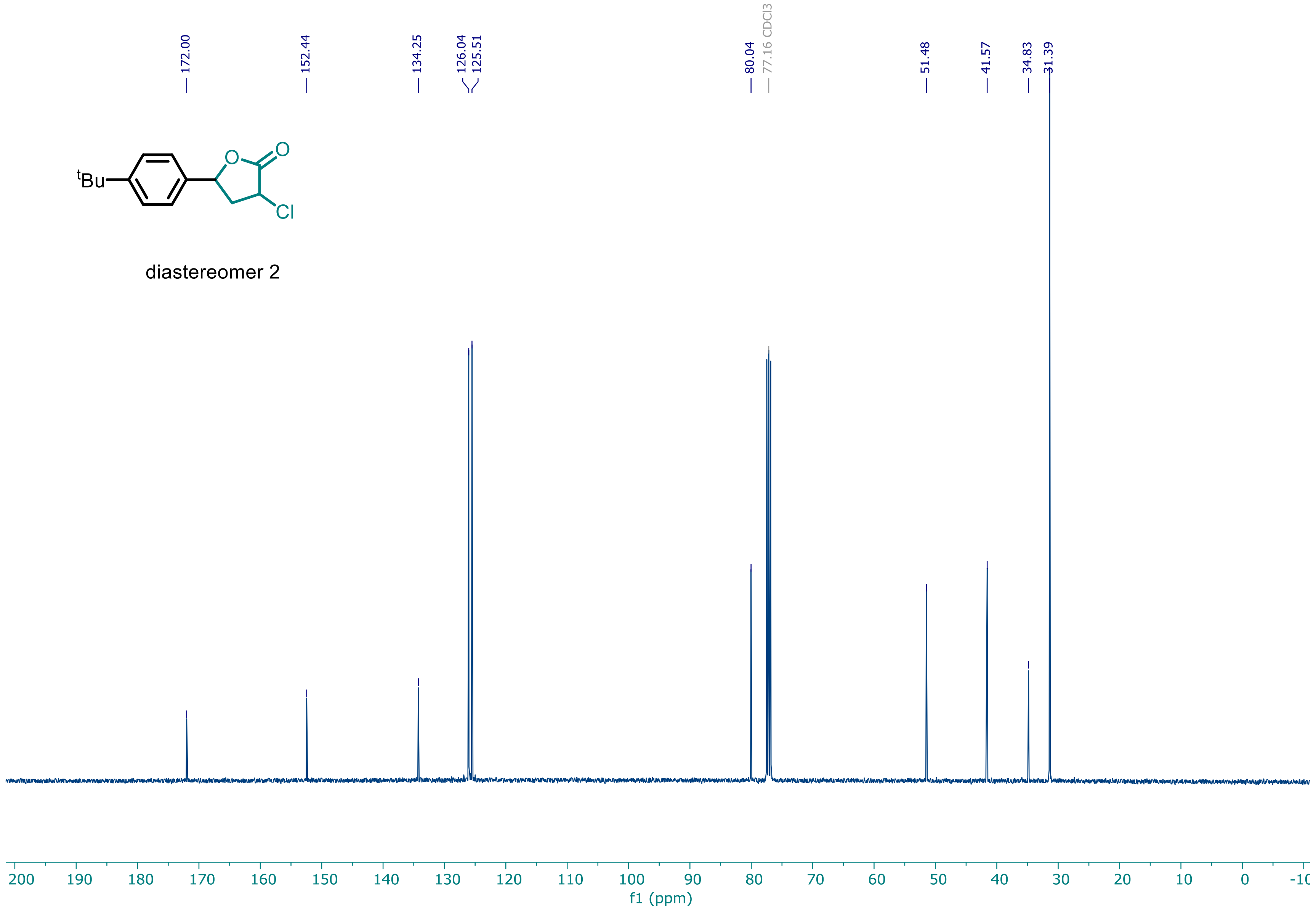

^{1}H NMR (300 MHz, $CDCl_3$) of **36**

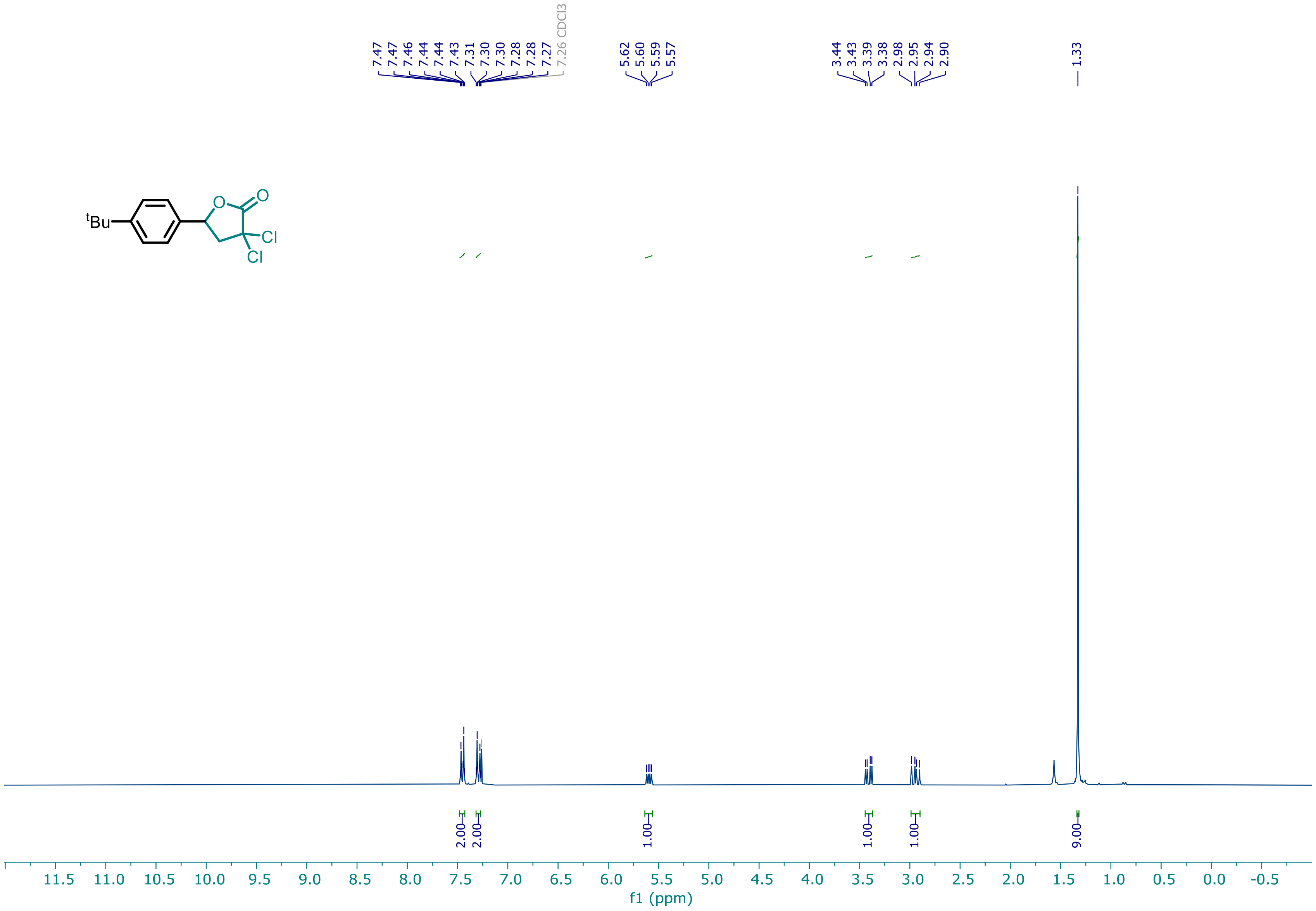

^{13}C NMR (75 MHz, $CDCl_3$) of **36**

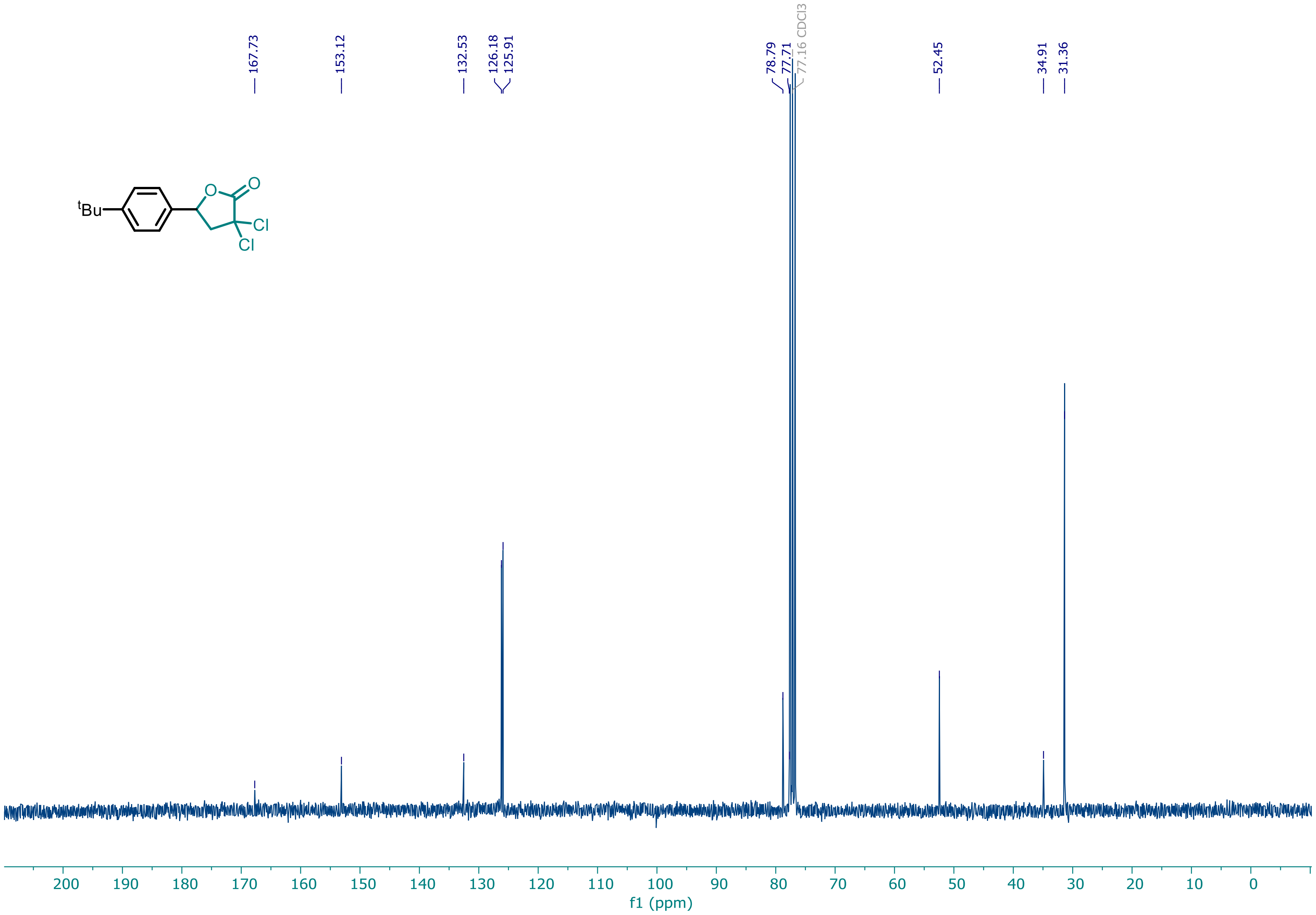

^{1}H NMR (300 MHz, $CDCl_3$) of **37**

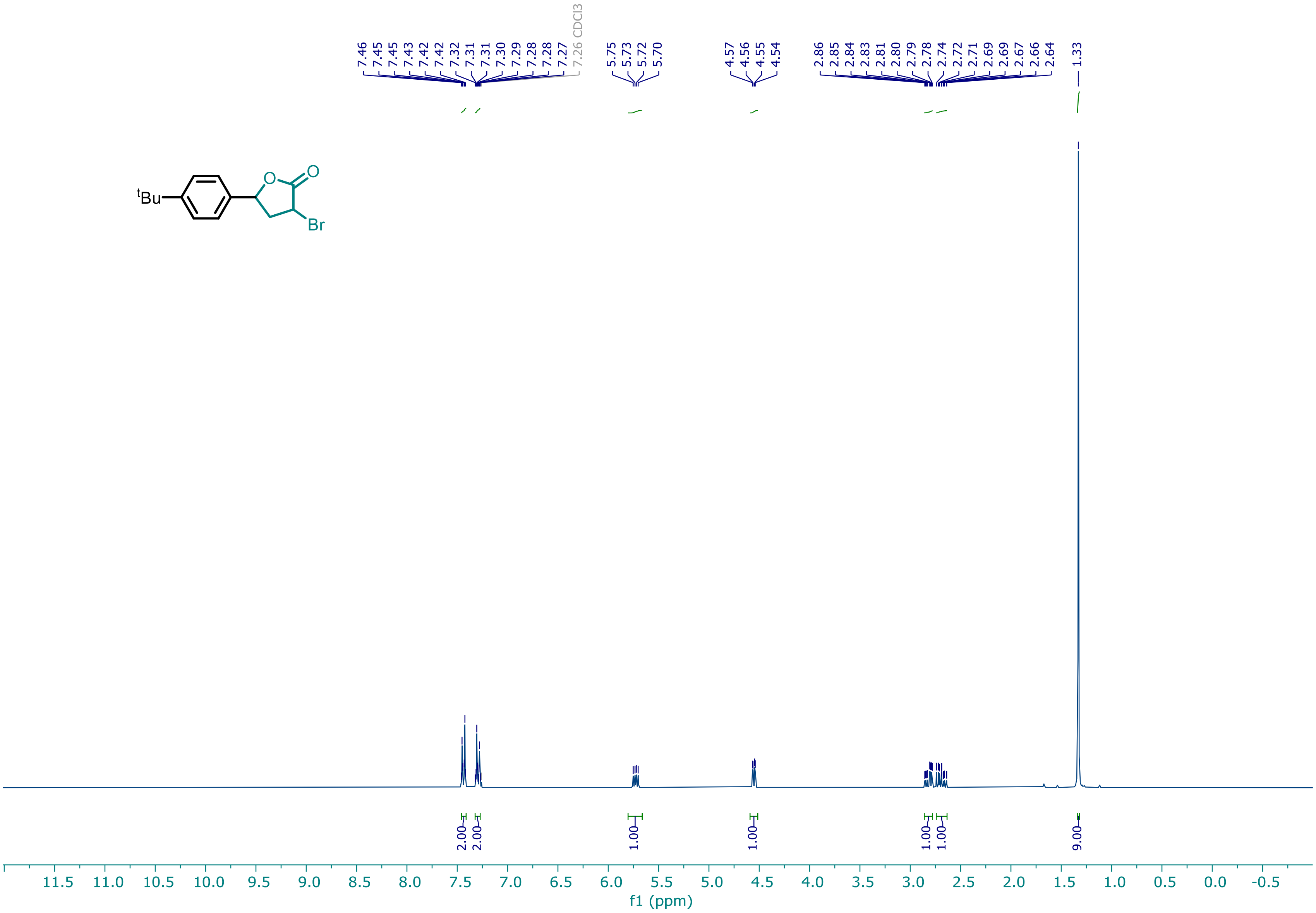

^{13}C NMR (75 MHz, $CDCl_3$) of **37**

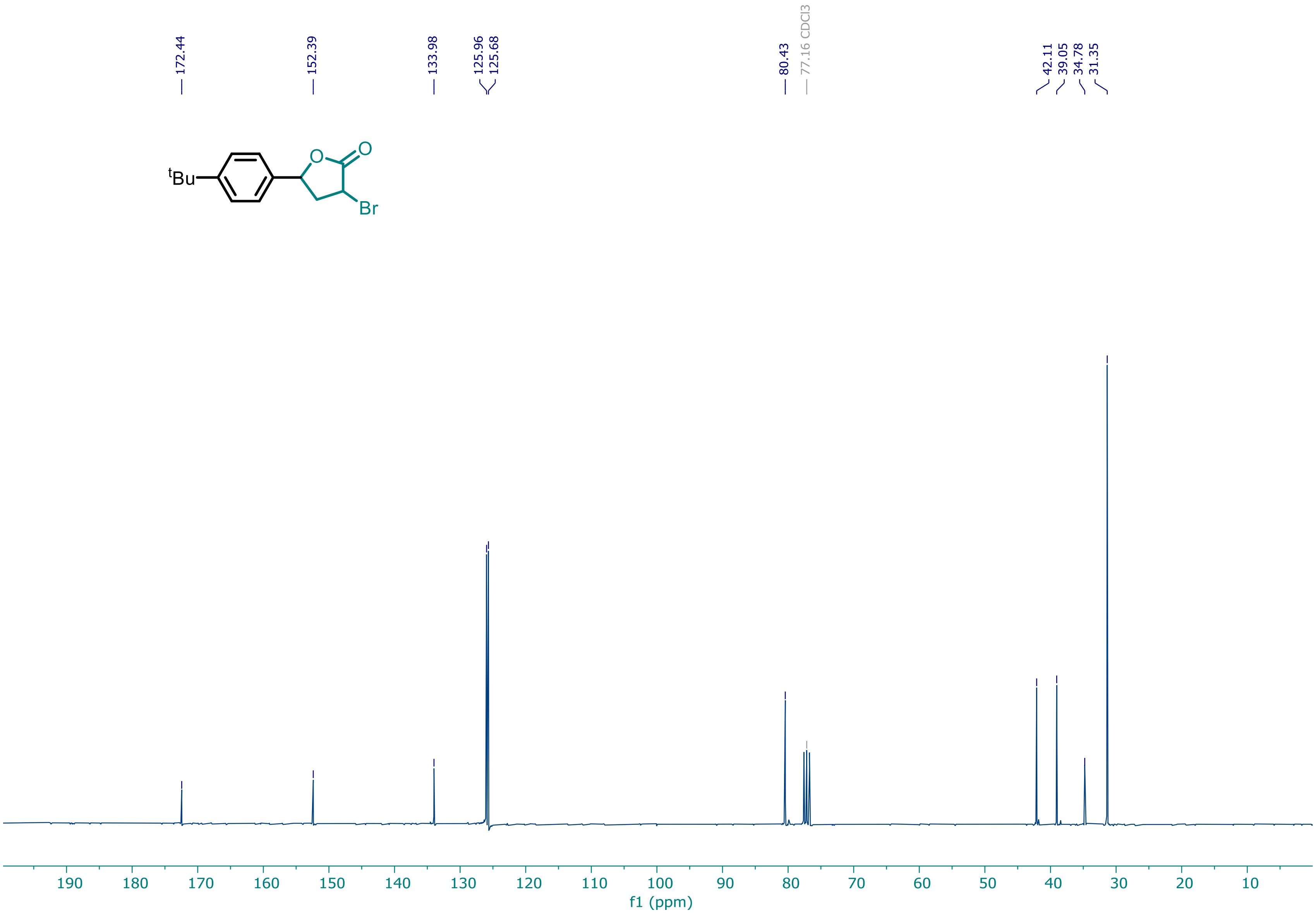

^{1}H NMR (500 MHz, $CDCl_3$) of **38**

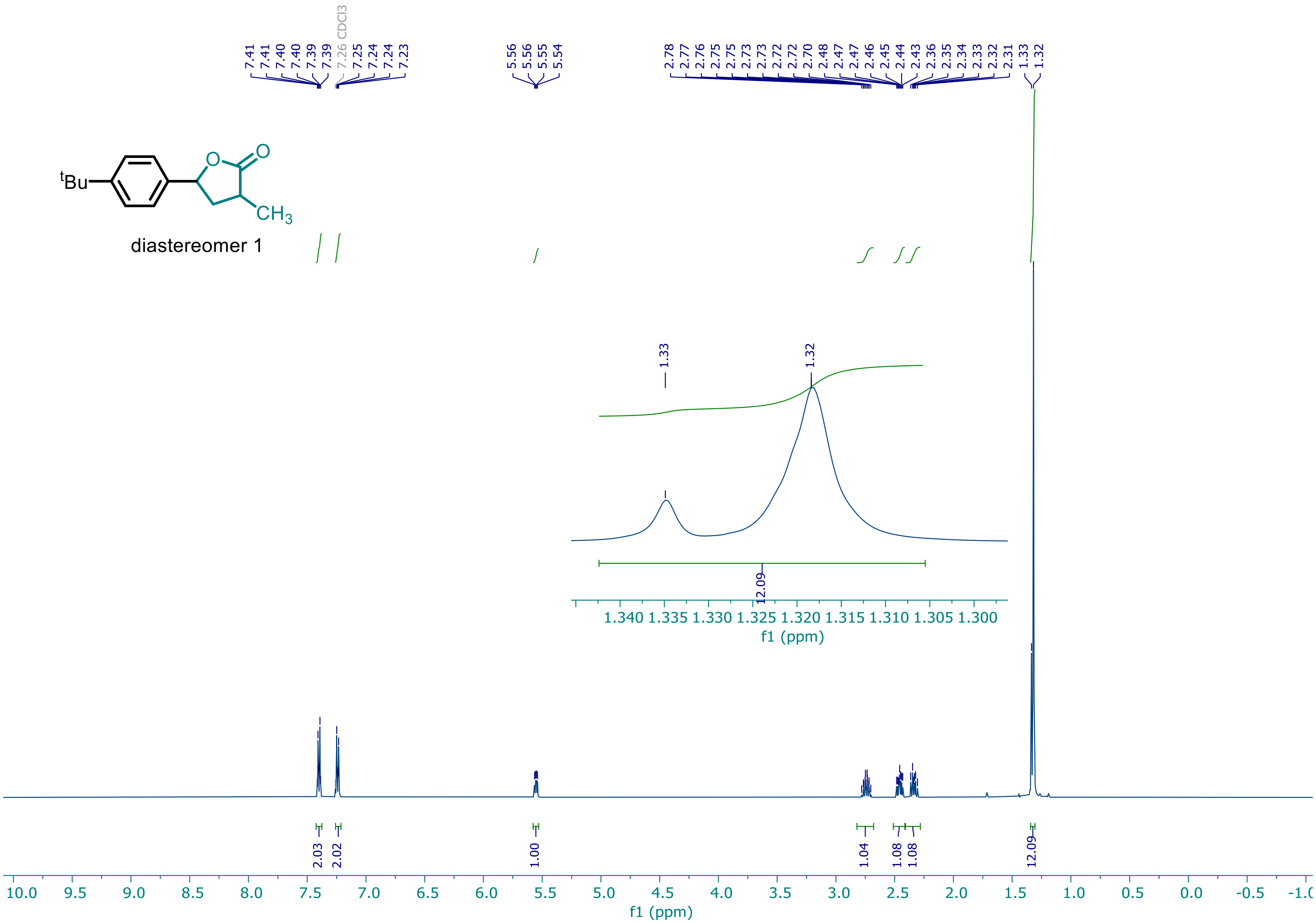

^{13}C NMR (126 MHz, $CDCl_3$) of **38**

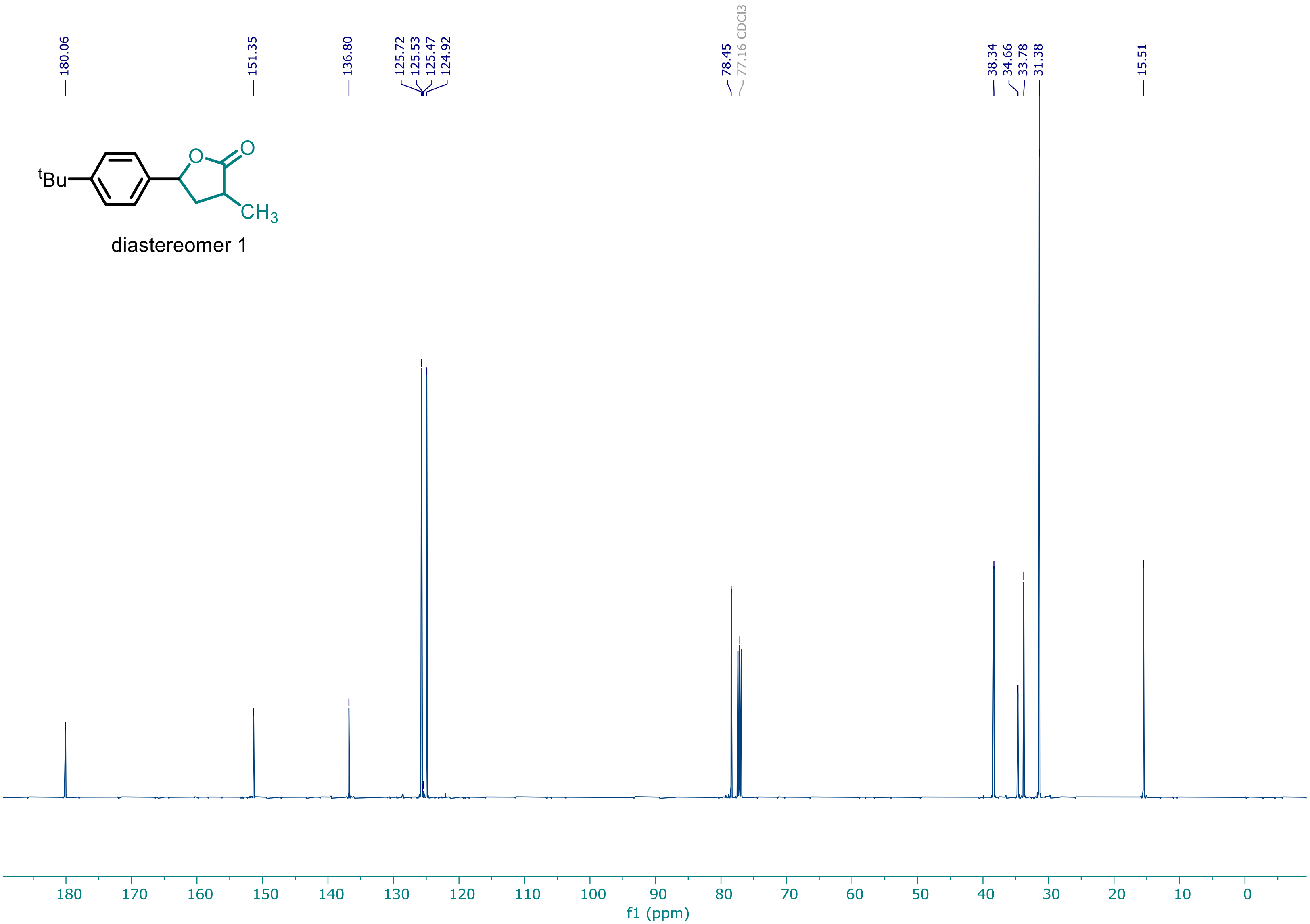

^{1}H NMR (500 MHz, $CDCl_3$) of **38**

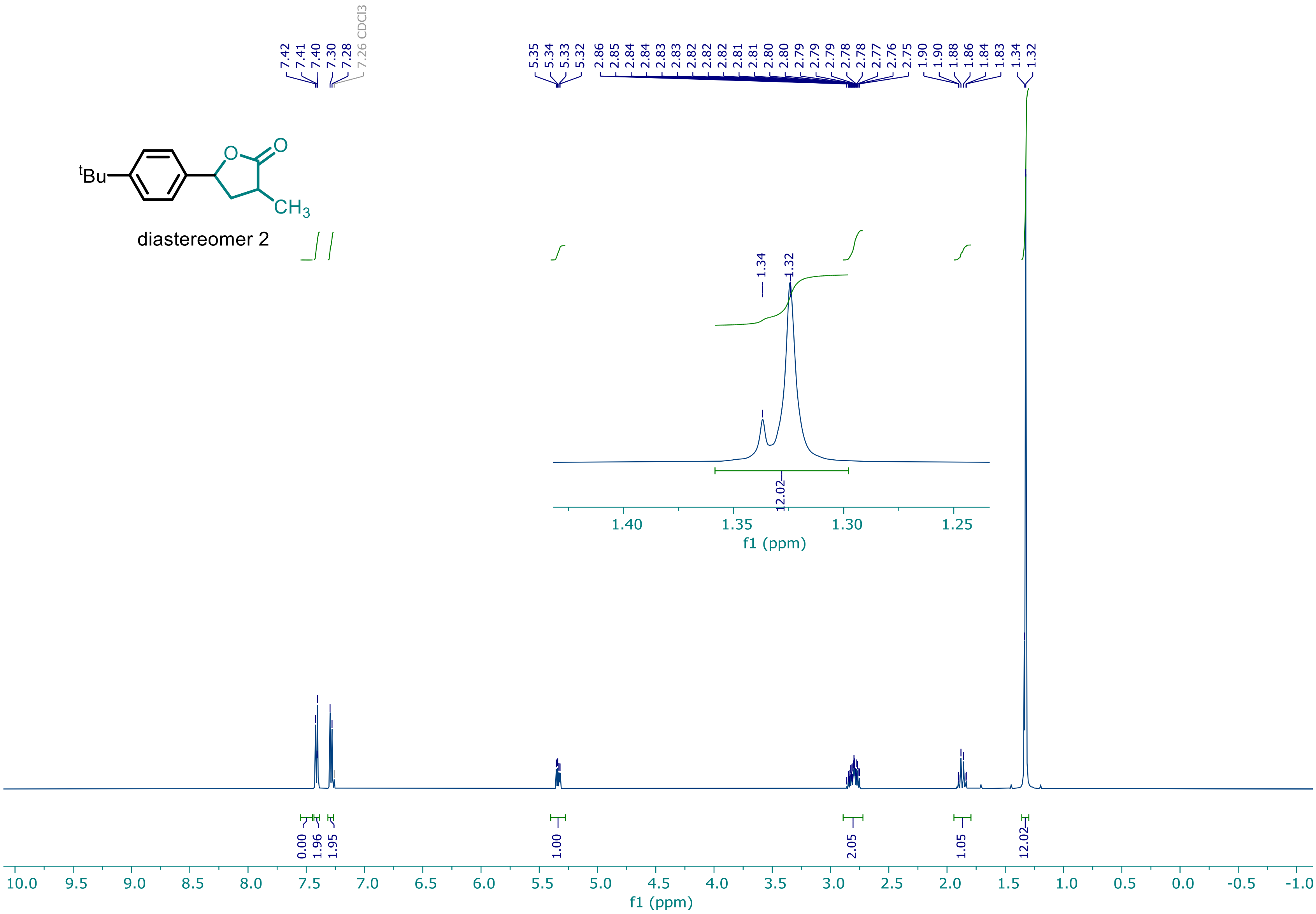

^{13}C NMR (126 MHz, CDCl$_3$) of **38**

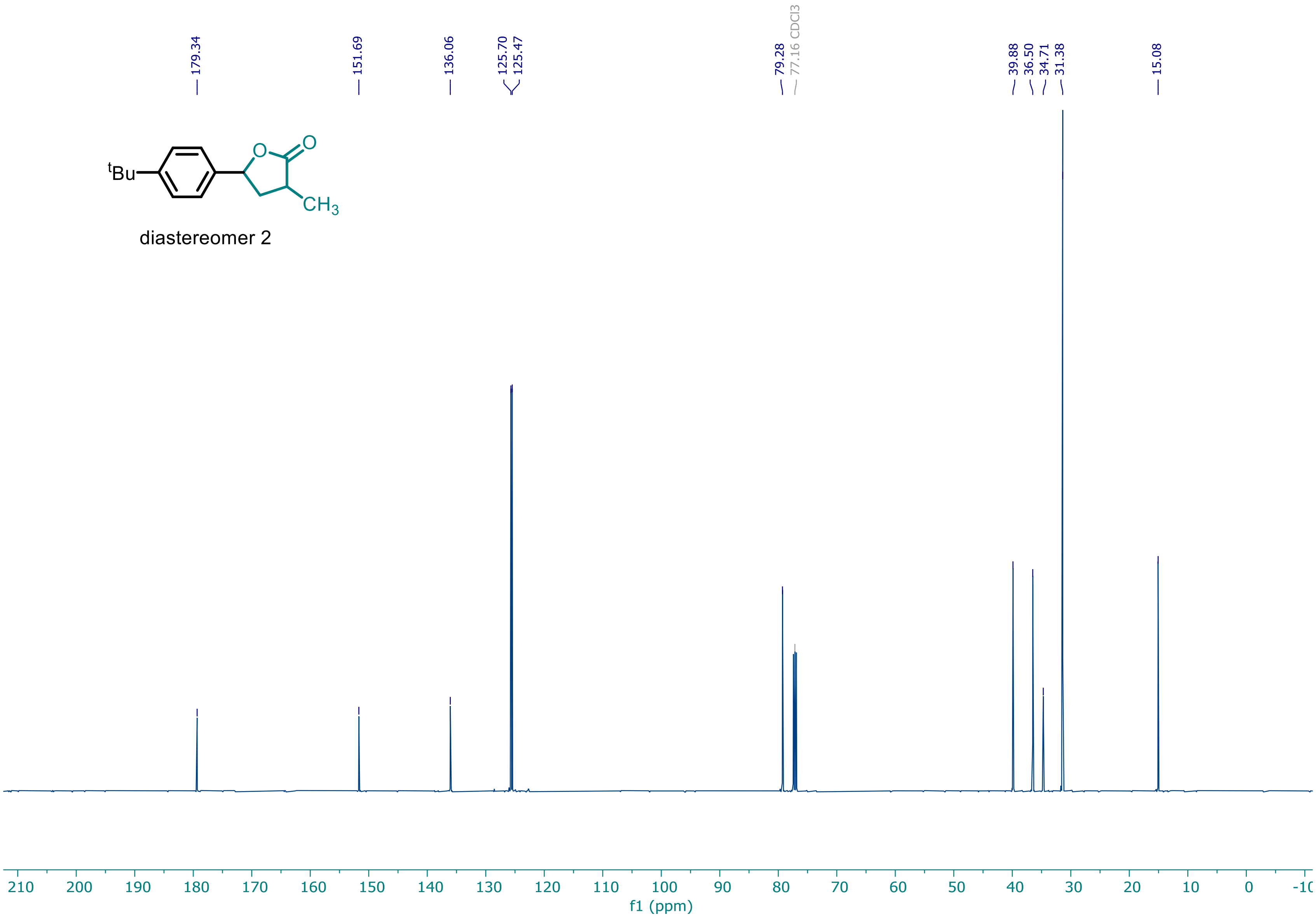

^{1}H NMR (300 MHz, $CDCl_3$) of **39a**

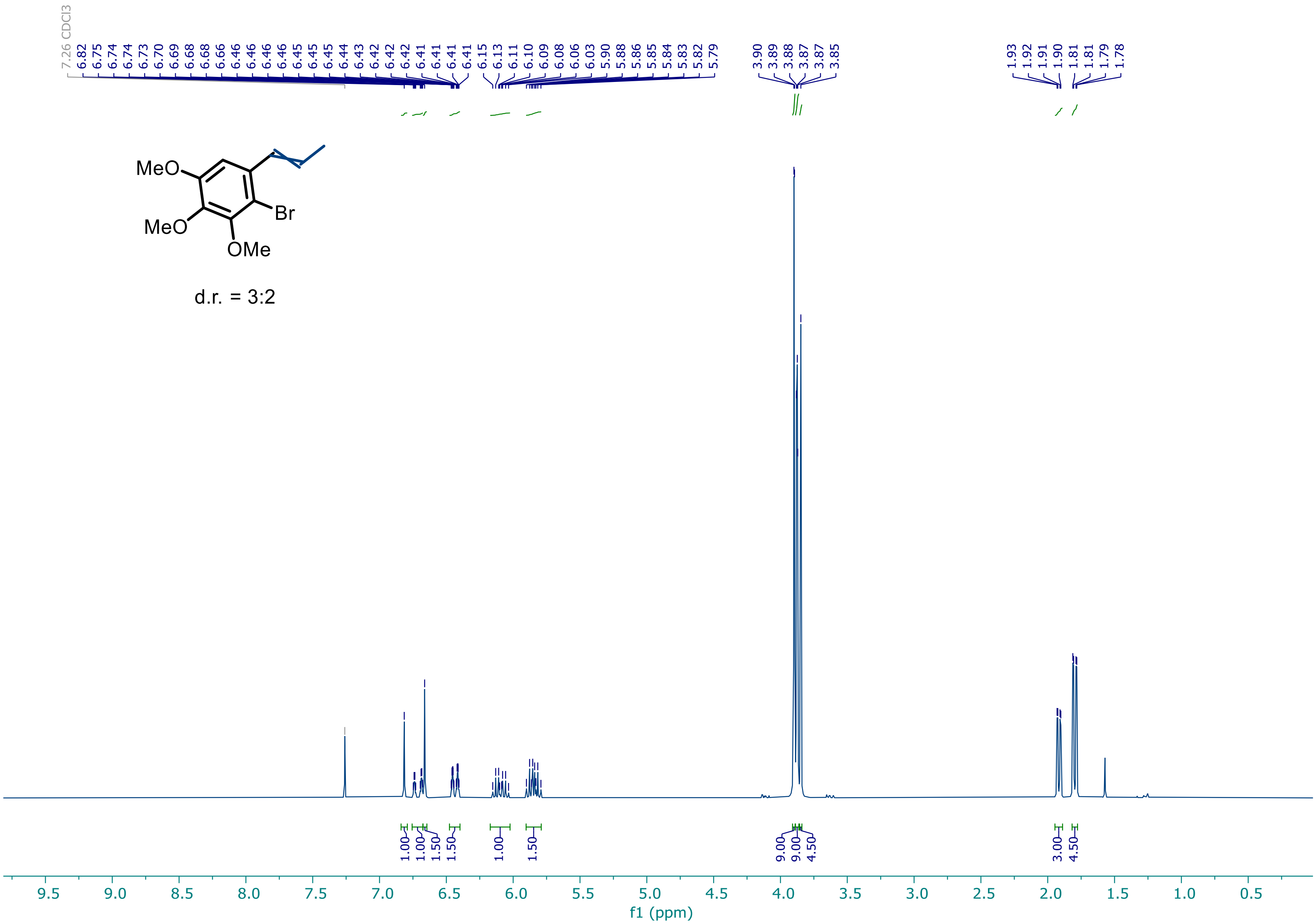

^{13}C NMR (75 MHz, $CDCl_3$) of **39a**

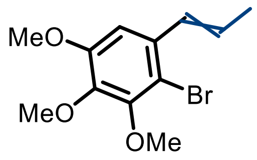

d.r. = 3:2

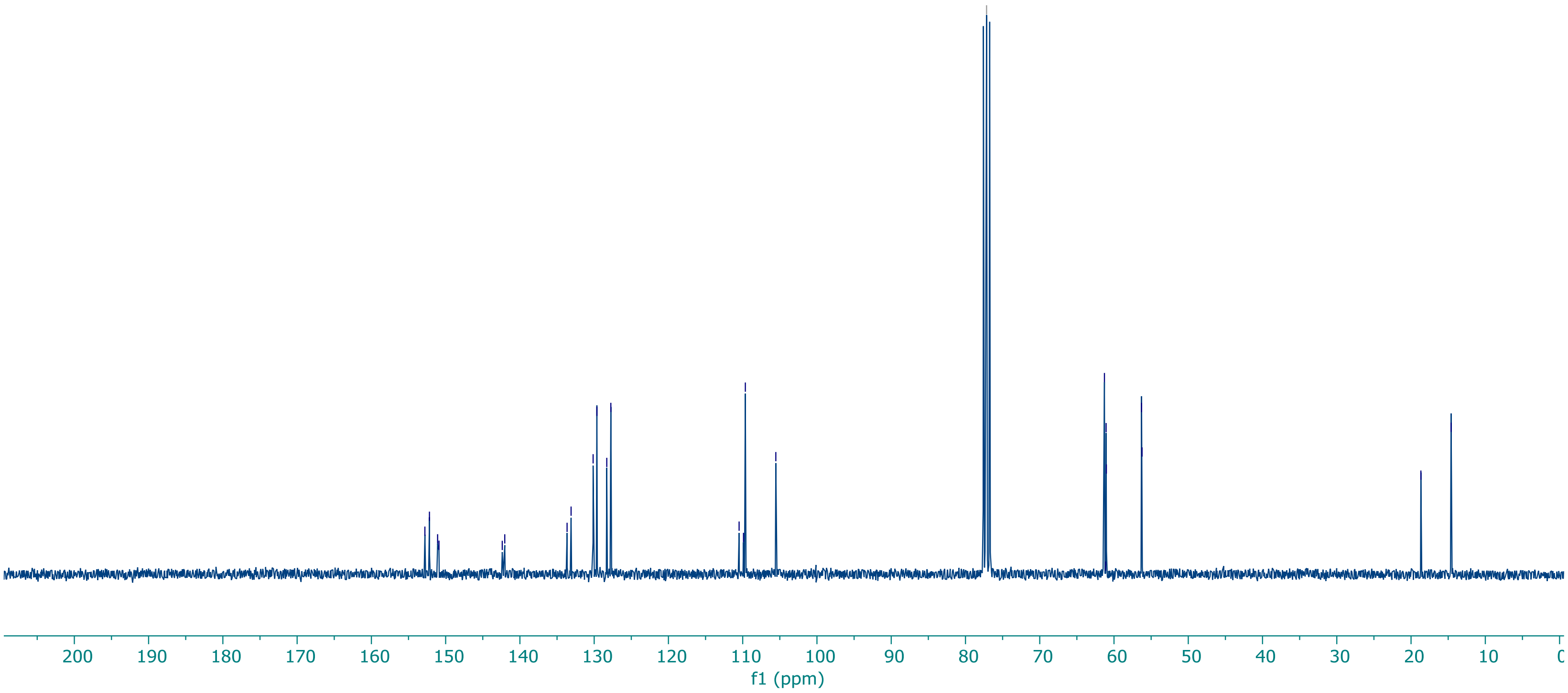

^{1}H NMR (300 MHz, CDCl$_3$) of **39**

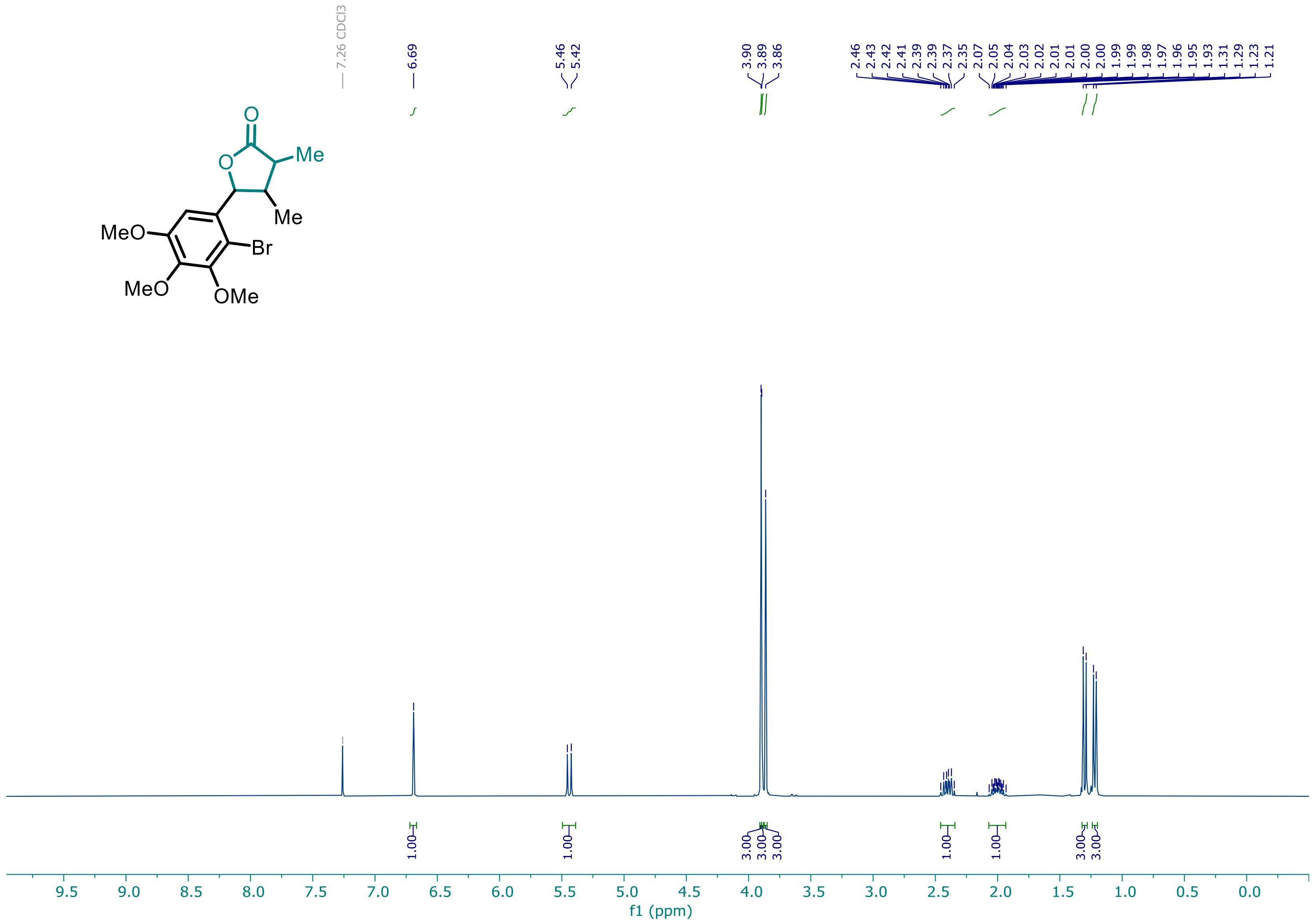

^{13}C NMR (126 MHz, $CDCl_3$) of **39**

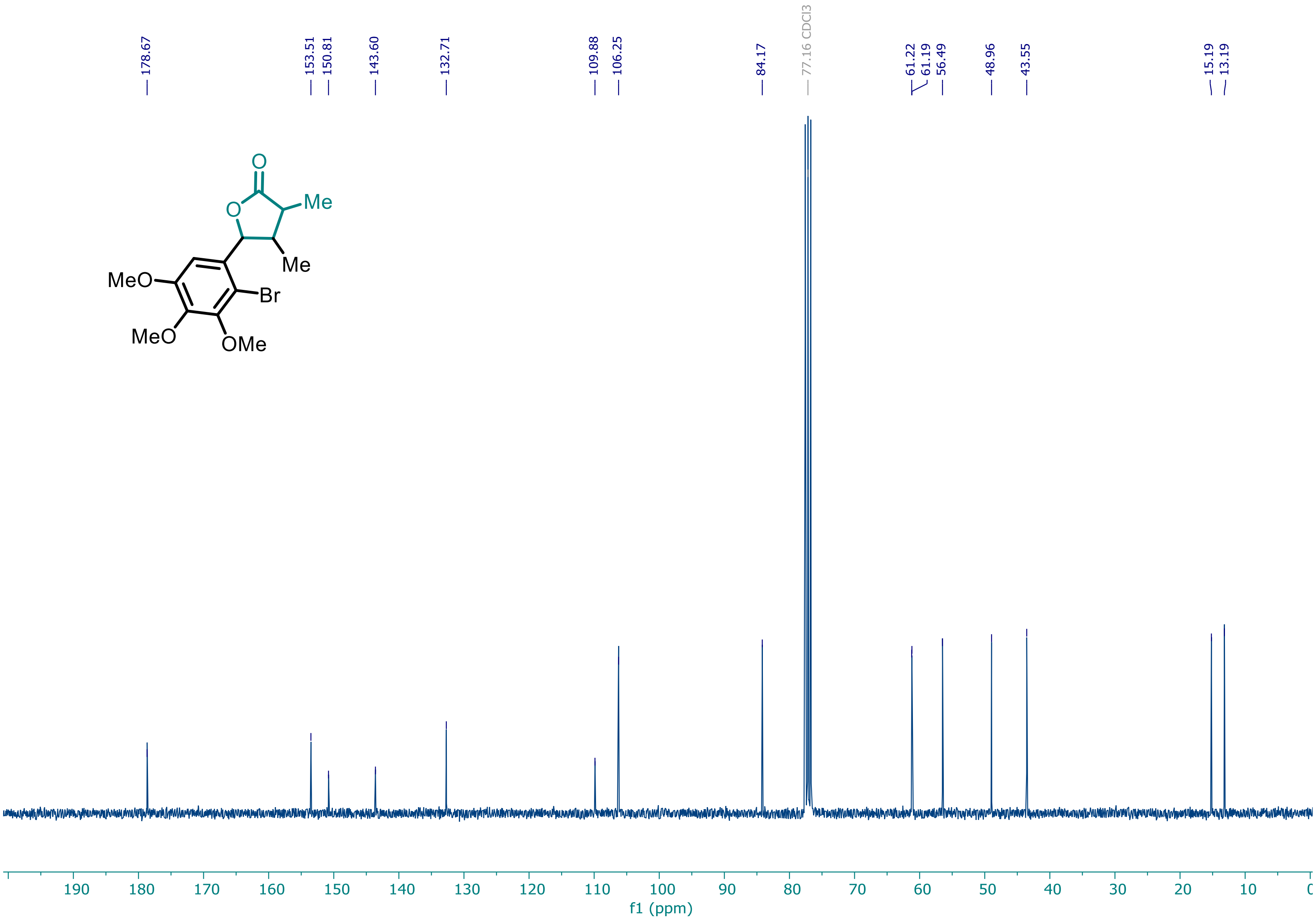

^{1}H NMR (500 MHz, $CDCl_3$) of **40**

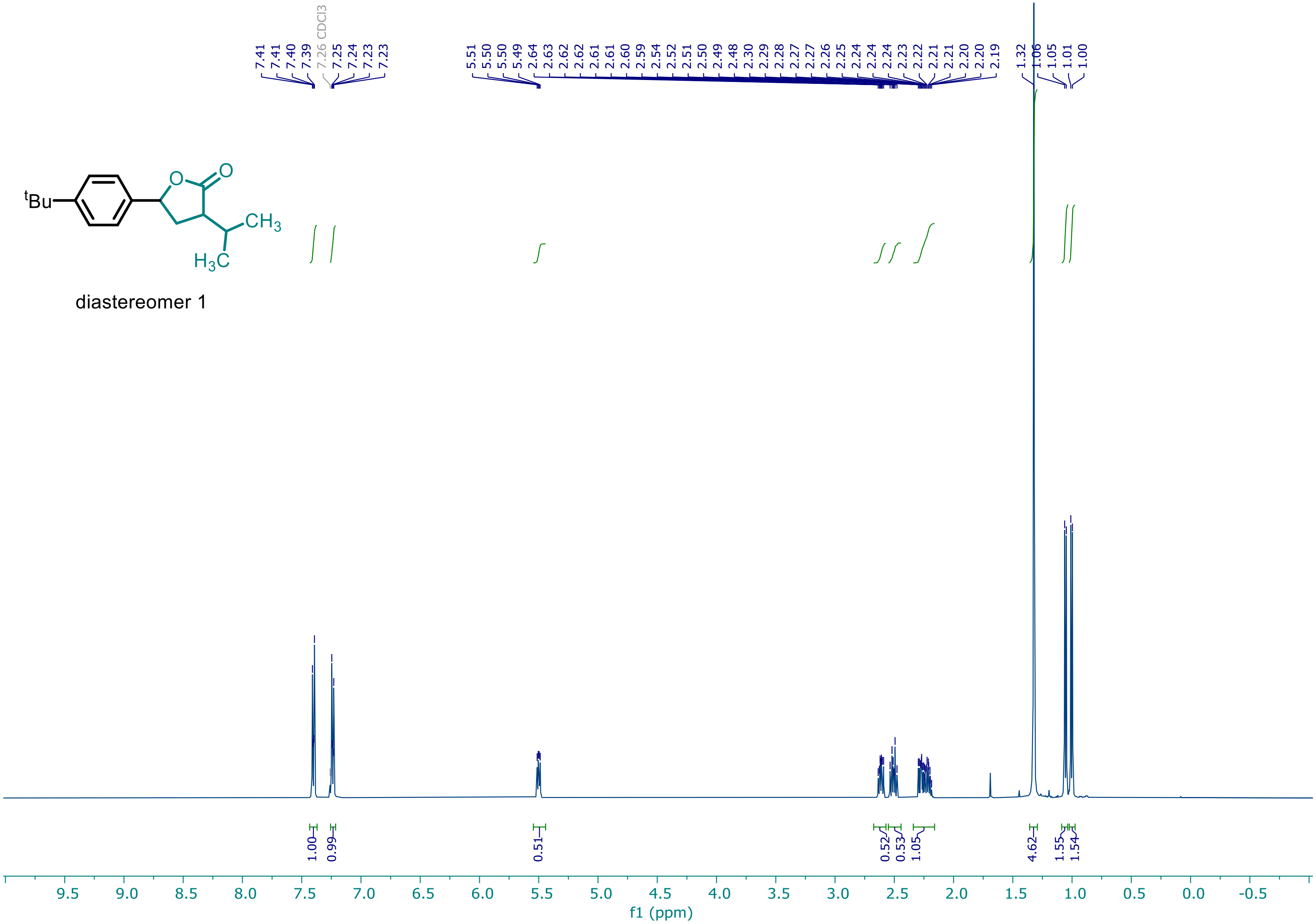

^{13}C NMR (126 MHz, $CDCl_3$) of **40**

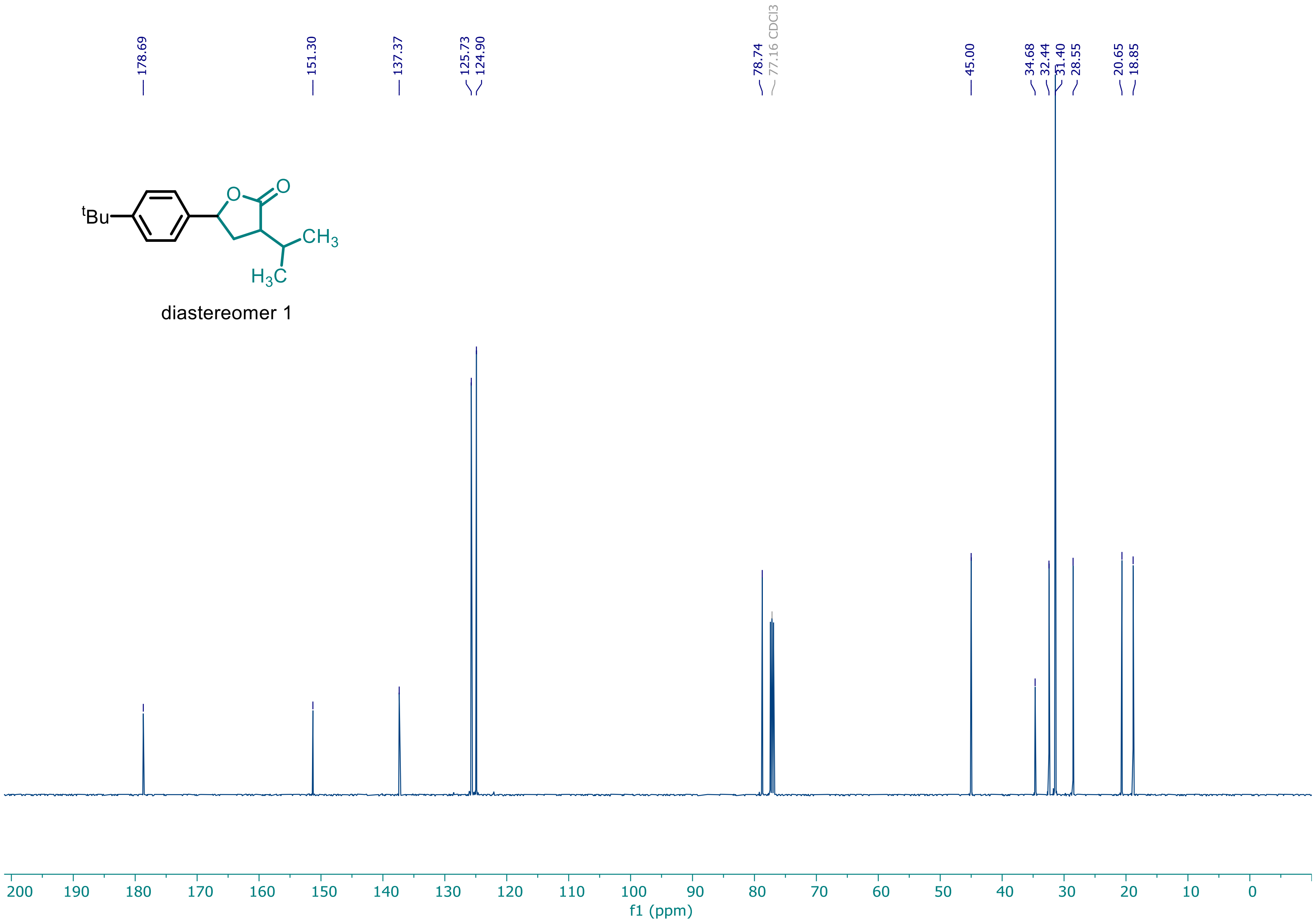

^{1}H NMR (500 MHz, $CDCl_3$) of **40**

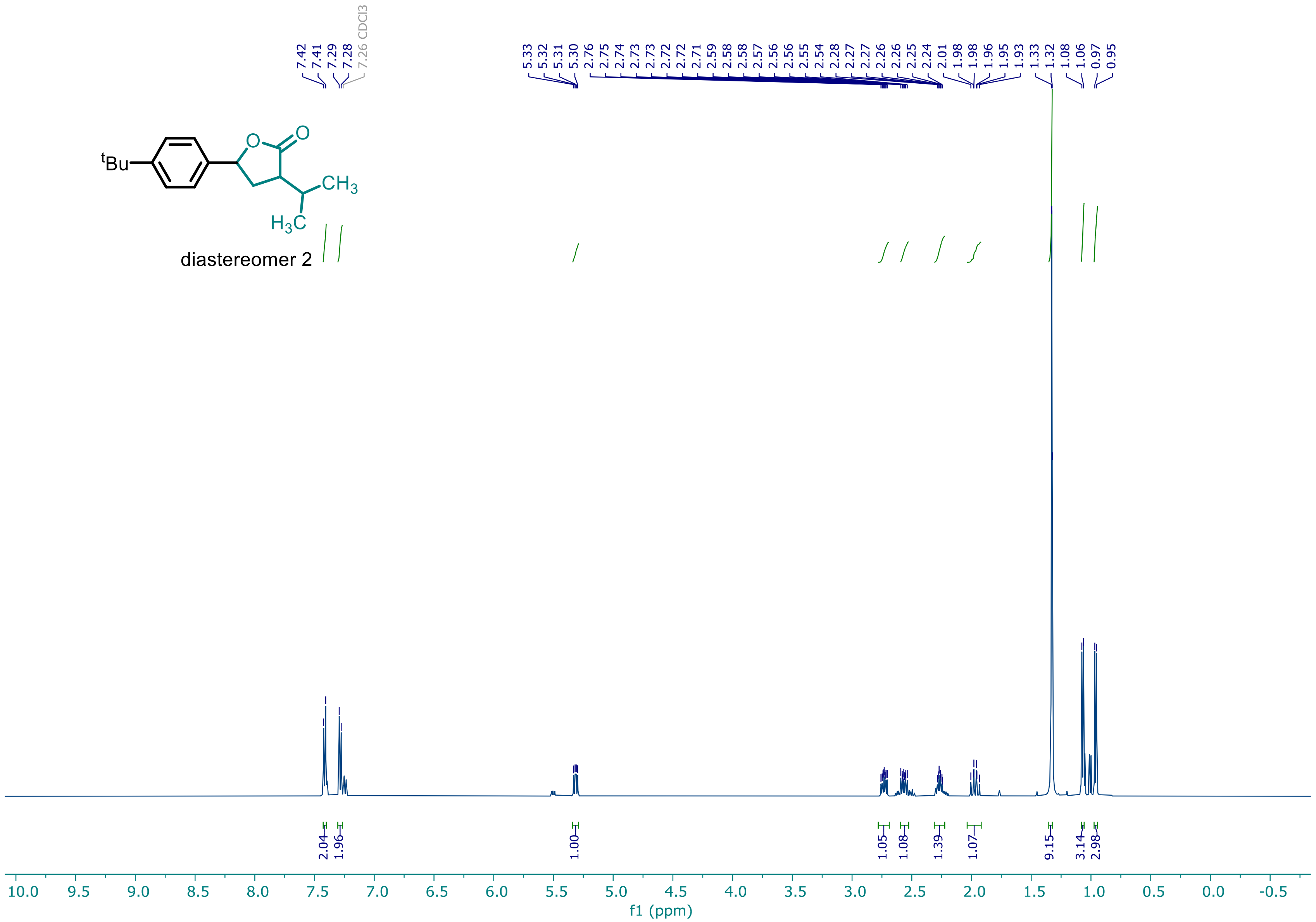

^{13}C NMR (126 MHz, $CDCl_3$) of **40**

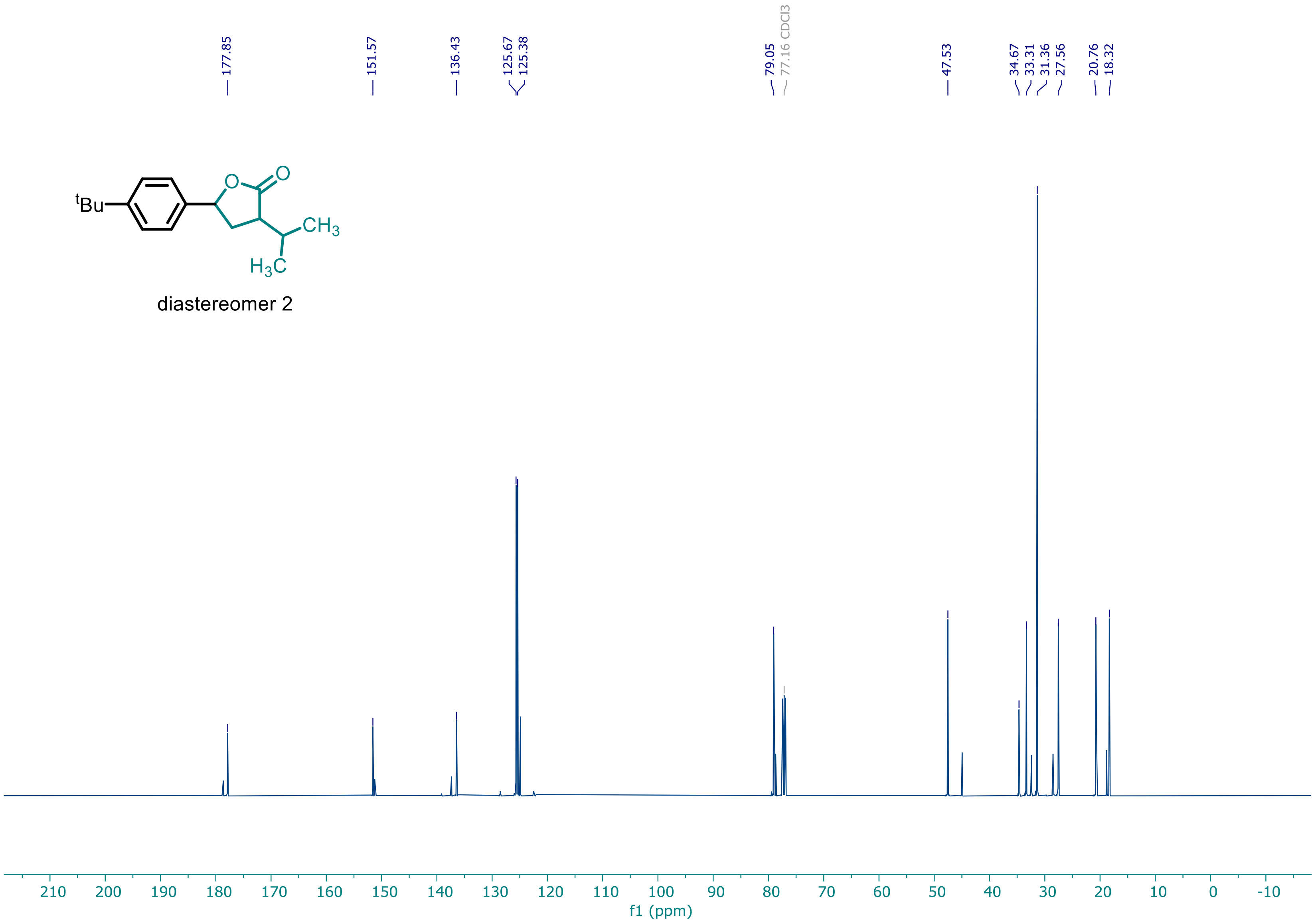

^{1}H NMR (300 MHz, $CDCl_3$) of **41**

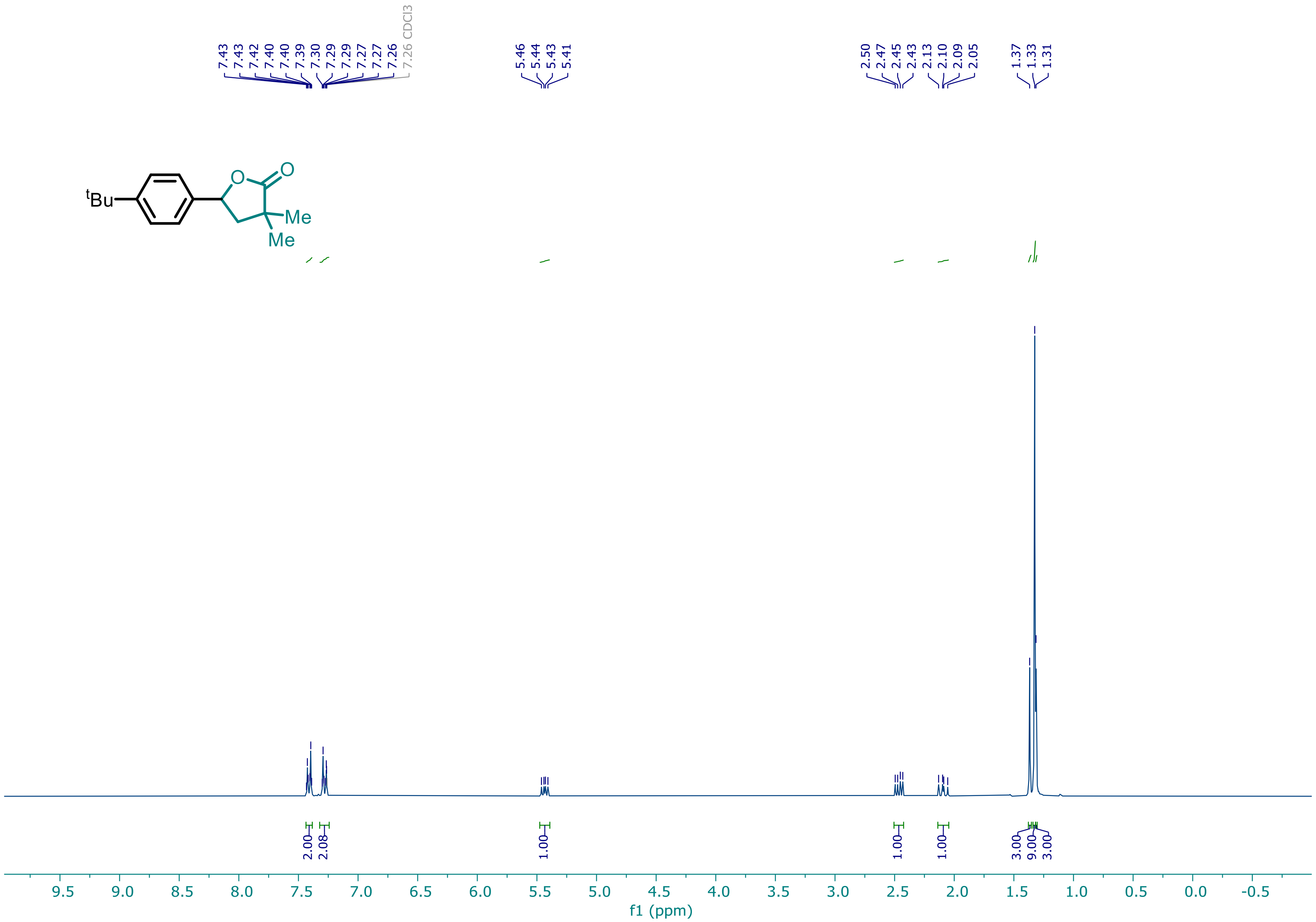

^{13}C NMR (75 MHz, $CDCl_3$) of **41**

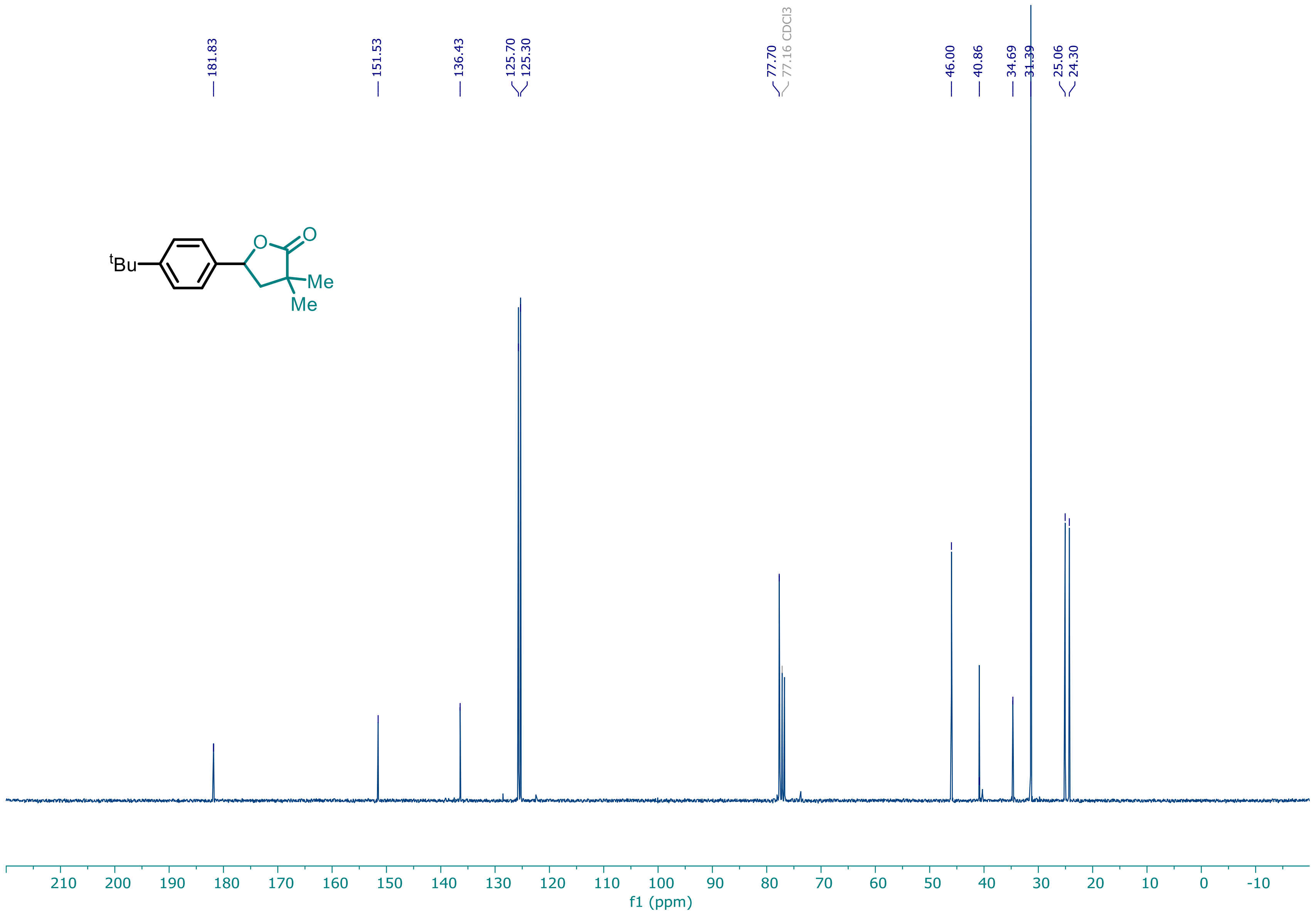

^{1}H NMR (300 MHz, $CDCl_3$) of **42**

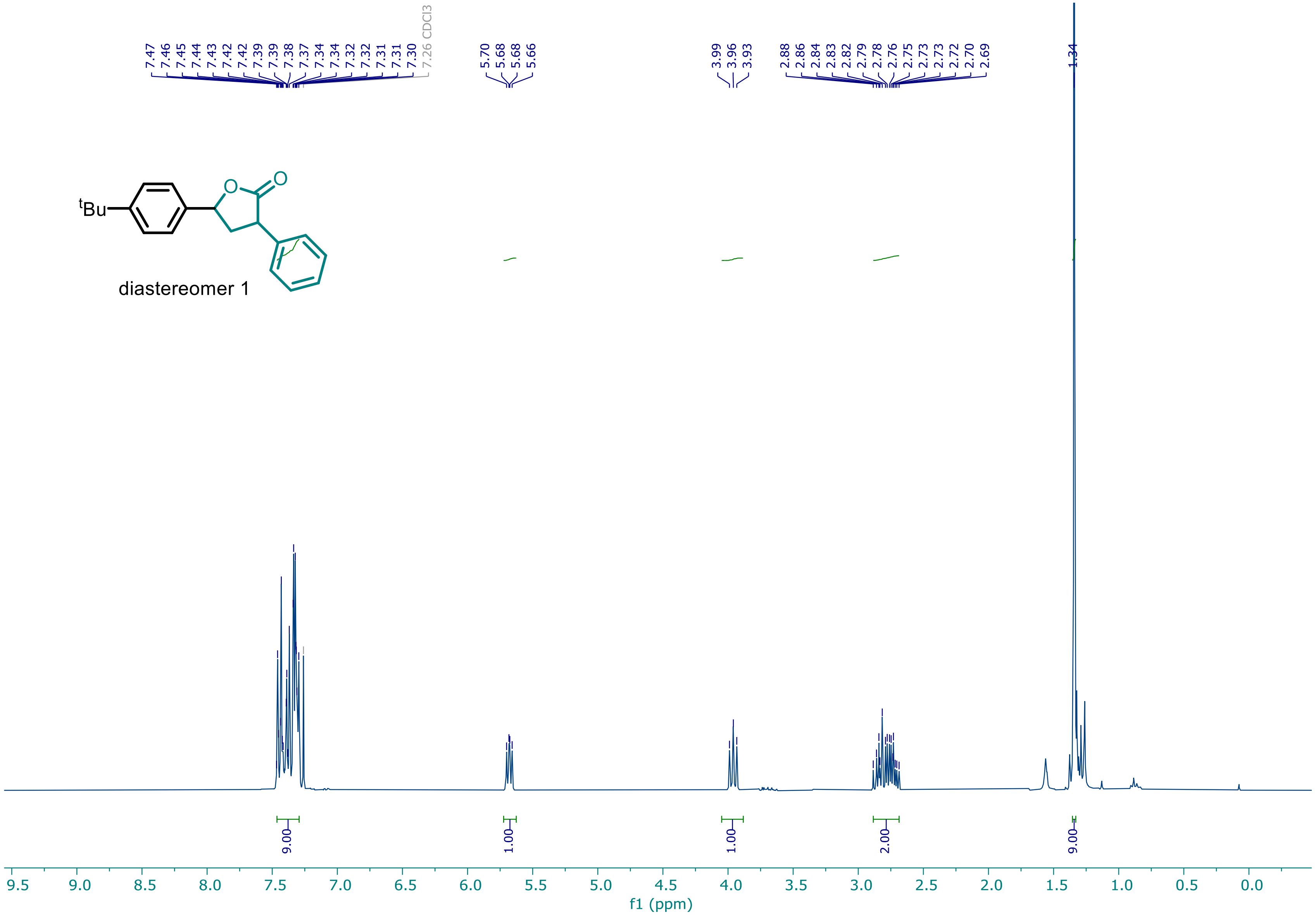

^{13}C NMR (75 MHz, CDCl$_3$) of **42**

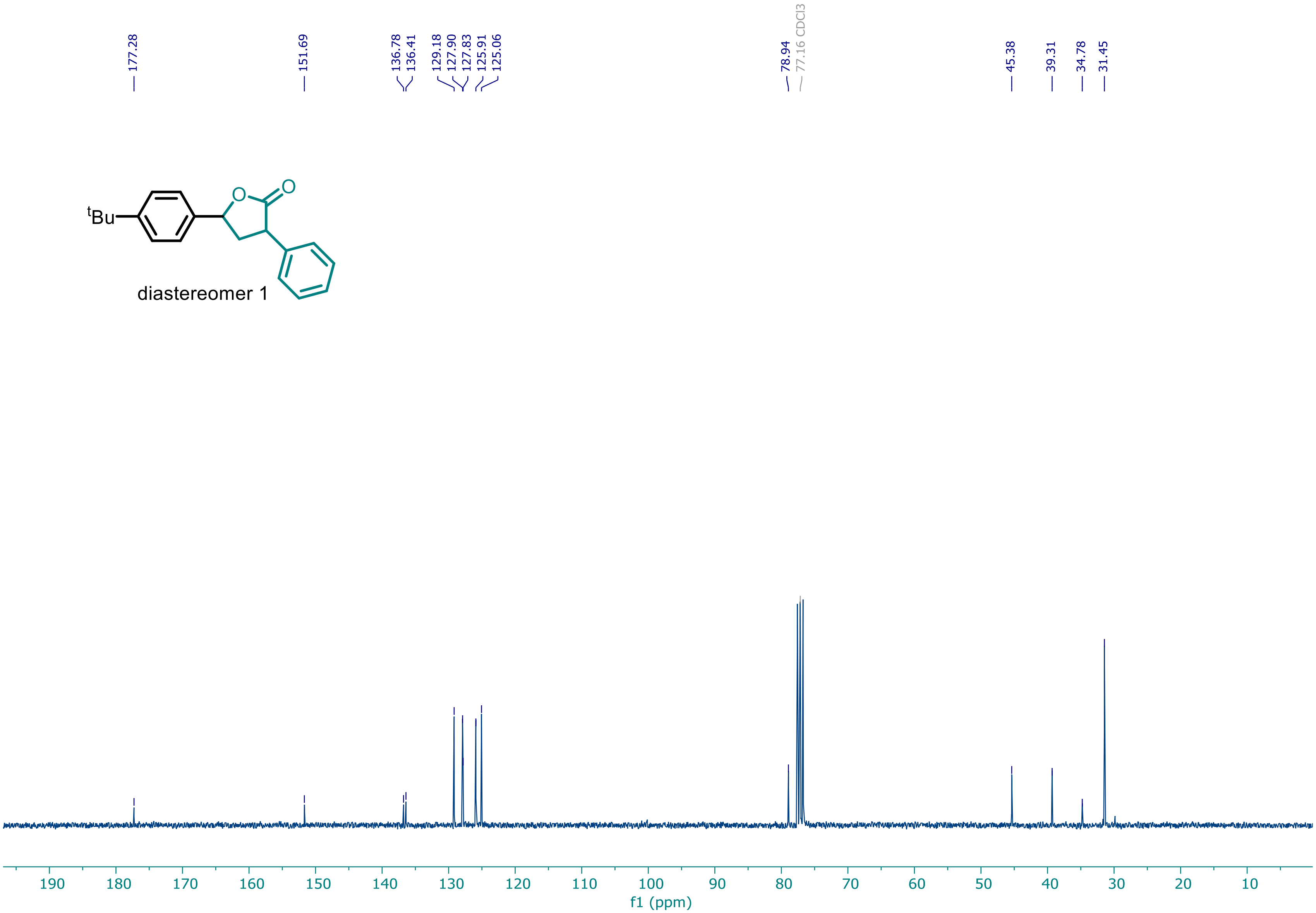

^{1}H NMR (300 MHz, $CDCl_3$) of **42**

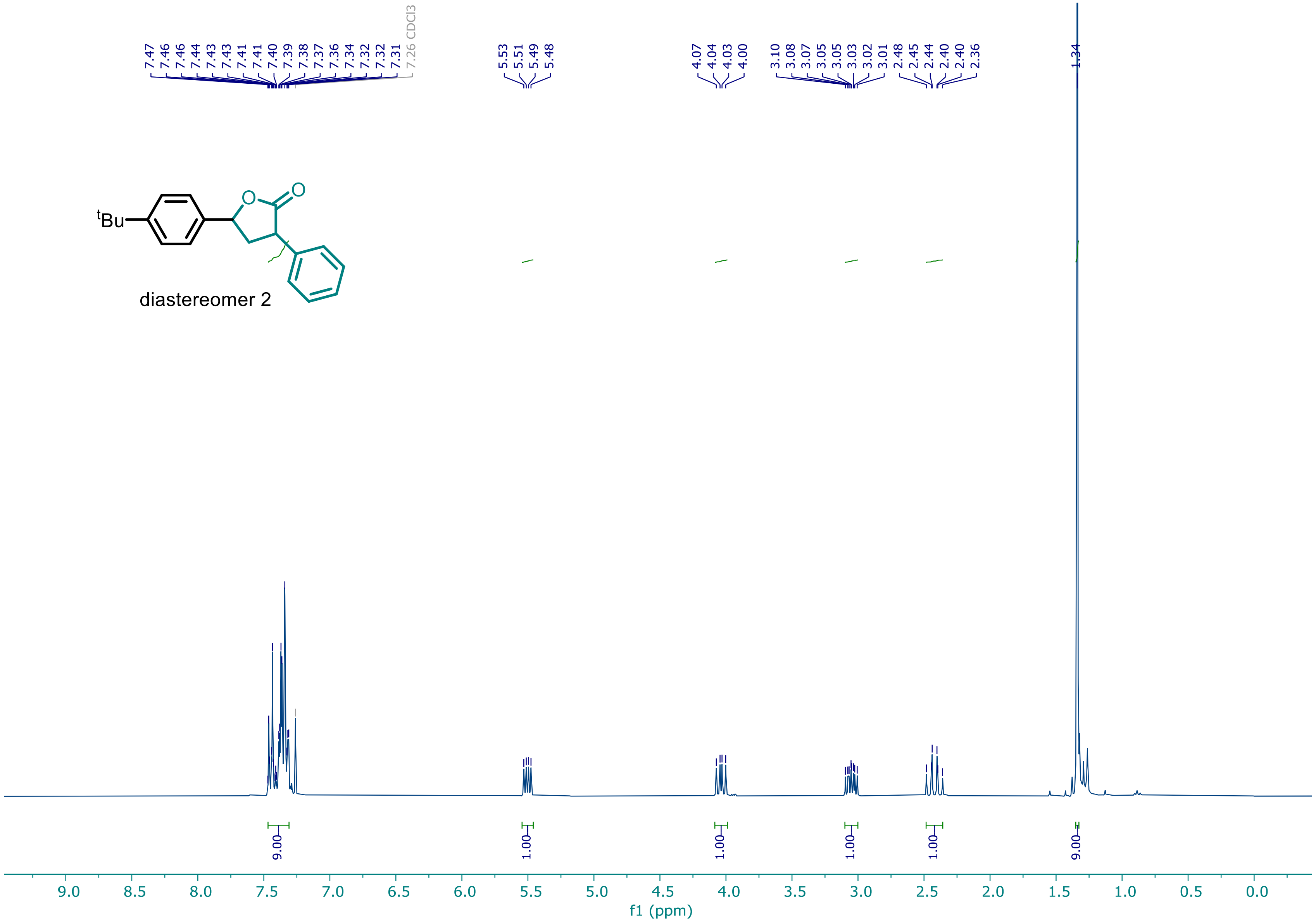

^{13}C NMR (75 MHz, CDCl$_3$) of **42**

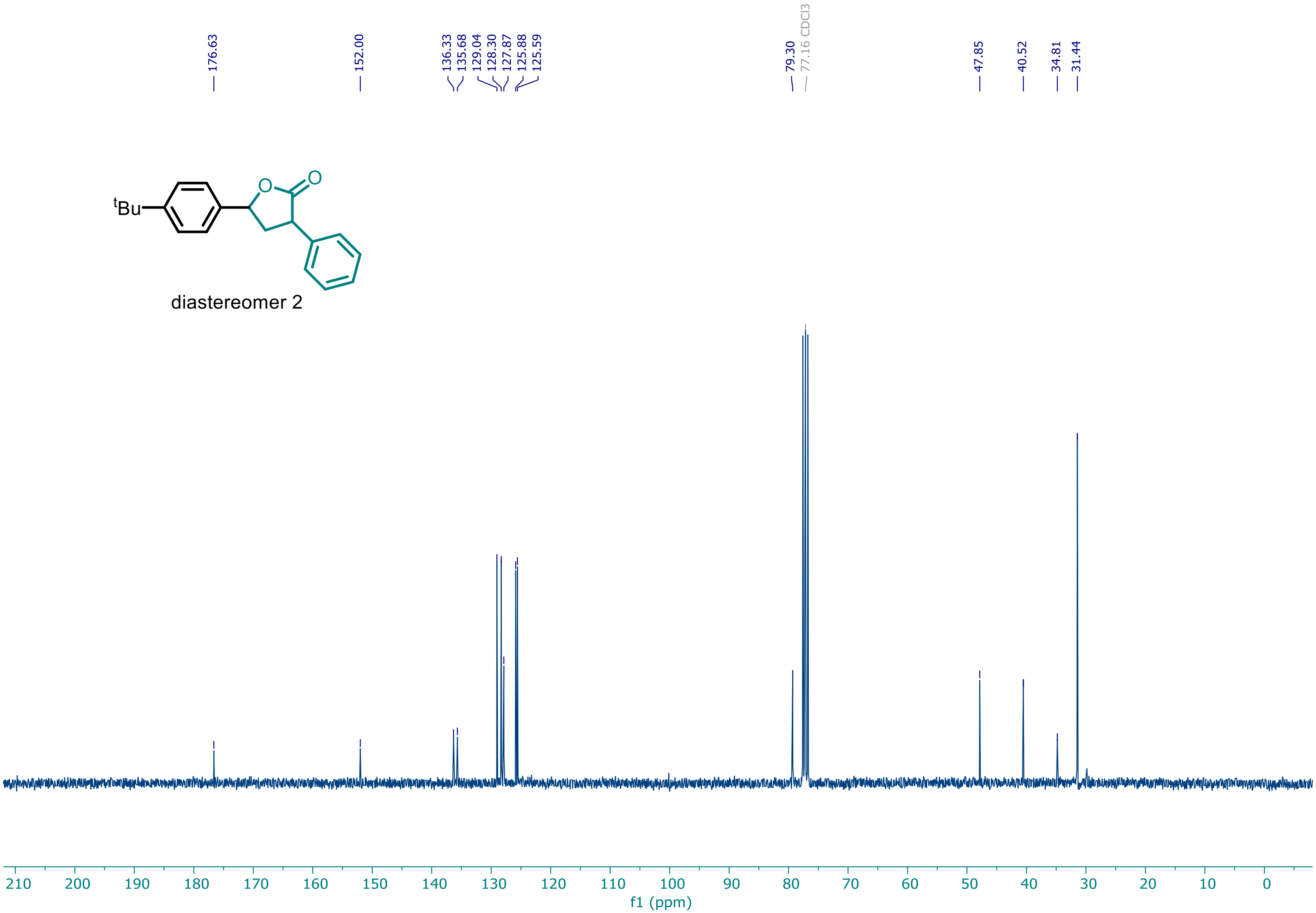

^{1}H NMR (300 MHz, $CDCl_3$) of **43**

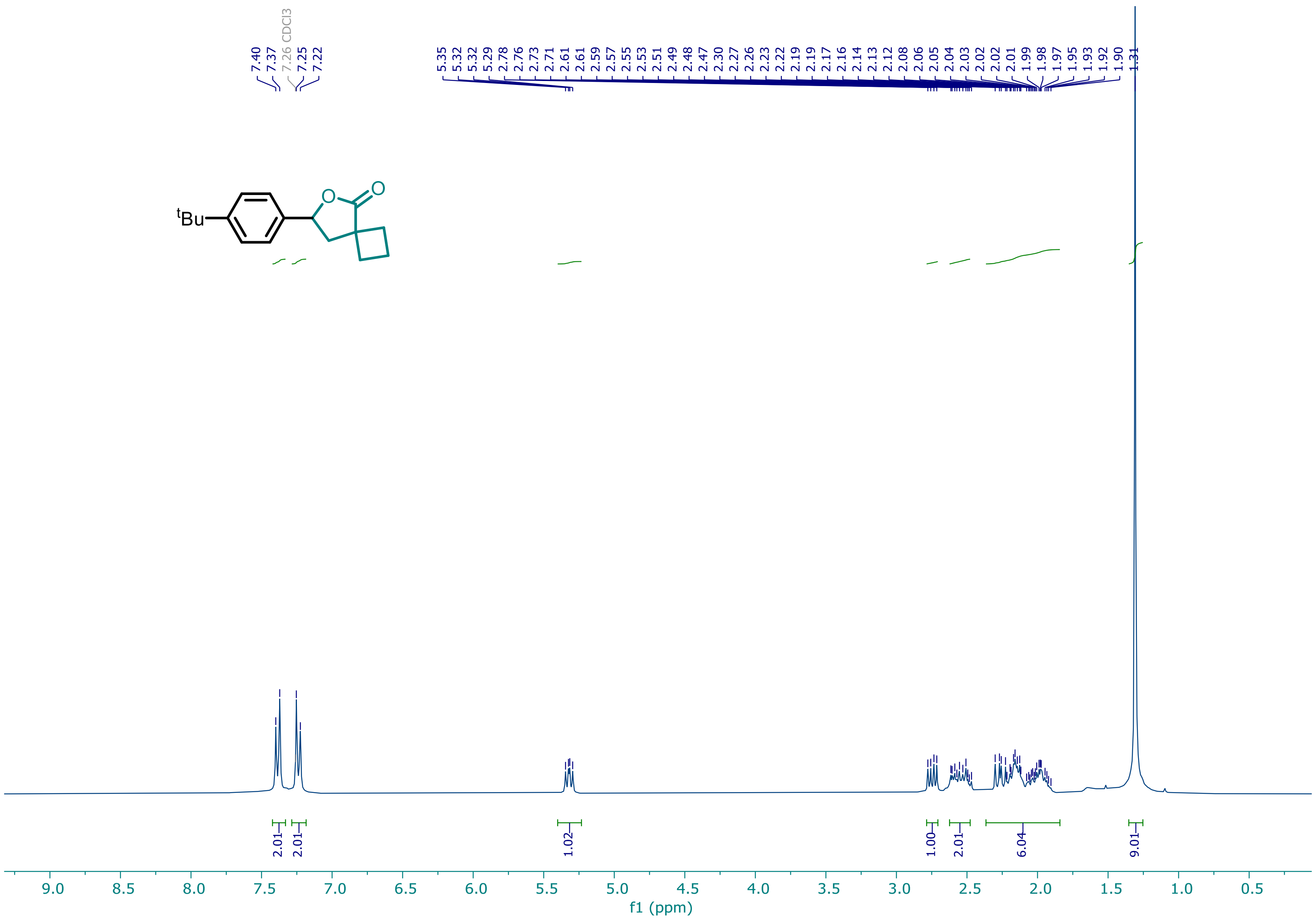

^{13}C NMR (75 MHz, $CDCl_3$) of **43**

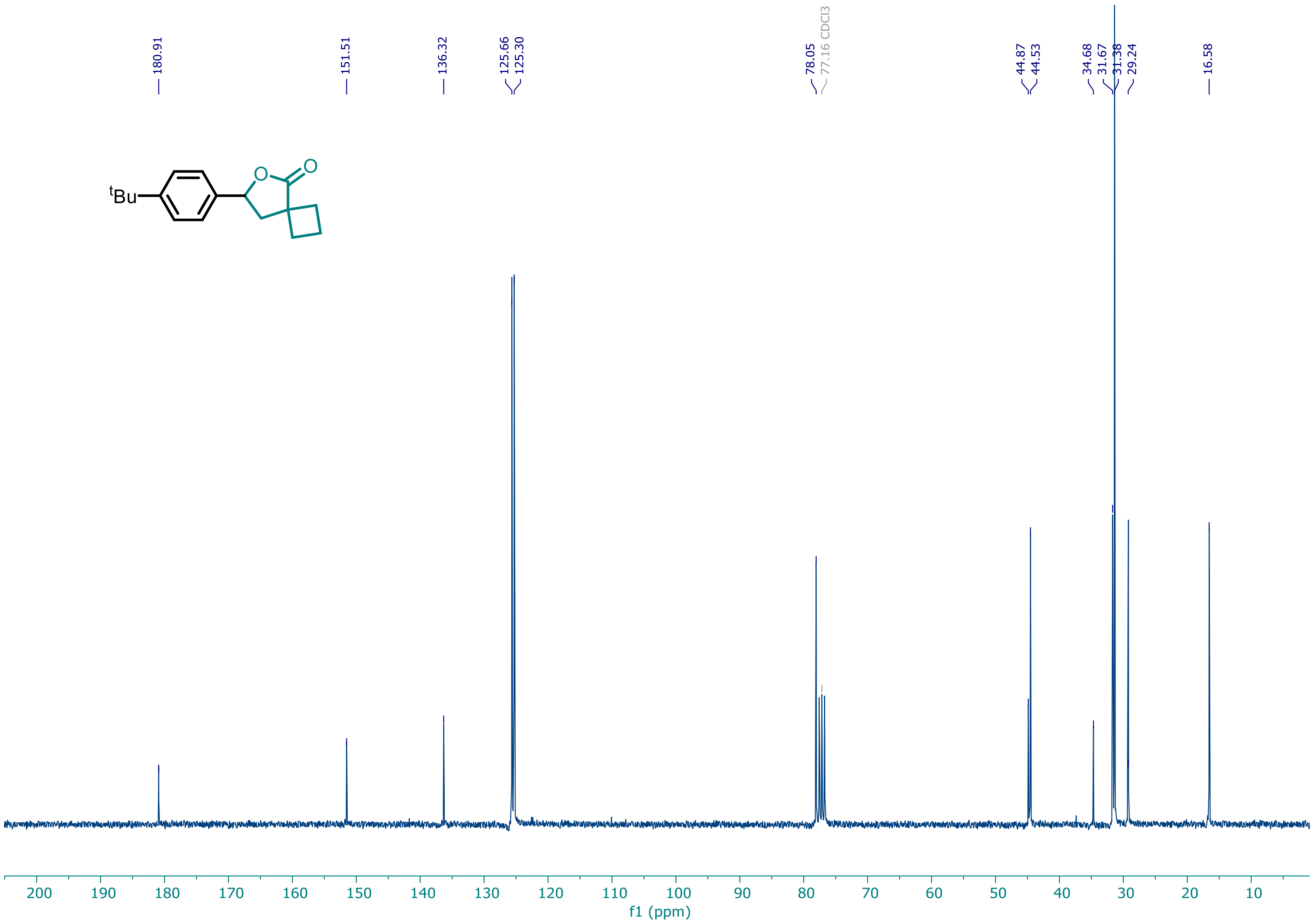

^{1}H NMR (300 MHz, $CDCl_3$) of **44**

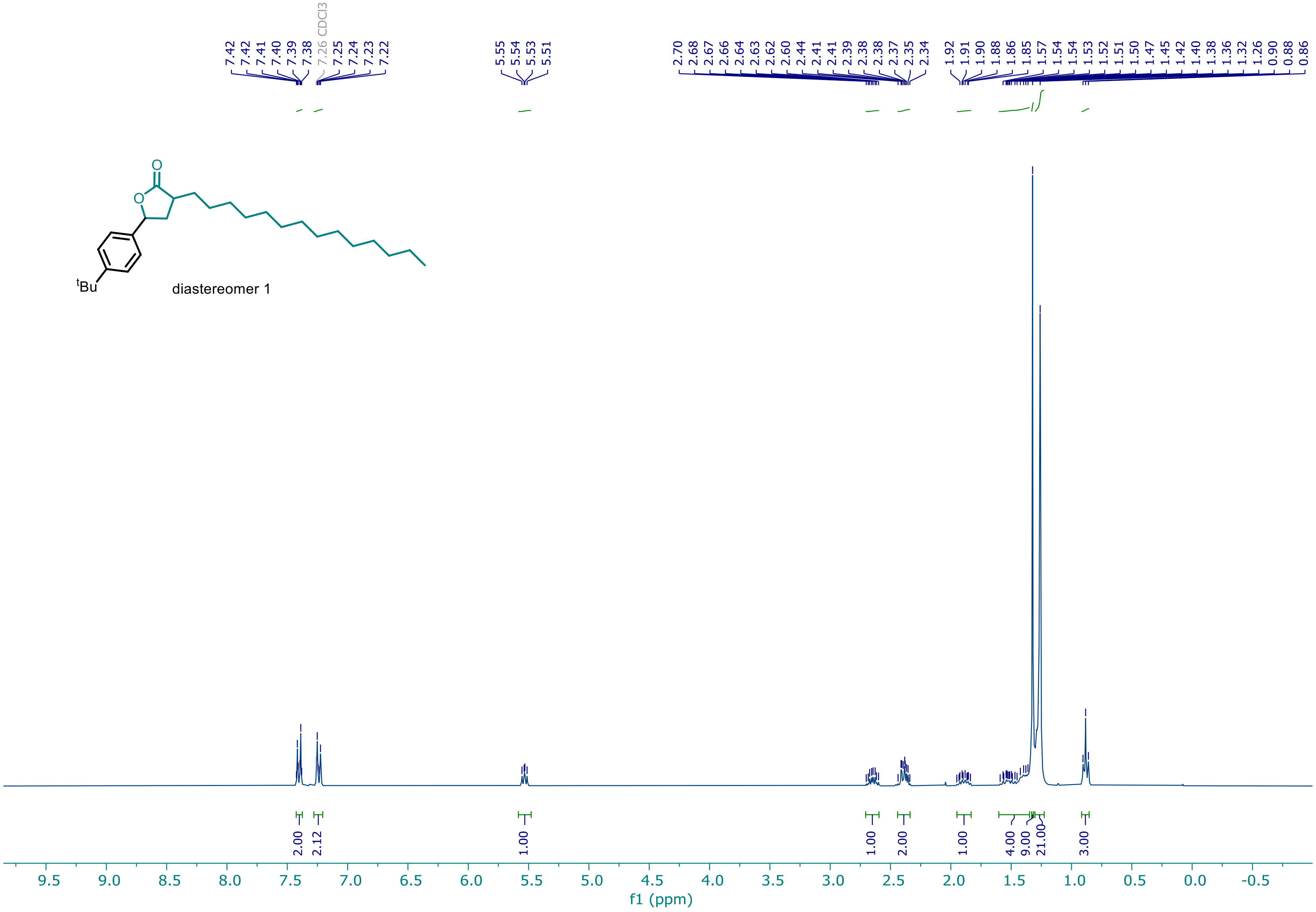

^{13}C NMR (75 MHz, $CDCl_3$) of **44**

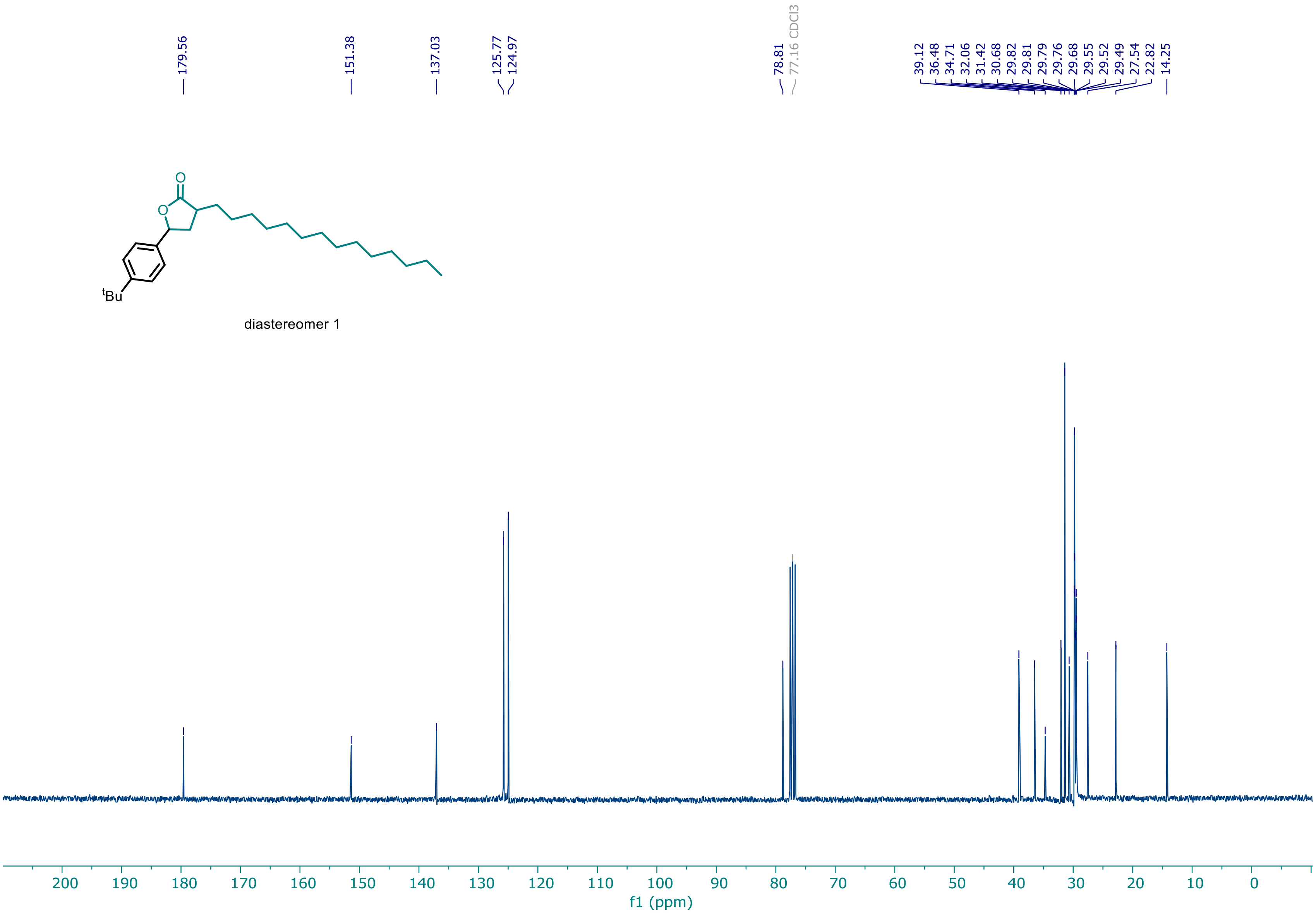

^{1}H NMR (300 MHz, $CDCl_3$) of **44**

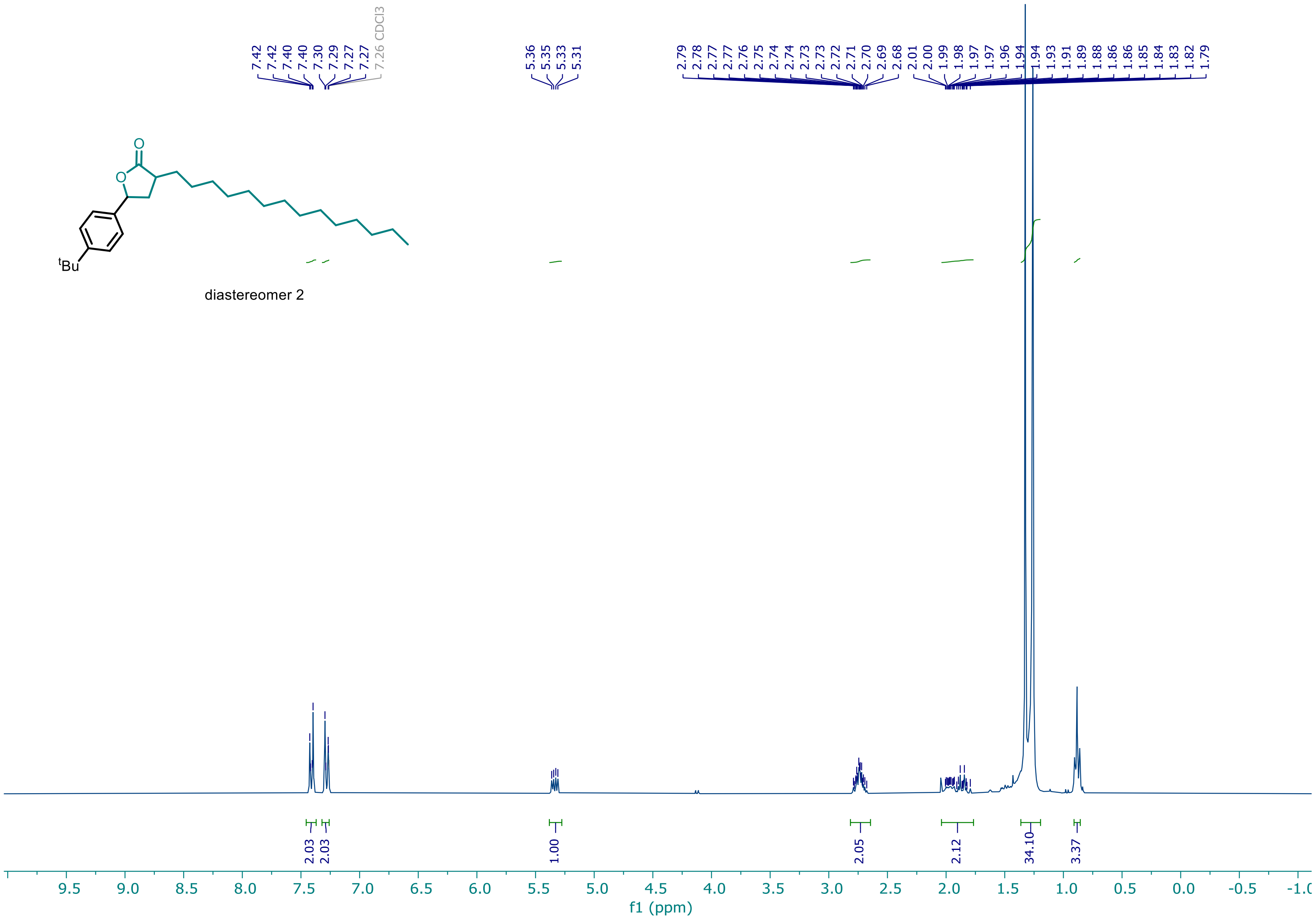

^{13}C NMR (75 MHz, $CDCl_3$) of **44**

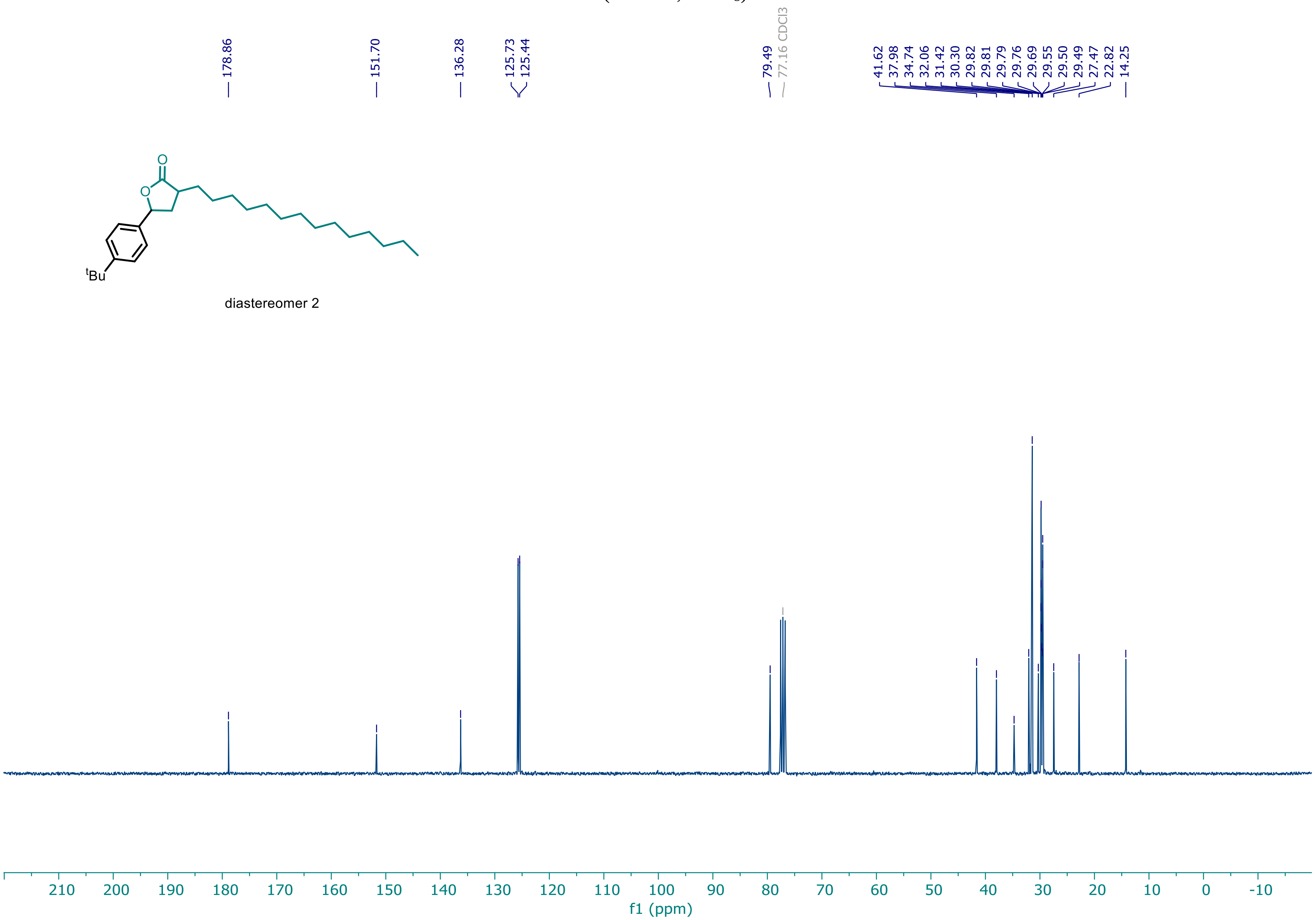

^{1}H NMR (300 MHz, $CDCl_3$) of **45**

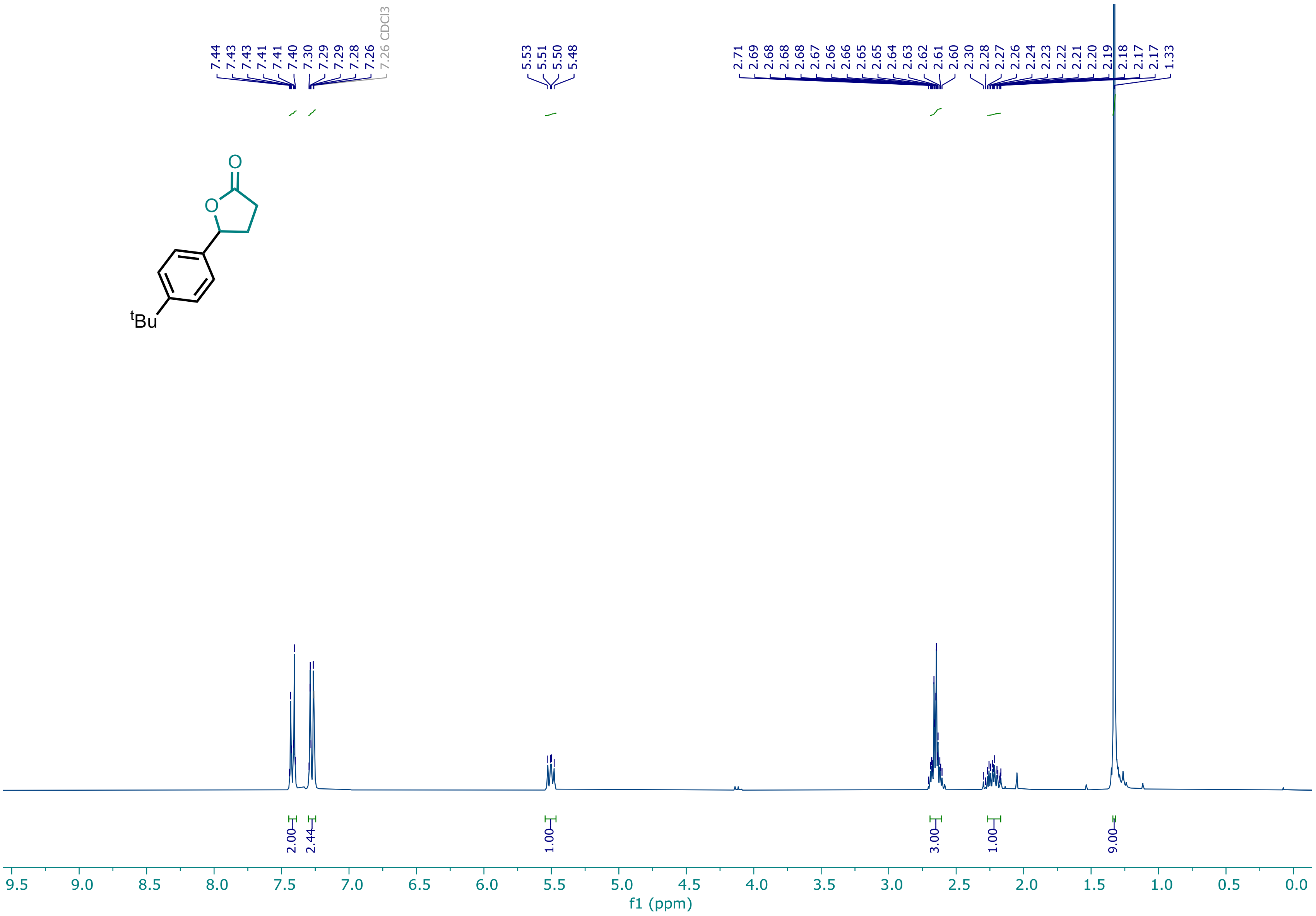

^{13}C NMR (75 MHz, $CDCl_3$) of **45**

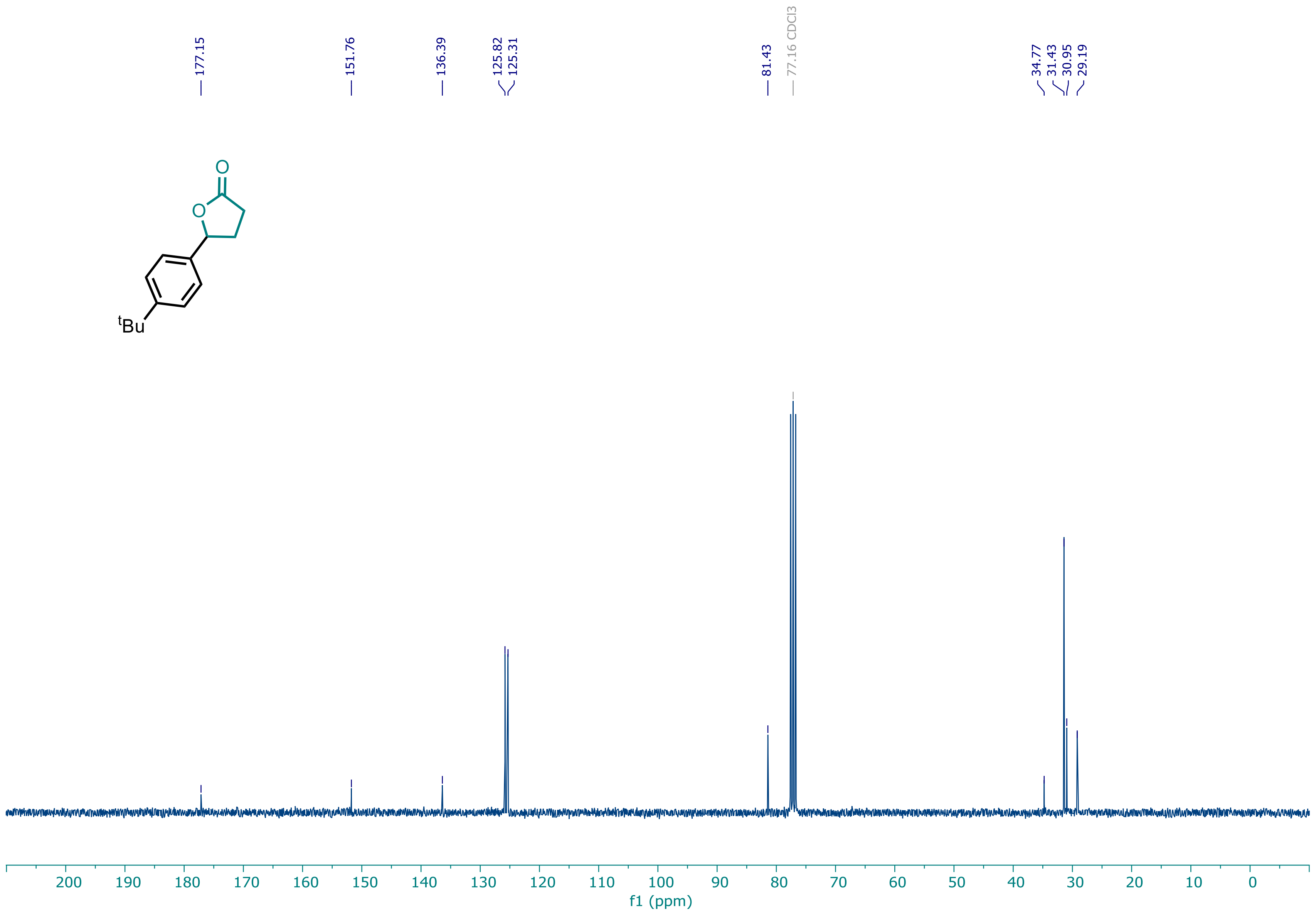

^{1}H NMR (400 MHz, $CDCl_3$) of **2**

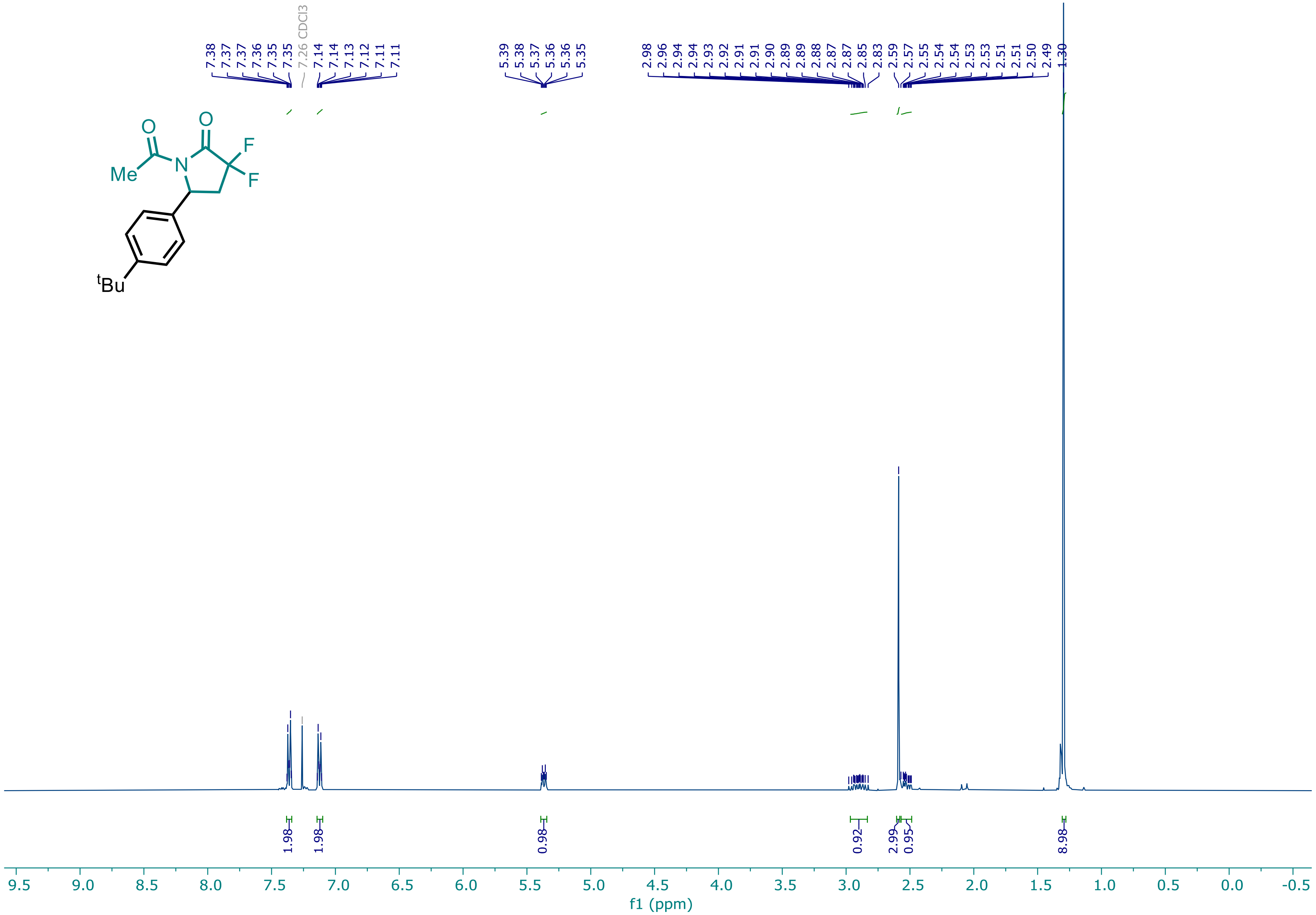

^{13}C NMR (101 MHz, $CDCl_3$) of **2**

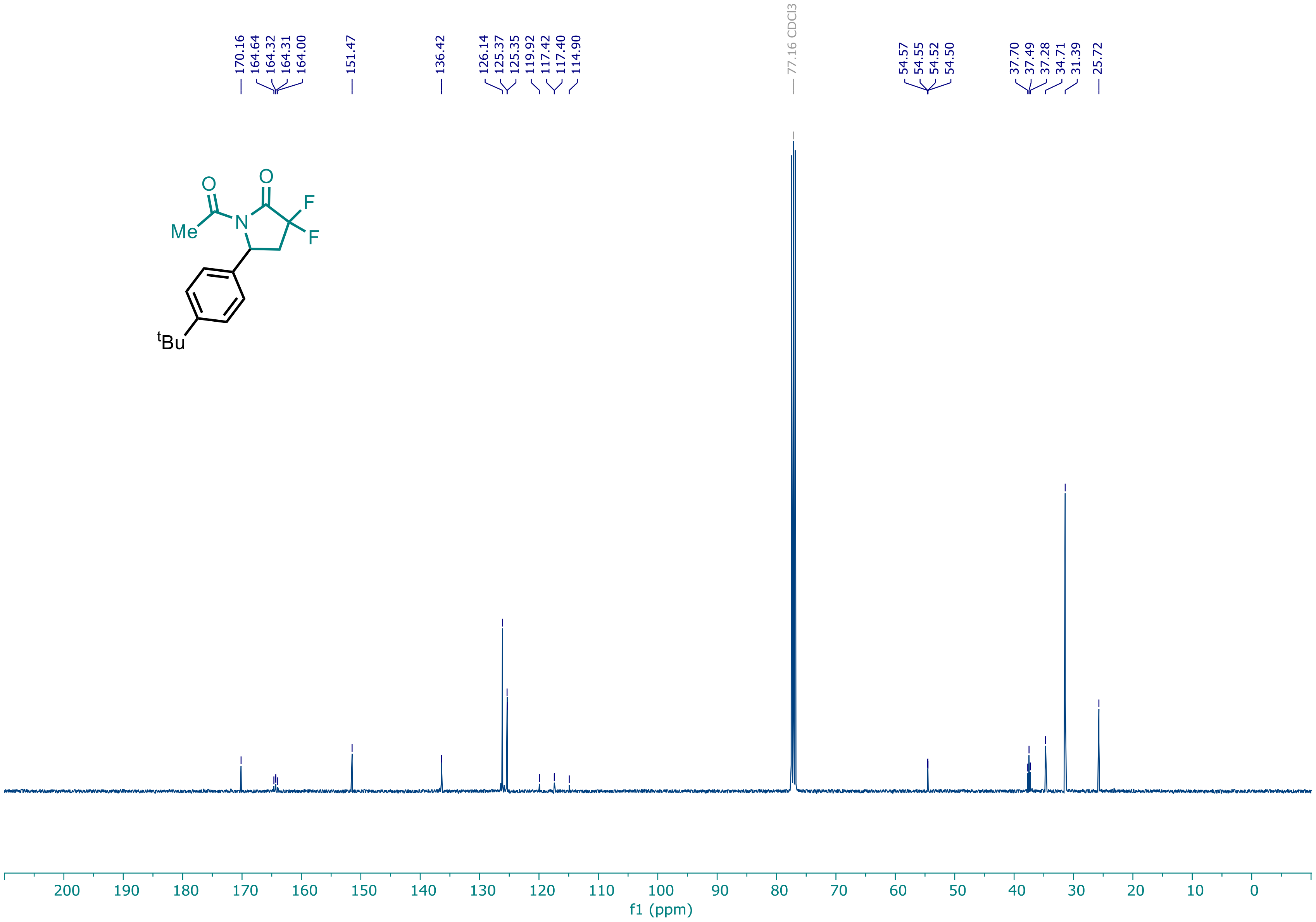

^{19}F NMR (376 MHz, CDCl$_3$) of **2**

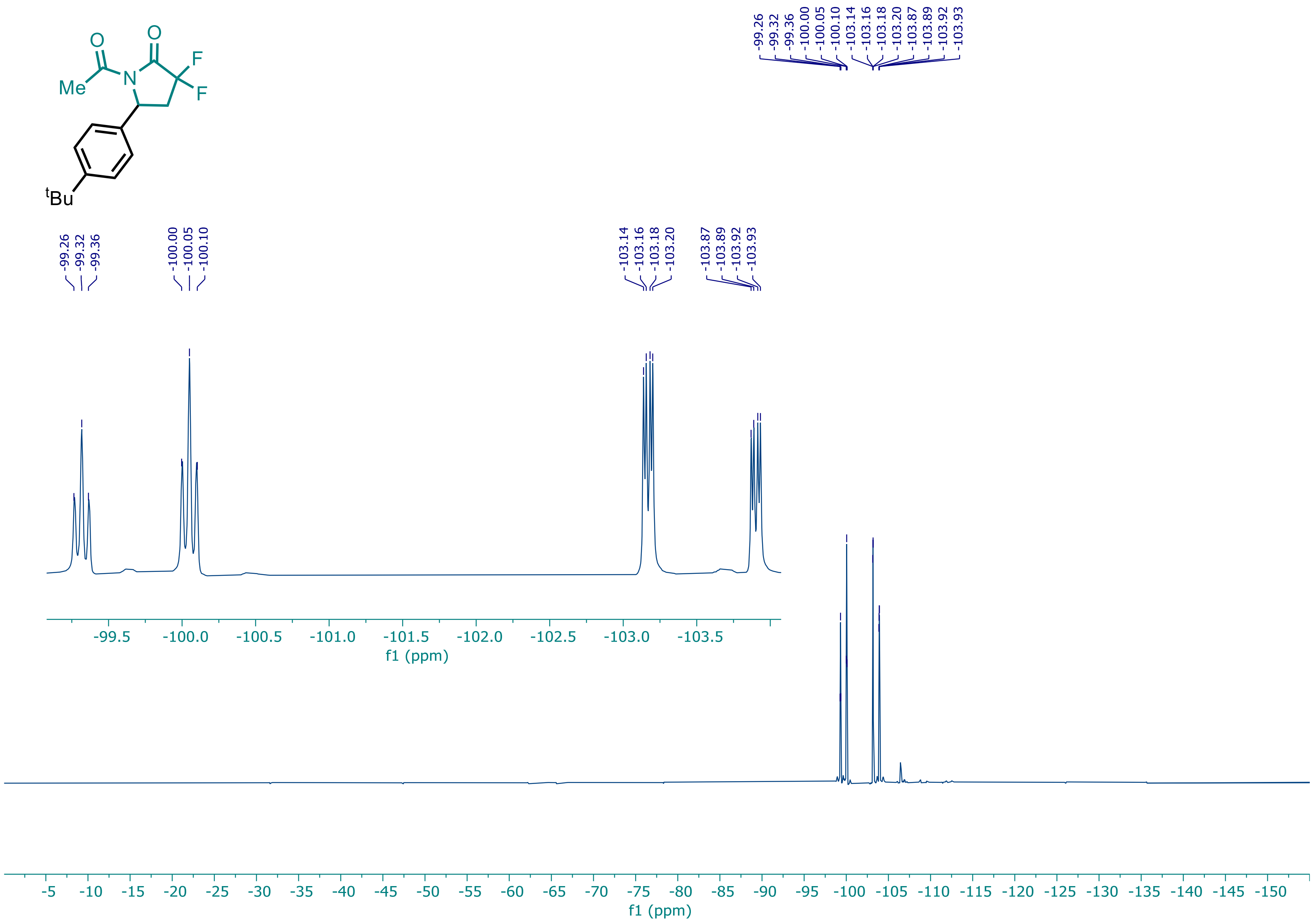

^{1}H NMR (400 MHz, $CDCl_3$) of **46**

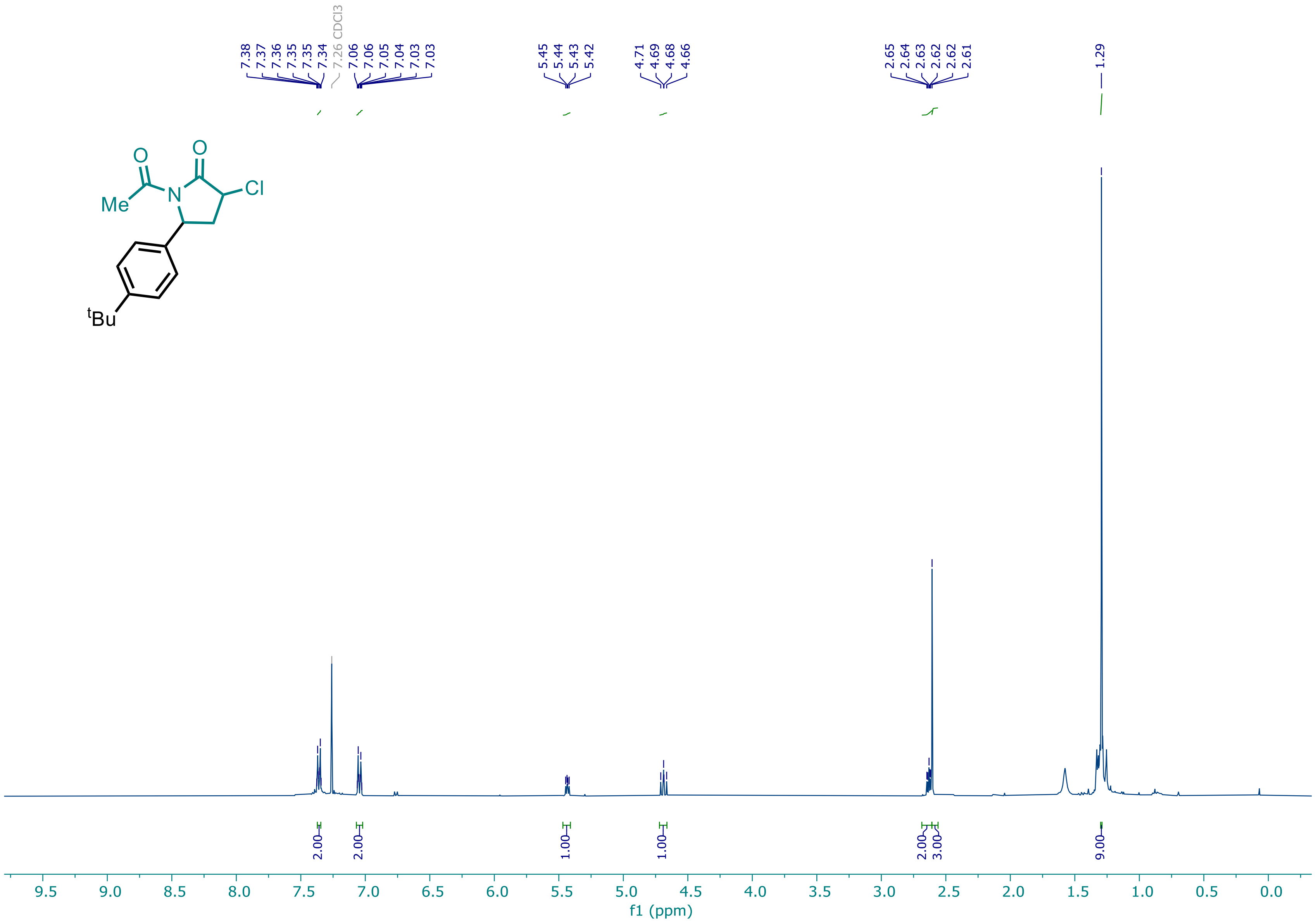

^{13}C NMR (101 MHz, $CDCl_3$) of **46**

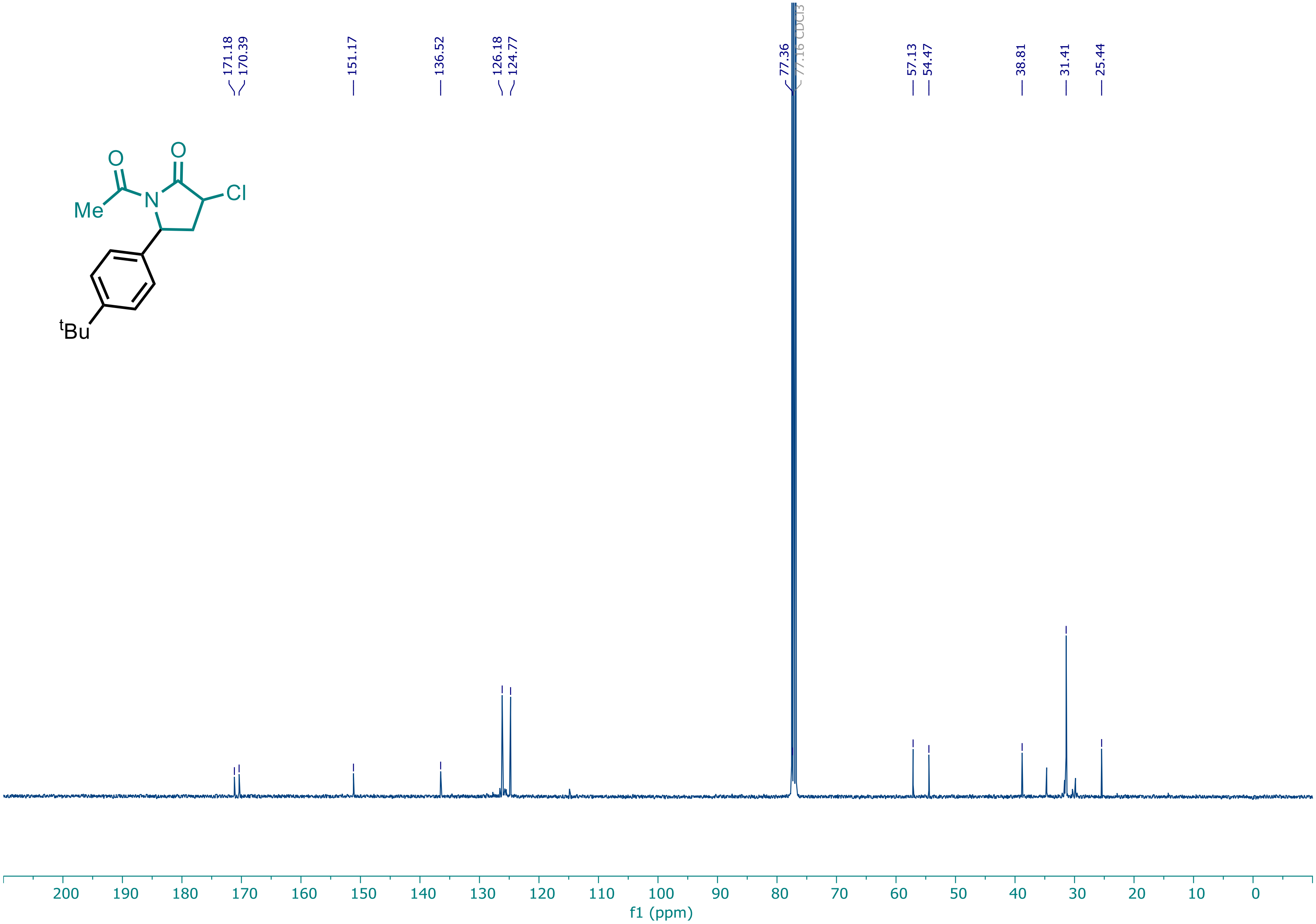

^{1}H NMR (300 MHz, $CDCl_3$) of **47**

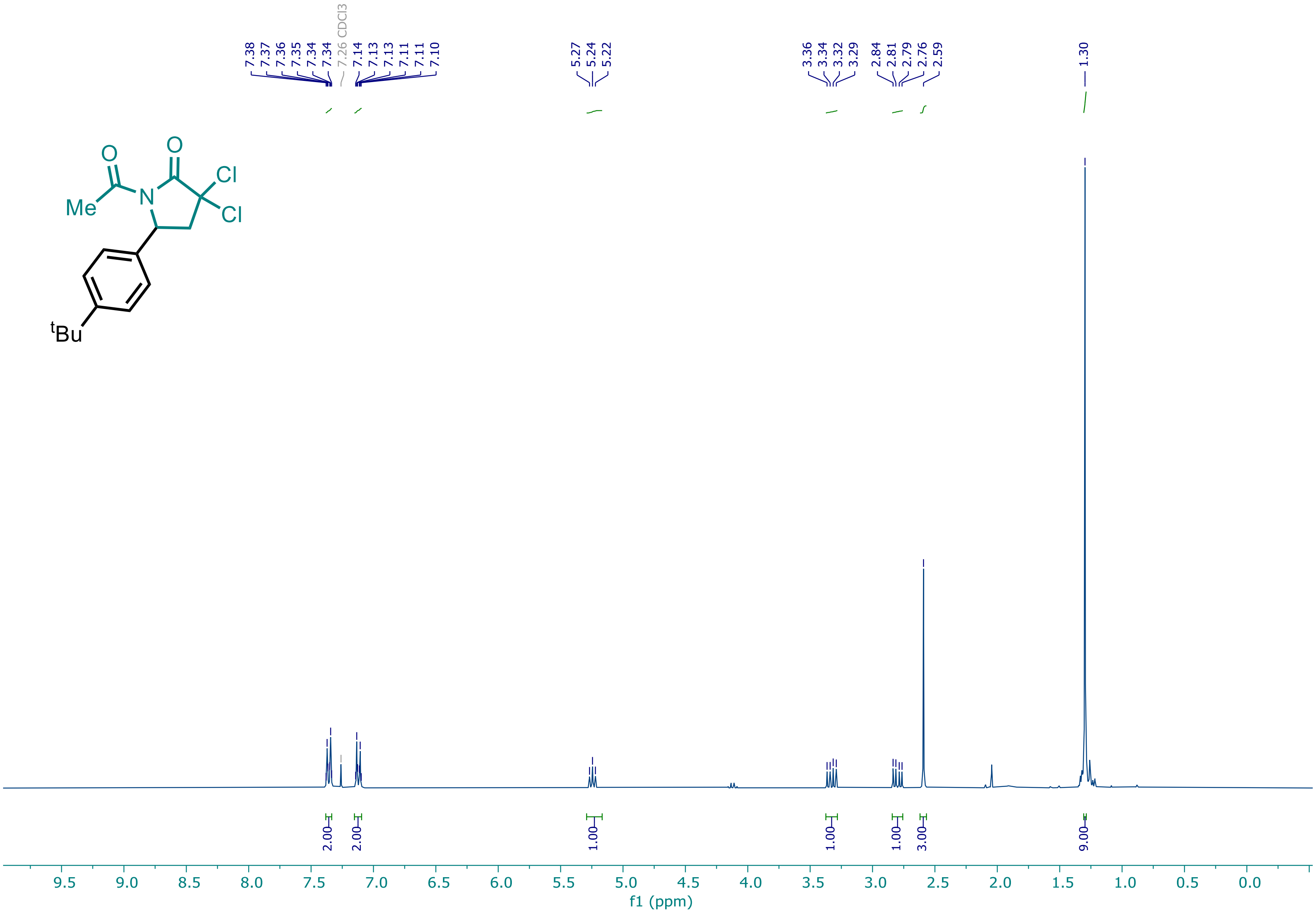

^{13}C NMR (75 MHz, CDCl$_3$) of **47**

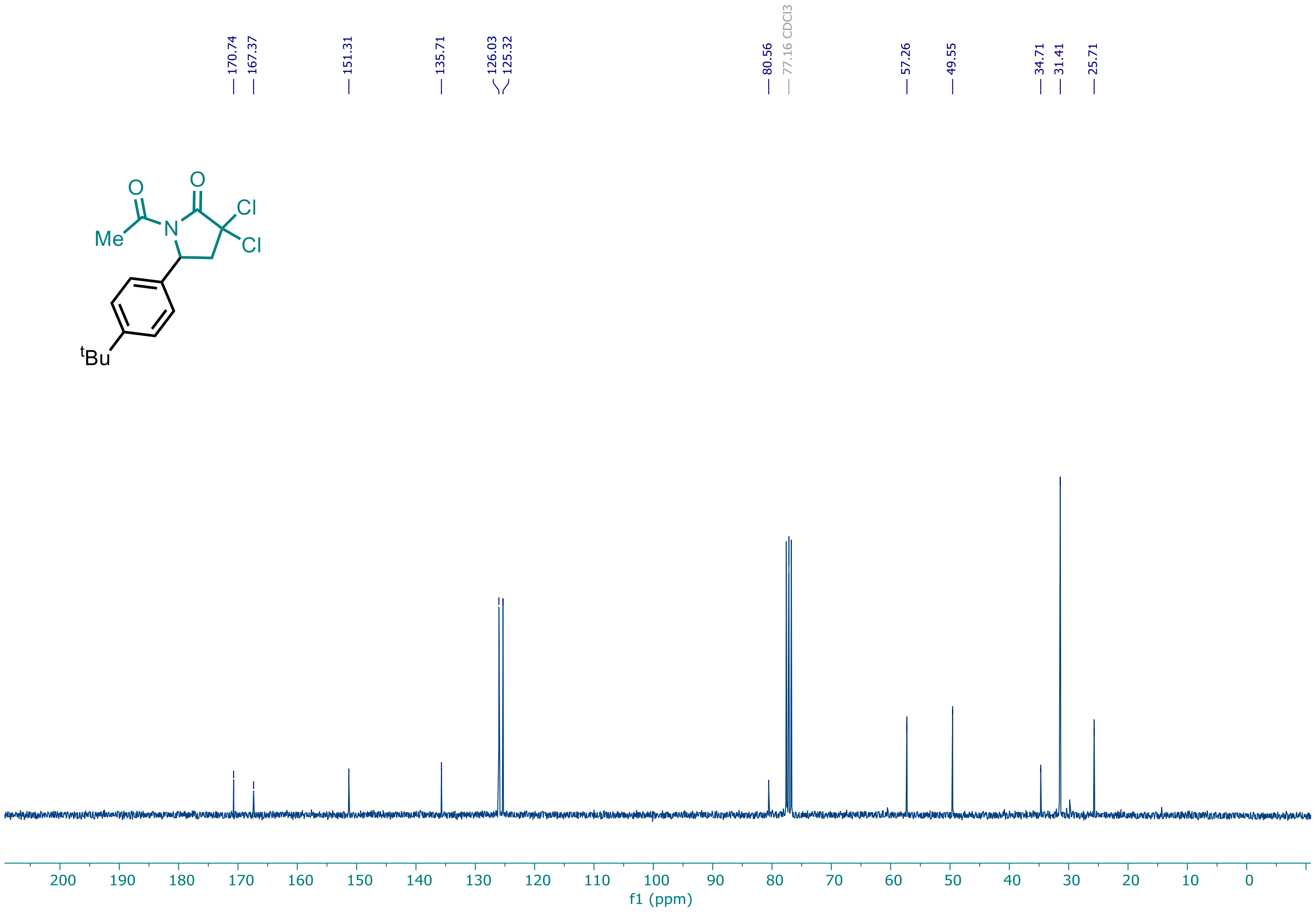

^{1}H NMR (300 MHz, CDCl3) of **48**

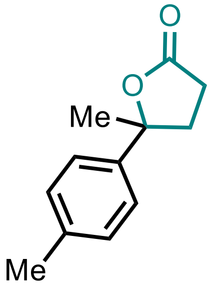

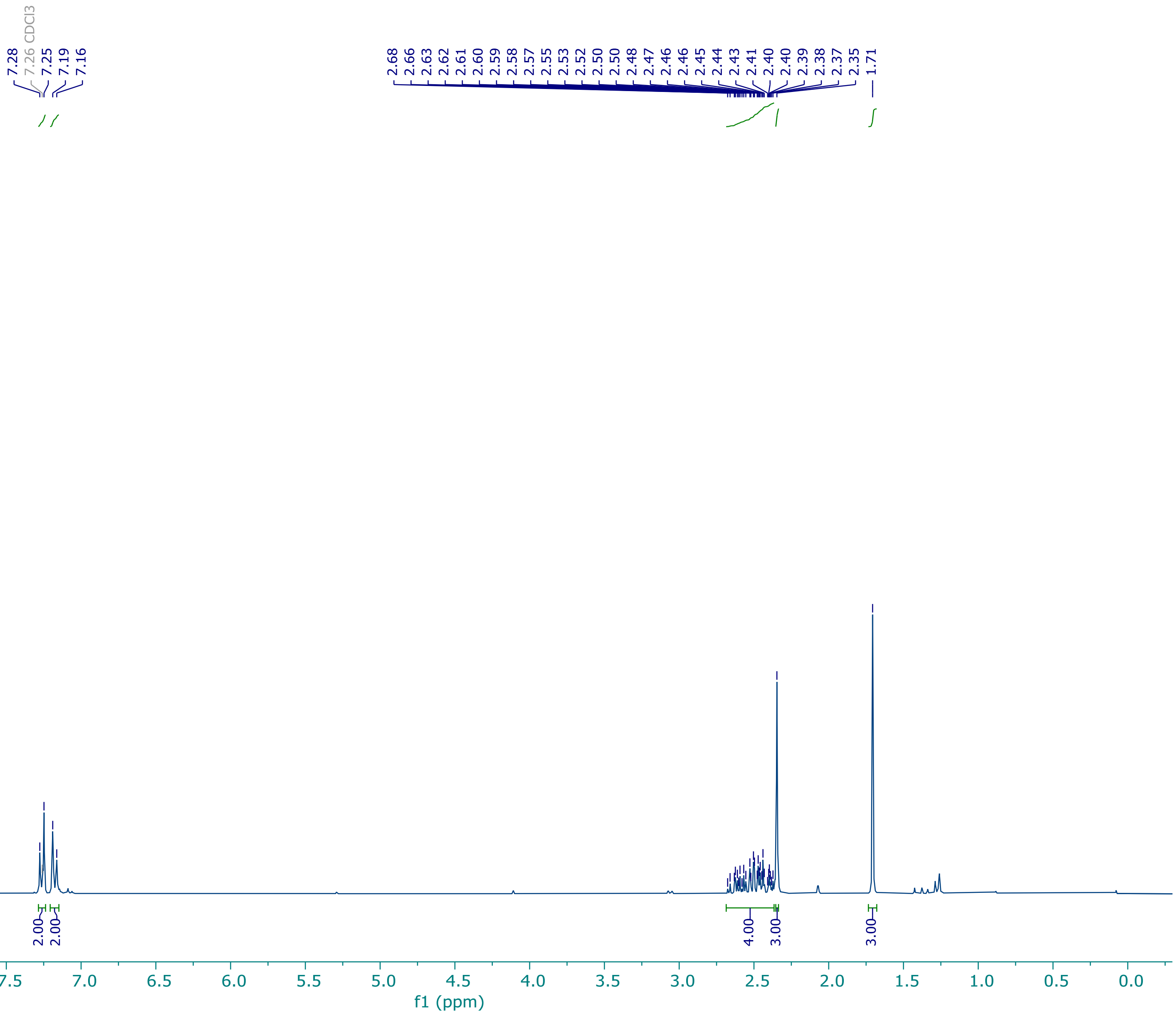

^{13}C NMR (75 MHz, $CDCl_3$) of **48**

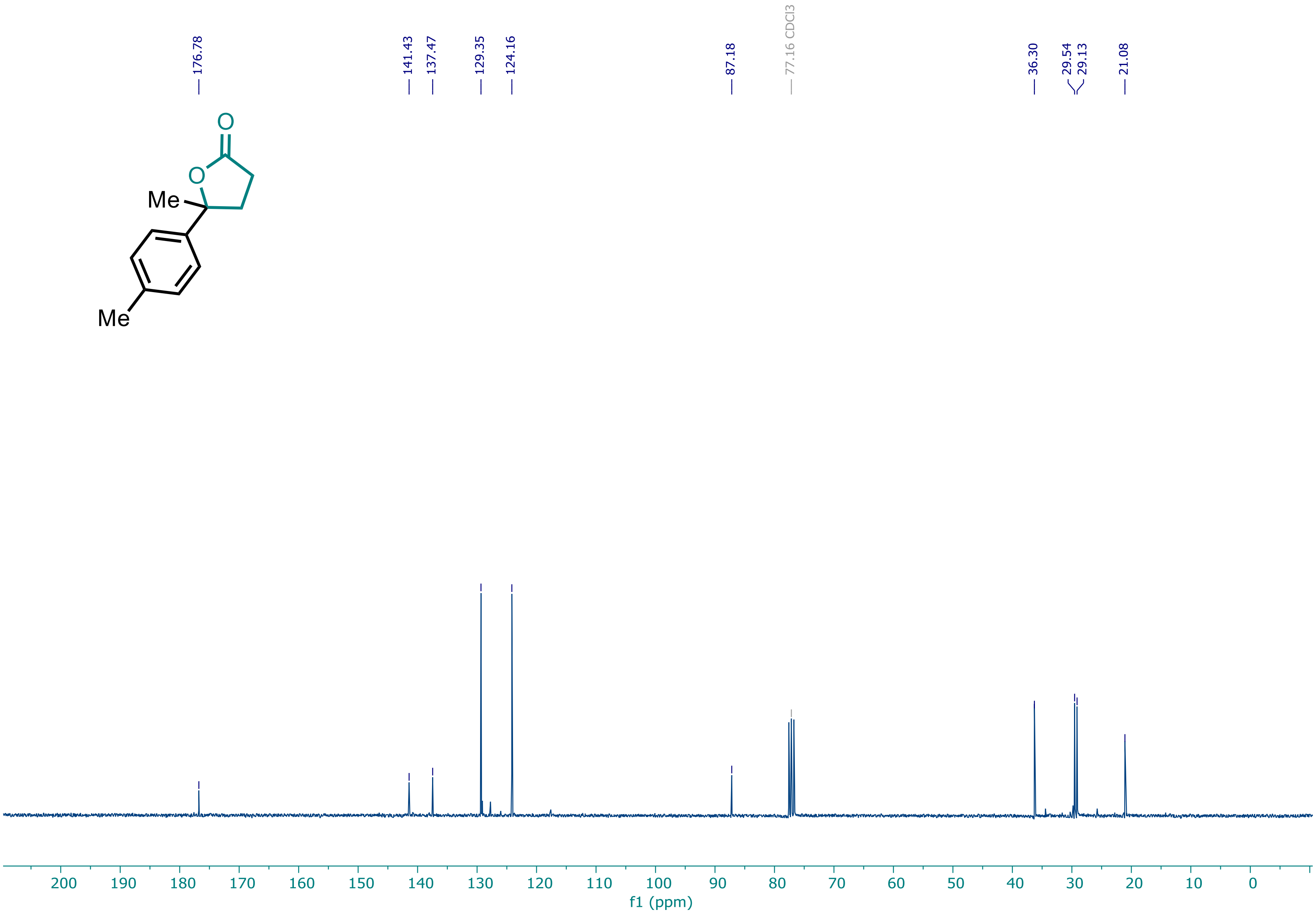

^{1}H NMR (300 MHz, $CDCl_3$) of **49**

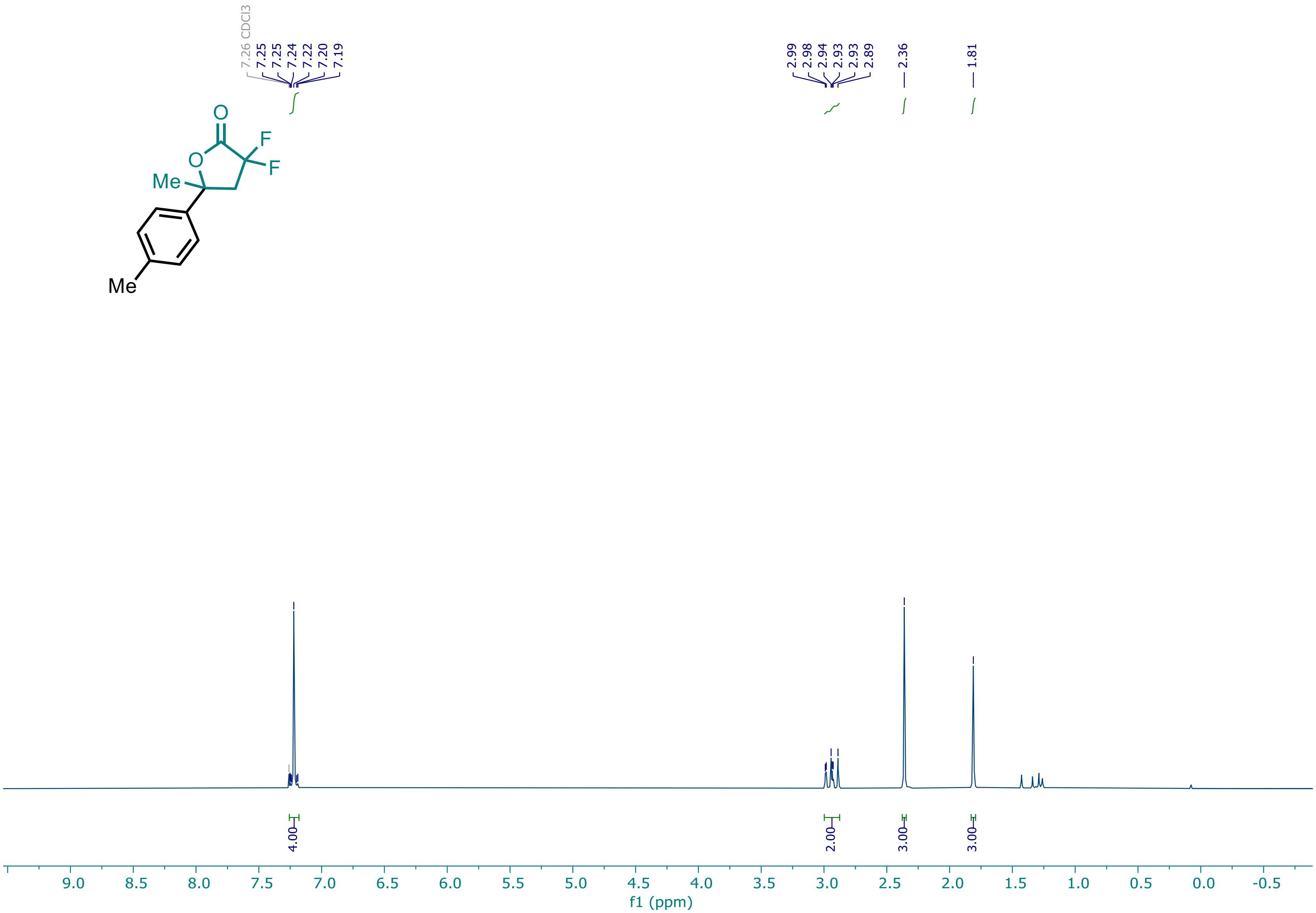

^{13}C NMR (75 MHz, $CDCl_3$) of **49**

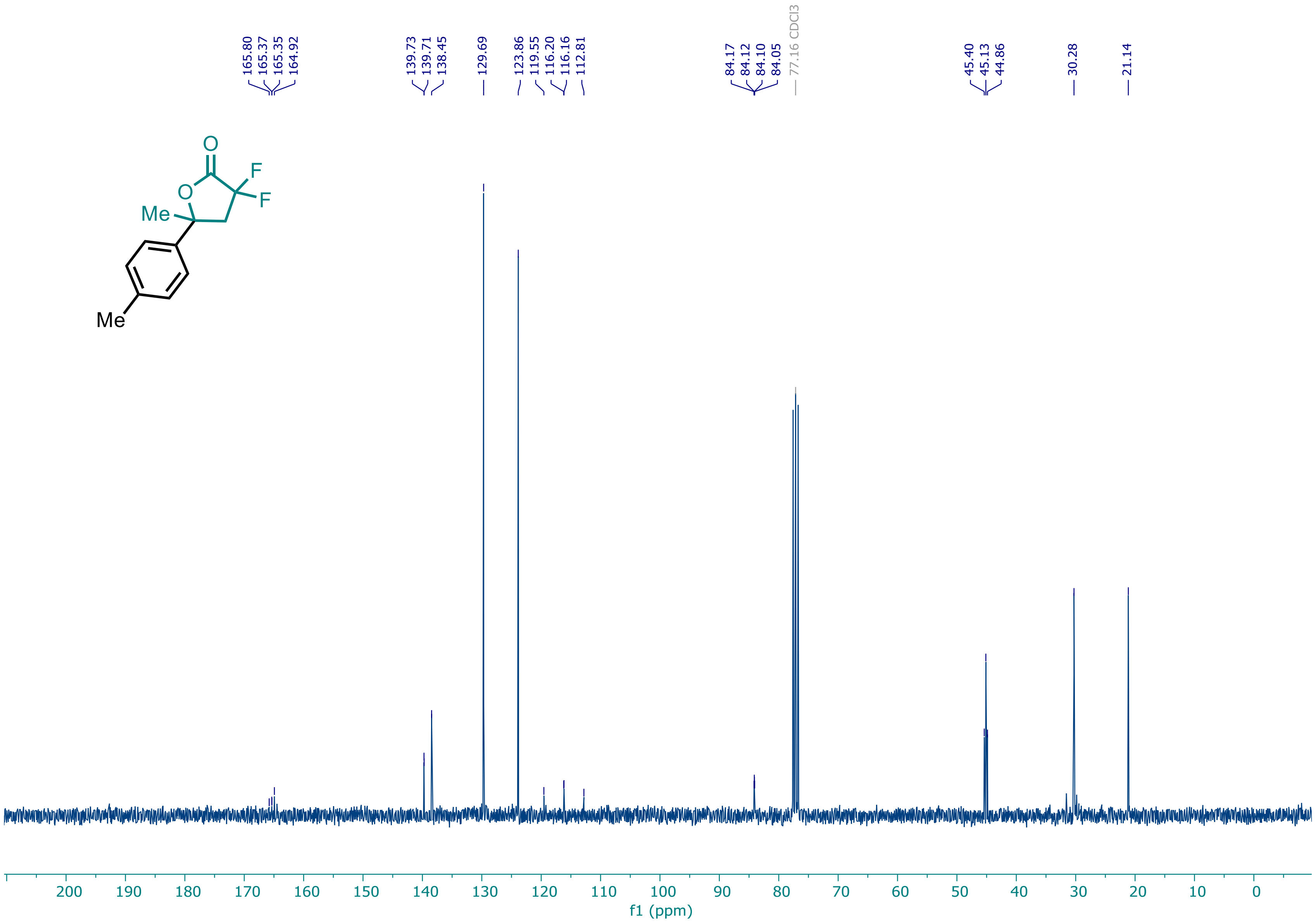

^{19}F NMR (282 MHz, $CDCl_3$) of **49**

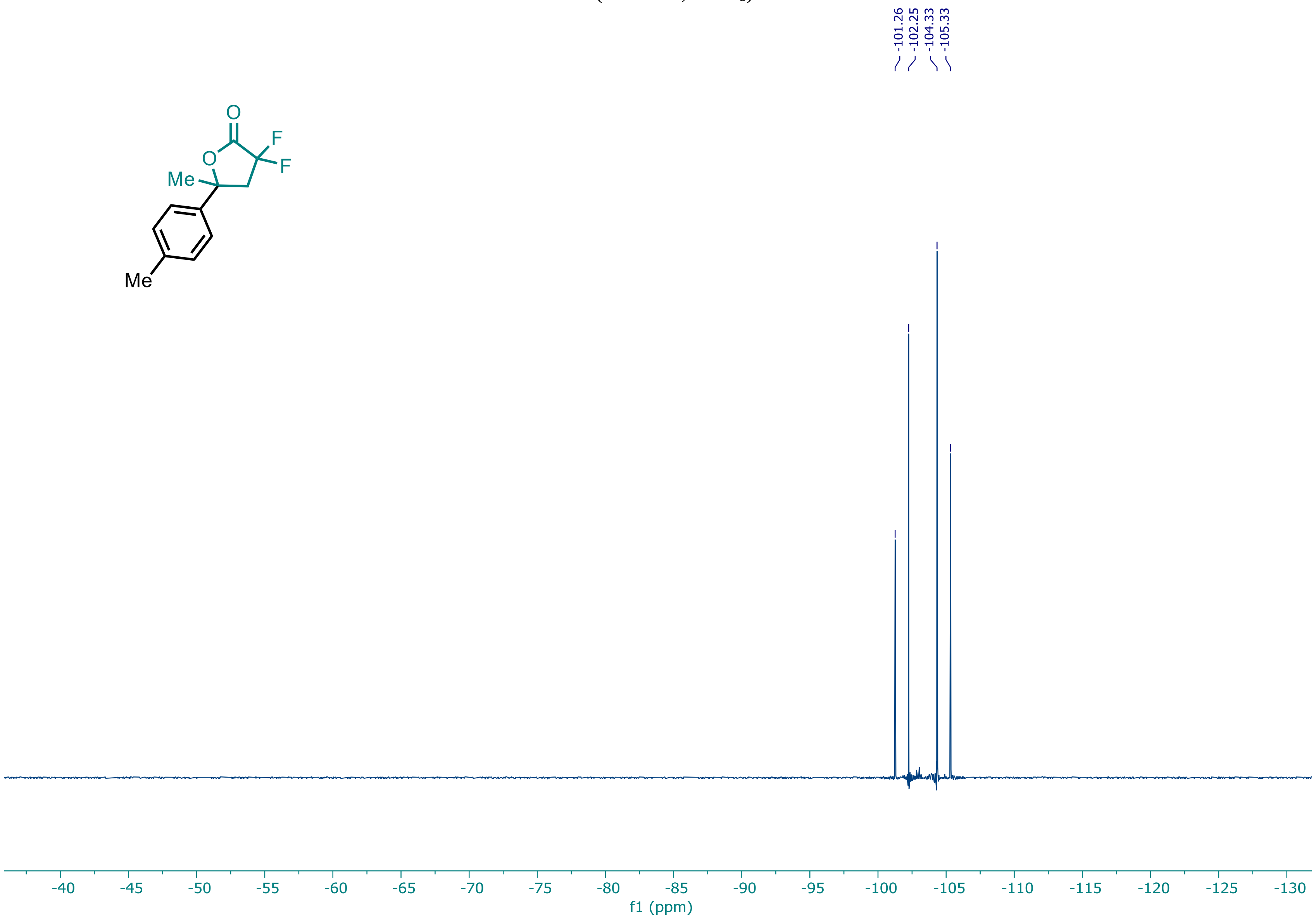

^{1}H NMR (300 MHz, $CDCl_3$) of **50**

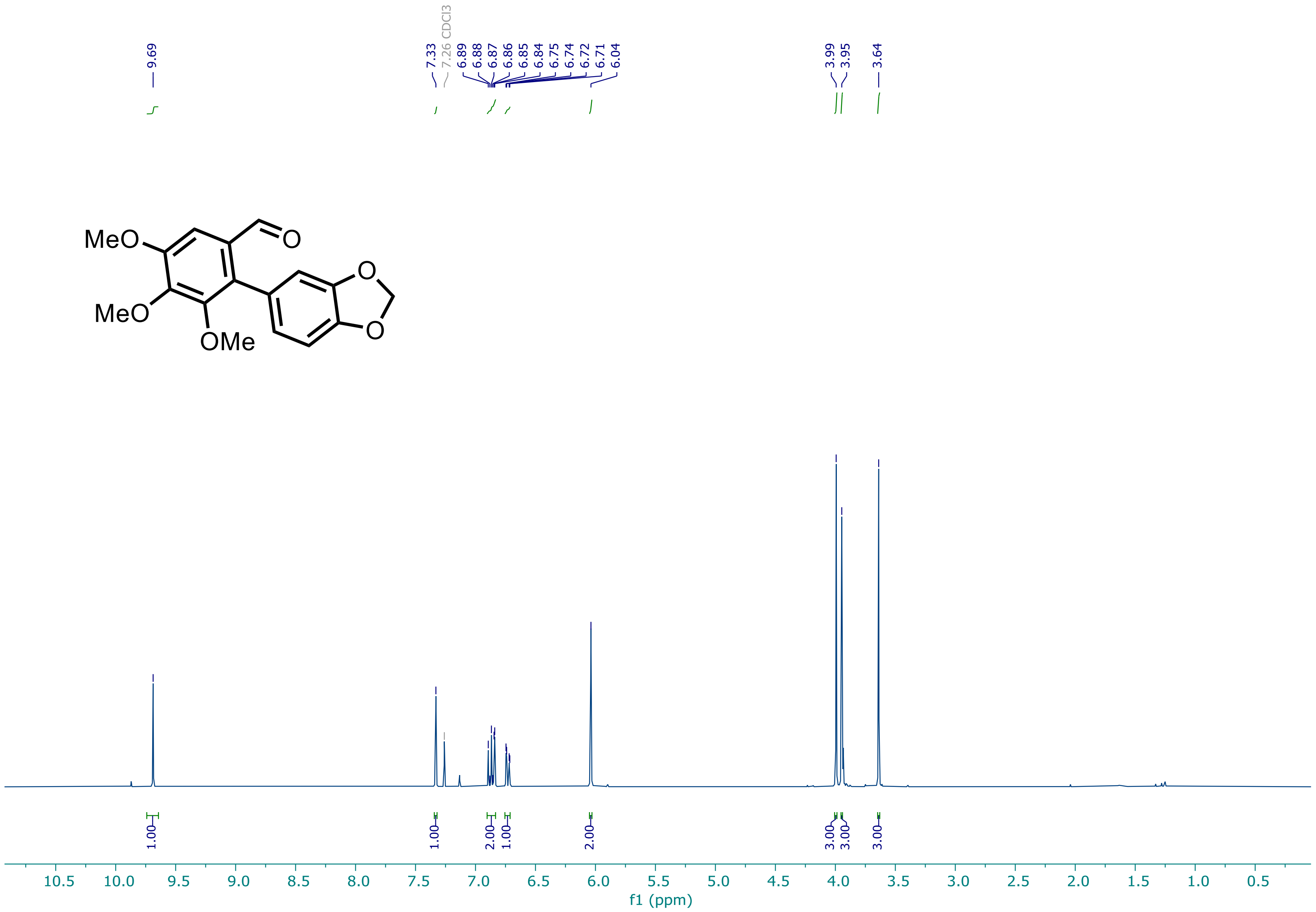

^{13}C NMR (75 MHz, $CDCl_3$) of **50**

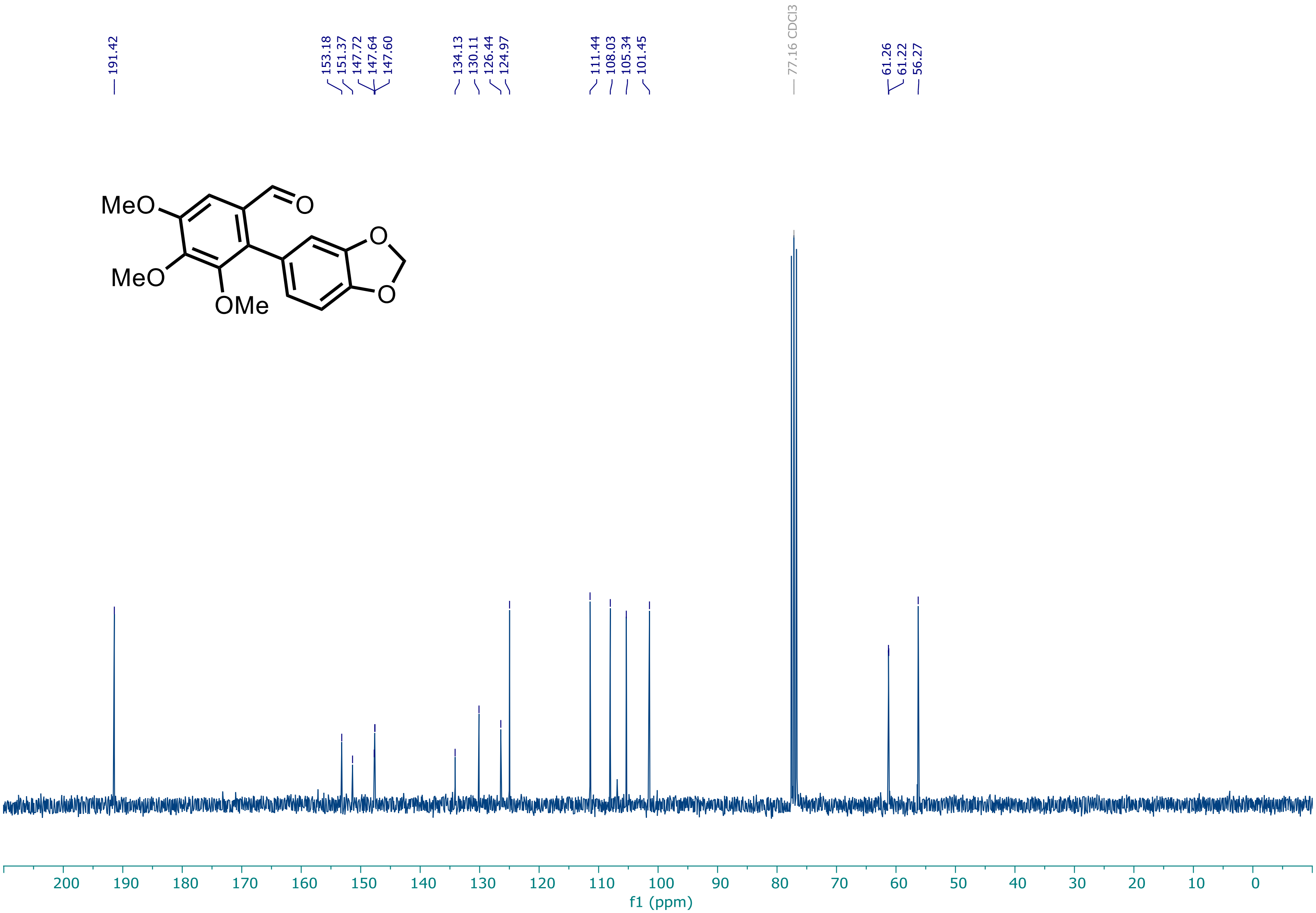

^{1}H NMR (300 MHz, $CDCl_3$) of **51**

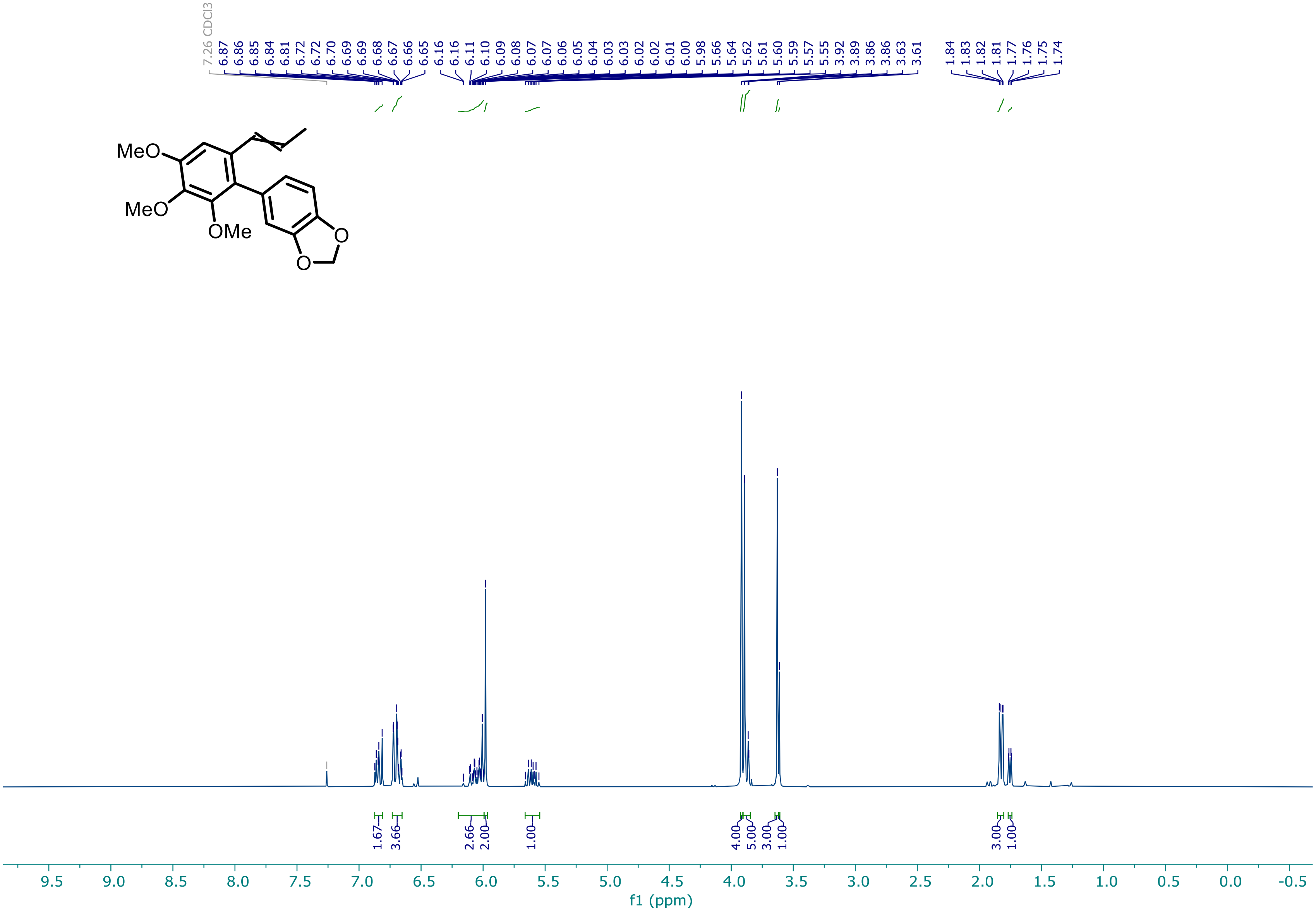

^{13}C NMR (75 MHz, $CDCl_3$) of **51**

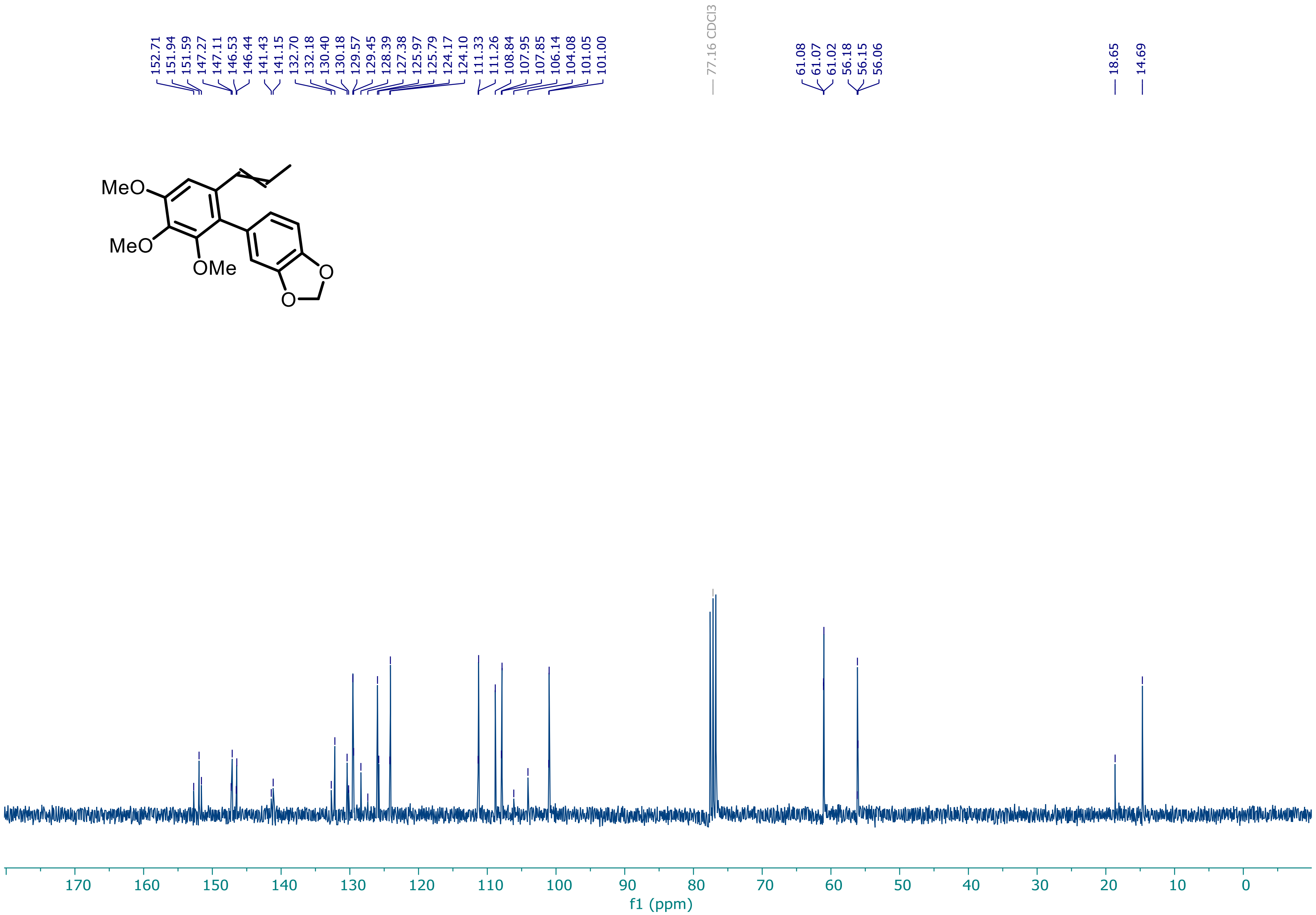

^{1}H NMR (400 MHz, $CDCl_3$) of **52**

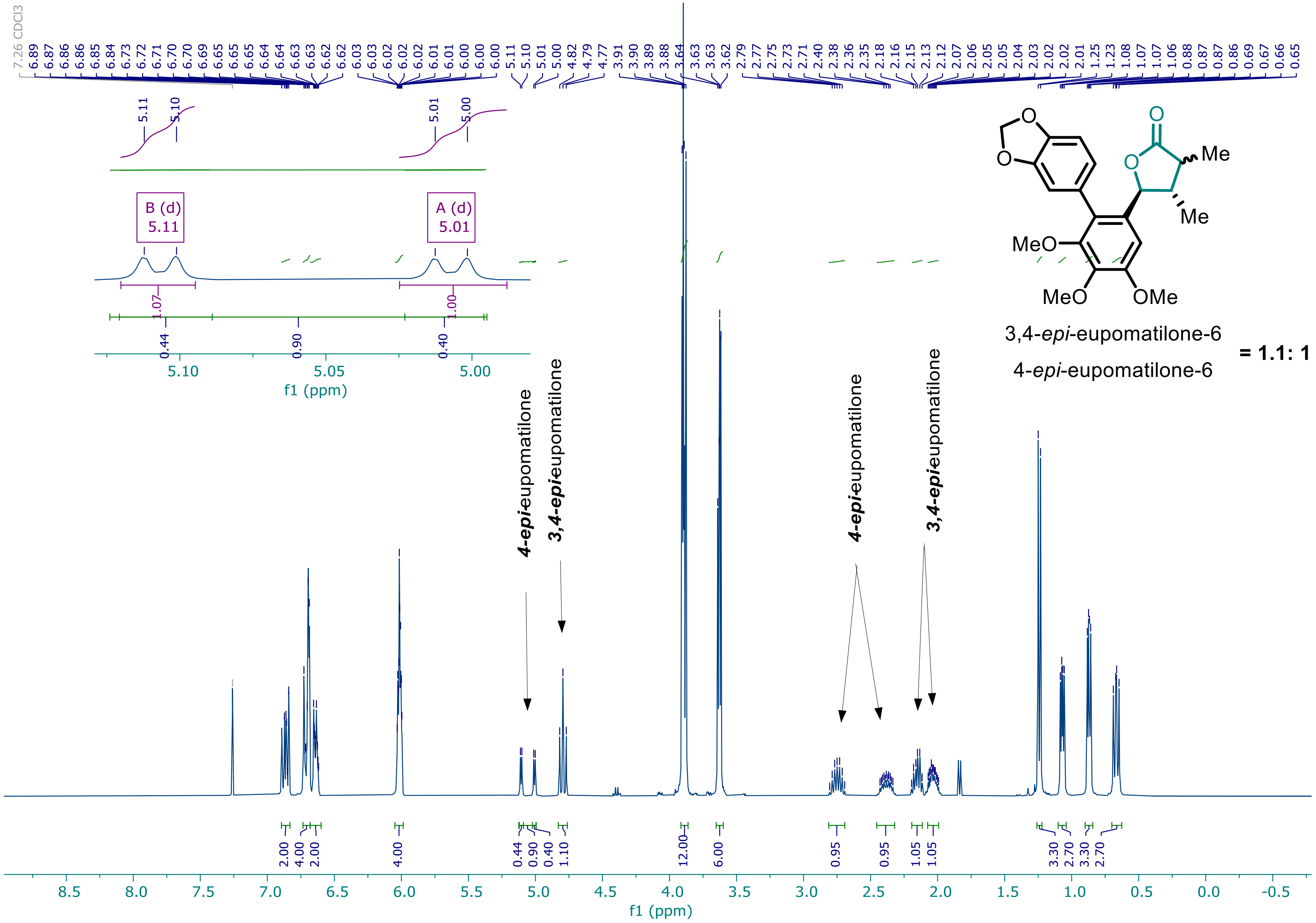

^{13}C NMR (101 MHz, CDCl$_3$) of **52**

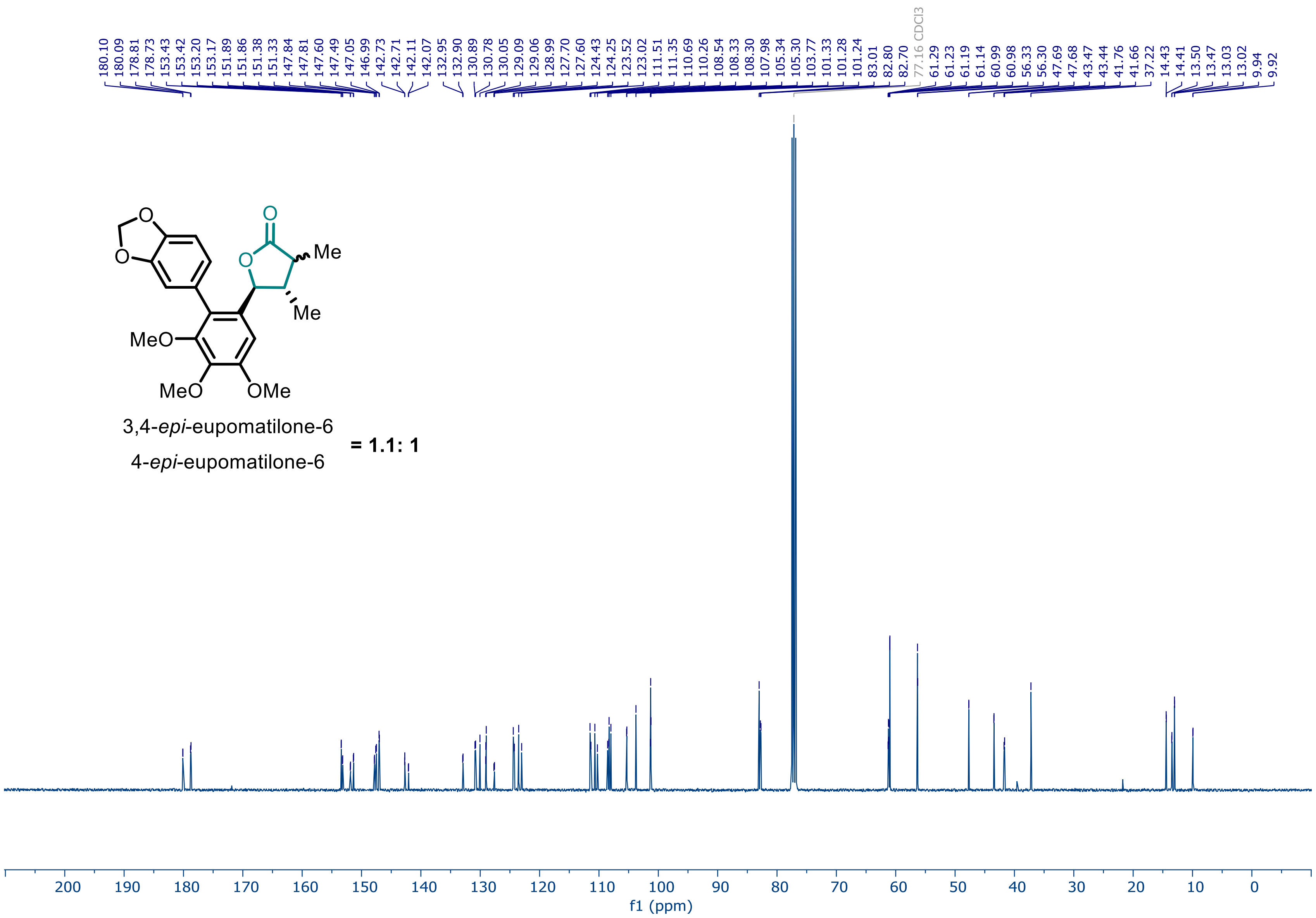

13. References

1. R. O. McCourt and E. M. Scanlan, *Org. Lett.*, 2019, **21**, 3460–3464.
2. J. L. Tu, W. Tang, W. Xu and F. Liu, *J. Org. Chem.*, 2021, **86**, 2929–2940.
3. S. Lauzon, H. Keipour, V. Gandon and T. Ollevier, *Org. Lett.*, 2017, **19**, 6324–6327.
4. C. Chatalova-Sazepin, Q. Wang, G. M. Sammis and J. Zhu, *Angew. Chem. Int. Ed.*, 2015, **54**, 5443–5446.
5. G. Z. Wang, R. Shang and Y. Fu, *Org. Lett.*, 2018, **20**, 888–891.
6. M. M. Amer, O. Olaizola, J. Carter, H. Abas and J. Clayden, *Org. Lett.*, 2020, **22**, 253–256.
7. Y. Chen, Y. Ma, L. Li, H. Jiang and Z. Li, *Org. Lett.*, 2019, **21**, 1480–1483.
8. **a**. S. Chen, J. Wang and L. G. Xie, *Org. Biomol. Chem.*, 2021, **19**, 4037–4042; **b**. D. A. Cruz, V. Sinka, P. de Armas, H. S. Steingruber, I. Fernández, V. S. Martín, P. O. Miranda and J. I. Padrón, *Org Lett*, 2021, **23**, 6105–6109.
9. G. J. Hedley, A. Ruseckas and I. D. W. Samuel, *Chem. Phys. Lett.*, 2008, **450**, 292–296.
10. K. C. Tang, K. L. Liu and I. C. Chen, *Chem. Phys. Lett.*, 2004, **386**, 437–441.
11. M. Kleinschmidt, C. van Wüllen and C. M. Marian, *J. Chem. Phys.*, 2015, **142**, 094301.
12. A. J. Fernandes, R. Giri, K. N. Houk and D. Katayev, *Angew. Chem. Int. Ed.*, 2024, e202318377.
13. R. Giri, I. Mosiagin, I. Franzoni, N. Yannick Nötel, S. Patra and D. Katayev, *Angew. Chem. Int. Ed.*, 2022, **61**, e202209143.
14. M. Zhang, Q. Li, J. H. Lin and J. C. Xiao, *Chin. J. Chem.*, 2023, **41**, 2819–2824.
15. V. S. Kostromitin, A. O. Sorokin, V. V Levin, and A. D. Dilman, *Chem. Sci.*, 2023,**14**, 3229-3234.
16. W. Yu, X. H. Xu and F. L. Qing, *Org Lett*, 2016, **18**, 5130–5133.
17. V. S. Kostromitin, A. O. Sorokin, V. V Levin, and A. D. Dilman, *Org. Lett*. 2023, **25**, 6598–6602.
18. R. Maji, S. Ghosh, O. Grossmann, P. Zhang, M. Leutzsch, N. Tsuji and B. List, *J. Am. Chem. Soc.*, 2023, **145**, 8788–8793
19. B. Sreedhar, D. Yada and P. S. Reddy *Adv. Synth. Catal.*, 2011, **353**, 2823 – 283
20. S. H. Yu, M. J. Ferguson, R. McDonald and D. G. Hall, *J. Am. Chem. Soc.*, 2005, **127**, 12808–12809..
21. Carroll R. Anthony and Taylor C. Walter, *Aust. J. Chem.*, 1991, **44**, 1705–1714.
22. Oxford Diffraction (2018). CrysAlisPro (Version 1.171.40.37a). Oxford Diffraction Ltd., Yarnton, Oxfordshire, UK.
23. G. M. Sheldrick, (2015). *Acta Cryst*., **A71**, 3–8.
24. G. M. Sheldrick, (2015). *Acta Cryst.*, **C71**, 3–8.
25. Dolomanov, O.V., Bourhis, L.J., Gildea, R.J, Howard, J.A.K. and Puschmann, H. *J. Appl. Cryst.*, 2009, **42**, 339–341.
